# Supplementary material for: SpaceBF: spatial coexpression analysis using Bayesian fused approaches in spatial omics datasets
Source: Gigascience. 2026 Jan 20;15:giag006. doi: 10.1093/gigascience/giag006 (PMC12954175; doi:10.1093/gigascience/giag006)
Supplement: giag006_Supplemental_File [file giag006_supplemental_file.pdf]

Supplementary Material for SpaceBF: Spatial coexpression analysis using Bayesian Fused approaches in spatial omics datasets

Souvik Seal and Brian Neelon

Department of Public Health Sciences, College of Medicine, Medical University of South Carolina, Charleston, USA

June 2025

1 A brief review of existing spatial priors

In the Bayesian framework, the spatial structure of the parameter vectors can be modeled in several ways:

1. *Gaussian process (GP) model*: We assume that $\beta_0^{mm'}(s)$ and $\beta_1^{mm'}(s)$ follow stationary, zero-centered Gaussian spatial process models [1] with covariance functions

$$\text{cov}(\beta_0^{mm'}(s), \beta_0^{mm'}(s')) = \sigma_0^2\, \rho_0(\|s - s'\|; \phi_0), \quad \text{cov}(\beta_1^{mm'}(s), \beta_1^{mm'}(s')) = \sigma_1^2\, \rho_1(\|s - s'\|; \phi_1),$$

where ρ_0 and ρ_1 are valid correlation functions with range (or decay) parameters ϕ_0, ϕ_1 and marginal variances σ_0^2, σ_1^2. Typical choices for ρ_k include (for a distance d)

   (a) the exponential correlation $\rho_j(d; \phi_j) = \exp(-d/\phi_j)$,

   (b) the squared-exponential (Gaussian) correlation $\rho_j(d; \phi_j) = \exp\{-d^2/(2\phi_j^2)\}$,

   (c) or a Matérn family with smoothness parameter ν_j, $\rho_j(d; \phi_j, \nu_j) = \frac{2^{1-\nu_j}}{\Gamma(\nu_j)} \left(\frac{d}{\phi_j}\right)^{\nu_j} K_{\nu_j}\left(\frac{d}{\phi_j}\right)$, where $K_{\nu_j}(\cdot)$ denotes the modified Bessel function of the second kind.

The joint distributions of $\boldsymbol{\beta}_0^{mm'} = (\beta_0^{mm'}(s_1), \ldots, \beta_0^{mm'}(s_n))^\top$ and $\boldsymbol{\beta}_1^{mm'} = (\beta_1^{mm'}(s_1), \ldots, \beta_1^{mm'}(s_n))^\top$ can be written as

$$\boldsymbol{\beta}_j^{mm'} \sim MVN(0, \sigma_j^2 H_j(\phi_j)), \;\; [[H_j(\phi_j)]]_{kk'} = \rho_j(||s_k - s_{k'}||, \phi_j), \;\; j \in \{0, 1\}.$$

2. *Conditionally autoregressive (CAR) model*: Let $W = [[W_{kk'}]]$ be a binary adjacency matrix between n locations, with $W_{kk'} = 1$ if locations s_k and $s_{k'}$ fall within a preset distance or 0 otherwise ($k \neq k'$). Let $W_{k+} = \sum_{k'} W_{kk'}$ be the k-th row-sum of W, and $D_w = diag(W_{k+})$. Univariate CAR priors on the two sets of coefficients can be written as

$$\beta_j^{mm'}(s_k)|\beta_j^{mm'}(s_k'), k' \neq k \sim N\left(p_j \sum_{k'} \frac{W_{kk'}}{W_{k+}} \beta_j^{mm'}(s_k'), \sigma_j^2 \frac{1}{W_{k+}}\right), \;\; k = 1, \ldots, n, \;\; j \in \{0, 1\},$$

where p_j's are "propriety" constants, both less than 1 and such that $(D_w - p_0 W)$ and $(D_w - p_1 W)$ are positive definite (PD). Using matrix notation, the distributions can be written as

$$\pi(\boldsymbol{\beta}_j^{mm'}|.) \propto \exp\left(-\frac{1}{2\sigma_j^2} \boldsymbol{\beta}_j^{mm',T}(D_w - p_j W)\boldsymbol{\beta}_j^{mm'}\right), \;\; j \in \{0, 1\}$$

$p_0 = p_1 = 1$ leads to a special class of priors known as intrinsic CAR (ICAR) prior. Since an ICAR prior distribution is improper (resulting posteriors are proper), a small diagonal adjustment to the precision matrix $(D_w - W)$ might improve performance [2, 3]. Unlike a GP prior that assumes an unobserved infinite process to model the continuous spatial dependence, the CAR and ICAR priors use a spatially defined graph structure, assuming a finite process on a discrete set of points.

3. *Gaussian Markov random field (GMRF)*: Let $G = (V, E)$ be an undirected neighborhood graph over the spatial locations, with $V = \{s_1, \ldots, s_n\}$ and $\{s_k, s_k'\} \in E$ indicates that locations s_k and $s_{k'}$ are neighbors. The Gaussian Markov property associated with G assumes that, for $k \neq k'$, the absence of an edge implies conditional independence:

$$\{s_k, s_{k'}\} \notin E \quad \Rightarrow \quad \beta_j^{mm'}(s_k) \perp\!\!\!\perp \beta_j^{mm'}(s_{k'}) \mid \{\beta_j^{mm'}(s_\ell) : \ell \neq k, k'\}, \;\; j \in \{0, 1\}.$$

Under Gaussianity, this is equivalent to sparsity in the precision matrix. Specifically, the GMRF

priors induced by G are given (up to a normalizing constant) by

$$\pi(\boldsymbol{\beta}_j^{mm'} \mid \cdot) \propto \exp\left\{-\frac{1}{2\sigma_j^2}\boldsymbol{\beta}_j^{mm'\top} Q_j \boldsymbol{\beta}_j^{mm'}\right\}, \ (Q_j)_{kk'} = 0 \text{ for } k \neq k' \text{ whenever } \{s_k, s_{k'}\} \notin E,$$

where Q_j's are (possibly singular) precision matrices encoding the conditional dependence structure implied by G. The CAR prior is a special case of a GMRF prior. In fact, *the pairwise difference GMRF prior* [4] leads to the following density

$$\pi(\boldsymbol{\beta}_j^{mm'}|.) \propto \exp\left(-\frac{1}{2\sigma_j^2}\sum_{(s_k,s_{k'})\in E}(\beta_j^{mm'}(s_k) - \beta_j^{mm'}(s_{k'}))^2\right), \ j \in \{0,1\}. \tag{1}$$

which are the same as ICAR priors [1]. Of note, replacing the L^2 distance with the L^1 distance or any general even function $\Phi(z)$, increasing w.r.t. $|z|$, is briefly discussed in Besag (1991) [5]. Our proposed spatial horseshoe prior can also be viewed as a GMRF with an unfixed (random) precision structure, as discussed in the main text and Section 2.5 of this text.

In the above specifications and throughout this manuscript, we have assumed $\boldsymbol{\beta}_0^{mm'}$ and $\boldsymbol{\beta}_1^{mm'}$ are a priori independent, thereby ignoring any correlation between them; such dependence can be accommodated, for example, by adopting a bivariate (multivariate) CAR prior [6]. The GP models are computationally expensive ($O(n^3)$) and often require further approximations, such as predictive process [7] and nearest neighbor Gaussian process [8], to be applicable on high-dimensional datasets. The GMRF and CAR priors are computationally more tractable, with further potential for scalability through the use of advanced approximation techniques, such as the integrated nested Laplace approximation (INLA) [4].

2 More discussions on the proposed approach

Let $G = (V, E)$ denote the MST network (or a general adjacency network) between the locations constructed using the L^2 distance, where V and E denote the sets of vertices and edges, respectively, with $|E| = p$.

2.1 Spatial fused lasso and its connection to GMRF

Following the main text, for a pair of locations $(s_{k_i^1}, s_{k_i^2})$ connected by edge $i \in E$, let $\Delta\beta_i^{(j)} \equiv \beta_j^{mm'}(s_{k_i^1}) - \beta_j^{mm'}(s_{k_i^2})$ denote the difference in coefficients, for $j \in \{0, 1\}$. The fused lasso prior can be written as

$$\begin{aligned}
\pi(\boldsymbol{\beta}_0^{mm'}|\ldots) &\propto \prod_{i\in E} \exp\left(-\frac{\lambda_0}{\sigma}\Delta\beta_i^{(0)}\right), \ \boldsymbol{\beta}_0^{mm'} = (\beta_0^{mm'}(s_1), \ldots, \beta_0^{mm'}(s_n))^\top, \\
\pi(\boldsymbol{\beta}_1^{mm'}|\ldots) &\propto \prod_{i\in E} \exp\left(-\frac{\lambda_1}{\sigma}\Delta\beta_i^{(1)}\right), \ \boldsymbol{\beta}_1^{mm'} = (\beta_1^{mm'}(s_1), \ldots, \beta_1^{mm'}(s_n))^\top,
\end{aligned} \tag{2}$$

where σ^2 is the variance of the error term $\epsilon(s_k)$, present only in the Gaussian model. Scaling by σ inside the exponent in Eq. 2 follows Park and Casella (2008) [9], who demonstrated in the Bayesian lasso setting that this scaling is needed to obtain a globally optimal solution. In our simulation studies, however, its impact on performance was minimal. From a modeling perspective, an L^1 distance is expected to be more effective than an L^2 distance for inducing exact fusion of coefficients at nearby spatial locations. Indeed, Besag (1991; see Eq. 4.4) [5] recommends such a specification "if discontinuities in the risk surface are expected." In spatial omics applications, sharp transitions or boundaries in the interaction surface are biologically plausible (e.g., across tissue compartments), which further motivates the use of an L^1-based construction.

For the posterior sampling, the Laplacian likelihood can be expanded as a superposition of an infinite number of Gaussian distributions [10],

$$\frac{\sqrt{\lambda}}{2}\exp\left[-\sqrt{\lambda}|x|\right] = \int_0^\infty \sqrt{\frac{1}{2\pi\zeta}}\exp\left[-\frac{x^2}{2\zeta}\right]\frac{\lambda}{2}\exp\left[-\frac{\lambda\zeta}{2}\right]d\zeta$$

Suppose there are p edges in E. Exploiting the above property, we introduce two new latent mixing vectors of length p as $\boldsymbol{\zeta}_0 = (\zeta_{10}, \ldots, \zeta_{p0})^\top$ and $\boldsymbol{\zeta}_1 = (\zeta_{11}, \ldots, \zeta_{p1})^\top$, where ζ_{i0} and ζ_{i1} correspond to $\Delta\beta_i^{(0)}$ and $\Delta\beta_i^{(1)}$, respectively. We then write the conditional prior of $(\boldsymbol{\beta}_0^{mm'}, \boldsymbol{\beta}_1^{mm'})$ as

$$\begin{aligned}
\pi(\boldsymbol{\beta}_0^{mm'}|\boldsymbol{\zeta}_0, \sigma^2, .) &= \prod_{i=1}^{p} \sqrt{\frac{1}{2\pi\zeta_{i0}\sigma^2}}\exp\left[-\frac{\left(\beta_0^{mm'}(s_{k_i^1}) - \beta_0^{mm'}(s_{k_i^2})\right)^2}{2\zeta_{i0}\sigma^2}\right] \\
\pi(\boldsymbol{\beta}_1^{mm'}|\boldsymbol{\zeta}_1, \sigma^2, .) &= \prod_{i=1}^{p} \sqrt{\frac{1}{2\pi\zeta_{i1}\sigma^2}}\exp\left[-\frac{\left(\beta_1^{mm'}(s_{k_i^1}) - \beta_1^{mm'}(s_{k_i^2})\right)^2}{2\zeta_{i1}\sigma^2}\right]
\end{aligned} \tag{3}$$

The mixture components follow the joint distribution $\pi(\boldsymbol{\zeta}_0, \boldsymbol{\zeta}_1) = \prod_{i=1}^{p}\left[\frac{\lambda_0}{2}\frac{\lambda_1}{2}\exp\left[-\frac{\lambda_0\zeta_{i0}}{2} - \frac{\lambda_1\zeta_{i1}}{2}\right]\right]$. We

notice that Eq. 3 is a weighted version of the pairwise difference GMRF prior or ICAR prior from Eq. 1.

2.2 MCMC scheme for both priors in the Gaussian model

For notational simplicity, we omit the covariate vector $Z(s_k)$ and its associated coefficient vector α_m in the derivations below; these terms can be incorporated into the framework in a straightforward way.

2.2.1 Spatial fused lasso

We multiply the densities from Eq. 3 and simplify using a matrix notation as

$$\pi(\boldsymbol{\beta}^{mm'}|.) \propto \left[\frac{1}{\prod_{i=1}^{p}\sqrt{\zeta_{i0}\zeta_{i1}}}\right] \exp\left[-\frac{1}{2\sigma^2}(\boldsymbol{\beta}^{mm'})^\top B\boldsymbol{\beta}^{mm'}\right] \tag{4}$$

where $\boldsymbol{\beta}^{mm'} = (\boldsymbol{\beta}_0^{mm'}, \boldsymbol{\beta}_1^{mm'})^\top$ and B is a block-diagonal matrix with two blocks B_0 and B_1 defined as

$$[B_0]_{k_i^1,k_i^2} = [B_0]_{k_i^2,k_i^1} = -\zeta_{i0}^{-1}, \qquad [B_1]_{k_i^1,k_i^2} = [B_1]_{k_i^2,k_i^1} = -\zeta_{i1}^{-1}, \qquad i = 1,\dots,p,$$

with $[B_0]_{k,k'} = [B_1]_{k,k'} = 0$ for all other off-diagonal pairs $(k \neq k')$,

$$[B_0]_{k,k} = -\sum_{k'\neq k}[B_0]_{k,k'}, \qquad [B_1]_{k,k} = -\sum_{k'\neq k}[B_1]_{k,k'}.$$

If σ^2 is omitted from Eqs. 3 and 4, we can redefine B as $\sigma^2 B$ and keep the subsequent derivation as it is. Assuming $\epsilon(s_k)$'s follow IID $N(0,\sigma^2)$, we write the distribution of $\mathbf{X}^m$, without the priors, as

$$p(\mathbf{X}^m|\mathbf{X}^{*m'}, \boldsymbol{\beta}^{mm'}, \sigma^2) \propto \exp\left[-\frac{1}{2\sigma^2}((\mathbf{X}^m - \mathbf{X}^{*m'}\boldsymbol{\beta}^{mm'})^\top(\mathbf{X}^m - \mathbf{X}^{*m'}\boldsymbol{\beta}^{mm'})\right], \tag{5}$$

where $\mathbf{X}^{*m'} = [I_n,\ diag(\mathbf{X}^{m'})]_{n\times 2n}$, where I_n is the identity matrix of dimension n. Now, the conditional posterior distributions for a Gibbs sampling are:

1. $$p(\boldsymbol{\beta}^{mm'}|.) \propto \exp\left[-\frac{1}{2\sigma^2}(\boldsymbol{\beta}^{mm'} - M)^\top\Sigma^{-1}(\boldsymbol{\beta}^{mm'} - M)\right],$$
$$\Sigma = \left(B + (\mathbf{X}^{*m'})^\top\mathbf{X}^{*m'}\right)^{-1}, \ M = \Sigma\left((\mathbf{X}^{*m'})^\top\mathbf{X}^m\right)$$

2.
$$p(\zeta_{i0}|.) \propto \zeta_{i0}^{-1/2} \exp\left[-\frac{1}{2} \left(\frac{\left(\beta_0^{mm'}(s_{k_i^1}) - \beta_0^{mm'}(s_{k_i^2})\right)^2}{\zeta_{i0}\sigma^2} + \lambda_0 \zeta_{i0} \right) \right]$$
$$p(\zeta_{i1}|.) \propto \zeta_{i1}^{-1/2} \exp\left[-\frac{1}{2} \left(\frac{\left(\beta_1^{mm'}(s_{k_i^1}) - \beta_1^{mm'}(s_{k_i^2})\right)^2}{\zeta_{i1}\sigma^2} + \lambda_1 \zeta_{i1} \right) \right]$$

3. Assuming an improper prior on σ^2: $\pi(\sigma^2) = 1/\sigma^2$,

$$p(\sigma^2|.) \propto \left(\frac{1}{\sigma^2}\right)^{n/2+(2n)/2+1} \exp\left[-\frac{1}{2\sigma^2}((\mathbf{X}^m - \mathbf{X}^{*m'}\boldsymbol{\beta}^{mm'})^\top (\mathbf{X}^m - \mathbf{X}^{*m'}\boldsymbol{\beta}^{mm'}) + (\boldsymbol{\beta}^{mm'})^\top B \boldsymbol{\beta}^{mm'}) \right]$$

4. Putting a gamma prior with hyper-parameters δ_{1s}, δ_{2s} on the shrinkage parameter λ_s, the corresponding posterior distribution can be derived as

$$\pi(\lambda_s|\delta_{1s}, \delta_{2s}) = \frac{\delta_{1s}^{\delta_{2s}}}{\Gamma(\delta_{2s})} \lambda_s^{\delta_{2s}-1} \exp(-\delta_{1s}\lambda_s), \text{ for } s = 0, 1,$$
$$p(\lambda_s|.) \propto \left[\lambda_s^{p+\delta_{2s}-1}\right] \exp\left[-\lambda_s \left(\delta_{1s} + \frac{1}{2} \sum_{i=1}^{p} \zeta_{is} \right) \right],$$

2.2.2 Spatial fused horseshoe

To derive the Gibbs sampling algorithm for spatial fused horseshoe, we introduce new latent variables, $\gamma_{0i}, \gamma_{1i}, \epsilon_0, \epsilon_1$ to write the half-Cauchy priors as mixtures of inverse gamma (IG) priors [11] as below

$$\begin{aligned} \Lambda_{0i}^2|\gamma_{0i} &\sim IG\left(\frac{1}{2}, \frac{1}{\gamma_{0i}}\right), & \Lambda_{1i}^2|\gamma_{1i} &\sim IG\left(\frac{1}{2}, \frac{1}{\gamma_{1i}}\right), \\ \gamma_{0i} &\sim IG\left(\frac{1}{2}, 1\right), & \gamma_{1i} &\sim IG\left(\frac{1}{2}, 1\right), \\ \tau_0^2|\epsilon_0 &\sim IG\left(\frac{1}{2}, \frac{1}{\epsilon_0}\right), & \tau_1^2|\epsilon_1 &\sim IG\left(\frac{1}{2}, \frac{1}{\epsilon_1}\right), \\ \epsilon_0 &\sim IG\left(\frac{1}{2}, 1\right), & \epsilon_1 &\sim IG\left(\frac{1}{2}, 1\right). \end{aligned}$$

We redefine the matrix B from the previous section with new B_0 and B_1 as

$$[B_0]_{k_i^1,k_i^2} = [B_0]_{k_i^2,k_i^1} = -(\tau_0^2\Lambda_{0i}^2)^{-1}, \; [B_1]_{k_i^1,k_i^2} = [B_1]_{k_i^2,k_i^1} = -(\tau_1^2\Lambda_{1i}^2)^{-1},$$
$$[B_0]_{k,k} = -\sum_{k'\neq k} [B_0]_{k,k'}, \; [B_1]_{k,k} = -\sum_{k'\neq k} [B_1]_{k,k'}.$$

The required conditional posterior distributions for a Gibbs sampling are

1. Posterior distributions highlighted in points 1-3 from Section 2.2.1.

2.
$$\Lambda_{0i}^2|. \sim IG\left(1, \frac{1}{\gamma_{0i}} + \frac{\left(\beta_0^{mm'}(s_{k_i^1}) - \beta_0^{mm'}(s_{k_i^2})\right)^2}{2\tau_0^2\sigma^2}\right), \quad \gamma_{0i}|. \sim IG\left(1, 1 + \frac{1}{\Lambda_{0i}^2}\right),$$

$$\tau_0^2|. \sim IG\left(\frac{p+1}{2}, \frac{1}{\epsilon_0} + \sum_{i=1}^{p} \frac{\left(\beta_0^{mm'}(s_{k_i^1}) - \beta_0^{mm'}(s_{k_i^2})\right)^2}{2\Lambda_{0i}^2\sigma^2}\right), \quad \epsilon_0|. \sim IG\left(1, 1 + \frac{1}{\tau_0^2}\right),$$

$$\Lambda_{1i}^2|. \sim IG\left(1, \frac{1}{\gamma_{1i}} + \frac{\left(\beta_1^{mm'}(s_{k_i^1}) - \beta_1^{mm'}(s_{k_i^2})\right)^2}{2\tau_1^2\sigma^2}\right), \quad \gamma_{1i}|. \sim IG\left(1, 1 + \frac{1}{\Lambda_{1i}^2}\right),$$

$$\tau_1^2|. \sim IG\left(\frac{p+1}{2}, \frac{1}{\epsilon_1} + \sum_{i=1}^{p} \frac{\left(\beta_1^{mm'}(s_{k_i^1}) - \beta_1^{mm'}(s_{k_i^2})\right)^2}{2\Lambda_{1i}^2\sigma^2}\right), \quad \epsilon_1|. \sim IG\left(1, 1 + \frac{1}{\tau_1^2}\right).$$

In both MCMC algorithms above, we sample $\boldsymbol{\beta}^{mm'} = (\boldsymbol{\beta}_0^{mm'}, \boldsymbol{\beta}_1^{mm'})^\top$ jointly. However, under our prior specification the two components are independent, so their full conditional distributions can be derived and sampled separately, which is computationally more efficient.

2.2.3 MCMC scheme for an NB model using Pólya-gamma augmentation

For the NB model, we use a data-augmented Gibbs sampler based on Pillow and Scott (2012) [12] and Polson et al. (2013) [13]. For $b > 0$ and $c \in \mathbb{R}$, a random variable M is said to have a PG distribution if

$$M \sim PG(b, c) = \frac{1}{2\pi^2} \sum_{g=1}^{\infty} \frac{d_g}{(g - 1/2)^2 + c^2/(4\pi^2)}$$

where d_g's are independent $Gamma(b, 1)$ random variables. Introducing latent Pólya-Gamma (PG)-distributed weights w_k, for $k = 1, \ldots, n$, the NB mass function (Eq. 2 from the main text) can be expanded as a mixture of distributions as

$$p(X^m(s_k)|\psi_m(s_k), r_m) \propto \exp(\kappa_k \eta_m(s_k)) \int_0^\infty \exp(-w_k \eta_m(s_k)^2/2) p(w_k|X^m(s_k) + r_m, 0) dw_k,$$

where $\kappa_k = (X^m(s_k) - r_m)/2$, w_k's are independently distributed as $PG(X^m(s_k) + r_m, \eta_m(s_k))$, and $p(w_k|X^m(s_k) + r_m, 0)$ denotes the $PG(X^m(s_k) + r_m, 0)$ density. Then, for given w_k's, defining $y_k = \frac{X^m(s_k)-r_m}{2w_k}$, we can write the posterior sampling distribution of $\boldsymbol{\beta}^{mm'}$ as

$$p(\boldsymbol{\beta}^{mm'}|.) \propto \pi(\boldsymbol{\beta}^{mm'}|.) \exp\left[-\frac{1}{2}(\mathbf{Y} - \mathbf{X}^{*m'}\boldsymbol{\beta}^{mm'})^\top \Omega(\mathbf{Y} - \mathbf{X}^{*m'}\boldsymbol{\beta}^{mm'})\right], \ \Omega = diag(\mathbf{w}),$$

where $\mathbf{Y} = (y_1, \ldots, y_n)^\top$ and $\mathbf{w} = (w_1, \ldots, w_n)^\top$, and $\pi(\boldsymbol{\beta}^{mm'}|.)$ is the joint prior from Eq. 4. Upon simplification, the posterior sampling step of $\boldsymbol{\beta}^{mm'}$ can be written as

$$p(\boldsymbol{\beta}^{mm'}|.) \propto \exp\left[-\frac{1}{2\sigma^2}(\boldsymbol{\beta}^{mm'} - M)^\top \Sigma^{-1}(\boldsymbol{\beta}^{mm'} - M)\right],$$

$$\Sigma = \left(B + (\mathbf{X}^{*m'})^\top \Omega \mathbf{X}^{*m'}\right)^{-1}, \ M = \Sigma\left((\mathbf{X}^{*m'})^\top \Omega \mathbf{Y}\right).$$

Combining the steps together, the resulting Gibbs sampler proceeds as follows:

1. For $k = 1, \ldots, n$, draw w_k from $PG(X^m(s_k)+r_m, \eta_m(s_k))$, where $\eta_m(s_k) = \beta_0^{mm'}(s_k)+X^{m'}(s_k)\beta_1^{mm'}(s_k)$.

2. For $k = 1, \ldots, n$, define $y_k = \frac{X^m(s_k)-r_m}{2w_k}$.

3. Simulate $\boldsymbol{\beta}^{mm'}$ from $MVN(M, \Sigma)$, where

$$\Sigma = \left(B + (\mathbf{X}^{*m'})^\top \Omega \mathbf{X}^{*m'}\right)^{-1}, \ M = \Sigma\left((\mathbf{X}^{*m'})^\top \Omega \mathbf{Y}\right).$$

4. In case of the spatial fused lasso, consider steps 2 and 4 from Section 2.2.1, without the σ^2 term. In case of the spatial fused horseshoe, consider step 2 from Section 2.2.2, without the σ^2 term.

5. Update r_m using a conjugate Gamma distribution and introducing latent terms l_k's that follow the Chinese restaurant table (CRT) distribution, as described in Dadaneh et al. (2018) [14]

$$l_k \sim CRT(X^m(s_k), r_m),$$

$$r_m|L^1, \ldots, l_n, . \sim Gamma(a + \sum_{k=1}^n l_k, b - \sum_{k=1}^n \psi_m(s_k)).$$

2.3 Connection of the Gaussian model with bivariate spatial processes

In a univariate Gaussian process (GP)-based spatial model with an intercept and no covariates, it is typically assumed that $\mathbf{X}^m \sim MVN(\mu_m \mathbf{1}, \sigma_m^2 H + \sigma_E^2 I_n)$, where H is a spatial covariance matrix with $H_{kk'} = \rho(||s_k - s_{k'}||, \phi)$, $\rho(.)$ is a known stationary correlation function with hyperparameter ϕ. I_n is the $n \times n$ identity matrix. σ_m^2 and σ_E^2 are spatial and aspatial variances, respectively. In a bivariate (or multivariate) context, to model the dependency between the two variables $\mathbf{X}^m$ and $\mathbf{X}^{m'}$, usually a bivariate process with a separable Kronecker product-based covariance matrix is considered (see chapter 9 of Banerjee et al. (2008) [7],

$$\begin{bmatrix} \mathbf{X}^m \\ \mathbf{X}^{m'} \end{bmatrix} \sim MVN \left(\begin{bmatrix} \mu_m \mathbf{1} \\ \mu_{m'} \mathbf{1} \end{bmatrix}, \mathbf{\Sigma} = \underbrace{\begin{bmatrix} \sigma_m^2 & \nu \sigma_m \sigma_{m'} \\ \nu \sigma_m \sigma_{m'} & \sigma_{m'}^2 \end{bmatrix}}_{T} \otimes H \right). \tag{6}$$

The autocorrelation of both variables has the form $corr(X^m(s_k), X^m(s_{k'})) = \rho(||s_k - s_{k'}||, \phi)$. While the parameter ν appears as an "at-location" correlation term since $corr(X^m(s_k), X^{m'}(s_k)) = \nu$, it also regulates the cross-correlation as $corr(X^m(s_k), X^{m'}(s_{k'})) = \nu \rho(||s_k - s_{k'}||, \phi)$ (between locations). Notice that the aspatial variance terms (σ_E^2) have been dropped to utilize the computational benefits associated with a Kronecker product structure, such as the ease of computing the inverse: $\mathbf{\Sigma}^{-1} = T^{-1} \otimes H^{-1}$. With appropriate priors on every parameter, a Bayesian model-fitting approach is straightforward.

To demonstrate how the Gaussian spatially varying coefficients (SVC) model (Eq. 1 from the main text) may alternatively be used to estimate ν and ϕ, we focus on the conditional distribution [15] of $\mathbf{X}^m$,

$$\mathbf{X}^m | \mathbf{X}^{m'} \sim MVN(\tilde{\boldsymbol{\mu}}_m, \tilde{\mathbf{\Sigma}}_m),\ \tilde{\boldsymbol{\mu}}_m = \mu_m \mathbf{1} + \mathbf{\Sigma}_{12} \mathbf{\Sigma}_{22}^{-1} (\mathbf{X}^{m'} - \mu_{m'} \mathbf{1}),\ \tilde{\mathbf{\Sigma}}_m = \mathbf{\Sigma}_{11} - \mathbf{\Sigma}_{12} \mathbf{\Sigma}_{22}^{-1} \mathbf{\Sigma}_{21}, \tag{7}$$

where $\mathbf{\Sigma}_{11} = \sigma_m^2 H$, $\mathbf{\Sigma}_{12} = \nu \sigma_m \sigma_{m'} H$, and $\mathbf{\Sigma}_{22} = \sigma_{m'}^2 H$ are the respective blocks of the covariance matrix $\mathbf{\Sigma}$. The conditional mean and covariance matrix can be simplified as

$$\tilde{\boldsymbol{\mu}}_m = \mu_m \mathbf{1} + \nu \frac{\sigma_m}{\sigma_{m'}} (\mathbf{X}^{m'} - \mu_{m'} \mathbf{1}),\ \tilde{\mathbf{\Sigma}}_m = (1 - \nu^2) \sigma_m^2 H.$$

Therefore, given $\mathbf{X}^{m'}$ and consistent estimators of $\mu_{m'}$, $\sigma^2_{m'}$ (such as sample moments obtained from marginal distribution) or simply upon standardizing $\mathbf{X}^{m'}$ by mean and SD, a linear regression model [15] becomes appealing to estimate ν and other parameters as

$$\mathbf{X}^m = \mu_m \mathbf{1} + \nu \frac{\sigma_m}{\sigma_{m'}} (\mathbf{X}^{m'} - \mu_{m'} \mathbf{1}) + \mathbf{w}, \;\; \mathbf{w} \sim MVN\left(\mathbf{0}, \sigma^{*2}_m H\right), \;\; \sigma^{*2}_m = (1-\nu^2)\sigma^2_m. \tag{8}$$

where $\mathbf{w} = (w_1, \dots, w_n)^\top$ is a latent spatially varying random effect. In a Bayesian estimation, $\mathbf{w}$ is generally sampled at each MCMC step (could be marginalized otherwise). Thus, the correspondence with the SVC model (Eq. 1 from the main text) is found by letting $\beta_0(s_k) = \mu_m + w_k$ and $\beta_1(s_k) = \nu\sigma_m/\sigma_{m'} = \beta_1, \forall k \in \{1, \dots, n\}$. With such a special case of the SVC model, one can assume a GP prior on $\boldsymbol{\beta}_0 = \mu_m \mathbf{1} + \mathbf{w}$ as $\boldsymbol{\beta}_0 \sim MVN(\mathbf{0}, \sigma^{*2}_m H)$, and pursue a Bayesian approach to estimate $\boldsymbol{\beta}_0, \beta_1, \sigma^*_m$, and ϕ, from which the original parameters ν and σ_m are easily recovered. μ_m is identifiable up to an additive constant. The extra noise term $\epsilon(s_k)$ in the SVC model (Eq. 1 from the main text) that we do not find in Eq. 8 might help in capturing any potential aspatial source of variation that is missed by the Kronecker structure (Eq. 6). Additional covariates such as $Z(s_k)$ may be added to the mean in Eq. 6 and the derivation remains similar.

A natural question arises: where does our model make simplifications compared to directly fitting the joint bivariate process from Eq. 6? Recall that the spatial covariance matrix H is not fixed; instead, it is a function of the hyperparameter ϕ. We have $\pi(\mathbf{X}^m, \mathbf{X}^{m'}) = \pi(\mathbf{X}^m | \mathbf{X}^{m'})\pi(\mathbf{X}^{m'})$. While the effect of disregarding $\pi(\mathbf{X}^{m'})$ might be minimal on the inference of ν, we lose information on ϕ, estimating it solely based on the spatial autocorrelation of $\mathbf{X}^m$. Moreover, it is unclear which of the two variables, $\mathbf{X}^{m'}$ or $\mathbf{X}^m$, should be conditioned on. If there is prior knowledge on the biological causal mechanism between the variables, one variable might be preferred over the other as the so-called outcome. For example, in the LR analysis, it might be more appropriate to treat receptor expression as a function of ligand expression. As a side note, the simple Pearson correlation is still an asymptotically unbiased estimator of ν but with a large variance due to the spatial autocorrelation present in both variables [16, 17, 18], which explains its inflated type 1 error in simulations. The spatially weighted cross-correlation measures, such as bivariate Moran's I or Lee's L statistic, are essentially Pearson correlation between spatially lagged variables, and therefore, produce inflated type 1 errors as well (see Section 2.4).

By treating $\beta_1(s)$ as a spatial process, our SVC framework leads to a more general bivariate spatial process of $(\mathbf{X}^m, \mathbf{X}^{m'})^\top$, than Eq. 6. Conditional on the process $\beta_1(s)$, we can derive the covariance of the bivariate process as follows. In the Gaussian SVC model, let $\mathbf{X}^{m'} \sim MVN(\mu_{m'}\mathbf{1}, \sigma^2_{m'}H)$, $\boldsymbol{\beta}_0 \sim MVN(\mathbf{0}, \sigma^2_{m0}H)$ (independent of $\mathbf{X}^{m'}$), $\epsilon(s_k) = 0$, and $\boldsymbol{\beta}_1$ be a fixed vector. We have $cov(X^m(s_k), X^{m'}(s_k)) = cov(\beta_0(s_k) + \beta_1(s_k)X^{m'}(s_k), X^{m'}(s_k)) = \beta_1(s_k)\sigma^2_{m'}$ and at-location correlation becomes, $corr(X^m(s_k), X^{m'}(s_k)) = \frac{\beta_1(s_k)\sigma^2_{m'}}{\sqrt{(\sigma^2_{m0}+\beta_1^2(s_k)\sigma^2_{m'})\sigma^2_{m'}}} = \nu(s_k)$ (let) which is not a constant anymore. Autocorrelation takes the form, $corr(X^m(s_k), X^m(s_{k'})) = \frac{\rho(||s_k - s_{k'}||, \phi)(\beta_1(s_k)\beta_1(s_{k'})\sigma^2_{m'} + \sigma^2_{m0})}{\sqrt{(\sigma^2_{m0}+\beta_1^2(s_k)\sigma^2_{m'})(\sigma^2_{m0}+\beta_1^2(s_{k'})\sigma^2_{m'})}} = \rho(||s_k - s_{k'}||, \phi)(\nu(s_k)\nu(s_{k'}) + l(s_k)l(s_{k'}))$, $l(s_k) = \frac{\sigma^2_{m0}}{\sigma^2_{m0}+\beta_1^2(s_k)\sigma^2_{m'}}$, and cross-correlation becomes $corr(X^m(s_k), X^{m'}(s_{k'})) = \rho(||s_k - s_{k'}||, \phi)\nu(s_k)$ which is asymmetric in $(s_k, s_{k'})$. Using a multivariate notation,

$$\begin{bmatrix} \mathbf{X}^m \\ \mathbf{X}^{m'} \end{bmatrix} \sim MVN\left(\begin{bmatrix} \mu_{m'}\boldsymbol{\beta}_1 \\ \mu_{m'}\mathbf{1} \end{bmatrix}, \begin{bmatrix} \sigma^2_{m0}H + \sigma^2_{m'}D_{\boldsymbol{\beta}_1}HD^\top_{\boldsymbol{\beta}_1} & \sigma^2_{m'}D_{\boldsymbol{\beta}_1}H \\ \sigma^2_{m'}HD_{\boldsymbol{\beta}_1} & \sigma^2_{m'}H \end{bmatrix} \right), D_{\boldsymbol{\beta}_1} = diag(\boldsymbol{\beta}_1). \quad (9)$$

When $\boldsymbol{\beta}_1 = \nu\frac{\sigma_m}{\sigma_{m'}}\mathbf{1}$ or $D_{\boldsymbol{\beta}_1} = \nu\frac{\sigma_m}{\sigma_{m'}}I_n$, and $\sigma^2_{m0} = (1-\nu^2)\sigma^2_m$, we have $cov(\mathbf{X}^m) = \sigma^2_m H$, $cov(\mathbf{X}^m, \mathbf{X}^{m'}) = \nu\sigma_m\sigma_{m'}H$, same as Eq. 6.

2.4 Asymptotic mean and variance of bivariate Moran's I

In this section, we derive the asymptotic mean and variance of the bivariate Moran's I statistic and provide sufficient regularity conditions for these results. We then discuss how these conditions may fail in practice, potentially making bivariate Moran's I an unreliable measure of cross-correlation. Throughout, we assume

$$\begin{bmatrix} \mathbf{X}^m \\ \mathbf{X}^{m'} \end{bmatrix} \sim \text{MVN}\left(\begin{bmatrix} \mathbf{0} \\ \mathbf{0} \end{bmatrix}, \begin{bmatrix} \boldsymbol{\Sigma}_m & \boldsymbol{\Sigma}_{mm'} \\ \boldsymbol{\Sigma}^\top_{mm'} & \boldsymbol{\Sigma}_{m'} \end{bmatrix} \right).$$

Using matrix notation, the bivariate Moran's I for a pair of genes (m, m') can be written as [19]

$$I_{BV} = \frac{n}{s_0} \frac{(\mathbf{X}^m)^\top W \mathbf{X}^{m'}}{\sqrt{(\mathbf{X}^m)^\top \mathbf{X}^m} \sqrt{(\mathbf{X}^{m'})^\top \mathbf{X}^{m'}}} = \frac{n}{s_0} \frac{D_{mm'}}{(D_m D_{m'})^{1/2}}, \quad s_0 = \sum_{i,j} w_{ij},$$

where W is a matrix of spatial weights (symmetric or row-standardized), and $D_m = (\mathbf{X}^m)^\top \mathbf{X}^m$, $D_{m'} = (\mathbf{X}^{m'})^\top \mathbf{X}^{m'}$, $D_{mm'} = (\mathbf{X}^{m'})^\top W \mathbf{X}^m$. $s_0 = n$ if W is row-standardized. We assume $\mathbf{\Sigma}_m, \mathbf{\Sigma}_{m'} \succ 0$, i.e., positive definite. By standard properties of the multivariate normal and quadratic forms [6],

$$\begin{aligned}
&\mathbb{E}(D_{mm'}) = \mathbb{E}[(\mathbf{X}^m)^\top W \mathbf{X}^{m'}] = \operatorname{tr}(W\mathbb{E}[\mathbf{X}^{m'}(\mathbf{X}^m)^\top]) = \operatorname{tr}(W\mathbf{\Sigma}_{mm'});\\
&\mathbb{E}(D_m) = \mathbb{E}[(\mathbf{X}^m)^\top \mathbf{X}^m] = \operatorname{tr}(\mathbf{\Sigma}_m),\ \ \mathbb{E}(D_{m'}) = \mathbb{E}[(\mathbf{X}^{m'})^\top \mathbf{X}^{m'}] = \operatorname{tr}(\mathbf{\Sigma}_{m'});\\
&\operatorname{Var}(D_m) = 2\operatorname{tr}(\mathbf{\Sigma}_m^2),\ \ \operatorname{Var}(D_{m'}) = 2\operatorname{tr}(\mathbf{\Sigma}_{m'}^2);\\
&D_m = \sum_{i=1}^n \lambda_{m,i} Y_{m,i}^2,\ \ D_{m'} = \sum_{i=1}^n \lambda_{m',i} Y_{m',i}^2;\\
&\operatorname{Var}(D_{mm'}) = \mathbb{E}(D_{mm'}^2) - \mathbb{E}(D_{mm'})^2 \qquad (10)\\
&= \mathbb{E}[(\mathbf{X}^m)^\top W \mathbf{X}^{m'}(\mathbf{X}^{m'})^\top W^\top \mathbf{X}^m] - \operatorname{tr}(W\mathbf{\Sigma}_{mm'})^2\\
&= \operatorname{tr}(W\mathbb{E}[\mathbf{X}^{m'}(\mathbf{X}^{m'})^\top W^\top \mathbf{X}^m(\mathbf{X}^m)^\top]) - \operatorname{tr}(W\mathbf{\Sigma}_{mm'})^2\\
&= \operatorname{tr}(W\mathbb{E}_{m'}[\mathbb{E}_{m|m'}[\mathbf{X}^{m'}(\mathbf{X}^{m'})^\top W^\top \mathbf{X}^m(\mathbf{X}^m)^\top]|\mathbf{X}^{m'}]) - \operatorname{tr}(W\mathbf{\Sigma}_{mm'})^2\\
&= \operatorname{tr}(W\mathbb{E}_{m'}[\mathbf{X}^{m'}(\mathbf{X}^{m'})^\top]W^\top \mathbf{\Sigma}_m) - \operatorname{tr}(W\mathbf{\Sigma}_{mm'})^2\\
&= \operatorname{tr}(W\mathbf{\Sigma}_{m'}W^\top \mathbf{\Sigma}_m) - \operatorname{tr}(W\mathbf{\Sigma}_{mm'})^2,
\end{aligned}$$

where $\{\lambda_{m,i}\}_{i=1}^n$ and $\{\lambda_{m',i}\}_{i=1}^n$ are the eigenvalues of $\mathbf{\Sigma}_m$ and $\mathbf{\Sigma}_{m'}$, respectively. $Y_{m,i}$'s and $Y_{m',i}$'s are i.i.d. $N(0,1)$, leading to weighted χ^2 distributions for D_m and $D_{m'}$. Let $||.||_F$ and $||.||_{\text{op}}$ denote the Frobenius norm and operator norm, respectively. Next, we investigate the convergence properties of D_m and $D_{m'}$ under some realistic assumptions, to ultimately derive the asymptotic mean and variance of I_{BV} and their bounds (presented in Theorem 2).

Theorem 1. *For each $u \in \{m, m'\}$, let $\mathbf{Z}^u = (\mathbf{\Sigma}_u)^{-1/2}\mathbf{X}^u$ and $D_u = (\mathbf{X}^u)^\top \mathbf{X}^u = (\mathbf{Z}^u)^\top \mathbf{\Sigma}_u \mathbf{Z}^u$. Assume that $\operatorname{tr}(\mathbf{\Sigma}_u) = \Theta(n)$ and there exist constants $0 < c_u < C_u < \infty$ and n_0 such that for all $n \geq n_0$, $c_u \leq \lambda_{\min}(\mathbf{\Sigma}_u) \leq \|\mathbf{\Sigma}_u\|_{\text{op}} \leq C_u$ (i.e., eigenvalues are uniformly bounded away from 0 and ∞). Then,*

$$\frac{D_u}{\operatorname{tr}(\mathbf{\Sigma}_u)} \xrightarrow{L^2} 1 \ \ \textit{for}\ \ u \in \{m, m'\}$$

and further if $\mathbf{\Sigma}_{mm'} = \mathbf{0}$, i.e., $\mathbf{X}^u$'s and equivalently, $\mathbf{Z}^u$'s are independent,

$$\frac{(D_m D_{m'})^{-1/2}}{\big(\operatorname{tr}(\mathbf{\Sigma}_m)\operatorname{tr}(\mathbf{\Sigma}_{m'})\big)^{-1/2}} \xrightarrow{L^2} 1.$$

Proof. First, we find the bounds of $\operatorname{tr}(\mathbf{\Sigma}_u^2)$. Using Cauchy–Schwarz inequality and basic algebra,

$$(\operatorname{tr}(\mathbf{\Sigma}_u))^2 = \Big(\sum_{i=1}^n \lambda_{u,i}\Big)^2 \le n\sum_{i=1}^n \lambda_{u,i}^2 = n\ \operatorname{tr}(\mathbf{\Sigma}_u^2) \ \Rightarrow\ \operatorname{tr}(\mathbf{\Sigma}_u^2) \ \ge\ \frac{(\operatorname{tr}(\mathbf{\Sigma}_u))^2}{n},$$

$$\operatorname{tr}(\mathbf{\Sigma}_u^2) = \sum_{i=1}^n \lambda_{u,i}^2 \le \Big(\max_{1\le i\le n} \lambda_{u,i}\Big)\sum_{i=1}^n \lambda_{u,i} = \|\mathbf{\Sigma}_u\|_{\text{op}}\ \operatorname{tr}(\mathbf{\Sigma}_u).$$

Since $\|\mathbf{\Sigma}_u\|_{\text{op}}= O(1)$ and $\operatorname{tr}(\mathbf{\Sigma}_u) = \Theta(n)$,

$$\frac{(\operatorname{tr}(\mathbf{\Sigma}_u))^2}{n} \ \le\ \operatorname{tr}(\mathbf{\Sigma}_u^2)\ \le\ \|\mathbf{\Sigma}_u\|_{\text{op}}\operatorname{tr}(\mathbf{\Sigma}_u) \quad\Rightarrow\quad \operatorname{tr}(\mathbf{\Sigma}_u^2) = \Theta(n), \qquad u\in\{m,m'\}.$$

Note that

$$\mathbb{E}\left[\left(\frac{D_u}{\operatorname{tr}(\mathbf{\Sigma}_u)}-1\right)^2\right] = \frac{\operatorname{Var}(D_u)}{\operatorname{tr}(\mathbf{\Sigma}_u)^2} = \frac{2\ \operatorname{tr}(\mathbf{\Sigma}_u^2)}{\operatorname{tr}(\mathbf{\Sigma}_u)^2} = O\Big(\frac{n}{n^2}\Big) \to 0,$$

Thus, we have

$$\boxed{\frac{D_u}{\operatorname{tr}(\mathbf{\Sigma}_u)} \xrightarrow{L^2} 1 \qquad \text{for } u\in\{m,m'\}.}$$

Next, we show that the inverse of this term converges to 1 in the L^2 norm as well. Let $U = \frac{D_m}{\operatorname{tr}(\mathbf{\Sigma}_m)}$ and define $A := \{|U-1|\le 1/2\}$. On A we have $U \ge 1/2$, hence $U^{-2}\le 4$, and therefore

$$\mathbb{E}\left[\frac{(U-1)^2}{U^2}\mathbf{1}_A\right] \le 4\,\mathbb{E}[(U-1)^2] \ \longrightarrow\ 0 \qquad \text{since}\ \ U \xrightarrow{L^2} 1.$$

On A^c, we have $(U-1)^2 \le 2(U^2+1)$ which leads to

$$\frac{(U-1)^2}{U^2} \le 2(1+U^{-2}),$$

and further gives us

$$\mathbb{E}\left[\frac{(U-1)^2}{U^2}\mathbf{1}_{A^c}\right] \leq 2\,\Pr(A^c) + 2\,\mathbb{E}[U^{-2}\mathbf{1}_{A^c}].$$

Convergence in L^2 implies convergence in probability, and thus $U \xrightarrow{L^2} 1$ implies $U \xrightarrow{p} 1$, i.e., $\Pr(A^c) \to 0$. Also, by Cauchy-Schwarz inequality,

$$\mathbb{E}[U^{-2}\mathbf{1}_{A^c}] \leq (\mathbb{E}[U^{-4}])^{1/2}\,\Pr(A^c)^{1/2} \;\longrightarrow\; 0,$$

since $\sup_n \mathbb{E}(U^{-4}) < \infty$ (under Lemma 1 proved below) and $\Pr(A^c) \to 0$. Combining the two parts yields

$$\mathbb{E}[(U^{-1}-1)^2] = \mathbb{E}\left[\frac{(U-1)^2}{U^2}\right] = \mathbb{E}\left[\frac{(U-1)^2}{U^2}\mathbf{1}_A\right] + \mathbb{E}\left[\frac{(U-1)^2}{U^2}\mathbf{1}_{A^c}\right] \;\longrightarrow\; 0,$$

i.e., $U^{-1} \xrightarrow{L^2} 1$. The same argument applies to $V = \frac{D_{m'}}{\mathrm{tr}(\mathbf{\Sigma}_{m'})}$, resulting $V^{-1} \xrightarrow{L^2} 1$. Next we show that the product of U^{-1} and V^{-1}, $W_n := U^{-1}V^{-1}$, converges to 1 in L^2 norm as well.

Let $X_n := U^{-1}$ and $Y_n := V^{-1}$. Under $\mathbf{\Sigma}_{mm'} = \mathbf{0}$, U and V are independent, hence X_n and Y_n are independent. Then $X_nY_n - 1 = (X_n-1)Y_n + (Y_n-1)$, and,

$$\mathbb{E}[(X_nY_n-1)^2] \leq 2\,\mathbb{E}[(X_n-1)^2Y_n^2] + 2\,\mathbb{E}[(Y_n-1)^2].$$

By independence,

$$\mathbb{E}[(X_n-1)^2Y_n^2] = \mathbb{E}[(X_n-1)^2]\;\mathbb{E}[Y_n^2].$$

Since $X_n \to 1$ in L^2, we have $\mathbb{E}[(X_n-1)^2] \to 0$. Moreover, $\sup_n \mathbb{E}[Y_n^2] = \sup_n \mathbb{E}[V^{-2}] < \infty$ (e.g., implied by $\sup_n \mathbb{E}[V^{-4}] < \infty$ from Lemma 1). Also $Y_n \to 1$ in L^2 implies $\mathbb{E}[(Y_n-1)^2] \to 0$. Therefore,

$$\mathbb{E}[(U^{-1}V^{-1}-1)^2] \to 0, \qquad \text{i.e.,} \qquad W_n = U^{-1}V^{-1} = \frac{(D_mD_{m'})^{-1}}{(\,\mathrm{tr}(\mathbf{\Sigma}_m)\,\mathrm{tr}(\mathbf{\Sigma}_{m'}))^{-1}} \xrightarrow{L^2} 1.$$

Finally, we show $W_n^{1/2} = U^{-1/2}V^{-1/2} \xrightarrow{L^2} 1$. Since $W_n \xrightarrow{L^2} 1$, we also have $W_n \xrightarrow{p} 1$ and in particular $\Pr(W_n < 1/2) \to 0$. Moreover, by Cauchy–Schwarz inequality,

$$\mathbb{E}[(W_n-1)^2\mathbf{1}\{W_n<1/2\}] \le (\mathbb{E}[(W_n-1)^4])^{1/2}\,\Pr(W_n<1/2)^{1/2} \to 0,$$

because $\sup_n \mathbb{E}[W_n^4] = \sup_n \mathbb{E}[U^{-4}V^{-4}] = \sup_n \mathbb{E}[U^{-4}]\,\sup_n \mathbb{E}[V^{-4}] < \infty$ under independence and Lemma 1. On $\{W_n \ge 1/2\}$ we have ${W_n}^{1/2}+1 \ge 1$, hence

$$({W_n}^{1/2}-1)^2 = \frac{(W_n-1)^2}{({W_n}^{1/2}+1)^2} \le (W_n-1)^2.$$

Therefore,

$$\mathbb{E}[({W_n}^{1/2}-1)^2] \le \mathbb{E}[(W_n-1)^2\mathbf{1}\{W_n \ge 1/2\}] + \mathbb{E}[({W_n}^{1/2}-1)^2\mathbf{1}\{W_n<1/2\}] \le \mathbb{E}[(W_n-1)^2] + o(1) \to 0,$$

which proves ${W_n}^{1/2} = U^{-1/2}V^{-1/2} = \boxed{\dfrac{(D_m D_{m'})^{-1/2}}{(\,\mathrm{tr}(\mathbf{\Sigma}_m)\,\mathrm{tr}(\mathbf{\Sigma}_{m'}))^{-1/2}} \xrightarrow{L^2} 1.}$

□

Lemma 1. Assume $\mathbf{Z} \sim MVN(\mathbf{0}, I_n)$ and $\mathbf{\Sigma} \succ 0$ with similar constraints as before, $0 < c \le \lambda_{\min}(\mathbf{\Sigma}) \le \|\mathbf{\Sigma}\|_{\mathrm{op}} \le C < \infty$. Let $D = \mathbf{Z}^\top \mathbf{\Sigma} \mathbf{Z}$ and $U = D/\mathrm{tr}(\mathbf{\Sigma})$. Then for $n > 8$,

$$\mathbb{E}(U^{-4}) \le \left(\frac{C}{c}\right)^4 \frac{n^4}{(n-2)(n-4)(n-6)(n-8)},$$

and $\sup_{n\ge 16} \mathbb{E}(U^{-4}) < \infty$. Moreover, for any fixed integer $k \ge 4$, $\sup_{n \ge 4k} \mathbb{E}(U^{-k}) < \infty$.

Proof. By the eigenvalue bounds, $D \ge c\|\mathbf{Z}\|^2$ and $\mathrm{tr}(\mathbf{\Sigma}) \le Cn$, and $||\mathbf{Z}||^2 = \mathbf{Z}^\top\mathbf{Z} \sim \chi^2_n$, hence

$$U^{-4} = \left(\frac{\mathrm{tr}(\mathbf{\Sigma})}{D}\right)^4 \le \left(\frac{Cn}{c\,\|\mathbf{Z}\|^2}\right)^4 = \left(\frac{C}{c}\right)^4 \left(\frac{n}{\chi^2_n}\right)^4,$$

For any random variable $L \sim \chi^2_n$ and $n > 8$, the negative-moment identity gives

$$\mathbb{E}(L^{-4}) = 2^{-4}\frac{\Gamma(\frac{n}{2}-4)}{\Gamma(\frac{n}{2})} = \frac{1}{(n-2)(n-4)(n-6)(n-8)},$$

thus we have

$$\mathbb{E}\left[U^{-4}\right] \le \mathbb{E}\left[\left(\frac{C}{c}\right)^4 \left(\frac{n}{\chi_n^2}\right)^4\right] = \left(\frac{C}{c}\right)^4 \frac{n^4}{(n-2)(n-4)(n-6)(n-8)}.$$

Moreover, for $n \ge 16$, $(n-2)(n-4)(n-6)(n-8) \ge (n/2)^4$, hence the ratio $\frac{n^4}{(n-2)(n-4)(n-6)(n-8)} \le 16$ and finally, $\sup_{n\ge 16} \mathbb{E}(U^{-4}) \le 16(C/c)^4 < \infty$. More generally, for any fixed integer $k \ge 4$ and $n > 2k$, a similar argument yields

$$\mathbb{E}(U^{-k}) \le \left(\frac{C}{c}\right)^k \frac{n^k}{(n-2)(n-4)\cdots(n-2k)}, \qquad \sup_{n\ge 4k} \mathbb{E}(U^{-k}) < \infty.$$

□

Theorem 2. *If a) the eigenvalue conditions of Theorem 1 holds, b) $\mathbf{\Sigma}_{mm'} = \mathbf{0}$, and c) $\|W\|_F^2/s_0^2 = O(1)$,*

$$\mathbb{E}(I_{BV}) = o(1), \;\; \mathrm{Var}(I_{BV}) = \left(\frac{n}{s_0}\right)^2 \frac{\mathrm{tr}(W\,\mathbf{\Sigma}_{m'}\,W^\top\,\mathbf{\Sigma}_m)}{\mathrm{tr}(\mathbf{\Sigma}_m)\;\mathrm{tr}(\mathbf{\Sigma}_{m'})}(1+o(1)).$$

Proof. Let us write

$$I_{BV} = \frac{n}{s_0}\,\frac{D_{mm'}}{(D_m D_{m'})^{1/2}} = \frac{n}{s_0}\,\frac{D_{mm'}}{(\mathrm{tr}(\mathbf{\Sigma}_m)\,\mathrm{tr}(\mathbf{\Sigma}_{m'}))^{1/2}} \cdot \frac{(\mathrm{tr}(\mathbf{\Sigma}_m)\,\mathrm{tr}(\mathbf{\Sigma}_{m'}))^{1/2}}{(D_m D_{m'})^{1/2}}.$$

Define

$$T_0 := \frac{n}{s_0}\,\frac{D_{mm'}}{(\mathrm{tr}(\mathbf{\Sigma}_m)\,\mathrm{tr}(\mathbf{\Sigma}_{m'}))^{1/2}}, \qquad R_n := \frac{(D_m D_{m'})^{-1/2}}{(\mathrm{tr}(\mathbf{\Sigma}_m)\,\mathrm{tr}(\mathbf{\Sigma}_{m'}))^{-1/2}}.$$

We can write $I_{BV} = T_0 R_n$. When $\mathbf{\Sigma}_{mm'} = \mathbf{0}$, from Eq. 10, $\mathbb{E}(D_{mm'}) = 0$ or $\mathbb{E}(T_0) = 0$. Moreover,

$$\mathbb{E}(I_{BV}) = \mathbb{E}(T_0 R_n) = \mathbb{E}(T_0) + \mathbb{E}\{T_0(R_n - 1)\} = \mathbb{E}\{T_0(R_n - 1)\}.$$

By Cauchy–Schwarz inequality,

$$|\mathbb{E}\{T_0(R_n - 1)\}| \le \{\mathbb{E}(T_0^2)\}^{1/2}\{\mathbb{E}[(R_n - 1)^2]\}^{1/2}.$$

From Eq. 10, under $\mathbf{\Sigma}_{mm'} = \mathbf{0}$,

$$\mathrm{Var}(T_0) = \mathbb{E}(T_0^2) = \left(\frac{n}{s_0}\right)^2 \frac{\mathrm{Var}(D_{mm'})}{\mathrm{tr}(\mathbf{\Sigma}_m)\,\mathrm{tr}(\mathbf{\Sigma}_{m'})} = \left(\frac{n}{s_0}\right)^2 \frac{\mathrm{tr}(W\mathbf{\Sigma}_{m'}W^\top\mathbf{\Sigma}_m)}{\mathrm{tr}(\mathbf{\Sigma}_m)\,\mathrm{tr}(\mathbf{\Sigma}_{m'})}.$$

Using $\|M\|_F^2 = \mathrm{tr}(MM^\top)$, we have

$$\mathrm{tr}(W\mathbf{\Sigma}_{m'}W^\top\mathbf{\Sigma}_m) = \mathrm{tr}(\mathbf{\Sigma}_m^{1/2}W\mathbf{\Sigma}_{m'}W^\top\mathbf{\Sigma}_m^{1/2}) = \mathrm{tr}\Big((\mathbf{\Sigma}_m^{1/2}W\mathbf{\Sigma}_{m'}^{1/2})(\mathbf{\Sigma}_m^{1/2}W\mathbf{\Sigma}_{m'}^{1/2})^\top\Big) = \|\mathbf{\Sigma}_m^{1/2}W\mathbf{\Sigma}_{m'}^{1/2}\|_F^2.$$

By Frobenius-operator norm inequality: $\|AB\|_F \le \|A\|_{\mathrm{op}}\|B\|_F$ (applied twice),

$$\mathrm{tr}(W\mathbf{\Sigma}_{m'}W^\top\mathbf{\Sigma}_m) = \|\mathbf{\Sigma}_{m'}^{1/2}W\mathbf{\Sigma}_m^{1/2}\|_F^2 \le \|\mathbf{\Sigma}_m\|_{\mathrm{op}}\|\mathbf{\Sigma}_{m'}\|_{\mathrm{op}}\|W\|_F^2.$$

Since we assume $\|\mathbf{\Sigma}_u\|_{\mathrm{op}} \le C_u$ and $\mathrm{tr}(\mathbf{\Sigma}_u) = \Theta(n)$ (i.e., $\mathrm{tr}(\mathbf{\Sigma}_u) \ge l_u n$ for some $l_u > 0$) for $u \in \{m, m'\}$, it follows that

$$\mathbb{E}(T_0^2) \;\le\; \frac{C_m C_{m'}}{l_m l_{m'}}\,\frac{\|W\|_F^2}{s_0^2}.$$

Under the assumption of $\|W\|_F^2/s_0^2 = O(1)$ (as holds for common row-standardized adjacency weights), $\mathbb{E}(T_0^2) = O(1)$. Coupled with Theorem 1 yielding $\mathbb{E}[(R_n-1)^2] \to 0$, we have $\boxed{\mathbb{E}(I_{BV}) = o(1).}$

Write $R_n = 1 + \epsilon_n$ with $\epsilon_n := R_n - 1$. Then

$$\mathrm{Var}(I_{BV}) = \mathrm{Var}\left(T_0(1+\epsilon_n)\right) = \mathrm{Var}(T_0) + \mathrm{Var}(T_0\epsilon_n) + 2\ \mathrm{Cov}(T_0, T_0\epsilon_n).$$

By Cauchy–Schwarz inequality,

$$\mathrm{Var}(T_0\epsilon_n) \le \mathbb{E}[T_0^2\epsilon_n^2] \le \{\mathbb{E}(T_0^4)\}^{1/2}\{\mathbb{E}(\epsilon_n^4)\}^{1/2}, \qquad |\mathrm{Cov}(T_0, T_0\epsilon_n)| \le \{\mathbb{E}(T_0^4)\}^{1/2}\{\mathbb{E}(\epsilon_n^2)\}^{1/2}.$$

By Theorem 1, $\mathbb{E}(\epsilon_n^2) \to 0$. Letting $U = \frac{D_m}{\mathrm{tr}(\mathbf{\Sigma}_m)}$ and $V = \frac{D_{m'}}{\mathrm{tr}(\mathbf{\Sigma}_{m'})}$ as before and by using Lemma 1,

$$\sup_{n\ge 16}\mathbb{E}(R_n^8) = \sup_{n\ge 16}\mathbb{E}[(UV)^{-4}] = \sup_{n\ge 16}\mathbb{E}(U^{-4})\,\mathbb{E}(V^{-4}) < \infty.$$

This uniform 8th-moment bound implies $\sup_n \mathbb{E}(\epsilon_n^8) < \infty$ (since $|\epsilon_n|^8 = |R_n - 1|^8 \le 2^7(1 + R_n^8)$ and $\sup_n \mathbb{E}(R_n^8) < \infty$). Therefore, by the Riesz–Thorin interpolation theorem between L^2 and L^8 [20],

$$\|\epsilon_n\|_4 \le \|\epsilon_n\|_2^{1/3} \|\epsilon_n\|_8^{2/3} \quad \Longrightarrow \quad \mathbb{E}(\epsilon_n^4) \le (\mathbb{E}(\epsilon_n^2))^{2/3} \, (\mathbb{E}(\epsilon_n^8))^{1/3} \to 0,$$

since $\mathbb{E}(\epsilon_n^2) \to 0$ and $\sup_n \mathbb{E}(\epsilon_n^8) < \infty$. Using Lemma 2 (proved below), $\mathbb{E}(T_0^4) = O(\mathrm{Var}(T_0)^2)$ and thus,

$$\mathrm{Var}(T_0\epsilon_n) = o(\mathrm{Var}(T_0)), \qquad \mathrm{Cov}(T_0, T_0\epsilon_n) = o(\mathrm{Var}(T_0)),$$

and consequently

$$\boxed{\mathrm{Var}(I_{BV}) = \mathrm{Var}(T_0)\{1 + o(1)\} = \left(\frac{n}{s_0}\right)^2 \frac{\mathrm{tr}(W\,\mathbf{\Sigma}_{m'}\,W^\top\,\mathbf{\Sigma}_m)}{\mathrm{tr}(\mathbf{\Sigma}_m)\;\mathrm{tr}(\mathbf{\Sigma}_{m'})}(1 + o(1)).}$$

□

Lemma 2. Under $\mathbf{\Sigma}_{mm'} = \mathbf{0}$,

$$\mathbb{E}(D_{mm'}^4) = 3\Big\{2\ \mathrm{tr}[(W\mathbf{\Sigma}_{m'}W^\top\mathbf{\Sigma}_m)^2] + \mathrm{tr}(W\mathbf{\Sigma}_{m'}W^\top\mathbf{\Sigma}_m)^2\Big\} \ \le\ 9\ \mathrm{tr}(W\mathbf{\Sigma}_{m'}W^\top\mathbf{\Sigma}_m)^2 = 9\mathbb{E}(D_{mm'}^2)^2.$$

Consequently, for

$$T_0 := \frac{n}{s_0}\,\frac{D_{mm'}}{\{\mathrm{tr}(\mathbf{\Sigma}_m)\,\mathrm{tr}(\mathbf{\Sigma}_{m'})\}^{1/2}},$$

we have

$$\mathbb{E}(T_0^4) \le 9\,\mathbb{E}(T_0^2)^2, \qquad \text{hence} \qquad \mathbb{E}(T_0^4) = O(\mathbb{E}(T_0^2)^2) = O(\,\mathrm{Var}(T_0)^2).$$

Proof. Conditioning on $\mathbf{X}^m$, the random variable $D_{mm'} = (\mathbf{X}^{m'})^\top(W^\top\mathbf{X}^m)$ is Gaussian with mean 0 and conditional variance $(\mathbf{X}^m)^\top(W\mathbf{\Sigma}_{m'}W^\top)\mathbf{X}^m$. Therefore, $\mathbb{E}(D_{mm'}^2 \mid \mathbf{X}^m) = (\mathbf{X}^m)^\top(W\mathbf{\Sigma}_{m'}W^\top)\mathbf{X}^m$ and using properties of Gaussian moments,

$$\mathbb{E}(D_{mm'}^4 \mid \mathbf{X}^m) = 3\mathbb{E}(D_{mm'}^2 \mid \mathbf{X}^m)^2 = 3\Big((\mathbf{X}^m)^\top(W\mathbf{\Sigma}_{m'}W^\top)\mathbf{X}^m\Big)^2.$$

Taking expectation and using the standard Gaussian quadratic-form identity: for $\mathbf{X} \sim MVN(0, \mathbf{\Sigma})$ and symmetric A, $\mathbb{E}[(\mathbf{X}^\top A\mathbf{X})^2] = 2\ \mathrm{tr}(A\mathbf{\Sigma}A\mathbf{\Sigma}) + \mathrm{tr}(A\mathbf{\Sigma})^2$, with $A = W\mathbf{\Sigma}_{m'}W^\top$, yields

$$\mathbb{E}(D_{mm'}^4) = 3\Big\{2\ \mathrm{tr}[(W\boldsymbol{\Sigma}_{m'}W^\top\boldsymbol{\Sigma}_m)^2] + \mathrm{tr}(W\boldsymbol{\Sigma}_{m'}W^\top\boldsymbol{\Sigma}_m)^2\Big\}.$$

Finally, since $W\boldsymbol{\Sigma}_{m'}W^\top\boldsymbol{\Sigma}_m$ is *similar* to the positive definite matrix $\boldsymbol{\Sigma}_m^{1/2}W\boldsymbol{\Sigma}_{m'}W^\top\boldsymbol{\Sigma}_m^{1/2}$. Hence its eigenvalues are nonnegative [21], implying $\mathrm{tr}(M^2) \leq \mathrm{tr}(M)^2$, giving the bound $\mathbb{E}(D_{mm'}^4) \leq 9\ \mathrm{tr}(W\boldsymbol{\Sigma}_{m'}W^\top\boldsymbol{\Sigma}_m)^2$. The statement for T_0 follows simply by deterministic rescaling. □

Corollary 2.1. *Assume the conditions of Theorem 2 (in particular, $\boldsymbol{\Sigma}_{mm'} = \mathbf{0}$), so that*

$$\mathrm{Var}(I_{BV}) = \left(\frac{n}{s_0}\right)^2 \frac{\mathrm{tr}(W\,\boldsymbol{\Sigma}_{m'}\,W^\top\,\boldsymbol{\Sigma}_m)}{\mathrm{tr}(\boldsymbol{\Sigma}_m)\ \mathrm{tr}(\boldsymbol{\Sigma}_{m'})}\{1 + o(1)\}.$$

Fix $a := \mathrm{tr}(\boldsymbol{\Sigma}_m)$ and $b := \mathrm{tr}(\boldsymbol{\Sigma}_{m'})$. Then, for all $\boldsymbol{\Sigma}_m, \boldsymbol{\Sigma}_{m'} \succeq 0$ with these traces,

$$\mathrm{tr}(W\,\boldsymbol{\Sigma}_{m'}\,W^\top\,\boldsymbol{\Sigma}_m)\ \leq\ ab\,\|W\|_{\mathrm{op}}^2 =\ ab\,\sigma_{\max}(W)^2,$$

where $\sigma_{\max}(W)$ is the maximum singular value of W and consequently

$$\mathrm{Var}(I_{BV})\ \leq\ \left(\frac{n}{s_0}\right)^2 \sigma_{\max}(W)^2\{1 + o(1)\}.$$

Moreover, this bound is sharp: equality holds (up to the $\{1 + o(1)\}$ factor) when $\boldsymbol{\Sigma}_m = a\,\mathbf{u}_1\mathbf{u}_1^\top$ and $\boldsymbol{\Sigma}_{m'} = b\,\mathbf{v}_1\mathbf{v}_1^\top$, where $\mathbf{u}_1, \mathbf{v}_1$ are the top left/right singular vectors of W (so that $W\mathbf{v}_1 = \sigma_{\max}(W)\mathbf{u}_1$). If W is symmetric, $\sigma_{\max}(W) = \lambda_{\max}(W)$ (maximum eigenvalue) and one may take $\mathbf{u}_1 = \mathbf{v}_1$.

Proof. Write $\mathrm{tr}(W\,\boldsymbol{\Sigma}_{m'}\,W^\top\,\boldsymbol{\Sigma}_m) = \|\boldsymbol{\Sigma}_m^{1/2}W\boldsymbol{\Sigma}_{m'}^{1/2}\|_F^2$ and using Frobenius-operator norm inequality as before,

$$\|\boldsymbol{\Sigma}_m^{1/2}W\boldsymbol{\Sigma}_{m'}^{1/2}\|_F^2 \leq \|\boldsymbol{\Sigma}_m^{1/2}\|_F^2\,\|W\|_{\mathrm{op}}^2\,\|\boldsymbol{\Sigma}_{m'}^{1/2}\|_F^2 = \mathrm{tr}(\boldsymbol{\Sigma}_m)\,\mathrm{tr}(\boldsymbol{\Sigma}_{m'})\|W\|_{\mathrm{op}}^2 = ab\,\sigma_{\max}(W)^2.$$

Plugging this into Theorem 2 yields the displayed upper bound on $\mathrm{Var}(I_{BV})$. For sharpness, let $\boldsymbol{\Sigma}_m = a\,\mathbf{u}_1\mathbf{u}_1^\top$ and $\boldsymbol{\Sigma}_{m'} = b\,\mathbf{v}_1\mathbf{v}_1^\top$. Then $\boldsymbol{\Sigma}_m^{1/2} = \sqrt{a}\,\mathbf{u}_1\mathbf{u}_1^\top$ and $\boldsymbol{\Sigma}_{m'}^{1/2} = \sqrt{b}\,\mathbf{v}_1\mathbf{v}_1^\top$, so

$$\boldsymbol{\Sigma}_m^{1/2} W \boldsymbol{\Sigma}_{m'}^{1/2} = \sqrt{ab}\,(\mathbf{u}_1^\top W \mathbf{v}_1)\,\mathbf{u}_1\mathbf{v}_1^\top = \sqrt{ab}\,\sigma_{\max}(W)\,\mathbf{u}_1\mathbf{v}_1^\top,$$

hence $\|\boldsymbol{\Sigma}_m^{1/2} W \boldsymbol{\Sigma}_{m'}^{1/2}\|_F^2 = ab\,\sigma_{\max}(W)^2$, achieving equality. □

Interpretation: Taken together, the above theorems and Corollary 2.1 show that, even under the null hypothesis $\boldsymbol{\Sigma}_{mm'} = \mathbf{0}$, the dispersion of I_{BV} can be large—on the order of the graph's strongest connectivity mode, as quantified by $\sigma_{\max}(W)$. Moreover, this dispersion is maximized *when each feature's spatial variability aligns with the same dominant graph pattern* (i.e., the leading singular vectors of W). Put simply, if the individual molecular expression profiles $\mathbf{X}^m$ and $\mathbf{X}^{m'}$ are each spatially autocorrelated yet independent, I_{BV} can take values far from zero even though its expectation tends to zero, particularly when their spatial structure is aligned.

2.5 Spatial fused horsehsoe and intrinsic GMRF

We reiterate, as discussed in the main text, that for a general adjacency graph G (not necessarily an MST), the proposed spatial fused horseshoe prior (and, analogously, the fused lasso) can be expressed as an intrinsic GMRF with an unfixed (random) precision structure of the following form:

$$\begin{aligned} \pi(\boldsymbol{\beta}_j^{mm'} \mid \Lambda_j, \tau_j^2, \sigma^2) &\propto (\tau_j^2\sigma^2)^{-\ell_{\text{rank}}/2} \exp\left\{ -\frac{1}{2\,\tau_j^2\sigma^2} \sum_{i=1}^{p} \frac{(\Delta\beta_i^{(j)})^2}{\Lambda_{ji}^2} \right\} \\ &= (\tau_j^2\sigma^2)^{-\ell_{\text{rank}}/2} \exp\left\{ -\frac{1}{2\,\tau_j^2\sigma^2}\, \boldsymbol{\beta}_j^{mm'\top} L(\Lambda_j) \boldsymbol{\beta}_j^{mm'} \right\},\ \ j \in \{0,1\}, \end{aligned} \tag{11}$$

where $D \in \mathbb{R}^{p\times n}$ is an oriented incidence matrix and $L(\Lambda_j) = D^\top \text{diag}(\Lambda_j^{-2})\, D$ is the weighted Laplacian matrix, with Λ_j^{-2} being the vector of edgewise precisions $\{\Lambda_{ji}^{-2}\}_{i=1}^p$, and $\ell_{\text{rank}} = \text{rank}\ (L(\Lambda_j)) = n - C$ where C is the number of connected components of the graph (typically, $C = 1$). When all local scales are set to one, $\Lambda_{ji} \equiv 1$, the prior reduces to the standard ICAR prior (up to a scale factor) [1]. The omitted normalizing factor [4] (the generalized determinant of $L(\Lambda_j)$) depends on Λ_j (and the graph) but not on $\boldsymbol{\beta}_j^{mm'}$ or τ_j^2; therefore, it plays no direct role in the Gibbs updates for $\boldsymbol{\beta}_j^{mm'}$ or τ_j^2. In our MCMC scheme

(Section 2.2), we update the local scales $\{\Lambda_{ji}\}_{i=1}^{p}$ using closed-form (conjugate) edge-wise full conditional distributions. This sampling scheme is exact on trees and, on general graphs, corresponds to a composite- or pseudo-likelihood approximation [22, 23].

2.6 Effects of cycles in the adjacency graph on the spatial fused horseshoe prior

Next, we investigate the implicit assumption of "independence" between the edge-wise differences: $\Delta\beta_i^{(j)}$'s in the spatial fused horseshoe prior (Eq. 4 of the main text). Let, $\mathbf{e}_j = [\Delta\beta_1^{(j)}, \Delta\beta_2^{(j)}, \ldots, \Delta\beta_p^{(j)}]^\top$. Since the individual components are assumed to be normally distributed "independently", the joint distribution of $\mathbf{e}_j$ can be written as $\mathbf{e}_j \sim MVN(0, D_j^*)$, $D_j^* = diag((\Lambda_{ji}^2\tau_j^2\sigma^2))_{i=1,\ldots,p}$. As discussed in the main text, $\mathbf{e}_j$ must also satisfy implicit constraints induced by cycles in the graph G. These constraints are absent when G is an MST, since an MST is acyclic. Let $\mathbf{A}$ be a $t \times p$ contrast matrix summarizing the constraints as $\mathbf{A}\mathbf{e}_j = \mathbf{0}$, where $1 \le t \le p$ denotes the number of cycles in E. Besag and Higdon (1999) [24] and Rue and Held (2005, Section 3.3.1) [4] have argued heuristically, in the context of intrinsic GMRF priors, that these cycle-induced constraints do not change the implied prior on $\boldsymbol{\beta}_j^{mm'}$ (Eq. 11); equivalently, the constraint $\mathbf{A}\mathbf{e}_j = \mathbf{0}$ need not be enforced explicitly. For completeness, we provide a formal proof for our setting in the following theorem.

Theorem 3. *The cycle constraint $\mathbf{A}\mathbf{e}_j = \mathbf{0}$ does not change the implied prior on $\boldsymbol{\beta}_j^{mm'}$, i.e., $\pi(\boldsymbol{\beta}_j^{mm'} \mid \cdot)$ as defined in Eq. 5 of the main text (and Eq. 11 here), up to a multiplicative normalizing constant.*

Proof. Note that $\pi(\boldsymbol{\beta}_j^{mm'} \mid \cdot) \propto \pi(\mathbf{e}_j)$ up to a constant (which depends on the random precision parameters), where $\mathbf{e}_j \sim MVN(\mathbf{0}, D_j^*)$. It therefore suffices to show that imposing the constraint $\mathbf{A}\mathbf{e}_j = \mathbf{0}$ does not alter the induced density/kernel in $\mathbf{e}_j$, i.e.,

$$\pi(\mathbf{e}_j \mid \mathbf{A}\mathbf{e}_j = \mathbf{0}) \propto \pi(\mathbf{e}_j).$$

By standard MVN results, conditional on the linear constraint $\mathbf{A}\mathbf{e}_j = \mathbf{0}$ the distribution of $\mathbf{e}_j$ is a degenerate MVN distribution (MVN_D) [25] with covariance

$$
\begin{aligned}
&\mathbf{e}_j \mid \mathbf{A}\mathbf{e}_j = \mathbf{0} \sim MVN_D(0, P_j), \qquad P_j = D_j^* - D_j^*\mathbf{A}^\top(\mathbf{A}D_j^*\mathbf{A}^\top)^{-1}\mathbf{A}D_j^*, \\
&\log \pi(\mathbf{e}_j \mid \mathbf{A}\mathbf{e}_j = \mathbf{0}) = -\frac{1}{2}\mathbf{e}_j^\top P_j^+ \mathbf{e}_j + c_j,
\end{aligned} \tag{12}
$$

where P_j^+ denotes the Moore–Penrose inverse of P_j, and c_j is a constant with respect to $\mathbf{e}_j$ (containing, in particular, the logarithm of the pseudo-determinant of P_j). An $\mathbf{e}_j$ satisfying $\mathbf{A}\mathbf{e}_j = \mathbf{0}$ can be obtained via the conditioning-by-kriging construction [4]: if $\mathbf{e}_j \sim MVN(0, D_j^*)$, then

$$
\mathbf{e}_j^* = \mathbf{e}_j - D_j^*\mathbf{A}^\top(\mathbf{A}D_j^*\mathbf{A}^\top)^{-1}(\mathbf{A}\mathbf{e}_j)
$$

satisfies $\mathbf{A}\mathbf{e}_j^* = \mathbf{0}$ and $\mathbf{e}_j^* \sim MVN_D(\mathbf{0}, P_j)$. However, this explicit transformation is not required for our claim, since we now show that the conditional kernel on the constrained subspace coincides with the unconditional kernel up to an additive constant.

Let us define, $M_j = I_n - D_j^{*1/2}\mathbf{A}^\top(\mathbf{A}D_j^*\mathbf{A}^\top)^{-1}\mathbf{A}D_j^{*1/2}$, so that $P_j = D_j^{*1/2}M_jD_j^{*1/2}$. Then M_j is symmetric and idempotent:

$$
\begin{aligned}
M_j^2 &= \left(I_n - D_j^{*1/2}\mathbf{A}^\top(\mathbf{A}D_j^*\mathbf{A}^\top)^{-1}\mathbf{A}D_j^{*1/2}\right)^2 \\
&= I_n - 2D_j^{*1/2}\mathbf{A}^\top(\mathbf{A}D_j^*\mathbf{A}^\top)^{-1}\mathbf{A}D_j^{*1/2} + D_j^{*1/2}\mathbf{A}^\top(\mathbf{A}D_j^*\mathbf{A}^\top)^{-1}\mathbf{A}D_j^*\mathbf{A}^\top(\mathbf{A}D_j^*\mathbf{A}^\top)^{-1}\mathbf{A}D_j^{*1/2} \\
&= I_n - D_j^{*1/2}\mathbf{A}^\top(\mathbf{A}D_j^*\mathbf{A}^\top)^{-1}\mathbf{A}D_j^{*1/2} = M_j.
\end{aligned}
$$

Hence, the Moore-Penrose inverse of M_j is itself, $M_j^+ = M_j$, and therefore

$$
P_j^+ = (D_j^{*1/2}M_jD_j^{*1/2})^+ = D_j^{*-1/2}M_j^+D_j^{*-1/2} = D_j^{*-1/2}M_jD_j^{*-1/2} = D_j^{*-1} - \mathbf{A}^\top(\mathbf{A}D_j^*\mathbf{A}^\top)^{-1}\mathbf{A}.
$$

Substituting this expression into Eq. (12) and using $\mathbf{A}\mathbf{e}_j = \mathbf{0}$ yields

$$
\begin{aligned}
\log \pi(\mathbf{e}_j \mid \mathbf{A}\mathbf{e}_j = \mathbf{0}) &= -\frac{1}{2}\mathbf{e}_j^\top\left(D_j^{*-1} - \mathbf{A}^\top(\mathbf{A}D_j^*\mathbf{A}^\top)^{-1}\mathbf{A}\right)\mathbf{e}_j + c_j \\
&= -\frac{1}{2}\mathbf{e}_j^\top D_j^{*-1}\mathbf{e}_j + c_j = \log \pi(\mathbf{e}_j) + c_j^*,
\end{aligned}
$$

for a constant c_j^* independent of $\mathbf{e}_j$. Thus, $\pi(\mathbf{e}_j \mid \mathbf{A}\mathbf{e}_j = \mathbf{0}) \propto \pi(\mathbf{e}_j)$, completing the proof. □

3 Real data results with kNN graphs

We repeated the melanoma and cSCC spatial transcriptomics analyses from the main text using a kNN graph with four neighbors (in place of the MST). As expected, the primary change was that the resulting

local estimates were smoother, with reduced posterior variance (i.e., greater stability), and the boundaries of transition regions became more apparent. Below, we report results for the same molecule pairs highlighted in the main text for each dataset.

3.1 Melanoma ST dataset

We summarize the melanoma results in Fig. 1. Using the global test, SpaceBF identified 72 LR pairs at a nominal significance level of 0.05 (42 at FDR 0.05). The larger number of discoveries is consistent with the improved stability of the local estimates; nevertheless, pairs can be prioritized by ranking their absolute posterior mean effects. For clearer visual separation, we clustered the slope surface estimates into four patterns rather than three, as in the main text (1C-D). The slope estimates remain similar to those obtained using the MST (Fig. 1E).

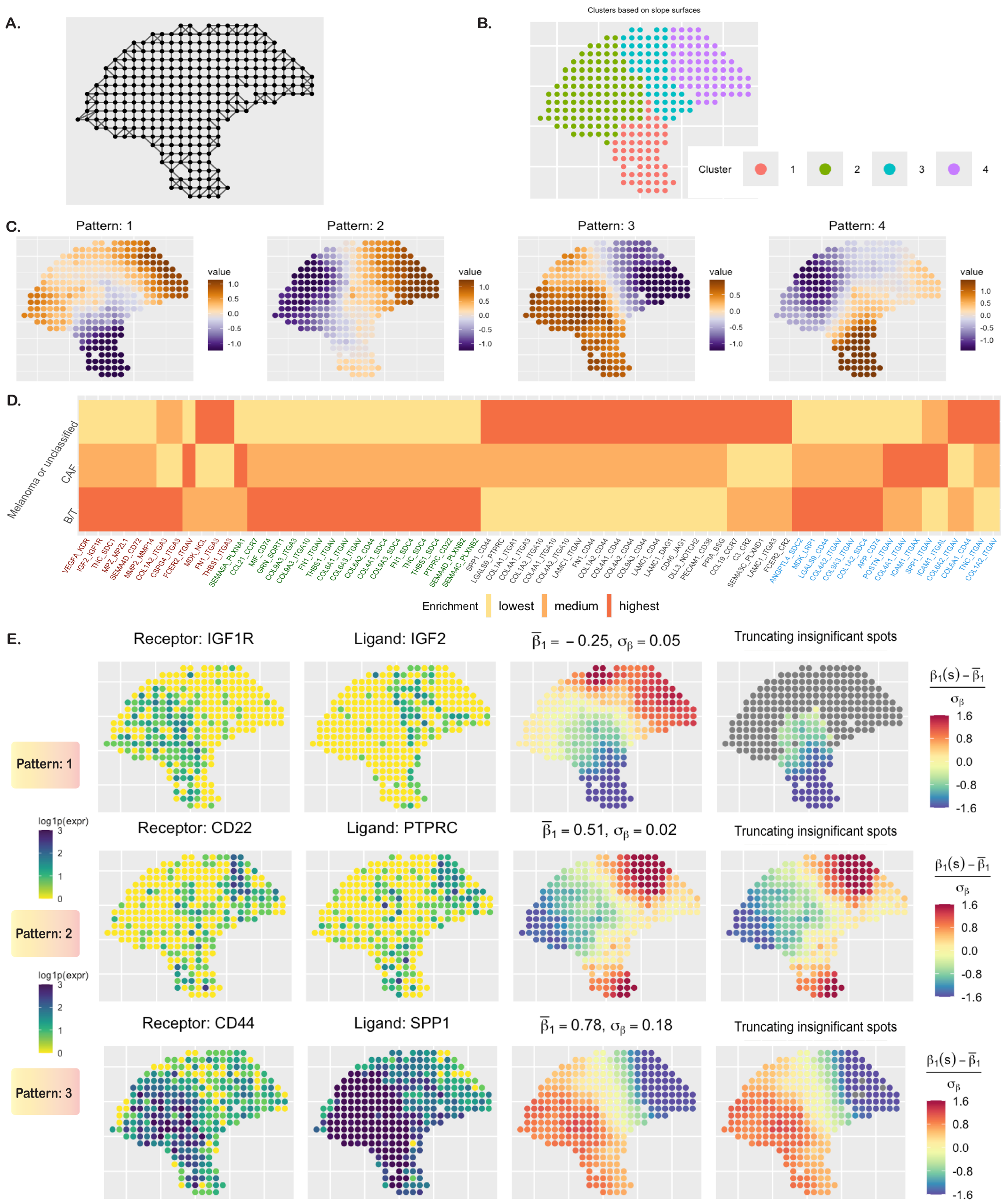

Figure 1: Cutaneous melanoma data analysis. **A**. kNN adjacency network. **B**. Clustering of spots based on centered and scaled estimates of slope surfaces of 72 statistically significant LR pairs. **C**. The four main spatial patterns of the estimated surfaces. **D**. Enrichment of LR interactions in three major cell types, with LR names arranged and color-coded according to their respective patterns. **E**. The first two columns show the expression of three LR pairs. The third column displays the centered and scaled slope surfaces. In the fourth column, insignificant spot-level slope estimates are greyed.

3.2 cSCC ST dataset

We summarize the cSCC results in Fig. 2. Using the kNN network, SpaceBF identified 45 keratin pairs as significant at the 0.05 level (all 45 at FDR < 0.05), consistent with the broad co-expression observed among keratins. We clustered the estimated slope surfaces into three spatial patterns (Fig. 2A). Relative to the MST-based results in the main text, these patterns are modestly different, but the surfaces are smoother and exhibit clearer transition boundaries. As shown in Fig. 2E, the estimated slope surfaces for (KRT17, KRT80), (KRT17, KRT78), and (KRT17, KRT6B) differ from their MST-based counterparts and are all assigned to pattern 1. For the first two pairs, estimation is particularly challenging due to sparsity, so some differences are expected; nevertheless, we consider the kNN-based surfaces more reliable in this setting because stronger local smoothing yields more stable estimates.

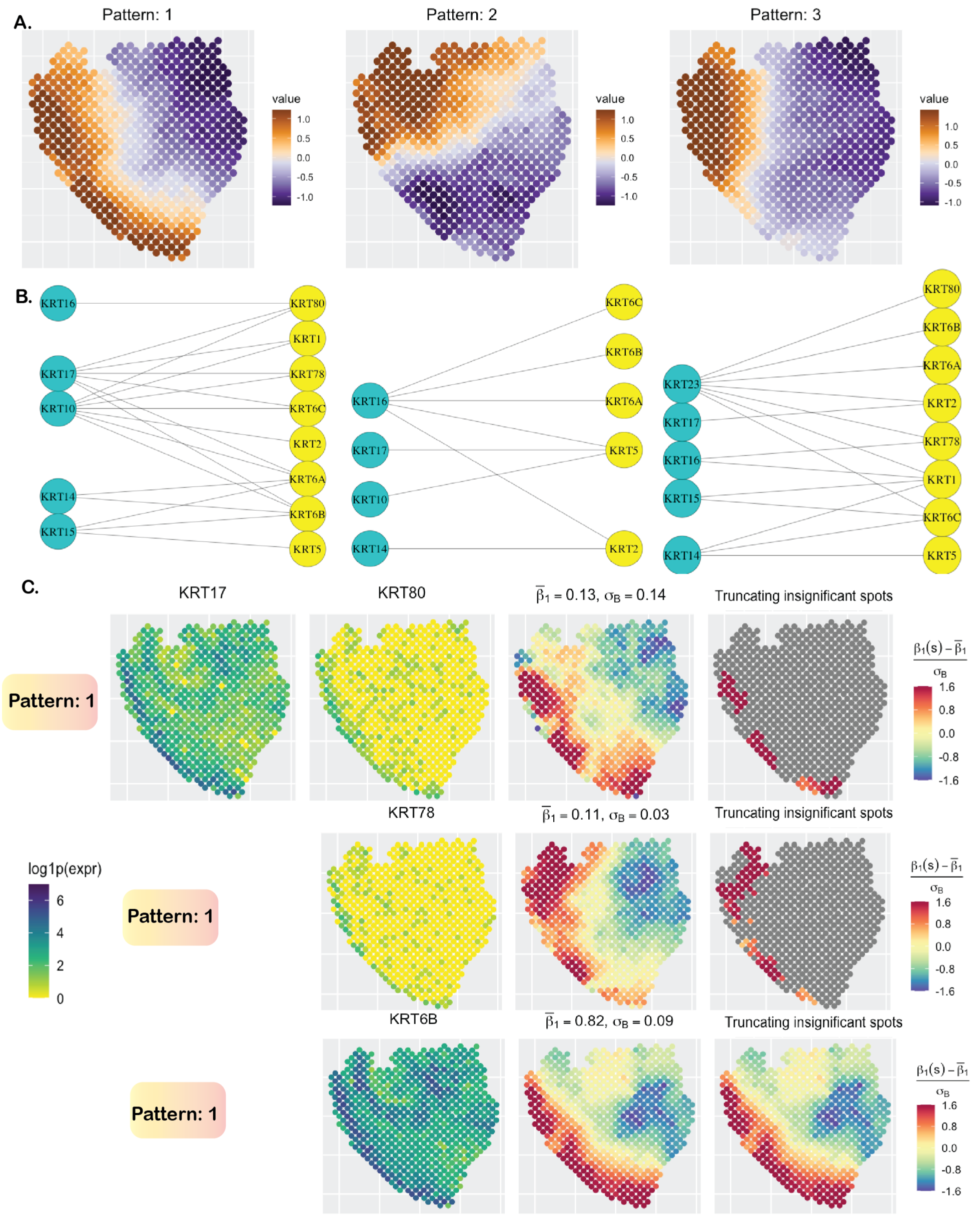

Figure 2: Cutaneous squamous cell carcinoma data analysis. **A**. The three main spatial patterns of the estimated surfaces. **B**. Bipartite graphs between Type I and Type 2 keratins based on their spatial pattern. **C**. Study of coexpression between the Type 1 keratin KRT17 and three different Type 2 keratins. The insignificant spot-level slope estimates are greyed in the last column.

4 Convergence plots

In the majority of our analyses, we considered 5,000 MCMC iterations with 2,500 burn-in. However, the algorithm typically converges within the first 1,000 iterations. Therefore, for datasets with a large number of locations, a smaller number of MCMC iterations may be sufficient and can be safely employed when computational resources are limited. For the convergence diagnostics, we computed the Geweke statistic [26] for each $\beta_1^{mm'}(s_k)$, implemented in the *R* package *coda* [27], and investigated the trace plots of a few randomly chosen $\beta_1^{mm'}(s_k)$'s (Figs. 3 and 4). When either the variable m or m' is highly sparse ($> 75\%$ zeroes), imposing additional normal priors on $\beta_0^{mm'}(s_k)$'s and $\beta_1^{mm'}(s_k)$'s with a moderately large variance, such as $N(0, 10)$, drastically improves mixing and overall convergence performance.

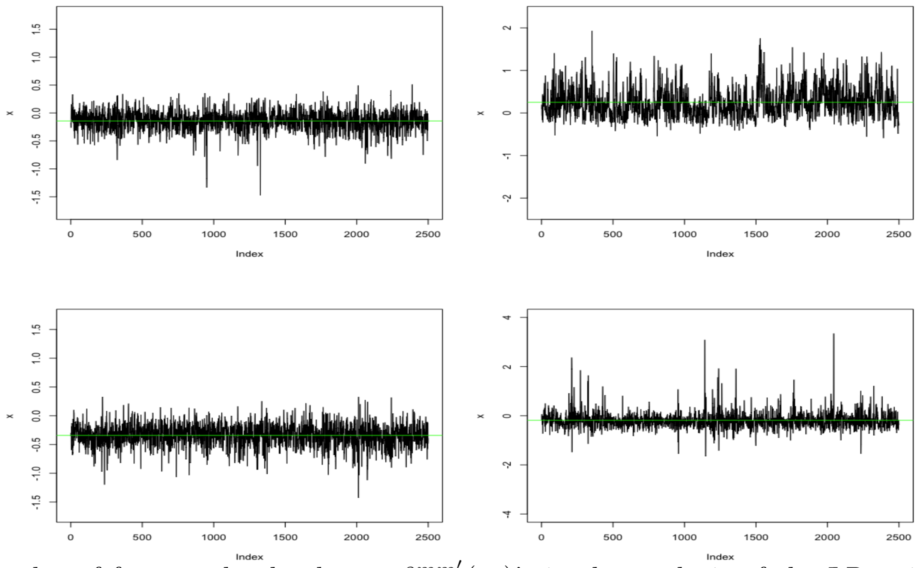

Figure 3: Trace plot of four randomly chosen $\beta_1^{mm'}(s_k)$'s in the analysis of the LR pair: (IGF2, IGF1R) from the melanoma dataset.

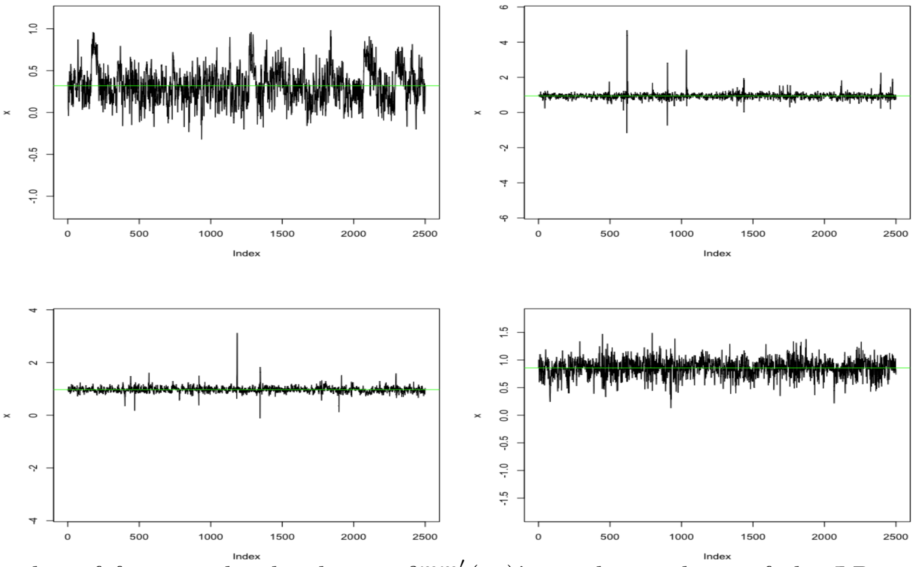

Figure 4: Trace plot of four randomly chosen $\beta_1^{mm'}(s_k)$'s in the analysis of the LR pair: (SPP1, CD44) from the melanoma dataset.

References

[1] S. Banerjee, B. P. Carlin, and A. E. Gelfand. *Hierarchical modeling and analysis for spatial data.* Chapman and Hall/CRC, 2014.

[2] H. Lu, C. S. Reilly, S. Banerjee, and B. P. Carlin. Bayesian areal wombling via adjacency modeling. *Environmental and ecological statistics*, 14:433–452, 2007.

[3] M. L. Lavine and J. S. Hodges. On rigorous specification of ICAR models. *The American Statistician*, 66(1):42–49, 2012.

[4] H. Rue and L. Held. *Gaussian Markov random fields: theory and applications.* Chapman and Hall/CRC, 2005.

[5] J. Besag, J. York, and A. Mollié. Bayesian image restoration, with two applications in spatial statistics. *Annals of the institute of statistical mathematics*, 43:1–20, 1991.

[6] K. V. Mardia, J. T. Kent, and C. C. Taylor. *Multivariate analysis.* John Wiley & Sons, 2024.

[7] S. Banerjee, A. E. Gelfand, A. O. Finley, and H. Sang. Gaussian predictive process models for large spatial data sets. *Journal of the Royal Statistical Society Series B: Statistical Methodology*, 70(4):825–848, 2008.

[8] A. Datta, S. Banerjee, A. O. Finley, and A. E. Gelfand. Hierarchical nearest-neighbor Gaussian process models for large geostatistical datasets. *Journal of the American Statistical Association*, 111(514):800–812, 2016.

[9] T. Park and G. Casella. The bayesian lasso. *Journal of the american statistical association*, 103(482):681–686, 2008.

[10] M. Pontil, S. Mukherjee, and F. Girosi. On the noise model of support vector machines regression. In *Algorithmic Learning Theory: 11th International Conference, ALT 2000 Sydney, Australia, December 11–13, 2000 Proceedings 11*, pages 316–324. Springer, 2000.

[11] E. Makalic and D. F. Schmidt. A simple sampler for the horseshoe estimator. *IEEE Signal Processing Letters*, 23(1):179–182, 2015.

[12] J. Pillow and J. Scott. Fully Bayesian inference for neural models with negative-binomial spiking. *Advances in neural information processing systems*, 25, 2012.

[13] N. G. Polson, J. G. Scott, and J. Windle. Bayesian inference for logistic models using Pólya–Gamma latent variables. *Journal of the American statistical Association*, 108(504):1339–1349, 2013.

[14] S. Z. Dadaneh, M. Zhou, and X. Qian. Bayesian negative binomial regression for differential expression with confounding factors. *Bioinformatics*, 34(19):3349–3356, 2018.

[15] A. C. Rencher and G. B. Schaalje. *Linear models in statistics.* John Wiley & Sons, 2008.

[16] S. Richardson and P. Clifford. Testing association between spatial processes. *Lecture Notes-Monograph Series*, pages 295–308, 1991.

[17] M. R. Dale and M.-J. Fortin. Spatial autocorrelation and statistical tests: some solutions. *Journal of Agricultural, Biological, and Environmental Statistics*, 14:188–206, 2009.

[18] D. A. Griffith. *Advanced spatial statistics: special topics in the exploration of quantitative spatial data series*, volume 12. Springer Science & Business Media, 2012.

[19] D. Wartenberg. Multivariate spatial correlation: a method for exploratory geographical analysis. *Geographical analysis*, 17(4):263–283, 1985.

[20] N. Dunford and J. T. Schwartz. *Linear operators, part 1: general theory.* John Wiley & Sons, 1988.

[21] A. R. Rao and P. Bhimasankaram. *Linear algebra*, volume 19. Springer, 2000.

[22] J. Besag. Statistical analysis of non-lattice data. *Journal of the Royal Statistical Society Series D: The Statistician*, 24(3):179–195, 1975.

[23] B. G. Lindsay. Composite likelihood methods. In *Statistical Inference from Stochastic Processes: Proceedings of the AMS-IMS-SIAM Joint Summer Research Conference Held August 9-15, 1987, with Support from the National Science Foundation and the Army Research Office*, volume 80, page 221. American Mathematical Soc., 1988.

[24] J. Besag and D. Higdon. Bayesian analysis of agricultural field experiments. *Journal of the Royal Statistical Society Series B: Statistical Methodology*, 61(4):691–746, 1999.

[25] C. R. Rao. *Linear statistical inference and its applications*, volume 2. Wiley New York, 1973.

[26] J. Geweke. Evaluating the accuracy of sampling-based approaches to the calculation of posterior moments. Technical report, Federal Reserve Bank of Minneapolis, 1991.

[27] M. Plummer, N. Best, K. Cowles, and K. Vines. Package 'coda'. *URL http://cran. r-project. org/web/packages/coda/coda. pdf, accessed January*, 25:2015, 2015.
