# Supplementary material for: SpaceBF: spatial coexpression analysis using Bayesian fused approaches in spatial omics datasets
Source: Gigascience. 2026 Jan 20;15:giag006. doi: 10.1093/gigascience/giag006 (PMC12954175; doi:10.1093/gigascience/giag006)
Supplement: giag006_GIGA-D-25-00259_Original_Submission [file giag006_giga-d-25-00259_original_submission.pdf]

GigaScience

SpaceBF: Spatial coexpression analysis using Bayesian Fused approaches in spatial omics datasets

--Manuscript Draft--

**Manuscript Number:**	GIGA-D-25-00259
**Full Title:**	SpaceBF: Spatial coexpression analysis using Bayesian Fused approaches in spatial omics datasets
**Article Type:**	Research
**Funding Information:**	NIH Clinical Center — Dr. Souvik Seal
**Abstract:**	Advancements in spatial omics technologies have enabled the measurement of expression profilesof different molecules, such as genes (using spatial transcriptomics), and peptides, lipids, or N-glycans(using mass spectrometry imaging), across thousands of spatial locations within a tissue. While identifyingindividual molecules with spatially variable expression is a well-studied statistical problem, robustmethodologies for detecting spatially varying co-expression between molecule pairs remain limited.To address this gap, we introduce a Bayesian fused modeling framework for estimating molecular coexpressionat both local (location-specific) and global (tissue-wide) levels, offering a refined understandingof cell-cell communication (CCC) mediated through ligand-receptor and other molecular interactions.Through extensive simulations, we demonstrate that our approach, termed SpaceBF, achieves superiorspecificity and power compared to existing methods that predominantly rely on geospatial metrics such asbivariate Moran’s I and Lee’s L. Applying our framework to real spatial transcriptomics and proteomicsdatasets, we uncover novel biological insights into molecular interactions across different cancers.
**Corresponding Author:**	Souvik Seal<br>Medical University of South Carolina<br>CHARLESTON, SC UNITED STATES
**Corresponding Author Secondary Information:**	
**Corresponding Author's Institution:**	Medical University of South Carolina
**Corresponding Author's Secondary Institution:**	
**First Author:**	Souvik Seal
**First Author Secondary Information:**	
**Order of Authors:**	Souvik Seal<br>Brian Neelon
**Order of Authors Secondary Information:**	
**Additional Information:**	
**Question**	**Response**
Are you submitting this manuscript to a special series or article collection?	No
**Experimental design and statistics**<br><br>Full details of the experimental design and statistical methods used should be given in the Methods section, as detailed in our Minimum Standards Reporting Checklist.	Yes

Powered by Editorial Manager® and ProduXion Manager® from Aries Systems Corporation

Information essential to interpreting the data presented should be made available in the figure legends.<br><br>Have you included all the information requested in your manuscript?	
**Resources**<br><br>A description of all resources used, including antibodies, cell lines, animals and software tools, with enough information to allow them to be uniquely identified, should be included in the Methods section. Authors are strongly encouraged to cite Research Resource Identifiers (RRIDs) for antibodies, model organisms and tools, where possible.<br><br>Have you included the information requested as detailed in our Minimum Standards Reporting Checklist?	Yes
**Availability of data and materials**<br><br>All datasets and code on which the conclusions of the paper rely must be either included in your submission or deposited in publicly available repositories (where available and ethically appropriate), referencing such data using a unique identifier in the references and in the "Availability of Data and Materials" section of your manuscript.<br><br>Have you have met the above requirement as detailed in our Minimum Standards Reporting Checklist?	Yes
GigaScience has policies and guidelines in place for the use of generative AI-writing tools such as ChatGPT. If you have used such writing tools to assist with writing the manuscript this must be declared and cited in the text. Authors should not list AI-writing tools and other AI-assisted technologies as an author or	No

co-author and should acknowledge that they are fully responsible for text generated or refined by AI-writing tools.<p><br><br>A summary of use (particularly in the introduction or among methods) needs to be included at the end of the paper, and the outputs should also be included as a supplementary file hosted in GigaDB or other open repositories. Please read our guidelines for more information. <p><br><br>By submitting to GigaScience, you are aware of the journal's AI-writing tools policy, and if you have declared use of such tools below, you have acknowledged this where appropriate in your manuscript and have made a summary of use and outputs available. <p><br><b>AI-assisted writing tools have been used in the preparation of this manuscript?	

Manuscript Click here to access/download;Manuscript;SpaceBF_main_GigaScience.pdf

SpaceBF: Spatial coexpression analysis using Bayesian Fused approaches in spatial omics datasets

Souvik Seal and Brian Neelon

Department of Public Health Sciences, College of Medicine, Medical University of South Carolina, Charleston, USA

June 2025

Abstract

Advancements in spatial omics technologies have enabled the measurement of expression profiles of different molecules, such as genes (using spatial transcriptomics), and peptides, lipids, or N-glycans (using mass spectrometry imaging), across thousands of spatial locations within a tissue. While identifying individual molecules with spatially variable expression is a well-studied statistical problem, robust methodologies for detecting spatially varying co-expression between molecule pairs remain limited. To address this gap, we introduce a Bayesian fused modeling framework for estimating molecular co-expression at both local (location-specific) and global (tissue-wide) levels, offering a refined understanding of cell-cell communication (CCC) mediated through ligand-receptor and other molecular interactions. Through extensive simulations, we demonstrate that our approach, termed SpaceBF, achieves superior specificity and power compared to existing methods that predominantly rely on geospatial metrics such as bivariate Moran's I and Lee's L. Applying our framework to real spatial transcriptomics and proteomics datasets, we uncover novel biological insights into molecular interactions across different cancers.

Keywords: Spatial co-expression, CCC, Bayesian fusion, Horseshoe prior, Bivariate association, Spatial transcriptomics

1 Introduction

Technological advances in spatial omics [1, 2, 3] have enabled *in situ* profiling of varying molecules, including genes (via spatial transcriptomics (ST)) [4, 5, 6, 7], lipids or peptides (using mass spectrometry imaging (MSI)) [8, 9, 10, 11], and immune proteins (through multiplex immunofluorescence (mIF)) [12, 13, 14, 15], within tissues. The technologies offer distinct yet complementary biological insights, differing in spatial resolution and the number of detectable molecules (throughput). For example, the next-generation sequencing (NGS)-based ST platform Visium (from 10X Genomics) [16] offers transcriptome-wide gene-expression profiling (throughput $\sim 20,000$) at a 55 μm spot-level resolution. MALDI MSI-based platforms (from Bruker Daltonics [17] and others) offer profiling different types of molecules, such as peptides, lipids, nucleotides, proteins, metabolites, and N-glycans, (throughput $\sim 50-1000$) at 10 μm spot-level resolution. The mIF platform PhenoCycler (from Akoya Biosciences) [18] enables protein profiling (throughput ~ 40) at a 0.6 μm cellular resolution. Despite these differences, the underlying data structure remains largely consistent across technologies and platforms, comprising a collection of spatial locations (from single or multiple samples) with observed expression or intensity of various molecules. Consequently, common biostatistical questions arise, centering the spatial dynamics of molecules within the complex tissue or tumor microenvironment (TME) [19, 20, 21, 22, 23].

In the context of ST datasets, identifying spatially variable genes (SVGs), i.e., the genes exhibiting spatially structured expression patterns across the tissue, has gained significant attention [24, 25, 26, 27, 28, 29, 30, 31, 32, 33, 34, 35, 36, 37, 38]. It enables critical downstream analyses such as discovering potential biomarkers and defining tissue regions that influence cellular differentiation and function [39, 40, 41, 42]. Analogously, for mIF or imaging mass cytometry (IMC) datasets, innovative methods [43, 44, 45, 46, 47, 48, 49, 50, 51] have been proposed to understand the spatial distribution of immune cell types (defined by binarizing the expression profile of immune proteins) across the TME. Building upon this univariate framework, which typically analyzes one molecule at a time, another widely investigated problem has been spatial domain detection, i.e., deconvolving the tissue into distinct, spatially contiguous neighborhoods based on multivariate gene expression (ST) [52, 53, 54, 55, 56, 57, 58, 59, 60, 61, 62, 63] or immune cell

type composition (mIF) [64, 65, 66, 67, 68, 69]. It aids mapping the molecular and functional landscape of tissues, elucidating disease progression, and guiding targeted therapies [70, 71, 72]. While some of the referenced methods can be adapted for use with MSI datasets, it is important to underscore the lack of sophisticated spatial functionalities of the existing bioinformatics toolboxes [73, 74, 75, 76].

While univariate and multivariate spatial analyses have garnered significant attention, a critical intermediate task remains underexplored: bivariate spatial co-expression analysis of molecular pairs at both "local" (spot/cell-specific) and "global" (tissue-wide) levels, aimed at precisely characterizing the spatial interaction or binding pattern of any two molecules throughout the tissue plane. To emphasize the importance of such an analysis, we review the concepts of cell-cell communication (CCC) [77, 78, 79, 80]. CCC is a fundamental biological process through which cells exchange information via direct contact or signaling molecules (ligands) binding to receptor molecules present on the same or different cells. It regulates essential biological functions, including tissue development [81] and immune responses [82], and its disruption has been implicated in the onset and progression of cancer [83]. Autocrine, juxtacrine, and paracrine signaling are three major pathways of CCC [84]. In autocrine signaling, ligands released by a cell bind to receptors on the same cell, while in juxtacrine and paracrine signaling, the ligands target adjacent and nearby cells. The study of ligand-receptor interactions (LRI), which involves identifying gene pairs (ligands and receptors) that show coordinated upregulation or downregulation across groups of cells, has become a fundamental approach for inferring CCC from single-cell RNA sequencing (scRNA-seq) datasets [85, 86, 87, 88, 89, 90, 91, 92, 93]. However, these approaches are prone to false positive interactions due to the lack of spatial context in scRNA-seq datasets, treating distant cell pairs similarly to nearby ones [94, 95, 96], which potentially leads to an overestimation of juxtacrine and paracrine signaling. ST datasets offer a natural avenue for improvement by enabling spatially constrained LRI analysis.

A limited number of tools exist for spatial LRI analysis or, more broadly, for assessing bivariate spatial co-expression of molecules in ST or MSI datasets. It should be emphasized that bivariate co-expression can manifest in two ways: (a) joint over- or under-expression within the same cells (correlation) and (b) joint over- or under-expression in neighboring cells (cross-correlation [97]). Some relevant methods

include MERINGUE [98], Giotto [99], SpaGene [100], SpaTalk [101], SpatialDM [102], CellChat V2 [103], LIANA+ [104], and Copulacci [105]. We skip the approaches that jointly analyze multiple LR pairs [106, 107]. Methods such as MERINGUE, Giotto, and SpaTalk provide only a global summary of spatial co-expression across a tissue, whereas others also offer local (spot/cell-specific) estimates. Let the standardized expression of two genes (m, m') be $X^m(s)$ and $X^{m'}(s)$ at location s for $s \in \{s_1, \ldots, s_n\}$, and $X^m = (X^m(s_1), \ldots, X^m(s_n))^T$, $X^{m'} = (X^{m'}(s_1), \ldots, X^{m'}(s_n))^T$. For a global summary of spatial co-expression, MERINGUE and SpatialDM leverage a popular geospatial metric termed the bivariate Moran's I (I_{BV}) [108, 109], interpreted as the Pearson correlation between one variable and the spatial lagged version of the other [110, 111, 112]. Mathematically, $I_{BV} \propto (X^m)^T W X^{m'}$, where $W = [[w_{k_1 k_2}]]$ is the spatial weight matrix that controls the spatial lagging. As W, MERINGUE uses a binary adjacency matrix based on the Delaunay triangulation [113] of the spatial locations ($w_{k_1 k_2} = 1$ if locations (s_{k_1}, s_{k_2}) are connected, or 0 otherwise). SpatialDM uses a kernel covariance matrix or Gram matrix [114] based on the L_2 distance between locations ($w_{k_1 k_2} = k_l(|s_{k_1} - s_{k_2}|^2)$, where k_l is a kernel function with lengthscale parameter l [115]). For local estimates of spatial correlation, SpatialDM considers the bivariate local Moran's I ($I_{BV}^{local}(s)$) based on the local indicators of spatial association (LISA) approach [116]. The LIANA+ toolbox implements SpatialDM and introduces a similar spatially weighted cosine similarity index. Of note, a newer package named Voyager [117] considers Lee's L statistic [110], which has a slightly different formulation than I_{BV}. A critical yet often overlooked aspect of ST data analysis is that gene expression, measured in terms of unique molecular identifier (UMI) count, is inherently a discrete random variable (RV). However, the above methods assume normality upon a variance-stabilizing transformation [118, 119, 120], which may obscure true signals and have been widely criticized both within the ST literature [26, 55, 121, 122] and in broader contexts [123, 124, 125, 126]. Addressing this issue, Copulacci models a pair of genes as bivariate Poisson-distributed RVs, with their correlation in spatially adjacent cells captured using a Gaussian copula [127]. For inference, these methods typically rely on a permutation test [128].

Bivariate Moran's I (I_{BV}) and Lee's L statistic, as implemented in MERINGUE, SpatialDM, LIANA+, and Voyager, are primarily recommended as exploratory metrics for assessing cross-correlation rather than

as rigorous hypothesis testing tools [129, 97], in traditional spatial statistical literature. In simulation studies (see Section 2.2), we have shown that even when two variables are independently simulated with certain spatial covariance structures, the unmodeled spatial autocorrelation introduces a confounding effect on the bivariate association, leading to significantly inflated type 1 error rates. A similar issue is well documented, as extensive literature highlights the limitations of using simple Pearson correlation to assess dependencies between two variables in the presence of spatial autocorrelation [130, 131, 132, 133, 134]. By extension, since I_{BV} and Lee's L are both fundamentally based on Pearson correlation between spatially lagged variables, they may be susceptible to similar pitfalls. Furthermore, these spatially weighted association indices, being model-free, are unable to seamlessly adjust for cell-level covariates such as cell type, a limitation also present in Copulacci. As a side note, mapping to the aforementioned CCC pathways, Pearson correlation between ligand and receptor can be interpreted as a proxy for autocrine signaling, while cross-correlation may reflect a combination of juxtacrine and paracrine signaling.

We approach the bivariate spatial co-expression detection as a generalized linear regression problem, modeling a molecule m as the outcome and the other molecule m' as the predictor (see Section 4). For ST datasets, gene expression or UMI count is modeled as an overdispersed negative binomial (NB)-distributed RV [135], while an alternative Gaussian model is considered for continuous cases. The regression coefficients, both intercept ($\beta_0^{mm'}(s)$) and slope ($\beta_1^{mm'}(s)$), are assumed to vary across locations (s) exhibiting spatial dependency. Known as the spatially varying coefficients (SVC) model [136], this framework provides exceptional flexibility and precision in capturing locally changing co-expression patterns through $\beta_1^{mm'}(s)$. A large positive $\beta_1^{mm'}(s)$ suggests strong positive co-expression at location s, i.e., joint up or down-regulation, whereas a large negative value indicates avoidance or repulsion. The average of $\beta_1^{mm'}(s)$'s, $\overline{\beta_1^{mm'}} = \sum_k \beta_1^{mm'}(s_k)/n$, provides a summary of the global co-expression pattern. Similar models have been widely used in fields such as disease mapping [137, 138], econometrics [139, 140], ecological studies [141, 142], and neuroimaging research [143, 144]. In the Bayesian paradigm, the spatial dependency between $\beta_0^{mm'}(s)$'s and $\beta_1^{mm'}(s)$'s is typically modeled using a conditionally autoregressive (CAR) [145, 146, 147] or Gaussian process (GP) priors [148, 97, 149]. In contrast, we introduce a locally

adaptive spatial Gaussian Markov random field (GMRF) prior [150] based on the concepts of fusion penalties [151, 152, 153] and horseshoe prior [154, 155, 156], extending a related work in the frequentist setup [157]. Briefly, the prior incorporates the spatial similarity between two adjacent locations, (s_{k_1}, s_{k_2}), by encouraging $|\beta_0^{mm'}(s_{k_1}) - \beta_0^{mm'}(s_{k_2})| \approx 0$ and $|\beta_1^{mm'}(s_{k_1}) - \beta_1^{mm'}(s_{k_2})| \approx 0$. Adjacency is defined using the minimum spanning tree (MST) [158, 159, 160] constructed with the L_2 distances between locations as edge weights. The MST of a connected, weighted graph offers the most economical connectivity between all vertices without cycles, minimizing the total edge weight. A detailed discussion on the choice of MST as the underlying spatial graph, along with its implications for the resulting GMRF prior, is provided. The proposed method, SpaceBF, is evaluated against existing approaches using realistic simulation scenarios, showcasing its high specificity and power. It is further applied to three real datasets: a) an ST dataset on cutaneous melanoma [39] for spatial LRI analysis, b) an ST dataset on cutaneous squamous cell carcinoma [161] for keratin-interaction analysis, and c) a spatial proteomics dataset on ductal carcinoma in situ (DCIS) from the Medical University of South Carolina (MUSC) for peptide co-localization analysis.

2 Result

2.1 Real data analysis

2.1.1 Melanoma ST dataset

We analyzed a cutaneous melanoma dataset [39] from a long-term survivor (10+ years), collected using the ST technology [4], comprising 293 spots, each 100 μm in size and at a 200 μm center-to-center distance. There are 16,148 genes, forming 1,180 known ligand-receptor (LR) pairs as available from CellChatDB [88]. There are three major pathologist-annotated regions as seen in the histology image (Fig. 1A), collected from Thrane et al. (2018), and 6 major cell types (Fig. 1B) predicted using the RCTD [162] package based on overall gene expression [102]. After filtering out genes with extremely low expression ($< 0.2 \times 293 \approx 59$ reads), 161 LR pairs remain, which were examined using our method SpaceBF. To briefly summarize the SpaceBF workflow, it first constructs an MST based on the spatial coordinates of

the spots (Fig. 1C). Then, for every LR pair: (m', m), it considers Eq. 2 with the receptor expression as $X^m(s_k)$ and the ligand expression as $X^{m'}(s_k)$, and s_k representing a spot. Following parameter estimation via a Markov Chain Monte Carlo (MCMC) procedure, the framework performs two hypothesis tests to assess the significance of spatial co-expression at both global and local levels (see Section 4.3). Using the global test in this dataset, SpaceBF identified 53 LR pairs at a significance level 0.05 (33 at an FDR of 0.1). The estimated slope surface $\beta_1^{mm'}(s_k)$ of different LR pairs exhibits distinct patterns. To highlight these differences, we classify the detected LR pairs into 3 major patterns (Fig. 1E) based on hierarchical clustering [163] of the standardized vector $\boldsymbol{\beta}_1^{mm'*} = (\beta_1^{mm'}(s_1) - \overline{\beta_1^{mm'}}, \ldots, \beta_1^{mm'}(s_n) - \overline{\beta_1^{mm'}})^T / \sigma_\beta^{mm'}$, where $\overline{\beta_1^{mm'}} = \sum_k \beta_1^{mm'}(s_k)/n$ and $\sigma_\beta^{mm'}$ are the tissue-wide average and the SD of estimated $\beta_1^{mm'}(s_k)$'s, respectively. 20 LR pairs follow pattern 1, while 22 and 11 LR pairs correspond to patterns 2 and 3, respectively. Similarly, the spots are grouped into 4 clusters based on the spot-level vectors of slopes corresponding to the 53 detected LR pairs (Fig. 1D). It is evident that clusters 1 and 3 correspond to the melanoma region, while clusters 2 and 4 loosely correspond to the stroma and lymphoid regions, respectively. Returning to the LR patterns, in Fig. 1F, the LR pairs are arranged sequentially from pattern 1 to 3, highlighting the enrichment of their interaction in three major cell types. For example, $\sum_{k \in \text{B/T cells}} \beta_1^{mm'*}(s_k)$ represents the enrichment within B/T cells relative to the average enrichment $\overline{\beta_1^{mm'}}$ and scaled by the SD. The levels "highest," "medium," and "lowest" indicate the degree of enrichment, with "highest" corresponding to the greatest or most positive enrichment and so on. The majority of LR pairs following pattern 1 exhibit higher or more positive interaction in B/T cells within the lymphoid region (some in CAF cells) and more negative interaction (avoidance or repulsion) in the melanoma region or cells. Pattern 2 mostly corresponds to LR pairs with the highest enrichment in CAF cells, while pattern 3 clearly corresponds to the pairs with the highest enrichment in melanoma cells. Next, we investigate the biological relevance of the estimated slope surfaces for a selected set of LR pairs. The LR pair (IGF2, IGF1R) [164] corresponds to pattern 1 and demonstrates a negative association overall, with an estimated average slope of $\overline{\beta_1^{mm'}} = -0.212$, and the p-value $= 0.024$, which is consistent with a visual inspection (Fig. 1G). It could indicate a lack of binding between these genes, which would be a generally favorable

factor for the survivor [165]. Setting the insignificant $\beta_1^{mm'*}(s_k)$ values to 0 based on the local test, the negative interaction found in the melanoma region has the highest credibility. The pair (PTPRC, CD22) [166] follows pattern 2, with $\overline{\beta_1^{mm'}} = 0.422$ and p-value of 6.28×10^{-6}. PTPRC, also known as CD45, is a facilitator of T-cell receptor (TCR) and B-cell receptor (BCR) signaling [167], while CD22 is primarily an inhibitor of BCR signaling [168]. Their overall positive co-expression, particularly in the lymphoid region, is likely associated with a balanced B cell regulation, helping to prevent autoimmunity and promoting lymphoid growth in other regions as part of the immune response. The final LR pair we discuss is (SPP1, CD44) [169], which follows pattern 3, exhibiting a highly positive overall co-expression with $\overline{\beta_1^{mm'}} = 0.79$ and p-value of 1.03×10^{-6}. This strong interaction displays a decreasing gradient from the melanoma region to the lymphoid region, which aligns with its known role in dysregulated cytoskeletal remodeling [170], facilitating melanoma cell invasion into surrounding tissues.

2.1.2 cSCC ST dataset

We analyzed a cutaneous squamous cell carcinoma (cSCC) dataset [161] on a patient sample with a histopathologic subtype of "moderately differentiated" cSCC [171]. The dataset was collected using the ST technology with 621 spots, each of size 110 μm and a center-to-center distance of 150 μm. There are $16,643$ genes of which 45 are keratins (14 after filtering low-count genes, $< 0.2 \times 621 \approx 124$ reads). These keratins can be classified into two types: 1) type 1, which includes KRT10, KRT14–KRT17, and KRT23, and 2) type 2, which includes KRT1, KRT2, KRT5, KRT6A, KRT6B, KRT6C, KRT78, and KRT80. The keratins pair together to form intermediate filaments, providing structural support to epithelial cells [172]. In the context of cSCC and other carcinomas, keratins are emerging as highly significant targets for therapeutic intervention [173, 174, 175]. Of note, some of the keratins belong to the GO term: "keratinocyte differentiation" (GO:0030216) and were reported to exhibit strong spatial correlation in an earlier work [176] involving the same dataset. We utilized SpaceBF to investigate the binding between type 1 and type 2 keratins, resulting in a set of 48 keratin pairs. In the histology image (Fig. 2A), the deep blue areas at the top and left sides correspond to tumor regions, while the whitish region at the bottom represents a

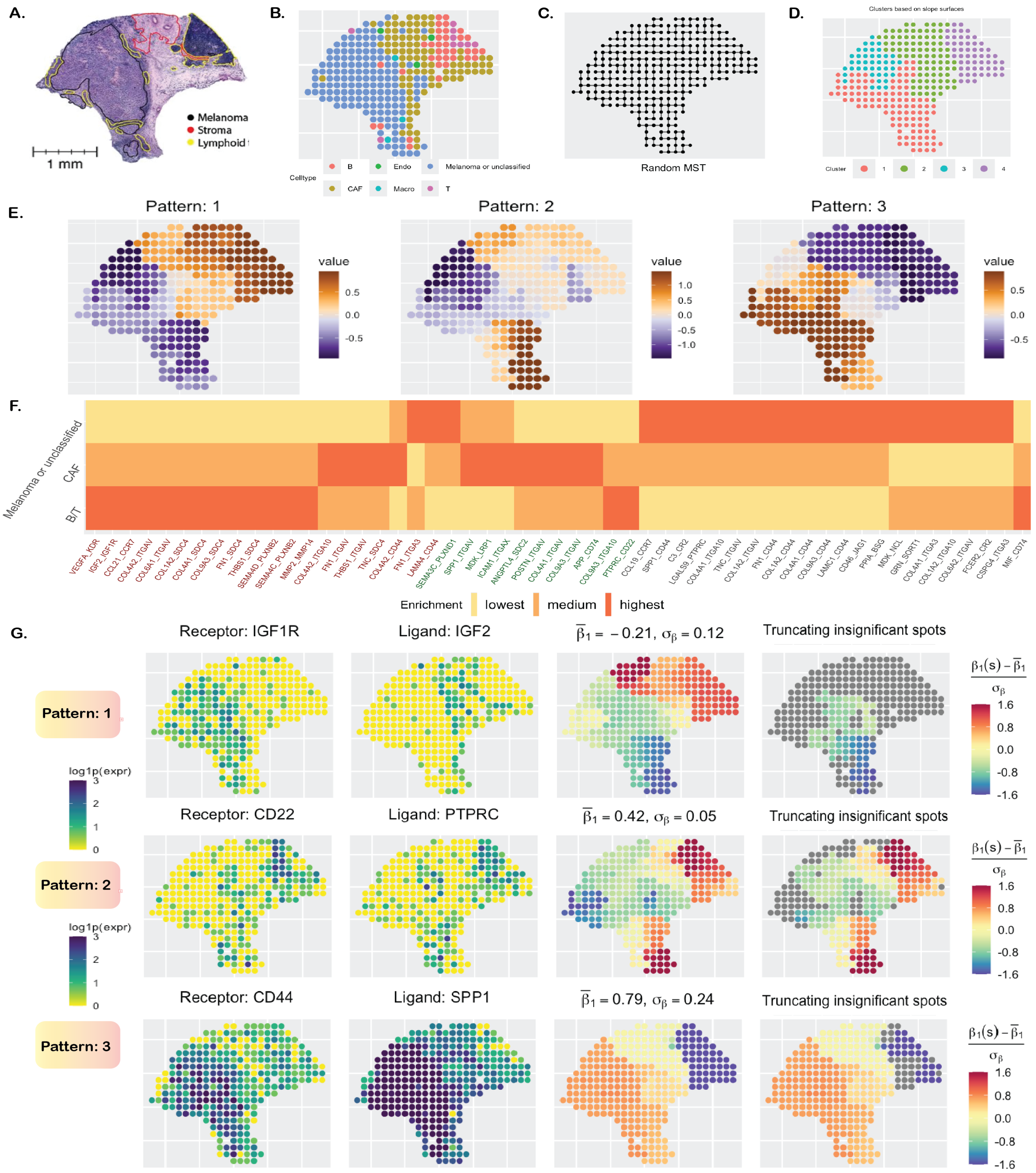

Figure 1: Cutaneous melanoma data analysis. **A**. Annotated H&E-stained image. **B**. Cell types based on gene expression. **C**. Minimum spanning tree (MST) capturing the spatial structure. **D**. Clustering of spots based on centered and scaled estimates of slope surfaces of 53 statistically significant LR pairs. **E**. The three main spatial patterns of the estimated surfaces. **F**. Enrichment of LR interactions in three major cell types, with LR names arranged and color-coded according to their respective patterns. **G**. The first two columns show the expression of three LR pairs. The third column displays the centered and scaled slope surfaces. In the fourth column, insignificant spot-level slope estimates are greyed.

non-tumor region possibly composed of keratinized layers and stroma [177]. However, the tumor and non-tumor regions are not clearly delineated, a feature characteristic of moderately differentiated cSCC, though the spatial clusters obtained using the BayesSpace package [52] on the transcriptome-wide gene expression profile (Fig. 2C) partially elucidate this distinction. The constructed MST is shown in Fig. 2B. Using the global test, SpaceBF identified 39 keratin pairs at a significance level of 0.05 (41 at an FDR < 0.1), suggesting that most pairs bind to each other, albeit to varying degrees. Similar to the earlier analysis, we classify the detected slope surfaces into 3 major patterns (Fig. 2D) based on hierarchical clustering of the standardized vector $\boldsymbol{\beta}_1^{mm'*}$. We represent the keratin pairs as bipartite graphs between type 1 and 2 keratins under each pattern (Fig. 2E). One important observation is that type 2 keratins KRT6A, KRT6B, and KRT6C are isoforms of keratin 6 [178] and thus, co-express highly, meaning their binding patterns with any specific type 1 keratin should be similar, as correctly identified by SpaceBF. For instance, the slope surfaces of KRT10 with KRT6A, 6B, and 6C all align with pattern 1, while the slope surfaces of KRT16 with KRT6A, 6B, and 6C all correspond to pattern 2. This consistency underscores the reliability of SpaceBF in identifying true local patterns. In Fig. 2F, we present the estimated slopes for KRT17, which is a well-established therapeutic target in various cancers [179, 180, 181], binding with three type 2 keratins: KRT80 (pattern 1, p-value = 0.006), KRT78 (pattern 2, p-value = 0.03), and KRT6B (pattern 3, p-value = 5.59×10^{-6}). Notably, the average slope estimates for KRT17-KRT80 and KRT17-KRT78 interactions are small (≈ 0.1), whereas for KRT17-KRT6B, the average slope is substantially higher at 0.82, with the highest local estimates observed mostly in tumor regions. These trends are also evident from the individual expression profiles provided in Fig. 2F. Although the expression patterns of KRT80 and KRT78 appear similar, a closer examination reveals that KRT80 exhibits a thicker band of expression on the left, specifically within the tumor regions. This distinction contributes to the difference in co-expression patterns of KRT17-KRT80 and KRT17-KRT78. As previously noted, both association levels are low, also indicated by the small number of significant spots identified by the local test, 72 and 49, respectively.

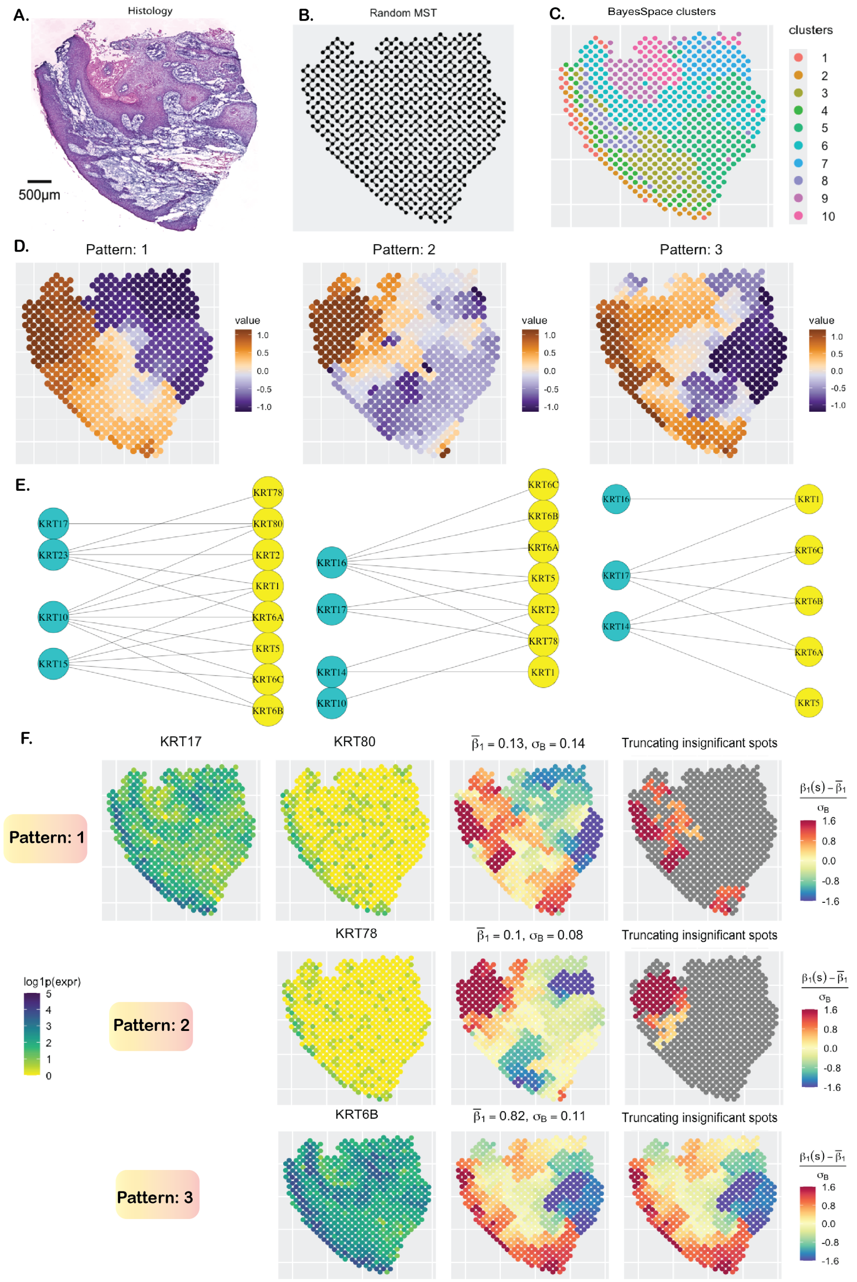

Figure 2: Cutaneous squamous cell carcinoma data analysis. **A**. H&E-stained image. **B**. MST capturing the spatial structure. **C**. Spatial clusters obtained using the BayesSpace package. **D**. The three main spatial patterns of the estimated surfaces. **E**. Bipartite graphs between type 1 and type 2 keratins based on their spatial pattern. **F**. Study of the binding between the type 1 keratin KRT17 and three different type 2 keratins, with each slope surface exhibiting a unique spatial pattern. The insignificant spot-level slope estimates are greyed in the last column.

2.1.3 DCIS proteomics dataset

We analyzed a single-sample ductal carcinoma in situ (DCIS) dataset collected using the MALDI MSI spatial proteomics platform, as part of an ongoing study at the MUSC aimed at defining the proteomic landscape of DCIS and invasive breast cancer (IBC), in terms of collagen peptides and immune cell types. DCIS is marked by the abnormal growth of malignant epithelial cells confined to the breast's milk ducts, without invading the surrounding stromal tissue. While prognosis is excellent, around $20-40\%$ of diagnosed DCIS progress to IBC [182, 183]. Understanding proteomic co-localization within the extracellular matrix (ECM) of a DCIS tissue is crucial for assessing progression risk and predicting therapeutic response, as the ECM plays a key role in regulating tumor cell proliferation, migration, and survival [184]. In this dataset, there are 5,548 tissue spots and 12 ECM peptides, whose $\binom{12}{2}$ pairwise interactions were of our interest. As the peptide expression is continuous-valued, we used the Gaussian model of SpaceBF for this analysis (Eq. 1). Seven of the peptides are derived from the COL1A1 gene, while the remaining peptides originate from COL1A2, COL3A1, and FN1 (Fig. 3C). From the histology image (Fig. 3A), the stromal ECM can be identified by the light pink staining of fibrous connective tissue, while epithelium regions are highlighted in deep blue. Using hierarchical clustering, we group the standardized slope surfaces ($\boldsymbol{\beta}_1^{mm'*}$) into three patterns (Fig. 3B), and the spots into three clusters based on the spot-level vectors of standardized slopes (Fig. 3D). While the differences among the three patterns are subtle, the spot clusters are well-defined and spatially distinct: the red cluster (cluster 3) aligns with stromal regions, the green cluster (cluster 2) with epithelial regions, and the light blue cluster (cluster 1) is a mixture of both. It is important to note that the MSI image has substantially lower resolution compared to the histology image, making one-to-one correspondence between the two inherently challenging. Patterns 1 and 2 (Fig. 3B), which visually resemble each other, both suggest strong co-localization of the associated peptide pairs in the stroma. This is expected, as all of these peptides are known to constitute the stromal ECM. The tree diagram in Fig. 3C shows the hierarchical relationships between the peptide pairs, with their patterns indicated on the right. The module highlighted by the yellow box includes pairs involving peptide 1125 (from COL1A2) and 7 other peptides. From Figs. 3E and 3F (top row), peptides 1125, 1212, 1386, and 1681 (from the module)

show pronounced co-expression in the stromal region. Correspondingly, the estimated slope surfaces (Fig. 3F, bottom row) for the pairs (1125, 1212), (1125, 1386), and (1125, 1681) all fall under pattern 1, but the association strength is notably higher for (1125, 1212): $\overline{\boldsymbol{\beta}_1^{mm'}} = 0.92$, compared to 0.72 and 0.68 for the other two. Although the existing literature on these interactions is limited, the findings will inform future comparative analyses of ECM compositions across DCIS subtypes and stages of progression [185, 186].

2.2 Simulation studies

We consider the spatial coordinates ($n = 293$) from the cutaneous melanoma dataset. In simulation design 1, one NB-distributed random variable (RV), $\mathbf{X}^{m'}$ is generated using a Gaussian copula with a spatial covariance matrix H based on an exponential kernel (for varying lengthscale l) and the L_2 distance. Another NB-distributed RV, $\mathbf{X}^m$ is then generated using the NB model from Eq. 2 with a constant slope $\beta_1^{mm'}(s) = \nu$ and $\boldsymbol{\beta}_0^{mm'}$ simulated using a Gaussian process (GP) model [97] with the spatial covariance matrix H. More details on the design are provided in Section 4.4.1. From Fig. 4A, we notice how the structure of H changes as the lengthscale l varies. The off-diagonal elements of H ($H_{k_1k_2}$) can range between 0 and 1. When $l = 0.6$, only the nearest locations (k_1, k_2) exhibit high $H_{k_1k_2}$ with most of the other values being close to 0. In contrast, for $l = 18$, the majority of location pairs have high $H_{k_1k_2}$ (≈ 1), inducing an exceptionally strong spatial autocorrelation in both variables. To visibly understand how ν might affect the relationship between $\mathbf{X}^m$ and $\mathbf{X}^{m'}$, in Fig. 4B, we show the spatial expression of $\mathbf{X}^m$ for the same $\mathbf{X}^{m'}$ but three different values of ν, $\{-0.75, 0, 0.75\}$. It is somewhat evident that nonzero ν's result in a visibly positive or negative association, while $\nu = 0$ produces a random pattern of $\mathbf{X}^m$. In Fig. 4C, we show the type 1 error ($\nu = 0$) and power ($\nu \neq 0$) comparison of the different methods, including SpaceBF, for three values of the lengthscale l. When $l = 3.6$, both variables exhibit considerable spatial autocorrelation, yet SpaceBF maintains the correct type 1 error. In contrast, all other methods suffer from inflated type 1 errors. Notably, simple Pearson correlation, while still inflated, performs better in controlling type 1 error compared to methods based on bivariate Moran's I or Lee's L. Although SpaGene does not rely on these traditional metrics, it still fails to control type 1 error. The issue becomes more

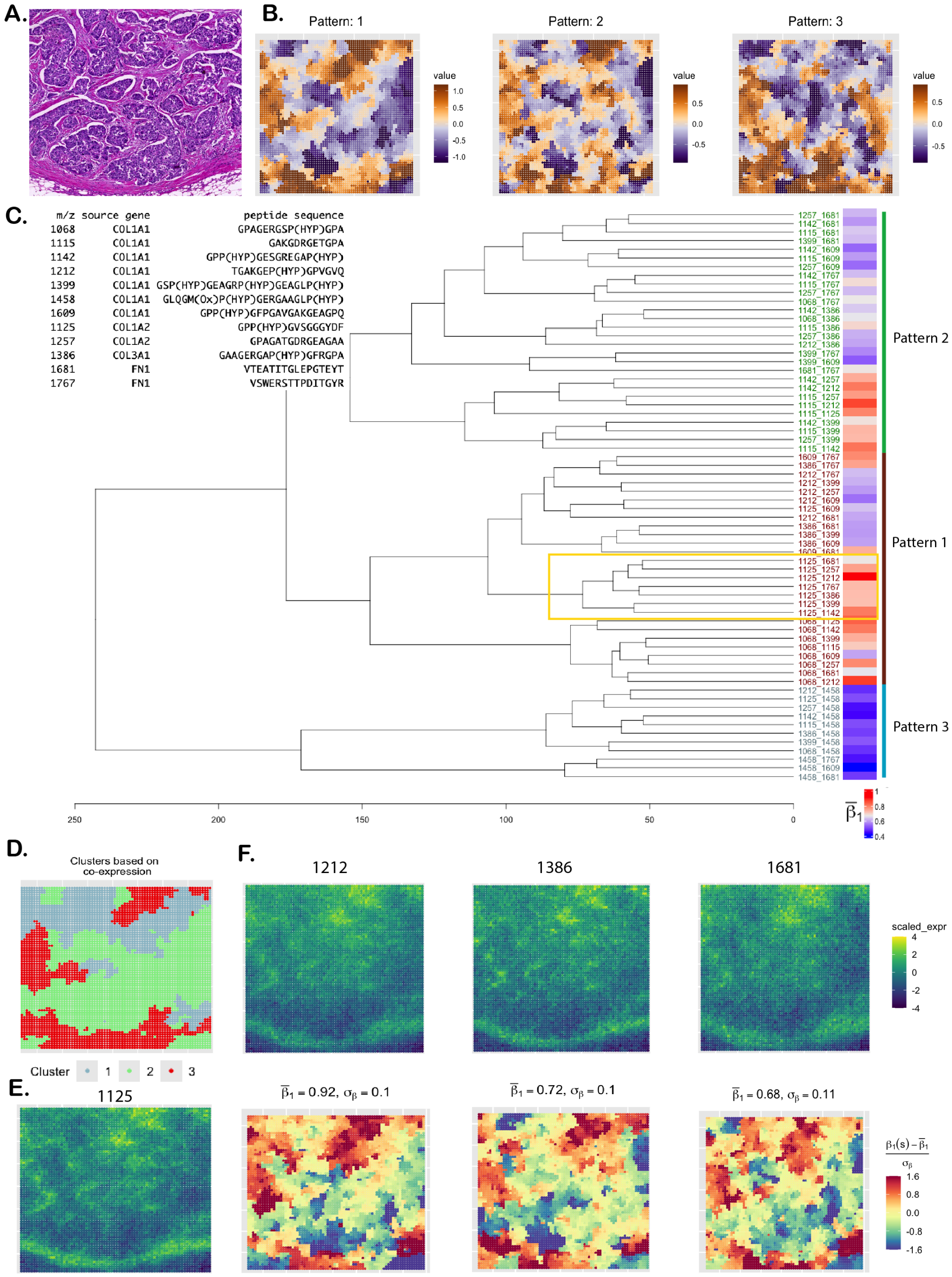

Figure 3: DCIS data analysis. **A**. H&E-stained image. **B**. Patterns of standardized co-expression (slope) of 66 peptide pairs (from 12 peptides). **C**. Peptide description and dendrogram corresponding to the patterns. Mean slope estimates are presented as a heatmap on the right. **D**. Clustering of spots based on the slope surfaces. **E**. Scaled expression of the peptide 1125 forming the yellow-bordered module in the dendrogram. **F**. Scaled expression of three peptides belonging to the same module (top row) and their spatial co-expression with peptide 1125 (bottom row).

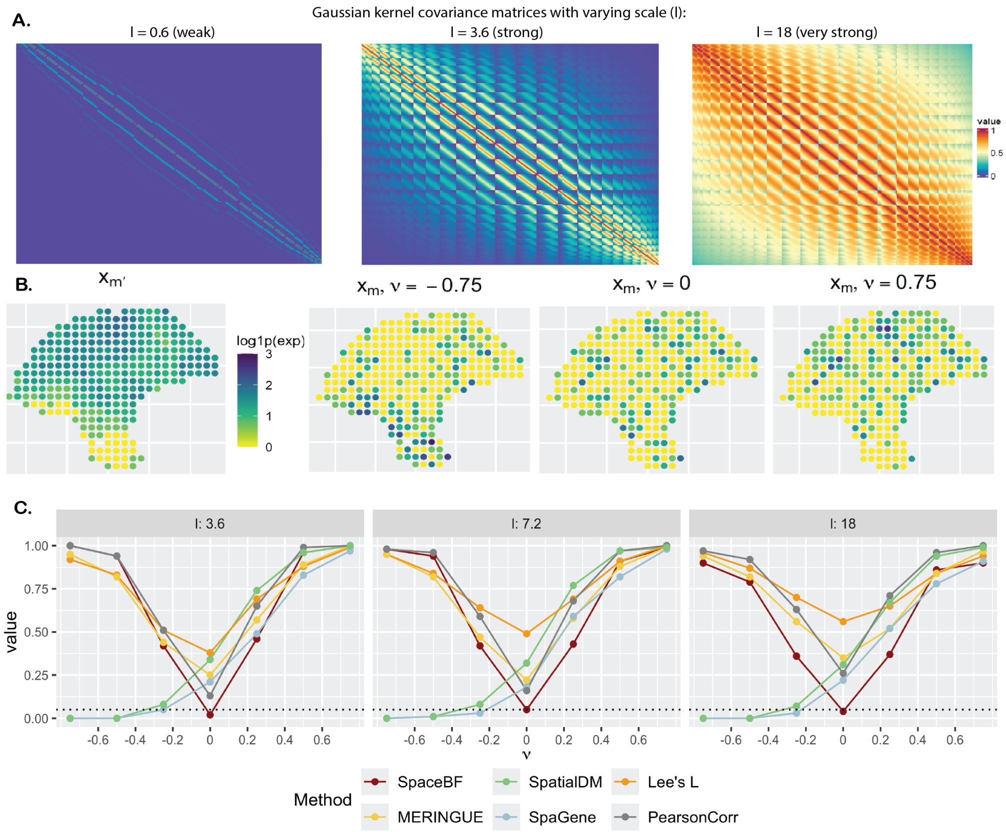

Figure 4: Comparison of global tests under the simulation design 1. **A**. Heatmap of the spatial covariance matrix H with an exponential kernel and L_2 distance, for varying values of the lengthscale parameter l. **B**. Simulated $\mathbf{X}^m$ based on Eq. 2, for a fixed $\mathbf{X}^{m'}$ but different values of the constant slope $\beta_1^{mm'}(s) = \nu$. **C**. Performance of the methods in terms of type 1 error ($\nu = 0$) and power ($\nu \neq 0$). The dotted line represents the significance level 0.05.

pronounced as l increases. Notably, Lee's L exhibits the highest inflation in the majority of cases. SpaceBF also retains a high detection power throughout all three cases. Although the power declines slightly for the largest l, as expected, due to a decrease in effective sample size from increased spatial autocorrelation. In summary, the simulation effectively demonstrates the specificity and power of our method.

In simulation design 2, we generate $(\mathbf{X}^m, \mathbf{X}^{m'})$ jointly as bivariate spatially correlated NB-distributed RVs. This setup is more complex than the previous one, as the association is non-linear and driven by the Kronecker product-based spatial covariance structure (see Section 4.4.2). The methods, except SpaceBF, perform poorly in terms of the type 1 error for $l \geq 1.8$. When the spatial autocorrelation is

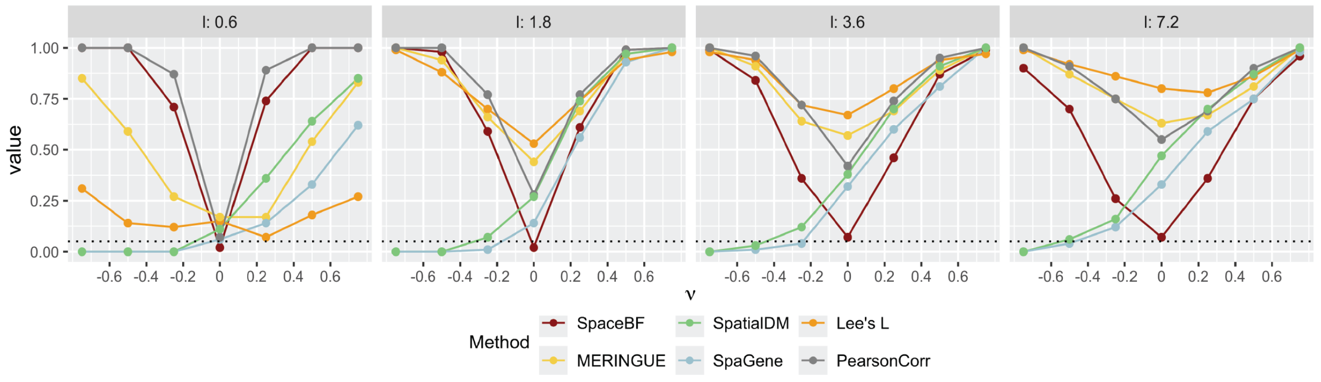

Figure 5: Comparison of global tests under simulation design 2 for lengthscale l between $\{0.6, 1.8, 3.6, 7.2\}$.

the weakest ($l = 0.6$), Pearson correlation performs well as expected, while spatially weighted indices still show a slight inflation. SpaceBF achieves controlled type 1 error and steady detection power across varying l's. As earlier, the power decreases as the effective sample size decreases. Together, these two simulation designs demonstrate SpaceBF's robustness under complex data generation processes. Finally, we argue that incorporating $\beta_0^{mm'}(s)$ in SpaceBF accounts for the spatial autocorrelation of variable m, thereby mitigating bias in the association analysis of (m, m'). As previously noted, Pearson correlation is already recognized to be suboptimal in such scenarios, and spatially weighted indices, essentially Pearson correlation between spatially lagged variables, thus also remain susceptible to spurious detections. Recall that MERINGUE uses a binary spatial weight matrix W based on the Delaunay triangulation, while SpatialDM uses a continuous spatial weight matrix having a similar form as H for a particular choice of the lengthscale. Lee's L is based on a binary $k-$NN network in our study. Hence, the performance of these methods could be sensitive to choices of the spatial weight matrices, i.e., different networks or lengthscale l values. SpatialDM and SpaGene focus solely on the joint over-expression of molecules, neglecting joint under-expression. As a result, for most values of $\nu < 0$, these methods show almost no detection power.

3 Discussion

We have developed a rigorous framework for studying spatial co-expression of a pair of molecules in the context of spatial transcriptomics (ST) and mass spectrometry imaging (MSI) datasets, at both global (tissue-wide) and local (cell/spot-specific) levels. Existing tools mostly rely on two exploratory geospatial metrics, namely, bivariate Moran's I [108] and Lee's L [110], which lead to highly spurious association inference as demonstrated by our simulation studies. Our proposed approach, SpaceBF, builds on the widely used spatially varying coefficients model [136], effectively capturing spatial autocorrelation and locally varying co-expression patterns. We introduce a novel spatial GMRF prior based on the minimum spanning tree (MST) network [187, 160] between locations and a fused horseshoe prior [153, 156], extending a recent frequentist framework from a different domain [157]. We elucidate the prior's theoretical properties and explore its connections to the alternative approaches. Integrating this prior both within a Gaussian linear regression model and a more complex negative binomial regression framework [135], SpaceBF is broadly applicable across various analytical contexts and data types.

We conduct a comprehensive evaluation of the proposed method under challenging simulation scenarios, demonstrating its ability to maintain well-controlled type I error rates alongside strong detection power. In three real-world applications, two spatial transcriptomics (ST) datasets and one mass spectrometry imaging (MSI) dataset, the method exhibits robust performance in identifying biologically meaningful molecular interactions, including ligand-receptor (LR) signaling, keratin binding, and peptide co-localization. Notably, the analysis of the cutaneous melanoma sample reveals spatially variable patterns of cell-cell communication, as assessed through LR interactions. The LR pairs exhibit coordinated over- or under-expression within spatially distinct tissue regions (identified from histology) or specific cell types (inferred from transcriptome-wide gene expression). This level of granular understanding may provide critical insights for developing novel, targeted tissue-specific therapies in broader clinical settings [188, 189, 190].

Utilizing the MST as the underlying spatial graph provides several advantages: 1) its uniqueness, eliminating the need to tune additional hyperparameters such as the lengthscale parameter in Gaussian process models [97], and 2) reduced computational complexity by inducing a sparser precision matrix. In

all of our simulation studies, where spatial dependency is consistently generated using a Gaussian process with an exponential kernel and varying lengthscales, the MST-based approach performs exceptionally well, underscoring its robustness. Nevertheless, fixing the spatial structure to a spanning tree may not always be optimal, as it could exclude important edges [191]. Future work will consider treating the spanning tree as an unknown parameter to be iteratively updated [192]. While covariate adjustment is readily accommodated in our framework, further exploration is warranted to better understand cell-type-specific co-expression patterns across diverse datasets. In addition, extending the models to handle multiple spatially varying predictors presents a promising avenue, which we aim to pursue with particular attention to computational scalability and multicollinearity. Although SpaceBF has only been used in the ST and MSI datasets, it could also be useful in multiplex immunofluorescence (mIF) or imaging mass cytometry datasets where the molecular outcome of interest is generally immune cell types. To study cell type co-localization in such cases, one could split an mIF image into regular grids and count how many cells of two types (m, m') fall into each grid. Assuming that the grid centers are the locations s_k's, an MST can be constructed between them, and the spatial cell counts $X^m(s_k)$ and $X^{m'}(s_k)$ can be analyzed as before.

4 Methods

4.1 Gaussian and negative binomial models

We assume a single sample or image with n spots/cells. Let $X^m(s_k)$ and $X^{m'}(s_k)$ denote the expression of a pair of molecules m and m', and $C(s_k)$ be a vector of p covariates observed at spot/cell location s_k, for $k \in \{1, \ldots, n\}$. For example, $C(s_k)$ can be the cell type indicator or a vector of cell type proportions [162]. We consider the following Gaussian spatially varying coefficients (SVC) model [136]

$$X^m(s_k) = \beta_0^{mm'}(s_k) + X^{m'}(s_k)\beta_1^{mm'}(s_k) + C(s_k)^T\alpha_m + \epsilon(s_k), \;\; k = 1, \ldots, n, \tag{1}$$

where $\beta_0^{mm'}(s_k)$ and $\beta_1^{mm'}(s_k)$ denote spatially varying intercept and slope, respectively, α_m is a fixed effect vector, and $\epsilon(s_k)$ is an independent error term. To interpret the model, a significantly positive $\beta_1^{mm'}(s_k)$ implies that the molecules (m, m') co-express at the location s_k, while a significantly negative

value suggests avoidance. Intuitively, $\beta_0^{mm'}(s_k)$ accounts for spatial autocorrelation of molecule m. More discussion on the underlying bivariate spatial process is provided in the Supplementary Material. For a count-valued $X^m(s_k)$ (e.g., genes in the ST datasets), we consider a spatially varying negative binomial (NB) distribution [135] as $NB(\psi_m(s_k), r_m)$ with the failure probability $\psi_m(s_k)$ modeled following Pillow and Scott (2012) [193],

$$\begin{aligned} &\eta_m(s_k) = \beta_0^{mm'}(s_k) + X^{m'}(s_k)\beta_1^{mm'}(s_k) + C(s_k)^T\alpha_m \\ &p(X^m(s_k)|\psi_m(s_k), r_m) \propto (1-\psi_m(s_k))^{r_m}\psi_m(s_k)^{X^m(s_k)},\ \ \psi_m(s_k) = \frac{\exp(\eta_m(s_k))}{1+\exp(\eta_m(s_k))}, \end{aligned} \tag{2}$$

where $p(.|.)$ denotes the conditional probability mass function (PMF) and the dispersion parameter $r_m(> 0)$ is assumed to be constant across locations. To explain how this framework effectively models overdispersion: as $r_m \to \infty$, it reduces to a Poisson model; in contrast, as $r_m \to 0$, the counts become increasingly dispersed relative to the Poisson distribution [135]. Admittedly, this model is limited as the count-valued nature of the molecule $X^{m'}(s_k)$ is not prioritized, appearing as a spatially varying predictor. Jointly modeling $(X^m(s_k), X^{m'}(s_k))$ as bivariate NB (BNB) random variables is a possible approach that we do not pursue, as the existing definitions of the BNB distribution (outside of Copula-based constructions) [194, 195, 196, 197] vary considerably, often leading to restrictive correlation structures and inefficient MCMC sampling.

The models in Eqs. 1 and 2 are over-parametrized and do not incorporate spatial dependency between $\beta_0^{mm'}(s_k)$'s and $\beta_1^{mm'}(s_k)$'s, which we discuss next. Let $G = (V, E)$ denote the MST network between the locations constructed using the L_2 distance: $|s_{k_1} - s_{k_2}|^2$ for a pair (s_{k_1}, s_{k_2}), where V and E are the sets of vertices and edges, respectively. Given a connected, weighted graph, an MST is an acyclic subgraph that connects all vertices and minimizes the sum of the weights of the included edges. Because of this property, MST is routinely used to develop transportation and telecommunication networks [198]. For a regular grid, MST is not unique, as the inter-point distances are not distinct. However, a unique random MST can be curated by simply adding small random values to the distances [187, 160].

4.2 Spatial modeling

4.2.1 Spatial fused lasso

In a recent study [157] of the temperature-salinity relationship in the Atlantic Ocean, Li et al. (2019) consider Eq. 1 and elegantly promote spatial homogeneity of the coefficients by considering fused lasso penalties [151, 199]: $|\beta_0^{mm'}(s_{k_1}) - \beta_0^{mm'}(s_{k_2})| \approx 0$ and $|\beta_1^{mm'}(s_{k_1}) - \beta_1^{mm'}(s_{k_2})| \approx 0$ for $(s_{k_1}, s_{k_2}) \in E$, in a frequentist setup. These constraints are intuitive, as it is reasonable to expect both the degree of co-expression, $\beta_1^{mm'}(s)$, and the effect of "unmeasured" factors, $\beta_0^{mm'}(s)$, to remain homogeneous across adjacent or connected locations. Extending this idea, a Bayesian fused lasso [200, 153] approach can be considered with Laplacian priors on the pair-wise differences of the coefficients as

$$\begin{aligned}
\pi(\boldsymbol{\beta}_0^{mm'}|\ldots) &\propto \prod_{(s_{k_1}, s_{k_2}) \in E} \exp\left(-\frac{\lambda_0}{\sigma}|\beta_0^{mm'}(s_{k_1}) - \beta_0^{mm'}(s_{k_2})|\right), \ \boldsymbol{\beta}_0^{mm'} = (\beta_0^{mm'}(s_1), \ldots, \beta_0^{mm'}(s_n))^T, \\
\pi(\boldsymbol{\beta}_1^{mm'}|\ldots) &\propto \prod_{(s_{k_1}, s_{k_2}) \in E} \exp\left(-\frac{\lambda_1}{\sigma}|\beta_1^{mm'}(s_{k_1}) - \beta_1^{mm'}(s_{k_2})|\right), \ \boldsymbol{\beta}_1^{mm'} = (\beta_1^{mm'}(s_1), \ldots, \beta_1^{mm'}(s_n))^T,
\end{aligned} \tag{3}$$

where σ^2 is the variance of the error term $\epsilon(s_k)$, λ_0 and λ_1 are regularization parameters that control the strength of fusion and are assumed to follow gamma priors. Note that σ^2 is only present in the Gaussian model (Eq. 1) and could be omitted from the above exponents for simplicity. We discuss the resemblance of the prior to the intrinsic CAR (ICAR) prior [146] and, more generally, the intrinsic GMRF (IGMRF) prior [150] in the Supplementary Material. Theoretically, using the L_1 distance seems appealing, as it has the potential to achieve better spatial smoothing by "exactly" fusing coefficient values at adjacent locations, unlike the L_2 distance implied by the ICAR prior. This is analogous to how lasso regression enforces sparsity in solutions, while ridge regression only shrinks effect sizes toward 0 [201]. For transparency, such a spatial fused lasso prior has already been proposed in the existing literature [192, 202].

4.2.2 Spatial fused horseshoe

In variable selection problems, failure of the Bayesian lasso or Laplacian prior to achieve exact sparsity, unlike the frequentist analog, has been reported, while also underestimating larger effect sizes [203, 204, 205].

Consequently, the Bayesian fused lasso might struggle to promote spatial smoothness and preserve distinct local features simultaneously. For variable selection, the advantages of the horseshoe prior have been convincingly demonstrated to handle unknown sparsity and large outlying signals [154, 206, 207]. The horseshoe prior belongs to the class of global-local shrinkage priors [208], characterized by a "global" hyperparameter that controls overall shrinkage, while "local" hyperparameters control shrinkage per coefficient. Following recent developments on Bayesian fused horseshoe approach [156, 209], we assume that

$$\begin{aligned}\beta_0^{mm'}(s_{k_i^1}) - \beta_0^{mm'}(s_{k_i^2})|\Lambda_{0i}^2, \tau_0^2, \sigma^2 &\sim N(0, \Lambda_{0i}^2\tau_0^2\sigma^2),\ \ \Lambda_{0i} \sim C^+(0,1),\ \ \tau_0 \sim C^+(0,1)\\ \beta_1^{mm'}(s_{k_i^1}) - \beta_1^{mm'}(s_{k_i^2})|\Lambda_{1i}^2, \tau_1^2, \sigma^2 &\sim N(0, \Lambda_{1i}^2\tau_1^2\sigma^2),\ \ \Lambda_{1i} \sim C^+(0,1),\ \ \tau_1 \sim C^+(0,1)\end{aligned} \tag{4}$$

independently, where $(s_{k_i^1}, s_{k_i^2})$ are the nodes or locations associated with the i-th edge from E. τ_0 and τ_1 are the global hyper-parameters, Λ_{0i}'s and Λ_{1i}'s are the local hyperparameters, and C^+ stands for the half-Cauchy distribution. The resulting conditional prior PDFs of $(\boldsymbol{\beta}_0^{mm'}, \boldsymbol{\beta}_1^{mm'})$ are

$$\begin{aligned}\pi(\boldsymbol{\beta}_0^{mm'}|.) &\propto \prod_{i=1}^{p} \frac{1}{\Lambda_{0i}\tau_0\sigma} \exp\left[-\frac{(\beta_0^{mm'}(s_{k_i^1}) - \beta_0^{mm'}(s_{k_i^2}))^2}{2\Lambda_{0i}^2\tau_0^2\sigma^2}\right]\\ \pi(\boldsymbol{\beta}_1^{mm'}|.) &\propto \prod_{i=1}^{p} \frac{1}{\Lambda_{1i}\tau_1\sigma} \exp\left[-\frac{(\beta_1^{mm'}(s_{k_i^1}) - \beta_1^{mm'}(s_{k_i^2}))^2}{2\Lambda_{1i}^2\tau_1^2\sigma^2}\right]\end{aligned} \tag{5}$$

The spatial fused horseshoe prior, with its half-Cauchy priors on both global and local hyperparameters, has heavy tails that allow substantial differences between neighboring locations to escape excessive shrinkage, unlike the Laplacian prior. At the same time, its infinitely tall spike at zero strongly shrinks small differences toward zero. This dual behavior enables the prior to preserve spatial homogeneity while still accommodating sharp local variations. The relation of such a locally adaptive fusion prior to the general class of GMRF priors was first highlighted by Faulkner and Mining (2017) [210] in a longitudinal modeling context, encouraging fusion between coefficients across time points. In the Supplementary Material, we provide a discussion on this relation and details of the Gibbs sampling steps, which include the Pólya-Gamma data augmentation strategy [193, 211] for the NB model (Eq. 2).

One critical aspect that deserves elucidation is the assumption of independence between edge-wise differences. Specifically, for any two edges i, i', $\beta_1^{mm'}(s_{k_i^1}) - \beta_1^{mm'}(s_{k_i^2})$ and $\beta_1^{mm'}(s_{k_{i'}^1}) - \beta_1^{mm'}(s_{k_{i'}^2})$ are assumed to be independent in Eq. 4, conditional on the hyperparameters. To see how this assumption

could be problematic for a general graph (not the MST), we briefly highlight one example from Rue and Held (2005) [150] provided in the context of IGMRF priors. Suppose there are only three locations A, B, and C, all neighbors of each other. Letting $e_1 = \beta_1^{mm'}(A) - \beta_1^{mm'}(B)$, $e_2 = \beta_1^{mm'}(B) - \beta_1^{mm'}(C)$, and $e_3 = \beta_1^{mm'}(C) - \beta_1^{mm'}(A)$, Eq. 4 proceeds to assume e_1, e_2, e_3 are independent and normally distributed with non-identical parameters. However, we notice that there is a "hidden" linear constraint, $e_1+e_2+e_3 = 0$, that directly contradicts the model assumption of independence. Analogously, using a highly connected or dense spatial neighborhood graph G introduces numerous hidden constraints corresponding to the cycles in G. Interestingly enough, as demonstrated in Theorem 1 of the Supplementary Material, the hidden constraints do not require to be explicitly accounted for since the posterior sampling distribution of $\boldsymbol{\beta}_0$ and $\boldsymbol{\beta}_1$ (based on the priors in Eq. 5) remains unaffected. However, penalizing too many edge-wise differences and the implicit dependency might lead to over-smoothing and loss of local structures. Thus, the use of MST becomes crucial, as it is acyclic (no hidden constraints) and eliminates redundant relationships. Additionally, a sparser G enables faster Cholesky decomposition of the precision matrix, enhancing computational efficiency.

4.3 Hypothesis testing

We consider two types of hypothesis tests: 1) global test: to determine the significance of average association across the entire tissue domain $\left(H_0 : \overline{\beta_1^{mm'}} = \frac{1}{n}\sum_{k=1}^{n} \beta_1^{mm'}(s_k) = 0\right)$, based on the credible interval [212] of $\overline{\beta_1^{mm'}}$, and 2) local test: to determine the significance of location-level association $\left(H_0^k : \beta_1^{mm'}(s_k) = 0\right)$, directly based on the credible intervals of $\beta_1^{mm'}(s_k)$'s. Additionally, in the genomic context, having a measure analogous to the frequentist p-value is often beneficial. To this end, we utilize a metric termed the probability of direction (p_d), which quantifies the probability (between 0.5 and 1) that a parameter has an effect in a specific direction, either positive or negative [213, 214]. Mathematically, it is defined as the proportion of the posterior distribution that shares the same sign as the median. p_d resembles a two-sided frequentist p-value as $p_{\text{two-sided}} = 2(1 - p_d)$. It is implemented in the R package `bayestestR` [213].

4.4 Simulation design

We consider two different simulation designs as outlined below, for assessing the type 1 error and power of the model proposed in Eq. 2. The locations at which the variables are simulated are the same as the previously discussed cutaneous melanoma dataset ($n = 293$). We have observed that the results remain unaffected when a randomly generated set of locations or other real data-based sets of locations are used.

4.4.1 Simulation design 1

In the first design, we directly consider the model from Eq. 2 to generate $(\mathbf{X}^m, \mathbf{X}^{m'})$ based on two steps. First, we generate an NB-distributed RV, $\mathbf{X}^{m'}$, using Gaussian copula [127], incorporating spatial dependency between the observations via a kernel covariance matrix H with an exponential kernel on the L_2 distances and varying lengthscale (l) parameters [114]. Then, based on the simulated $\mathbf{X}^{m'}$, we generate $\mathbf{X}^m$ following Eq. 2 with a fixed slope $\beta_1^{mm'}(s_k) = \nu$. More specifically, for a fixed choice of l, failure probability $\psi_{m'}$, dispersion parameter $r_{m'}$ for variable m', and dispersion parameter r_m for variable m, we consider the following steps

1. Simulate a spatially autocorrelated normal RV of size n using a Gaussian process (GP) model:

$$\mathbf{Z}^{m'} \sim MVN\left(\mathbf{0}, H\right), \; H_{k_1k_2} = \exp\left(-\frac{|s_{k_1} - s_{k_2}|}{l}\right),$$

2. Transform to a vector of uniform RVs using the standard normal CDF (Φ):

$$\mathbf{U}^{m'} = \Phi(\mathbf{Z}^{m'}),$$

3. Convert to a vector of NB RVs using the inverse CDF of $NB(\psi_{m'}, r_{m'})$, denoted by $F^{-1}_{NB(\psi_{m'},r_{m'})}$:

$$\mathbf{X}^{m'} = F^{-1}_{NB(\psi_{m'},r_{m'})}(\mathbf{U}^{m'}),$$

   Each element of the resulting vector $\mathbf{X}^{m'}$ retains the marginal NB distribution, $NB(\psi_{m'}, r_{m'})$, where $\psi_{m'}$ is the failure probability and $r_{m'}$ is the dispersion.

4. Generate the link function to simulate variable m with a fixed slope of $\beta_1^{mm'}(s_k) = \nu$ (2):

$$\boldsymbol{\eta}_m = \boldsymbol{\beta}_0^{mm'} + \nu \log(\mathbf{X}^{m'} + 1), \quad \boldsymbol{\beta}_0^{mm'} \sim MVN\left(\mathbf{0}, 0.5H\right),$$

5. Convert the link vector to failure probabilities $\boldsymbol{\psi}_m$ and simulate $\mathbf{X}^m$ from the NB distribution:

$$\boldsymbol{\psi}_m = \frac{\exp(\boldsymbol{\eta}_m)}{1 + \exp(\boldsymbol{\eta}_m)}, \quad \mathbf{X}^m \sim NB(\boldsymbol{\psi}_m, r_m)$$

   where r_m is a prefixed dispersion parameter. The k-th element of $\mathbf{X}^m$ follows $NB(\psi_{mk}, r_m)$, where $\boldsymbol{\psi}_m = (\psi_{m1}, \ldots, \psi_{mn})^T$ and $\boldsymbol{\eta}_m = (\eta_{m1}, \ldots, \eta_{mn})^T$.

Three values of the lengthscale l are considered, $l = 3.6, 7.2, 18$, with the corresponding structure of H displayed in Fig. 4A. The failure probability of variable m' and dispersion parameters are kept fixed, $\psi_{m'} = 0.5$, $r_m = r_{m'} = 1$. The slope parameter ν is varied between $\{-0.75, -0.5, -0.25, 0, 0.25, 0.5, 0.75\}$, with negative and positive values representing negative and positive association, respectively. Higher absolute value of ν dictates the strength of association, and $\nu = 0$ corresponds to the null model, i.e., $\mathbf{X}^m$ and $\mathbf{X}^{m'}$ are independent.

4.4.2 Simulation design 2

In this design, $(\mathbf{X}^m, \mathbf{X}^{m'})$ are simulated jointly using a bivariate Gaussian copula and spatial dependency incorporated using a bivariate GP framework, where the joint covariance matrix has a Kronecker product structure, comprising a 2×2 correlation matrix and the distance kernel covariance matrix H (Eq. 9.11 from Banerjee et al. (2014)[97] and Eq. 6 from the Supplementary Material). Specifically, we consider the following steps

1. Simulate spatially cross-correlated normal RVs:

$$(\mathbf{Z}^m, \mathbf{Z}^{m'})^T \sim MVN\left(\begin{bmatrix}\mathbf{0}\\ \mathbf{0}\end{bmatrix}, \boldsymbol{\Sigma} = \begin{bmatrix}1 & \nu\\ \nu & 1\end{bmatrix} \otimes H\right), \quad H_{k_1k_2} = \exp\left(-\frac{|s_{k_1} - s_{k_2}|}{l}\right),$$

2. Transform to uniform RVs using the standard normal CDF (Φ):

$$\mathbf{U}^m = \Phi(\mathbf{Z}^m), \quad \mathbf{U}^{m'} = \Phi(\mathbf{Z}^{m'}),$$

3. Convert to NB random variables using the inverse CDFs:

$$\mathbf{X}^m = F^{-1}_{NB(\psi_m, r_m)}(\mathbf{U}^m), \quad \mathbf{X}^{m'} = F^{-1}_{NB(\psi_{m'}, r_{m'})}(\mathbf{U}^{m'}),$$

Each element of the resulting vectors $\mathbf{X}^m$ and $\mathbf{X}^{m'}$ retain the marginal NB distributions, $NB(\psi_m, r_m)$ and $NB(\psi_{m'}, r_{m'})$, respectively, where $\psi_m, \psi_{m'}$ are failure probabilities and $r_m, r_{m'}$ are dispersions.

The lengthscale l is varied between $\{0.6, 1.8, 3.6, 7.2\}$. The failure probabilities and dispersion parameters are kept fixed, $\psi_m = \psi_{m'} = 0.5$, $r_m = r_{m'} = 1$. The parameter ν is varied between $\{-0.75, -0.5, -0.25, 0, 0.25, 0.5, 0.75\}$, with similar implications on the direction and strength of association as before.

4.5 Competing methods

We compare SpaceBF to five different methods: 1) MERINGUE [98] with the Delaunay triangulation network, 2) SpatialDM [102] with the kernel weight matrix (lengthscale 1.2 as used in the original paper for the melanoma dataset), 3) Lee's L [110] with k-NN network (implemented in R package spdep [215], $k = 1$), 4) SpaGene [100], and 5) PearsonCorr, which is the simple Pearson correlation. We do not use the original packages of LIANA+ [104] and Voyager [117], as the former essentially implements SpatialDM and the latter is a direct application of Lee's L. Table 1 summarizes the methods in terms of their assumptions and limitations. We also attempted to evaluate the performance of SpatialCorr [176] and Copulacci [105]. SpatialCorr was straightforward to use, but it proved highly sensitive to the choice of the lengthscale l in its innovative use of the spatial covariance matrix H. Copulacci was slightly difficult to use and will be benchmarked in a future study.

5 Data and software availability

The melanoma and cSCC datasets are publicly available at the links: 1) cutaneous melanoma [39]: https://zenodo.org/records/8215682 with sample ID "`ST_mel1_rep2`", and 2) cSCC [161]: https://www.ncbi.nlm.nih.gov/geo/query/acc.cgi?acc=GSE144240 with sample ID

Method	Central metric or concept	Global and local co-expression tests	Test type	Potential sensitivity
**SpaceBF**	Spatially varying coefficients model with NB distribution	Global and local	Exact	MST-based spatial network
MERINGUE	Bivariate Moran's I	Global	Permutation test	Neighborhood network
SpatialDM	Bivariate Moran's I	Global and local	Permutation or exact test	Lengthscale in the kernel weight matrix
LIANA+	Bivariate Moran's I and cosine similarity	Global and local	Permutation test	As above
Voyager	Lee's L	Global and local	Permutation test	Neighborhood network
SpaGene	k-NN network and earth mover's distance	Global but with local interaction scores	Permutation test	Choice of k
PearsonCorr	Pearson correlation	Global	Permutation or	None
SpatialCorr *	Spatial kernel-weighted sample correlation	Global and local	Permutation test	Lengthscale in the kernel weight matrix
Copulacci *	Bivariate Poisson distribution along a neighborhood network	Global but with local interaction scores	Permutation test	Neighborhood network and fixed correlation term across locations

Table 1: Comparison of the methods in terms of the underlying assumptions. *methods that are not evaluated in the simulations.

"`GSM4284236 P6_cSCC_scRNA`". The spatial proteomics dataset is available on Zenodo at https://zenodo.org/records/15866928. A GitHub *R* package named `SpaceBF`, with the proposed method and the first two datasets in ".rda" format, is available at https://github.com/sealx017/SpaceBF/.

6 Funding

S.S. and B.N. were supported in part by the Biostatistics Shared Resource, Hollings Cancer Center, Medical University of South Carolina (P30 CA138313). S.S. was supported in part by NIH R21 CA286287-01A1. The content is solely the responsibility of the authors and does not necessarily represent the official views of the National Cancer Institute and the National Institutes of Health.

7 Acknowledgments

The authors are grateful to Dr. Peggi Angel from the Medical University of South Carolina for her help in the spatial proteomics dataset acquisition and interpretation. S.S. and B.N. contributed equally to the conceptualization and methodology of the project, and jointly wrote the first draft. S.S. conducted the validation, simulation experiments, and software development. The authors do not have any competing interests.

References

[1] J. R. Moffitt, E. Lundberg, and H. Heyn. The emerging landscape of spatial profiling technologies. *Nature Reviews Genetics*, 23(12):741–759, 2022.

[2] D. Bressan, G. Battistoni, and G. J. Hannon. The dawn of spatial omics. *Science*, 381(6657):eabq4964, 2023. PMCID: PMC7614974.

[3] K. Vandereyken, A. Sifrim, B. Thienpont, and T. Voet. Methods and applications for single-cell and spatial multi-omics. *Nature Reviews Genetics*, pages 1–22, 2023. PMCID: PMC9979144.

[4] P. L. Ståhl, F. Salmén, S. Vickovic, A. Lundmark, J. F. Navarro, J. Magnusson, S. Giacomello, M. Asp, J. O. Westholm, M. Huss, et al. Visualization and analysis of gene expression in tissue sections by spatial transcriptomics. *Science*, 353(6294):78–82, 2016.

[5] S. Shah, E. Lubeck, W. Zhou, and L. Cai. seqFISH accurately detects transcripts in single cells and reveals robust spatial organization in the hippocampus. *Neuron*, 94(4):752–758, 2017.

[6] M. Asp, J. Bergenstråhle, and J. Lundeberg. Spatially resolved transcriptomes—next generation tools for tissue exploration. *BioEssays*, 42(10):1900221, 2020.

[7] L. Moses and L. Pachter. Museum of spatial transcriptomics. *Nature Methods*, 19(5):534–546, 2022.

[8] P. M. Angel, A. Mehta, K. Norris-Caneda, and R. R. Drake. MALDI imaging mass spectrometry of N-glycans and tryptic peptides from the same formalin-fixed, paraffin-embedded tissue section. *Tissue proteomics: methods and protocols*, pages 225–241, 2018.

[9] J. M. Spraggins, K. V. Djambazova, E. S. Rivera, L. G. Migas, E. K. Neumann, A. Fuetterer, J. Suetering, N. Goedecke, A. Ly, R. Van de Plas, et al. High-performance molecular imaging with MALDI trapped ion-mobility time-of-flight (timsTOF) mass spectrometry. *Analytical chemistry*, 91(22):14552–14560, 2019.

[10] T. R. Hawkinson, H. A. Clarke, L. E. Young, L. R. Conroy, K. H. Markussen, K. M. Kerch, L. A. Johnson, P. T. Nelson, C. Wang, D. B. Allison, et al. In situ spatial glycomic imaging of mouse and human Alzheimer's disease brains. *Alzheimer's & Dementia*, 18(10):1721–1735, 2022.

[11] X. Ma and F. M. Fernández. Advances in mass spectrometry imaging for spatial cancer metabolomics. *Mass spectrometry reviews*, 43(2):235–268, 2024.

[12] K. Heinzmann, L. M. Carter, J. S. Lewis, and E. O. Aboagye. Multiplexed imaging for diagnosis and therapy. *Nature Biomedical Engineering*, 1(9):697–713, 2017.

[13] E. A. Burlingame, J. Eng, G. Thibault, K. Chin, J. W. Gray, and Y. H. Chang. Toward reproducible, scalable, and robust data analysis across multiplex tissue imaging platforms. *Cell reports methods*, 1(4):100053, 2021. PMCID: PMC8415641.

[14] S. M. Lewis, M.-L. Asselin-Labat, Q. Nguyen, J. Berthelet, X. Tan, V. C. Wimmer, D. Merino, K. L. Rogers, and S. H. Naik. Spatial omics and multiplexed imaging to explore cancer biology. *Nature methods*, 18(9):997–1012, 2021.

[15] C. C. Liu et al. Multiplexed ion beam imaging: insights into pathobiology. *Annual Review of Pathology: Mechanisms of Disease*, 17:403–423, 2022.

[16] Visium Spatial Gene Expression, 10X Genomics. `https://www.10xgenomics.com/products/spatial-gene-expression`. Accessed: 2024-10-25.

[17] MALDI Imaging - Reveal Greater Molecular Insight, Bruker. `https://www.bruker.com/en/applications/academia-life-science/imaging/maldi-imaging.html`. Accessed: 2024-10-25.

[18] The PhenoCycler-Fusion 2.0 Solution, Akoya Biosciences. `https://www.akoyabio.com/phenocycler/`. Accessed: 2024-10-25.

[19] A. Heindl, S. Nawaz, and Y. Yuan. Mapping spatial heterogeneity in the tumor microenvironment: a new era for digital pathology. *Laboratory investigation*, 95(4):377–384, 2015.

[20] Y. Yuan. Spatial heterogeneity in the tumor microenvironment. *Cold Spring Harbor perspectives in medicine*, 6(8):a026583, 2016. PMCID: PMC4968167.

[21] D. Lähnemann, J. Köster, E. Szczurek, D. J. McCarthy, S. C. Hicks, M. D. Robinson, C. A. Vallejos, K. R. Campbell, N. Beerenwinkel, A. Mahfouz, et al. Eleven grand challenges in single-cell data science. *Genome biology*, 21:1–35, 2020. PMCID: PMC7007675.

[22] R. Ahmed, R. Augustine, E. Valera, A. Ganguli, N. Mesaeli, I. S. Ahmad, R. Bashir, and A. Hasan. Spatial mapping of cancer tissues by OMICS technologies. *Biochimica et Biophysica Acta (BBA)-Reviews on Cancer*, 1877(1):188663, 2022. PMCID: PMC10283077.

[23] S. Nagasawa, J. Zenkoh, Y. Suzuki, and A. Suzuki. Spatial omics technologies for understanding molecular status associated with cancer progression. *Cancer Science*, 115(10):3208–3217, 2024.

[24] D. Edsgärd, P. Johnsson, and R. Sandberg. Identification of spatial expression trends in single-cell gene expression data. *Nature methods*, 15(5):339–342, 2018. PMCID: PMC6314435.

[25] V. Svensson, S. A. Teichmann, and O. Stegle. SpatialDE: identification of spatially variable genes. *Nature methods*, 15(5):343–346, 2018. PMCID: PMC6350895.

[26] S. Sun, J. Zhu, and X. Zhou. Statistical analysis of spatial expression patterns for spatially resolved transcriptomic studies. *Nature methods*, 17(2):193–200, 2020. PMCID: PMC7233129.

[27] J. Zhu, S. Sun, and X. Zhou. SPARK-X: non-parametric modeling enables scalable and robust detection of spatial expression patterns for large spatial transcriptomic studies. *Genome Biology*, 22(1):1–25, 2021. PMCID: PMC8218388.

[28] Q. Li, M. Zhang, Y. Xie, and G. Xiao. Bayesian modeling of spatial molecular profiling data via Gaussian process. *Bioinformatics*, 37(22):4129–4136, 2021. PMCID: PMC9502169.

[29] Y. Hao, S. Hao, E. Andersen-Nissen, W. M. Mauck, S. Zheng, A. Butler, M. J. Lee, A. J. Wilk, C. Darby, M. Zager, et al. Integrated analysis of multimodal single-cell data. *Cell*, 184(13):3573–3587, 2021. PMCID: PMC8238499.

[30] X. Jiang, G. Xiao, and Q. Li. A Bayesian modified Ising model for identifying spatially variable genes from spatial transcriptomics data. *Statistics in Medicine*, 41(23):4647–4665, 2022.

[31] K. Zhang, W. Feng, and P. Wang. Identification of spatially variable genes with graph cuts. *Nature Communications*, 13(1):5488, 2022. PMCID: PMC9485129.

[32] L. M. Weber, A. Saha, A. Datta, K. D. Hansen, and S. C. Hicks. nnSVG for the scalable identification of spatially variable genes using nearest-neighbor Gaussian processes. *Nature Communications*, 14(1):4059, 2023. PMCID: PMC10333391.

[33] S. Seal, B. G. Bitler, and D. Ghosh. SMASH: Scalable Method for Analyzing Spatial Heterogeneity of genes in spatial transcriptomics data. *PLoS Genetics*, 19(10):e1010983, 2023. PMCID: PMC10619839.

[34] R. Jiang, Z. Li, Y. Jia, S. Li, and S. Chen. SINFONIA: scalable identification of spatially variable genes for deciphering spatial domains. *Cells*, 12(4):604, 2023. PMCID: PMC9954745.

[35] S. D. Adhikari, J. Yang, J. Wang, and Y. Cui. Recent advances in spatially variable gene detection in spatial transcriptomics. *Computational and Structural Biotechnology Journal*, 2024. PMCID: PMC10869304.

[36] P. Cai, M. D. Robinson, and S. Tiberi. DESpace: spatially variable gene detection via differential expression testing of spatial clusters. *Bioinformatics*, 40(2):btae027, 2024. PMCID: PMC10868334.

[37] C. Chen, H. J. Kim, and P. Yang. Evaluating spatially variable gene detection methods for spatial transcriptomics data. *Genome Biology*, 25(1):18, 2024. PMCID: PMC10789051.

[38] G. Yan, S. H. Hua, and J. J. Li. Categorization of 34 computational methods to detect spatially variable genes from spatially resolved transcriptomics data. *Nature Communications*, 16(1):1141, 2025.

[39] K. Thrane, H. Eriksson, J. Maaskola, J. Hansson, and J. Lundeberg. Spatially resolved transcriptomics enables dissection of genetic heterogeneity in stage III cutaneous malignant melanoma. *Cancer research*, 78(20):5970–5979, 2018.

[40] J. F. Navarro, D. L. Croteau, A. Jurek, Z. Andrusivova, B. Yang, Y. Wang, B. Ogedegbe, T. Riaz, M. Støen, C. Desler, et al. Spatial transcriptomics reveals genes associated with dysregulated mitochondrial functions and stress signaling in Alzheimer disease. *Iscience*, 23(10), 2020. PMCID: PMC7522123.

[41] Y. Wang, S. Ma, and W. L. Ruzzo. Spatial modeling of prostate cancer metabolic gene expression reveals extensive heterogeneity and selective vulnerabilities. *Scientific reports*, 10(1):3490, 2020. PMCID: PMC7044328.

[42] A. Rao, D. Barkley, G. S. França, and I. Yanai. Exploring tissue architecture using spatial transcriptomics. *Nature*, 596(7871):211–220, 2021. PMCID: PMC8475179.

[43] E. R. Parra. Methods to determine and analyze the cellular spatial distribution extracted from multiplex immunofluorescence data to understand the tumor microenvironment. *Frontiers in Molecular Biosciences*, 8:668340, 2021. PMCID: PMC8226163.

[44] O. Vipond et al. Multiparameter persistent homology landscapes identify immune cell spatial patterns in tumors. *Proceedings of the National Academy of Sciences*, 118(41):e2102166118, 2021. PMCID: PMC8522280.

[45] N. P. Canete, S. S. Iyengar, J. T. Ormerod, H. Baharlou, A. N. Harman, and E. Patrick. spicyR: Spatial analysis of in situ cytometry data in R. *Bioinformatics*, 38(11):3099–3105, 2022. PMCID: PMC9326848.

[46] C. Wilson et al. Tumor immune cell clustering and its association with survival in African American women with ovarian cancer. *PLoS Computational Biology*, 18(3):e1009900, 2022. PMCID: PMC8920290.

[47] W. Kuswanto, G. Nolan, and G. Lu. Highly multiplexed spatial profiling with CODEX: bioinformatic analysis and application in human disease. *Seminars in Immunopathology*, 45(1):145–157, 2023. PMCID: PMC9684921.

[48] T. Vu, J. Wrobel, B. G. Bitler, E. L. Schenk, K. R. Jordan, and D. Ghosh. SPF: a spatial and functional data analytic approach to cell imaging data. *PLOS Computational Biology*, 18(6):e1009486, 2022.

[49] V. Milosevic. Different approaches to Imaging Mass Cytometry data analysis. *Bioinformatics Advances*, 3(1):vbad046, 2023.

[50] N. Osher, J. Kang, S. Krishnan, A. Rao, and V. Baladandayuthapani. SPARTIN: a Bayesian method for the quantification and characterization of cell type interactions in spatial pathology data. *Frontiers in Genetics*, 14:1175603, 2023. PMCID: PMC10232864.

[51] S. Seal, B. Neelon, P. M. Angel, E. C. O'Quinn, E. Hill, T. Vu, D. Ghosh, A. S. Mehta, K. Wallace, and A. V. Alekseyenko. SpaceANOVA: Spatial co-occurrence analysis of cell types in multiplex imaging data using point process and functional ANOVA. *Journal of Proteome Research*, 23(4):1131–1143, 2024. PMCID: PMC11002919.

[52] E. Zhao, M. R. Stone, X. Ren, J. Guenthoer, K. S. Smythe, T. Pulliam, S. R. Williams, C. R. Uytingco, S. E. Taylor, P. Nghiem, et al. Spatial transcriptomics at subspot resolution with BayesSpace. *Nature biotechnology*, 39(11):1375–1384, 2021. PMCID: PMC8763026.

[53] K. Dong and S. Zhang. Deciphering spatial domains from spatially resolved transcriptomics with an adaptive graph attention auto-encoder. *Nature communications*, 13(1):1739, 2022. PMCID: PMC8976049.

[54] C. Xu, X. Jin, S. Wei, P. Wang, M. Luo, Z. Xu, W. Yang, Y. Cai, L. Xiao, X. Lin, et al. DeepST: identifying spatial domains in spatial transcriptomics by deep learning. *Nucleic Acids Research*, 50(22):e131–e131, 2022. PMCID: PMC9825193.

[55] C. Allen, Y. Chang, B. Neelon, W. Chang, H. J. Kim, Z. Li, Q. Ma, and D. Chung. A Bayesian multivariate mixture model for high throughput spatial transcriptomics. *Biometrics*, 2022. PMCID: PMC10134739.

[56] L. Shang and X. Zhou. Spatially aware dimension reduction for spatial transcriptomics. *Nature Communications*, 13(1):7203, 2022. PMCID: PMC9684472.

[57] C. Allen, Y. Chang, Q. Ma, and D. Chung. MAPLE: a hybrid framework for multi-sample spatial transcriptomics data. *bioRxiv*, pages 2022–02, 2022.

[58] Y. Long, K. S. Ang, M. Li, K. L. K. Chong, R. Sethi, C. Zhong, H. Xu, Z. Ong, K. Sachaphibulkij, A. Chen, et al. Spatially informed clustering, integration, and deconvolution of spatial transcriptomics with GraphST. *Nature Communications*, 14(1):1155, 2023. PMCID: PMC9977836.

[59] Y. Yan and X. Luo. Bayesian Integrative Region Segmentation in Spatially Resolved Transcriptomic Studies. *Journal of the American Statistical Association*, pages 1–13, 2024.

[60] Y. Ma and X. Zhou. Accurate and efficient integrative reference-informed spatial domain detection for spatial transcriptomics. *Nature Methods*, pages 1–14, 2024.

[61] V. Singhal, N. Chou, J. Lee, Y. Yue, J. Liu, W. K. Chock, L. Lin, Y.-C. Chang, E. M. L. Teo, J. Aow, et al. BANKSY unifies cell typing and tissue domain segmentation for scalable spatial omics data analysis. *Nature Genetics*, 56(3):431–441, 2024. PMCID: PMC10937399.

[62] M. Varrone, D. Tavernari, A. Santamaria-Martínez, L. A. Walsh, and G. Ciriello. CellCharter reveals spatial cell niches associated with tissue remodeling and cell plasticity. *Nature Genetics*, 56(1):74–84, 2024.

[63] Z. Yuan. MENDER: fast and scalable tissue structure identification in spatial omics data. *Nature Communications*, 15(1):207, 2024. PMCID: PMC10770058.

[64] C. M. Schürch et al. Coordinated cellular neighborhoods orchestrate antitumoral immunity at the colorectal cancer invasive front. *Cell*, 182(5):1341–1359, 2020. PMCID: PMC7479520.

[65] Z. Chen, I. Soifer, H. Hilton, L. Keren, and V. Jojic. Modeling multiplexed images with spatial-LDA reveals novel tissue microenvironments. *Journal of Computational Biology*, 27(8):1204–1218, 2020. PMCID: PMC7415889.

[66] E. Patrick, N. P. Canete, S. S. Iyengar, A. N. Harman, G. T. Sutherland, and P. Yang. Spatial analysis for highly multiplexed imaging data to identify tissue microenvironments. *Cytometry Part A*, 103(7):593–599, 2023.

[67] R. Ehsani, I. Jonassen, L. A. Akslen, and D. Kleftogiannis. LOCATOR: feature extraction and spatial analysis of the cancer tissue microenvironment using mass cytometry imaging technologies. *Bioinformatics Advances*, 3(1):vbad146, 2023. PMCID: PMC10597586.

[68] X. Peng, J. W. Smithy, M. Yosofvand, C. E. Kostrzewa, M. Bleile, F. D. Ehrich, J. Lee, M. A. Postow, M. K. Callahan, K. S. Panageas, et al. Decoding Spatial Tissue Architecture: A Scalable Bayesian Topic Model for Multiplexed Imaging Analysis. *bioRxiv*, pages 2024–10, 2024.

[69] H. Mi, S. Sivagnanam, W. J. Ho, S. Zhang, D. Bergman, A. Deshpande, A. S. Baras, E. M. Jaffee, L. M. Coussens, E. J. Fertig, et al. Computational methods and biomarker discovery strategies for spatial proteomics: a review in immuno-oncology. *Briefings in Bioinformatics*, 25(5):bbae421, 2024.

[70] L. Zhang, D. Chen, D. Song, X. Liu, Y. Zhang, X. Xu, and X. Wang. Clinical and translational values of spatial transcriptomics. *Signal Transduction and Targeted Therapy*, 7(1):111, 2022.

[71] R. Arora, C. Cao, M. Kumar, S. Sinha, A. Chanda, R. McNeil, D. Samuel, R. K. Arora, T. W. Matthews, S. Chandarana, et al. Spatial transcriptomics reveals distinct and conserved tumor core and edge architectures that predict survival and targeted therapy response. *Nature Communications*, 14(1):5029, 2023.

[72] Y. Jin, Y. Zuo, G. Li, W. Liu, Y. Pan, T. Fan, X. Fu, X. Yao, and Y. Peng. Advances in spatial transcriptomics and its applications in cancer research. *Molecular Cancer*, 23(1):129, 2024. PMCID: PMC9606570.

[73] S. Gibb and K. Strimmer. MALDIquant: a versatile R package for the analysis of mass spectrometry data. *Bioinformatics*, 28(17):2270–2271, 2012.

[74] P. Ràfols, D. Vilalta, J. Brezmes, N. Cañellas, E. Del Castillo, O. Yanes, N. Ramírez, and X. Correig. Signal preprocessing, multivariate analysis and software tools for MA (LDI)-TOF mass spectrometry imaging for biological applications. *Mass spectrometry reviews*, 37(3):281–306, 2018.

[75] K. A. Bemis, M. C. Föll, D. Guo, S. S. Lakkimsetty, and O. Vitek. Cardinal v. 3: a versatile open-source software for mass spectrometry imaging analysis. *Nature Methods*, 20(12):1883–1886, 2023.

[76] Y. Dong and U. Heinig. Mass Spectrometry Imaging Data Analysis with ShinyCardinal. *Preprint, Research Square*, 2024.

[77] J. E. Trosko, R. J. Ruch, et al. Cell-cell communication in carcinogenesis. *Front Biosci*, 3(3):d208–236, 1998.

[78] A. á. DeLise, L. Fischer, and R. Tuan. Cellular interactions and signaling in cartilage development. *Osteoarthritis and cartilage*, 8(5):309–334, 2000.

[79] B. L. Bassler. Small talk: cell-to-cell communication in bacteria. *Cell*, 109(4):421–424, 2002.

[80] D. Song, D. Yang, C. A. Powell, and X. Wang. Cell–cell communication: old mystery and new opportunity, 2019.

[81] J. Su, Y. Song, Z. Zhu, X. Huang, J. Fan, J. Qiao, and F. Mao. Cell–cell communication: new insights and clinical implications. *Signal Transduction and Targeted Therapy*, 9(1):196, 2024. PMCID: PMC11382761.

[82] M. Meier-Schellersheim, R. Varma, and B. R. Angermann. Mechanistic models of cellular signaling, cytokine crosstalk, and cell-cell communication in immunology. *Frontiers in immunology*, 10:2268, 2019. PMCID: PMC6798038.

[83] A. Dominiak, B. Chełstowska, W. Olejarz, and G. Nowicka. Communication in the cancer microenvironment as a target for therapeutic interventions. *Cancers*, 12(5):1232, 2020. PMCID: PMC7281160.

[84] N. K. Pandit. *Introduction to the pharmaceutical sciences.* Lippincott Williams & Wilkins, 2007.

[85] Y. Wang, R. Wang, S. Zhang, S. Song, C. Jiang, G. Han, M. Wang, J. Ajani, A. Futreal, and L. Wang. iTALK: an R package to characterize and illustrate intercellular communication. *BioRxiv*, page 507871, 2019.

[86] M. Efremova, M. Vento-Tormo, S. A. Teichmann, and R. Vento-Tormo. CellPhoneDB: inferring cell–cell communication from combined expression of multi-subunit ligand–receptor complexes. *Nature protocols*, 15(4):1484–1506, 2020.

[87] E. Armingol, A. Officer, O. Harismendy, and N. E. Lewis. Deciphering cell–cell interactions and communication from gene expression. *Nature Reviews Genetics*, 22(2):71–88, 2021. PMCID: PMC7649713.

[88] S. Jin, C. F. Guerrero-Juarez, L. Zhang, I. Chang, R. Ramos, C.-H. Kuan, P. Myung, M. V. Plikus, and Q. Nie. Inference and analysis of cell-cell communication using CellChat. *Nature communications*, 12(1):1088, 2021.

[89] Y. Zhang, T. Liu, X. Hu, M. Wang, J. Wang, B. Zou, P. Tan, T. Cui, Y. Dou, L. Ning, et al. CellCall: integrating paired ligand–receptor and transcription factor activities for cell–cell communication. *Nucleic acids research*, 49(15):8520–8534, 2021.

[90] Q. Liu, C.-Y. Hsu, J. Li, and Y. Shyr. Dysregulated ligand–receptor interactions from single-cell transcriptomics. *Bioinformatics*, 38(12):3216–3221, 2022. PMCID: PMC9191214.

[91] D. Dimitrov, D. Türei, M. Garrido-Rodriguez, P. L. Burmedi, J. S. Nagai, C. Boys, R. O. Ramirez Flores, H. Kim, B. Szalai, I. G. Costa, et al. Comparison of methods and resources for cell-cell communication inference from single-cell RNA-Seq data. *Nature communications*, 13(1):3224, 2022. PMCID: PMC9184522.

[92] Z. Liu, D. Sun, and C. Wang. Evaluation of cell-cell interaction methods by integrating single-cell RNA sequencing data with spatial information. *Genome Biology*, 23(1):218, 2022.

[93] J. Luo, M. Deng, X. Zhang, and X. Sun. ESICCC as a systematic computational framework for evaluation, selection, and integration of cell-cell communication inference methods. *Genome Research*, 33(10):1788–1805, 2023. PMCID: PMC10691505.

[94] A. A. Almet, Z. Cang, S. Jin, and Q. Nie. The landscape of cell–cell communication through single-cell transcriptomics. *Current opinion in systems biology*, 26:12–23, 2021. PMCID: PMC8104132.

[95] M. Bafna, H. Li, and X. Zhang. CLARIFY: cell–cell interaction and gene regulatory network refinement from spatially resolved transcriptomics. *Bioinformatics*, 39(Supplement_1):i484–i493, 2023. PMCID: PMC10311313.

[96] E. Armingol, H. M. Baghdassarian, and N. E. Lewis. The diversification of methods for studying cell–cell interactions and communication. *Nature Reviews Genetics*, 25(6):381–400, 2024. PMCID: PMC11139546.

[97] S. Banerjee, B. P. Carlin, and A. E. Gelfand. *Hierarchical modeling and analysis for spatial data.* Chapman and Hall/CRC, 2014.

[98] B. F. Miller, D. Bambah-Mukku, C. Dulac, X. Zhuang, and J. Fan. Characterizing spatial gene expression heterogeneity in spatially resolved single-cell transcriptomic data with nonuniform cellular densities. *Genome research*, 31(10):1843–1855, 2021. PMCID: PMC8494224.

[99] R. Dries, Q. Zhu, R. Dong, C.-H. L. Eng, H. Li, K. Liu, Y. Fu, T. Zhao, A. Sarkar, F. Bao, et al. Giotto: a toolbox for integrative analysis and visualization of spatial expression data. *Genome biology*, 22:1–31, 2021. PMCID: PMC7938609.

[100] Q. Liu, C.-Y. Hsu, and Y. Shyr. Scalable and model-free detection of spatial patterns and colocalization. *Genome research*, 32(9):1736–1745, 2022. PMCID: PMC9528978.

[101] X. Shao, C. Li, H. Yang, X. Lu, J. Liao, J. Qian, K. Wang, J. Cheng, P. Yang, H. Chen, et al. Knowledge-graph-based cell-cell communication inference for spatially resolved transcriptomic data with SpaTalk. *Nature Communications*, 13(1):4429, 2022. PMCID: PMC9338929.

[102] Z. Li, T. Wang, P. Liu, and Y. Huang. SpatialDM for rapid identification of spatially co-expressed ligand–receptor and revealing cell–cell communication patterns. *Nature communications*, 14(1):3995, 2023. PMCID: PMC10325966.

[103] S. Jin, M. V. Plikus, and Q. Nie. CellChat for systematic analysis of cell–cell communication from single-cell transcriptomics. *Nature Protocols*, pages 1–40, 2024.

[104] D. Dimitrov, P. S. L. Schäfer, E. Farr, P. Rodriguez-Mier, S. Lobentanzer, P. Badia-i Mompel, A. Dugourd, J. Tanevski, R. O. Ramirez Flores, and J. Saez-Rodriguez. LIANA+ provides an all-

in-one framework for cell–cell communication inference. *Nature Cell Biology*, 26(9):1613–1622, 2024. PMCID: PMC11392821.

[105] H. Sarkar, U. Chitra, J. Gold, and B. J. Raphael. A count-based model for delineating cell–cell interactions in spatial transcriptomics data. *Bioinformatics*, 40(Supplement_1):i481–i489, 2024. PMCID: PMC11211854.

[106] Z. Cang and Q. Nie. Inferring spatial and signaling relationships between cells from single cell transcriptomic data. *Nature communications*, 11(1):2084, 2020. PMCID: PMC7190659.

[107] Z. Cang, Y. Zhao, A. A. Almet, A. Stabell, R. Ramos, M. V. Plikus, S. X. Atwood, and Q. Nie. Screening cell–cell communication in spatial transcriptomics via collective optimal transport. *Nature Methods*, 20(2):218–228, 2023. PMCID: PMC9911355.

[108] D. Wartenberg. Multivariate spatial correlation: a method for exploratory geographical analysis. *Geographical analysis*, 17(4):263–283, 1985.

[109] M. M. Fischer and P. Nijkamp. *Geographic information systems, spatial modelling and policy evaluation.* Springer, 1993.

[110] S.-I. Lee. Developing a bivariate spatial association measure: an integration of Pearson's r and Moran's I. *Journal of geographical systems*, 3:369–385, 2001.

[111] L. Anselin, I. Syabri, O. Smirnov, et al. Visualizing multivariate spatial correlation with dynamically linked windows. In *Proceedings, CSISS Workshop on New Tools for Spatial Data Analysis, Santa Barbara, CA*, volume 2, 2002.

[112] S.-I. Lee. A generalized significance testing method for global measures of spatial association: an extension of the Mantel test. *Environment and Planning A*, 36(9):1687–1703, 2004.

[113] B. Delaunay. Sur la sphère vide. A la mémoire de Georges Voronoï. *Bulletin de l'Académie des Sciences de l'URSS*, (6):793–800, 1934.

[114] D. Liu, X. Lin, and D. Ghosh. Semiparametric regression of multidimensional genetic pathway data: least-squares kernel machines and linear mixed models. *Biometrics*, 63(4):1079–1088, 2007. PMCID: PMC2665800.

[115] C. K. Williams and C. E. Rasmussen. *Gaussian processes for machine learning*, volume 2. MIT press Cambridge, MA, 2006.

[116] L. Anselin. Local indicators of spatial association—LISA. *Geographical analysis*, 27(2):93–115, 1995.

[117] L. Moses, P. H. Einarsson, K. Jackson, L. Luebbert, A. S. Booeshaghi, S. Antonsson, N. Bray, P. Melsted, and L. Pachter. Voyager: exploratory single-cell genomics data analysis with geospatial statistics. *bioRxiv*, 2023.

[118] F. J. Anscombe. The transformation of Poisson, binomial and negative-binomial data. *Biometrika*, 35(3/4):246–254, 1948.

[119] C. Ahlmann-Eltze and W. Huber. Comparison of transformations for single-cell RNA-seq data. *Nature Methods*, 20(5):665–672, 2023. PMCID: PMC10172138.

[120] Y. Hao, T. Stuart, M. H. Kowalski, S. Choudhary, P. Hoffman, A. Hartman, A. Srivastava, G. Molla, S. Madad, C. Fernandez-Granda, et al. Dictionary learning for integrative, multimodal and scalable single-cell analysis. *Nature biotechnology*, 42(2):293–304, 2024. PMCID: PMC10928517.

[121] H. Li, B. Zhu, X. Jiang, L. Guo, Y. Xie, L. Xu, and Q. Li. An interpretable Bayesian clustering approach with feature selection for analyzing spatially resolved transcriptomics data. *Biometrics*, 80(3):ujae066, 2024. PMCID: PMC11285114.

[122] L. Atta, K. Clifton, M. Anant, G. Aihara, and J. Fan. Gene count normalization in single-cell imaging-based spatially resolved transcriptomics. *Genome Biology*, 25(1):153, 2024.

[123] R. O'Hara and J. Kotze. Do not log-transform count data. *Nature Precedings*, pages 1–1, 2010.

[124] X. Xiao, E. P. White, M. B. Hooten, and S. L. Durham. On the use of log-transformation vs. nonlinear regression for analyzing biological power laws. *Ecology*, 92(10):1887–1894, 2011.

[125] C. Feng, H. Wang, N. Lu, T. Chen, H. He, Y. Lu, and X. M. Tu. Log-transformation and its implications for data analysis. *Shanghai archives of psychiatry*, 26(2):105–109, 2014.

[126] R. M. West. Best practice in statistics: The use of log transformation. *Annals of Clinical Biochemistry*, 59(3):162–165, 2022. PMCID: PMC9036143.

[127] T. Schmidt. Coping with copulas. *Copulas-From theory to application in finance*, 3:1–34, 2007.

[128] J. H. Moore. Bootstrapping, permutation testing and the method of surrogate data. *Physics in Medicine & Biology*, 44(6):L11, 1999.

[129] R. P. Haining. *Spatial data analysis: theory and practice.* Cambridge university press, 2003.

[130] P. Clifford, S. Richardson, and D. Hemon. Assessing the significance of the correlation between two spatial processes. *Biometrics*, pages 123–134, 1989.

[131] R. Haining. Bivariate correlation with spatial data. *Geographical Analysis*, 23(3):210–227, 1991.

[132] S. Richardson and P. Clifford. Testing association between spatial processes. *Lecture Notes-Monograph Series*, pages 295–308, 1991.

[133] M. R. Dale and M.-J. Fortin. Spatial autocorrelation and statistical tests: some solutions. *Journal of Agricultural, Biological, and Environmental Statistics*, 14:188–206, 2009.

[134] D. A. Griffith. *Advanced spatial statistics: special topics in the exploration of quantitative spatial data series*, volume 12. Springer Science & Business Media, 2012.

[135] F. Mutiso, J. L. Pearce, S. E. Benjamin-Neelon, N. T. Mueller, H. Li, and B. Neelon. Bayesian negative binomial regression with spatially varying dispersion: Modeling COVID-19 incidence in Georgia. *Spatial Statistics*, 52:100703, 2022. PMCID: PMC9500097.

[136] A. E. Gelfand, H.-J. Kim, C. Sirmans, and S. Banerjee. Spatial modeling with spatially varying coefficient processes. *Journal of the American Statistical Association*, 98(462):387–396, 2003.

[137] T. Nakaya, A. S. Fotheringham, C. Brunsdon, and M. Charlton. Geographically weighted Poisson regression for disease association mapping. *Statistics in medicine*, 24(17):2695–2717, 2005.

[138] P. Congdon. Spatial heterogeneity in Bayesian disease mapping. *GeoJournal*, 84(5):1303–1316, 2019.

[139] B. Lu, M. Charlton, P. Harris, and A. S. Fotheringham. Geographically weighted regression with a non-Euclidean distance metric: a case study using hedonic house price data. *International Journal of Geographical Information Science*, 28(4):660–681, 2014.

[140] M. Helbich and D. A. Griffith. Spatially varying coefficient models in real estate: Eigenvector spatial filtering and alternative approaches. *Computers, Environment and Urban Systems*, 57:1–11, 2016.

[141] A. O. Finley. Comparing spatially-varying coefficients models for analysis of ecological data with non-stationary and anisotropic residual dependence. *Methods in ecology and evolution*, 2(2):143–154, 2011.

[142] N. Hamm, A. Finley, M. Schaap, and A. Stein. A spatially varying coefficient model for mapping PM10 air quality at the European scale. *Atmospheric Environment*, 102:393–405, 2015.

[143] H. Zhu, J. Fan, and L. Kong. Spatially varying coefficient model for neuroimaging data with jump discontinuities. *Journal of the American Statistical Association*, 109(507):1084–1098, 2014. PMCID: PMC4244662.

[144] T. Ge, N. Müller-Lenke, K. Bendfeldt, T. E. Nichols, and T. D. Johnson. Analysis of multiple sclerosis lesions via spatially varying coefficients. *The annals of applied statistics*, 8(2):1095, 2014. PMCID: PMC4243942.

[145] J. Besag. Spatial interaction and the statistical analysis of lattice systems. *Journal of the Royal Statistical Society: Series B (Methodological)*, 36(2):192–225, 1974.

[146] J. Besag, J. York, and A. Mollié. Bayesian image restoration, with two applications in spatial statistics. *Annals of the institute of statistical mathematics*, 43:1–20, 1991.

[147] J. Besag and C. Kooperberg. On conditional and intrinsic autoregressions. *Biometrika*, 82(4):733–746, 1995.

[148] S. Banerjee, A. E. Gelfand, A. O. Finley, and H. Sang. Gaussian predictive process models for large spatial data sets. *Journal of the Royal Statistical Society Series B: Statistical Methodology*, 70(4):825–848, 2008.

[149] A. Datta, S. Banerjee, A. O. Finley, and A. E. Gelfand. Hierarchical nearest-neighbor Gaussian process models for large geostatistical datasets. *Journal of the American Statistical Association*, 111(514):800–812, 2016.

[150] H. Rue and L. Held. *Gaussian Markov random fields: theory and applications.* Chapman and Hall/CRC, 2005.

[151] R. Tibshirani, M. Saunders, S. Rosset, J. Zhu, and K. Knight. Sparsity and smoothness via the fused lasso. *Journal of the Royal Statistical Society Series B: Statistical Methodology*, 67(1):91–108, 2005.

[152] A. Rinaldo. Properties and refinements of the fused lasso. *The Annals of Statistics*, 95(5B):2922–2952, 2009.

[153] G. Casella, M. Ghosh, J. Gill, and M. Kyung. Penalized regression, standard errors, and Bayesian lassos. *Bayesian Analysis*, 5(2):369 – 411, 2010.

[154] C. M. Carvalho, N. G. Polson, and J. G. Scott. Handling sparsity via the horseshoe. In *Artificial intelligence and statistics*, pages 73–80. PMLR, 2009.

[155] S. Banerjee. Horseshoe shrinkage methods for Bayesian fusion estimation. *Computational Statistics & Data Analysis*, 174:107450, 2022.

[156] Y. Kakikawa, K. Shimamura, and S. Kawano. Bayesian fused lasso modeling via horseshoe prior. *Japanese Journal of Statistics and Data Science*, 6(2):705–727, 2023.

[157] F. Li and H. Sang. Spatial homogeneity pursuit of regression coefficients for large datasets. *Journal of the American Statistical Association*, 2019.

[158] R. C. Prim. Shortest connection networks and some generalizations. *The Bell System Technical Journal*, 36(6):1389–1401, 1957.

[159] R. L. Graham and P. Hell. On the history of the minimum spanning tree problem. *Annals of the History of Computing*, 7(1):43–57, 1985.

[160] J. M. Steele. Minimal spanning trees for graphs with random edge lengths. In *Mathematics and Computer Science II: Algorithms, Trees, Combinatorics and Probabilities*, pages 223–245. Springer, 2002.

[161] A. L. Ji, A. J. Rubin, K. Thrane, S. Jiang, D. L. Reynolds, R. M. Meyers, M. G. Guo, B. M. George, A. Mollbrink, J. Bergenstråhle, et al. Multimodal analysis of composition and spatial architecture in human squamous cell carcinoma. *cell*, 182(2):497–514, 2020. PMCID: PMC7391009.

[162] D. M. Cable, E. Murray, L. S. Zou, A. Goeva, E. Z. Macosko, F. Chen, and R. A. Irizarry. Robust decomposition of cell type mixtures in spatial transcriptomics. *Nature biotechnology*, 40(4):517–526, 2022. PMCID: PMC8606190.

[163] P. Langfelder and S. Horvath. Fast R functions for robust correlations and hierarchical clustering. *Journal of statistical software*, 46:1–17, 2012.

[164] P. Delafontaine, Y.-H. Song, and Y. Li. Expression, regulation, and function of IGF-1, IGF-1R, and IGF-1 binding proteins in blood vessels. *Arteriosclerosis, thrombosis, and vascular biology*, 24(3):435–444, 2004.

[165] E. Capoluongo. Insulin-like growth factor system and sporadic malignant melanoma. *The American journal of pathology*, 178(1):26–31, 2011. PMCID: PMC3069928.

[166] T. R. Bakker, C. Piperi, E. A. Davies, and P. A. v. d. Merwe. Comparison of CD22 binding to native CD45 and synthetic oligosaccharide. *European journal of immunology*, 32(7):1924–1932, 2002.

[167] X. Li, Z. Yue, D. Wang, and L. Zhou. PTPRC functions as a prognosis biomarker in the tumor microenvironment of cutaneous melanoma. *Scientific Reports*, 13(1):20617, 2023. PMCID: PMC10667527.

[168] L. Nitschke, R. Carsetti, B. Ocker, G. Köhler, and M. C. Lamers. CD22 is a negative regulator of B-cell receptor signalling. *Current Biology*, 7(2):133–143, 1997.

[169] F. W. Bazer, G. Song, J. Kim, D. W. Erikson, G. A. Johnson, R. C. Burghardt, H. Gao, M. C. Satterfield, T. E. Spencer, and G. Wu. Mechanistic mammalian target of rapamycin (MTOR) cell signaling: effects of select nutrients and secreted phosphoprotein 1 on development of mammalian conceptuses. *Molecular and cellular endocrinology*, 354(1-2):22–33, 2012.

[170] W. Xie, J. Cheng, Z. Hong, W. Cai, H. Zhuo, J. Hou, L. Lin, X. Wei, K. Wang, X. Chen, et al. Multi-transcriptomic analysis reveals the heterogeneity and tumor-promoting role of SPP1/CD44-mediated intratumoral crosstalk in gastric cancer. *Cancers*, 15(1):164, 2022. PMCID: PMC9818284.

[171] D. E. Rowe, R. J. Carroll, and C. L. Day Jr. Prognostic factors for local recurrence, metastasis, and survival rates in squamous cell carcinoma of the skin, ear, and lip: implications for treatment modality selection. *Journal of the American Academy of Dermatology*, 26(6):976–990, 1992.

[172] J. T. Jacob, P. A. Coulombe, R. Kwan, and M. B. Omary. Types I and II keratin intermediate filaments. *Cold Spring Harbor perspectives in biology*, 10(4):a018275, 2018. PMCID: PMC5880164.

[173] L. Yang, S. Zhang, and G. Wang. Keratin 17 in disease pathogenesis: from cancer to dermatoses. *The Journal of pathology*, 247(2):158–165, 2019.

[174] S. Werner, L. Keller, and K. Pantel. Epithelial keratins: Biology and implications as diagnostic markers for liquid biopsies. *Molecular aspects of medicine*, 72:100817, 2020.

[175] O. Ogunnigbagbe, C. G. Bunick, and K. Kaur. Keratin 1 as a cell-surface receptor in cancer. *Biochimica et Biophysica Acta (BBA)-Reviews on Cancer*, 1877(1):188664, 2022. PMCID: PMC8818032.

[176] M. N. Bernstein, Z. Ni, A. Prasad, J. Brown, C. Mohanty, R. Stewart, M. A. Newton, and C. Kendziorski. SpatialCorr identifies gene sets with spatially varying correlation structure. *Cell Reports Methods*, 2(12), 2022. PMCID: PMC9795364.

[177] A. Buruiană, B.-A. Gheban, I.-A. Gheban-Roșca, C. Georgiu, D. Crișan, and M. Crișan. The tumor stroma of squamous cell carcinoma: a complex environment that fuels cancer progression. *Cancers*, 16(9):1727, 2024. PMCID: PMC11083853.

[178] P. E. Bowden. Mutations in a keratin 6 isomer (K6c) cause a type of focal palmoplantar keratoderma. *Journal of Investigative Dermatology*, 130(2):336–338, 2010.

[179] M. Fu and G. Wang. Keratin 17 as a therapeutic target for the treatment of psoriasis. *Journal of Dermatological Science*, 67(3):161–165, 2012.

[180] G. Baraks, R. Tseng, C.-H. Pan, S. Kasliwal, C. V. Leiton, K. R. Shroyer, and L. F. Escobar-Hoyos. Dissecting the oncogenic roles of keratin 17 in the hallmarks of cancer. *Cancer research*, 82(7):1159–1166, 2022. PMCID: PMC9016724.

[181] Y. Lin, W. Zhang, B. Li, and G. Wang. Keratin 17 in psoriasis: Current understanding and future perspectives. In *Seminars in cell & developmental biology*, volume 128, pages 112–119. Elsevier, 2022.

[182] S. Chootipongchaivat, N. T. van Ravesteyn, X. Li, H. Huang, H. Weedon-Fekjær, M. D. Ryser, D. L. Weaver, E. S. Burnside, B. M. Heckman-Stoddard, H. J. de Koning, et al. Modeling the natural history of ductal carcinoma in situ based on population data. *Breast Cancer Research*, 22:1–12, 2020. PMCID: PMC7251719.

[183] R. L. Siegel, K. D. Miller, H. E. Fuchs, and A. Jemal. Cancer statistics, 2022. *CA: a cancer journal for clinicians*, 72(1):7–33, 2022.

[184] E. S. Novoseletskaya, P. V. Evdokimov, and A. Y. Efimenko. Extracellular matrix-induced signaling pathways in mesenchymal stem/stromal cells. *Cell Communication and Signaling*, 21(1):244, 2023. PMCID: PMC10507829.

[185] T. S. Hulahan and P. M. Angel. From ductal carcinoma in situ to invasive breast cancer: the prognostic value of the extracellular microenvironment. *Journal of Experimental & Clinical Cancer Research*, 43(1):329, 2024. PMCID: PMC11664872.

[186] C. G. Kittrell, J. Macdonald, B. Sells, L. E. Young, D. DeNardo, P. M. Angel, and R. R. Drake. Establishing a multi-omic spatial ECM proteome and N-glycome of pancreatic ductal adenocarcinoma tissues. *Cancer Research*, 85(8_Supplement_1):2581–2581, 2025.

[187] A. M. Frieze. On the value of a random minimum spanning tree problem. *Discrete Applied Mathematics*, 10(1):47–56, 1985.

[188] Z. Zhao, A. Ukidve, J. Kim, and S. Mitragotri. Targeting strategies for tissue-specific drug delivery. *Cell*, 181(1):151–167, 2020.

[189] S. Yan, J. Na, X. Liu, and P. Wu. Different targeting ligands-mediated drug delivery systems for tumor therapy. *Pharmaceutics*, 16(2):248, 2024. PMCID: PMC10893104.

[190] Y. Sung, Y. Choi, E. S. Kim, J. H. Ryu, and I. C. Kwon. Receptor-ligand interactions for optimized endocytosis in targeted therapies. *Journal of Controlled Release*, 380:524–538, 2025.

[191] A. Datta, S. Banerjee, J. S. Hodges, and L. Gao. Spatial disease mapping using directed acyclic graph auto-regressive (DAGAR) models. *Bayesian analysis*, 14(4):1221, 2019. PMCID: PMC8046356.

[192] Z. T. Luo, H. Sang, and B. Mallick. A Bayesian contiguous partitioning method for learning clustered latent variables. *Journal of Machine Learning Research*, 22(37):1–52, 2021.

[193] J. Pillow and J. Scott. Fully Bayesian inference for neural models with negative-binomial spiking. *Advances in neural information processing systems*, 25, 2012.

[194] S. Cheon, S. H. Song, and B. C. Jung. Tests for independence in a bivariate negative binomial model. *Journal of the Korean Statistical Society*, 38(2):185–190, 2009.

[195] F. Famoye. On the bivariate negative binomial regression model. *Journal of Applied Statistics*, 37(6):969–981, 2010.

[196] H. Cho, C. Liu, J. S. Preisser, and D. Wu. A bivariate zero-inflated negative binomial model and its applications to biomedical settings. *Statistical Methods in Medical Research*, 32(7):1300–1317, 2023.

[197] N. Ahmad, V. V. Gayah, and E. T. Donnell. Copula-based bivariate count data regression models for simultaneous estimation of crash counts based on severity and number of vehicles. *Accident Analysis & Prevention*, 181:106928, 2023.

[198] M. Iqbal, A. P. U. Siahaan, N. E. Purba, and D. Purwanto. Prim's Algorithm for Optimizing Fiber Optic Trajectory Planning. *Int. J. Sci. Res. Sci. Technol*, 3(6):504–509, 2017.

[199] D. Hallac, J. Leskovec, and S. Boyd. Network lasso: Clustering and optimization in large graphs. In *Proceedings of the 21th ACM SIGKDD international conference on knowledge discovery and data mining*, pages 387–396, 2015.

[200] T. Park and G. Casella. The bayesian lasso. *Journal of the american statistical association*, 103(482):681–686, 2008.

[201] R. Tibshirani. Regression shrinkage and selection via the lasso. *Journal of the Royal Statistical Society Series B: Statistical Methodology*, 58(1):267–288, 1996.

[202] T. Sakai, J. Tsuchida, and H. Yadohisa. Bayesian geographically weighted regression using Fused Lasso prior. *Spatial Statistics*, page 100884, 2025.

[203] V. Rockova, E. Lesaffre, J. Luime, and B. Löwenberg. Hierarchical Bayesian formulations for selecting variables in regression models. *Statistics in medicine*, 31(11-12):1221–1237, 2012.

[204] M. Pavlou, G. Ambler, S. Seaman, M. De Iorio, and R. Z. Omar. Review and evaluation of penalised regression methods for risk prediction in low-dimensional data with few events. *Statistics in medicine*, 35(7):1159–1177, 2016. PMCID: PMC4982098.

[205] K. Walters, A. Cox, and H. Yaacob. The utility of the Laplace effect size prior distribution in Bayesian fine-mapping studies. *Genetic epidemiology*, 45(4):386–401, 2021.

[206] A. Bhadra, J. Datta, N. G. Polson, and B. Willard. The horseshoe+ estimator of ultra-sparse signals. *Arxiv*, 2017.

[207] J. Piironen and A. Vehtari. Sparsity information and regularization in the horseshoe and other shrinkage priors. *Arxiv*, 2017.

[208] A. Bhadra, J. Datta, N. G. Polson, and B. Willard. Default Bayesian analysis with global-local shrinkage priors. *Biometrika*, 103(4):955–969, 2016.

[209] M. Denis and M. G. Tadesse. Graph-structured variable selection with Gaussian Markov random field horseshoe prior. *Statistical Modelling*, page 1471082X241310958, 2023.

[210] J. R. Faulkner and V. N. Minin. Locally adaptive smoothing with Markov random fields and shrinkage priors. *Bayesian analysis*, 13(1):225, 2017.

[211] N. G. Polson, J. G. Scott, and J. Windle. Bayesian inference for logistic models using Pólya–Gamma latent variables. *Journal of the American statistical Association*, 108(504):1339–1349, 2013.

[212] P. M. Lee. *Bayesian statistics.* Oxford University Press London:, 1989.

[213] D. Makowski, M. S. Ben-Shachar, and D. Lüdecke. bayestestR: Describing effects and their uncertainty, existence and significance within the Bayesian framework. *Journal of open source software*, 4(40):1541, 2019.

[214] D. Makowski, M. S. Ben-Shachar, S. A. Chen, and D. Lüdecke. Indices of effect existence and significance in the Bayesian framework. *Frontiers in psychology*, 10:2767, 2019.

[215] R. Bivand. R packages for analyzing spatial data: A comparative case study with areal data. *Geographical Analysis*, 54(3):488–518, 2022.

Manuscript

Click here to access/download
Supplementary Material
SpaceBF_supp_GR.pdf
