# Supplementary material for: SpaceBF: spatial coexpression analysis using Bayesian fused approaches in spatial omics datasets
Source: Gigascience. 2026 Jan 20;15:giag006. doi: 10.1093/gigascience/giag006 (PMC12954175; doi:10.1093/gigascience/giag006)
Supplement: giag006_GIGA-D-25-00259_Revision_1 [file giag006_giga-d-25-00259_revision_1.pdf]

GigaScience

SpaceBF: Spatial coexpression analysis using Bayesian Fused approaches in spatial omics datasets

--Manuscript Draft--

**Manuscript Number:**	GIGA-D-25-00259R1
**Full Title:**	SpaceBF: Spatial coexpression analysis using Bayesian Fused approaches in spatial omics datasets
**Article Type:**	Technical Note
**Funding Information:**	NIH Clinical Center — Dr. Souvik Seal<br>American Cancer Society (IRG-24-1290553-23- IRG) — Dr. Souvik Seal
**Abstract:**	Advances in spatial omics enable measurement of genes (spatial transcriptomics) and peptides, lipids,or N-glycans (mass spectrometry imaging) across thousands of locations within a tissue. While detectingspatially variable molecules is a well-studied problem, robust methods for identifying spatially varyingco-expression between molecule pairs remain limited. We introduce SpaceBF, a Bayesian fused modelingframework that estimates co-expression at both local (location-specific) and global (tissue-wide) levels.SpaceBF enforces spatial smoothness via a fused horseshoe prior on the edges of a predefined spatialadjacency graph, allowing large, edge-specific differences to escape shrinkage while preserving overallstructure. In extensive simulations, SpaceBF achieves higher specificity and power than commonly usedmethods that leverage geospatial metrics, including bivariate Moran's I and Lee's L. We also benchmarkthe proposed prior against standard alternatives, such as intrinsic conditional autoregressive (ICAR) andMatern priors. Applied to spatial transcriptomics and proteomics datasets, SpaceBF reveals cancer-relevantmolecular interactions and patterns of cell–cell communication (e.g., ligand–receptor signaling),demonstrating its utility for principled, uncertainty-aware co-expression analysis of spatial omics data.
**Corresponding Author:**	Souvik Seal<br>Medical University of South Carolina<br>CHARLESTON, SC UNITED STATES
**Corresponding Author Secondary Information:**	
**Corresponding Author's Institution:**	Medical University of South Carolina
**Corresponding Author's Secondary Institution:**	
**First Author:**	Souvik Seal
**First Author Secondary Information:**	
**Order of Authors:**	Souvik Seal<br>Brian Neelon
**Order of Authors Secondary Information:**	
**Response to Reviewers:**	We thank the reviewers and the editor for their invaluable comments. We have attached a PDF file to the resubmission with our responses, which we will try to copy-paste here, but the formatting might be off with figures not displaying. Please consider the PDF file for review.<br><br>1General comments<br>We thank the reviewers for their insightful comments, which motivated several substantial additions, including a lot of new results and theoretical developments. In particular, we have extended the proposed spatial horseshoe prior to support general graphs (beyond the MST), and we now include additional benchmarks against commonly used spatial priors, including ICAR and Mat´ern priors. In addition, we derive

Powered by Editorial Manager® and ProduXion Manager® from Aries Systems Corporation

the asymptotic distribution of the bivariate Moran's I statistic and argue mathematically why it can be erratic when the two variables (e.g., genes) are truly independent, yet each exhibits spatial autocorrelation. All changes to the main text are highlighted in the color blue. The reference numbers here will not match the main text as they appear in document-specific order.

2Reviewer 1

Summary: The manuscript introduces a novel statistical framework for analyzing spatially varying molecular co-expression. Leveraging a Bayesian fused modeling approach, SpaceBF estimates both local (locationspecific) and global (tissue-wide) co-expression patterns, particularly useful for studying cell-cell communication via ligand-receptor interactions. The method outperforms traditional geospatial metrics like bivariate Moran's I and Lee's L in terms of specificity and precision. Application of SpaceBF to spatial omics data reveals new insights into molecular interactions across various cancer types, offering a powerful tool for spatial omics research. The paper is nicely written, well structured, and has great visualizations, but I have the following comments.

1.The authors missed a couple of key references related to co-expression analysis of spatial omics data such as JOBS (Chakrabarti et al., 2024) and SpaceX (Acharyya et al., 2022). The authors are recommended to include these references in the Introduction Section.

Response: We thank the reviewer for pointing out the manuscripts. We believe that these methods could be invaluable in extending our framework further, so we have now cited them in the Discussion
section:
"We have focused on pairwise analyses thus far; extending to joint modeling will follow prior works
[1, 2]."

2.A method-related figure can be included for visual illustration of the method.
Response: We thank the reviewer for the comment. We have now added a summary figure.

3.In Melanoma ST data analysis, authors have used the RCTD algorithm (Cable et al., 2022) for celltype estimation. It seems like the gene expression matrix has been used twice in the whole process: once in case of cell-type estimation and co-expression analysis afterwards. The obtained results can be highly correlated due to multiple uses of the gene expression matrix. It would be great if authors can address this issue.

Response: We thank the reviewer for the comment and apologize for the confusion. To clarify, the RCTD cell-types are shown solely for visual comparison and are not used in our model. We now state explicitly that the model includes no covariates:
"After filtering out genes with extremely low expression ($< 0.2 \times 293 \approx 59$ reads), 161 LR pairs remain, which were examined using our method SpaceBF, without adjusting for any covariates".
We also mention in the methods section that:
"To clarify, all applications in the manuscript assume no covariates, i.e., we do not include $C(s_k)$ or α_m, for simplicity. "
4.In the cSCC ST data analysis, BayesSpace (Zhao et al., 2021) algorithm has been used for spatial region identification. In Figure 2C, cluster numbers are provided only and those are not transferred to spatial regions. It is difficult to make spatial region specific inference without such regional annotation of clusters. The gene expression matrix is used multiple times in this case as well (spatial region identification and co-expression analysis).

Response: We thank the reviewer for the comment. The BayesSpace clusters are presented solely to visually elucidate the tumor microenvironment (TME) and are not used in our model. Our intention was to illustrate that tumor versus non-tumor regions are not readily discernible from histology alone, whereas BayesSpace reveals differences in molecular expression/co-expression patterns. We state more clearly now in the main text:

"We emphasize that these clusters are shown for visualization only and are not used in our analysis."

5.The spatial omics datasets are sparse in nature. It is possible that some these edges may not exist if the molecules are far apart. Authors are requested to justify the use shrinkage prior such as horseshoe rather than spike-and-slab prior.
Response: We thank the reviewer for this insightful comment. To clarify, our prior penalizes pairwise differences between coefficients at adjacent locations in the spatial graph. Specifically, for the i-th
edge connecting (ski1,ski2), the penalty acts on . Locations that are distant
are not linked by an edge and are therefore not directly penalized. A spike-and-slab prior on pairwise differences is feasible, but it is likely more computationally challenging than a horseshoe prior, which affords more tractable MCMC sampling. Furthermore, one can induce additional sparsity on the coefficients themselves by placing extra shrinkage priors on the individual) terms.

6.While the authors briefly mention about the associated computational costs, it is recommended to include a comparison of the computational costs for different approaches in the simulation studies. This would provide a more comprehensive understanding of the proposed method's efficiency and feasibility. It will be also interesting to see the scalability of the method for large scale datasets.

Response: We thank the reviewer for the input. We have now moved the computational cost section from the supplementary to the main text and enhanced it with more benchmarks (with different adjacency graphs and priors) and comments in a new subsection titled "Runtime comparison and convergence diagnostics":
"In most analyses we ran 5,000 MCMC iterations with 2,500 burn-in. We compared runtimes for our package SpaceBF across priors and spatial backbones (from sparser to denser). Figure 2 shows that HS and ICAR have comparable runtimes, scaling approximately linearly with n. Denser graphs (e.g., k-NN with k = 9) are marginally slower. For n = 5,000, SpaceBF completes in about 20 minutes on a Mac Pro (M3 Max). For substantially larger datasets, a practical alternative is to consider sdmTMB [3], which fits an NB SVC model via a Laplace-approximate maximum likelihood approach. It is extremely fast but can be less precise, may fail to converge, and often requires tuning the mesh density for interpretable results.
For the convergence diagnostics, we computed the Geweke statistic [4] for each), implemented in the R package coda [5], and investigated the trace plots of a few randomly chosen)'s (see the Supplementary Material). When either the variable m or m′ is highly sparse (> 75% zeroes), imposing additional normal priors on)'s and)'s with a moderate variance, such as
N(0,10), drastically improves mixing and overall convergence performance."
We further discuss the complexity in the Discussion section:
"Using the MST as the spatial graph offers several benefits: (i) uniqueness, removing the need to tune additional graph hyperparameters (e.g., GP lengthscales [6]); (ii) reduced computational burden via an exceptionally sparse precision matrix; and (iii) exact Gibbs updates for local horseshoe scales. In our simulations with spatial autocorrelation generated from a Gaussian process with an exponential kernel and varying lengthscales (but a domain-constant slope), the MST performs well, underscoring its robustness. Nonetheless, restricting the spatial structure to a single spanning tree can exclude salient edges [7], yielding noisier local slope estimates and overly sharp transition boundaries when coefficients vary spatially. In practice, a moderately denser graph, such as a kNN network with a small k, often achieves a better trade-off between computational efficiency and appropriate smoothness, as observed in our simulations. A more principled avenue could be to treat the spanning tree as unknown and update it iteratively within the model [8]. While we leverage the spam package [9] for fast sparse Cholesky factorization, overall complexity is graph-structure dependent (e.g., near O(n) on trees/MSTs and typically around O(n3/2) time for 2D planar/kNN graphs) [10]. As future work, we will pursue MCMC-free, variational-inference-based estimation to improve scalability [11, 12]."
7. To ensure the robustness of the proposed methodology, it is requested that the authors include a detailed sensitivity analysis for the selected priors and parameters.
Response: We thank the reviewer for the comment. For the horseshoe prior, we have simply used Carvalho et al. (2009)'s formulation [13],
,

where C+(0,1) denotes a half-cauchy distribution with location 0 and scale 1, and no additional hyperparameters require tuning ($\sigma 2 = 1$ in the NB model). Subsequent work [14] has suggested alternative priors for the global scale $\tau 1$ that may further improve performance; we leave this extension to future work. However, we conducted a comprehensive sensitivity analysis with respect to the choice

Figure 3: A. Power comparison of spatial priors under simulation design 2 for lengthscale l between {1.8,3.6,7.2}. B. MSE comparison of spatial priors under simulation design 3, linear partition boundary. C. MSE comparison of spatial priors under simulation design 3, circular boundary. In panel A, sdmTMB models are omitted due to recurrent convergence issues.
of adjacency graph, which indicated that the MST may not always be the most suitable option for our prior. Please see Sections 2.2.3 and 4.4.3. We have added substantial new text and several additional figures; for brevity, we present one representative figure here for reference.

3Reviewer 2

I read your manuscript “SpaceBF: Spatial coexpression analysis using Bayesian Fused approaches in spatial omics datasets” with interest. The manuscript presents SpaceBF, a Bayesian method for detecting spatial co-expression between pairs of molecules in spatial omics data. The topic is relevant since new technologies like spatial transcriptomics, mass spectrometry imaging, and multiplex immunofluorescence produce large data but current tools for co-expression are limited. The authors try to solve this gap with a new model and they also test it on real datasets. The paper is technical, but it also gives biological examples, which is helpful for readers. The paper has many strong points. First, the idea to use Bayesian fused horseshoe prior together with MST spatial structure is new and well explained. Second, the authors apply their method on three real datasets and they show interesting biology, for example IGF2-IGF1R relation, keratin isoform consistency, and stromal ECM peptides. Third, I appreciate that the code is open on GitHub. Also, the paper compares with other methods and deals with the common problem of variance-stabilizing transform by modeling UMI counts directly with negative binomial distribution. Overall, the work is clear and well organized, but there are some points where more explanation or clarification would help. In my review I give major and minor remarks that I hope will improve the paper.

3.1Major remarks:

1.Were you worried choosing MST may oversimplify spatial relationships, since many meaningful local neighborhoods may be excluded? Would the results of SpaceBF be significantly different if a different spatial graph, such as kNN, Delaunay triangulation, or kernel-based, was used instead of MST?

Response: We thank the reviewer for this helpful comment. As noted by the reviewer and mentioned in the Discussion section, an MST may be insufficient to capture spatial relationships in general datasets. Following the reviewer’s suggestion, we went back and conducted additional experiments, finding that our framework extends naturally to other spatial graphs, including kNN and Delaunay. We now report results using a Delaunay graph, where our initial concern about oversmoothing did not materialize in practice. We also compare our priors (with both MST and Delaunay) against a standard ICAR prior (with Delaunay), showing that our approach outperforms ICAR with minimal computational overhead. Note that, compared with an MST, a general spatial graph yields a precision matrix with more nonzero entries, increasing the computational cost of the Cholesky decomposition.
We have added these new results in Sections 2.2.3 and 4.4.3, including the Figure 3 that is listed above. We are attaching only a small excerpt of the additions below for reference:
“While the spatial horseshoe (HS) prior is introduced on a minimum spanning tree (MST), it can be placed on any spatial backbone (e.g., Delaunay or kNN graphs), albeit with a potential risk of oversmoothing. This simulation study evaluates how graph choice affects HS performance. A Delaunay network is substantially denser than an MST, whereas a kNN network can serve as a middle ground for small k. In Fig. 3, HS-MST denotes HS on the MST (the original SpaceBF setting used in previous simulations and applications), HS-Del denotes HS on the Delaunay graph, and HS-kNN denotes HS on a kNN graph with $k = 3$. As noted in the Methods section, the ICAR prior is a special case of the HS prior; we therefore include ICAR-Del and ICAR-kNN for comparison. For completeness, we also consider a stochastic partial

differential equation (SPDE) [15]-based NB SVC model implemented in the efficient R package sdmTMB [3], which uses a Mat´ern prior: sdmTMB-Mat´ern1 uses a denser mesh (cutoff = 1), and sdmTMB-Mat´ern2 uses a coarser mesh (cutoff = 1.5), see the Supplementary Material for a visual comparison.”
2.Since MST edges depend a lot on pairwise L2 distances, how stable are the results if spatial coordinates are a little noisy, or if there are tissue registration errors?

Response: We thank the reviewer for this excellent comment. Following the previous response, we believe that using a general, denser spatial graph will mitigate this problem. It will more strongly smooth the local slope estimates, potentially avoiding random registration errors.

3.The model puts one molecule as outcome and the other as predictor. Are the co-expression estimates still the same if you switch roles?

Response: We thank the reviewer for the comment. In our experiments, interchanging the roles of the outcome and predictor genes had minimal impact on the results, i.e., the top selected pairs are the same. This is a minor limitation of the framework, and we briefly discuss it in the Supplementary
Material. The choice can also be guided by biological considerations, e.g., treating the receptor as the outcome and the ligand as the predictor, reflecting the canonical direction of signal transduction. In practice, such prior knowledge of pathway architecture can inform the choice and improve
interpretability.

4.In the Results you mention “FDR < 0.1”. Can you explain which method you used for FDR? Also, are the discoveries robust if you change the threshold (for example 0.05 vs 0.1)?

Response: We thank the reviewer for the comment. We kept FDR at a modest 0.1 as our study is exploratory. As mentioned in the manuscript, we compute the approximate “p-value” using the R package bayestestR [16], and then adjust those values using the Benjamini-Hochberg method using “p.adjust” function in R. We have now added to Section 4.3 of the main text:
“For FDR control, we apply the Benjamini–Hochberg procedure using the p.adjust function in R.” Changing the threshold, we lose 6 LR pairs.

5.Do the simulation parameters (lengthscale, slope, dispersion) correspond to realistic biological signal strengths and spatial scales observed in real datasets? Three values of the lengthscale l are considered, l = 3.6, 7.2, 18. Why exactly these values? What does v = 0.75 mean in terms of effect size? How does l=18 compare to real tissue lengthscales?

Response: We thank the reviewer for this helpful comment. The kernel lengthscale governs the range of spatial correlation: larger lengthscales induce stronger, longer-range dependence and smoother fields. In Figure 4A of the main text, we illustrate how the kernel matrix evolves as the lengthscale l increases from low to high. Although the values $l \in \{3.6,7.2,18\}$ may appear arbitrary, they were selected to span regimes of increasing correlation range. At l = 18, most off-diagonal entries of the kernel covariance matrix approach 1, indicating extremely high spatial dependence. While we did not estimate l from real data in this study, we deliberately explored a broad range of values in our simulations to stress-test the method and cover worst-case scenarios. v is a correlation parameter taking values in [−1,1], with larger (smaller) values indicating a stronger positive (negative) association. A value of v = 0.75 denotes strong positive co-expression, which is likely extreme in the real data context.

6.Can you describe runtime and memory for larger datasets, like 10X Visium with 5,000-20,000 spots? Is the current MCMC practical for this scale, or do you think approximate inference (like variational Bayes or INLA) is needed?

Response: We thank the reviewer for raising this important point. Leveraging the efficient spam package in R, our methods are tractable for datasets with approximately 10,000 spots, with runtimes on the order of minutes (as shown in the figure). As a natural next step toward scaling to larger platforms such as Xenium, we plan to

	develop a variational inference implementation following several recent works in this area. Please see the detailed response to comment 6 of reviewer 1 to find our new additions to the text regarding this topic.<br>3.2Minor remarks:<br>1.How sensitive are the results to the choice of hyperparameters for the Horseshoe prior?<br>Response: We thank the reviewer for the comment. For the horseshoe prior, we have simply used Carvalho et al. (2009)'s formulation [13],<br>,<br>where C+(0,1) denotes a half-cauchy distribution with location 0 and scale 1, and no additional hyperparameters require tuning (σ2 = 1 in the NB model). Subsequent work [14] has suggested alternative priors for the global scale τ1 that may further improve performance; we leave this extension to future work.<br><br>2.In the Results you state that keratins "co-express highly, meaning their binding patterns with any specific type 1 keratin should be similar." Please make clear that SpaceBF measures co-expression, not direct binding, so that conclusions are not overstated.<br><br>Response: We thank the reviewer for the suggestion. We have now edited the text as "One important observation is that the Type 2 keratins KRT6A, KRT6B, and KRT6C are closely related isoforms of keratin 6 [17] and therefore tend to be strongly co-expressed. Consequently, their spatial association patterns with a given Type 1 keratin are expected to be similar, consistent with the patterns recovered by SpaceBF."<br><br>3.You mention SpatialCorr and Copulacci, but the comparison was not successful. Even if parameters were sensitive, I think one short numerical comparison in the supplement would be helpful.<br><br>Response: We thank the reviewer for this suggestion. In attempting to benchmark SpatialCorr and Copulacci, we found that both packages incorporate substantial built-in preprocessing and/or require explicit cell-type information, which is not straightforward to disable and is often unnecessary for the simple bivariate association testing problem considered in our manuscript. In addition, the available documentation did not provide sufficient guidance. After corresponding with the authors via GitHub and adapting portions of the implementations to ensure applicability to our simulation design, we obtained unstable and/or clearly incorrect outputs (e.g., SpatialCorr had almost 0 power in every case) that we could not reconcile within the scope of this revision. As these issues may reflect our implementation choices or an incomplete interpretation of the software, we do not present these results as a formal comparison at this time. We will continue to investigate these packages in follow-up work.<br><br>4.You filter out genes with fewer than 59 total reads (0.2 x number of spots). Can you justify the choice of this threshold and show if results are stable for other thresholds (for example 0.1x or 0.5x)? Since many ligands and receptors are lowly expressed, is there a risk of losing meaningful biology? Since the dataset has only 293 spots, thresholds can have strong effect.<br><br>Response: We thank the reviewer for this comment. Applying expression-based filtering is standard practice in spatial transcriptomics to reduce spurious findings driven by extremely low counts. For example, the MERINGUE pipeline (link) uses a minimum total-read threshold (e.g., min.reads = 100). We emphasize that our filtering is based solely on the total read count per gene and does not depend on the number of spots in which the gene is detected (i.e., we do not exclude genes simply because they are expressed in fewer than a specified number of spots). We agree with the reviewer's general point that, in highly sparse settings, the method may have difficulty reliably estimating a large number of parameters.<br>Following the reviewer's suggestion, we re-ran the analysis using a more permissive (0.1×) filtering threshold. This yielded 130 additional ligand–receptor pairs in the melanoma dataset (291 pairs in total), of which only 19 were significant. Because our method is applied to each pair independently, all previously identified pairs remained detected.<br>References<br>[1]S. Acharyya, X. Zhou, and V. Baladandayuthapani. SpaceX: gene co-expression

Powered by Editorial Manager® and ProduXion Manager® from Aries Systems Corporation

	network estimation for spatial transcriptomics. Bioinformatics, 38(22):5033–5041, 2022. PMCID: PMC9665869.<br>[2]A. Chakrabarti, Y. Ni, and B. K. Mallick. Joint Bayesian estimation of cell dependence and gene associations in spatially resolved transcriptomic data. Scientific Reports, 14(1):9516, 2024.<br>[3]S. C. Anderson, E. J. Ward, P. A. English, and L. A. Barnett. sdmTMB: an R package for fast, flexible, and user-friendly generalized linear mixed effects models with spatial and spatiotemporal random fields. BioRxiv, pages 2022–03, 2022.<br>[4]J. Geweke. Evaluating the accuracy of sampling-based approaches to the calculation of posterior moments. Technical report, Federal Reserve Bank of Minneapolis, 1991.<br>[5]M. Plummer, N. Best, K. Cowles, and K. Vines. Package 'coda'. URL http://cran. r-project. org/web/packages/coda/coda. pdf, accessed January, 25:2015, 2015.<br>[6]S. Banerjee, B. P. Carlin, and A. E. Gelfand. Hierarchical modeling and analysis for spatial data.<br>Chapman and Hall/CRC, 2014.<br>[7]A. Datta, S. Banerjee, J. S. Hodges, and L. Gao. Spatial disease mapping using directed acyclic graph auto-regressive (DAGAR) models. Bayesian analysis, 14(4):1221, 2019. PMCID: PMC8046356.<br>[8]Z. T. Luo, H. Sang, and B. Mallick. A Bayesian contiguous partitioning method for learning clustered latent variables. Journal of Machine Learning Research, 22(37):1–52, 2021.<br>[9]R. Furrer and S. R. Sain. spam: A sparse matrix R package with emphasis on MCMC methods for<br>Gaussian Markov random fields. Journal of Statistical Software, 36:1–25, 2010.<br>[10]R. J. Lipton, D. J. Rose, and R. E. Tarjan. Generalized nested dissection. SIAM journal on numerical analysis, 16(2):346–358, 1979.<br>[11]S. E. Neville, J. T. Ormerod, and M. Wand. Mean field variational Bayes for continuous sparse signal shrinkage: pitfalls and remedies. Electronic Journal of Statistics, 8:1113–1151, 2014.<br>[12]V. Ravikumar, T. Xu, W. N. Al-Holou, S. Fattahi, and A. Rao. Efficient inference of spatiallyvarying Gaussian Markov random fields with applications in gene regulatory networks. IEEE/ACM transactions on computational biology and bioinformatics, 20(5):2920–2932, 2023.<br>[13]C. M. Carvalho, N. G. Polson, and J. G. Scott. Handling sparsity via the horseshoe. In Artificial intelligence and statistics, pages 73–80. PMLR, 2009.<br>[14]J. Piironen and A. Vehtari. Sparsity information and regularization in the horseshoe and other shrinkage priors. Arxiv, 2017.<br>[15]F. Lindgren, H. Rue, and J. Lindstr¨om. An explicit link between Gaussian fields and Gaussian Markov random fields: the stochastic partial differential equation approach. Journal of the Royal Statistical Society Series B: Statistical Methodology, 73(4):423–498, 2011.<br>[16]D. Makowski, M. S. Ben-Shachar, and D. Lu¨decke. bayestestR: Describing effects and their uncertainty, existence and significance within the Bayesian framework. Journal of open source software, 4(40):1541, 2019.<br>[17]P. E. Bowden. Mutations in a keratin 6 isomer (K6c) cause a type of focal palmoplantar keratoderma.<br>Journal of Investigative Dermatology, 130(2):336–338, 2010.
**Additional Information:**	
**Question**	**Response**
Are you submitting this manuscript to a special series or article collection?	No
**Experimental design and statistics**<br><br>Full details of the experimental design and statistical methods used should be given in the Methods section, as detailed in our Minimum Standards Reporting Checklist. Information essential to interpreting the	Yes

data presented should be made available in the figure legends.<br><br>Have you included all the information requested in your manuscript?	
**Resources**<br><br>A description of all resources used, including antibodies, cell lines, animals and software tools, with enough information to allow them to be uniquely identified, should be included in the Methods section. Authors are strongly encouraged to cite Research Resource Identifiers (RRIDs) for antibodies, model organisms and tools, where possible.<br><br>Have you included the information requested as detailed in our Minimum Standards Reporting Checklist?	Yes
**Availability of data and materials**<br><br>All datasets and code on which the conclusions of the paper rely must be either included in your submission or deposited in publicly available repositories (where available and ethically appropriate), referencing such data using a unique identifier in the references and in the "Availability of Data and Materials" section of your manuscript.<br><br>Have you have met the above requirement as detailed in our Minimum Standards Reporting Checklist?	Yes
GigaScience has policies and guidelines in place for the use of generative AI-writing tools such as ChatGPT. If you have used such writing tools to assist with writing the manuscript this must be declared and cited in the text. Authors should not list AI-writing tools and other AI-assisted technologies as an author or co-author and should acknowledge that they are fully responsible for text	No

generated or refined by AI-writing tools.<p><br><br>A summary of use (particularly in the introduction or among methods) needs to be included at the end of the paper, and the outputs should also be included as a supplementary file hosted in GigaDB or other open repositories. Please read our guidelines for more information. <p><br><br>By submitting to GigaScience, you are aware of the journal's AI-writing tools policy, and if you have declared use of such tools below, you have acknowledged this where appropriate in your manuscript and have made a summary of use and outputs available. <p><br><b>AI-assisted writing tools have been used in the preparation of this manuscript?	

Manuscript

Click here to access/download;Manuscript;SpaceBF_main_edited.pdf

SpaceBF: Spatial coexpression analysis using Bayesian Fused approaches in spatial omics datasets

Souvik Seal and Brian Neelon

Department of Public Health Sciences, College of Medicine, Medical University of South Carolina, Charleston, USA

November 2025

Abstract

Advances in spatial omics enable measurement of genes (spatial transcriptomics) and peptides, lipids, or N-glycans (mass spectrometry imaging) across thousands of locations within a tissue. While detecting spatially variable molecules is a well-studied problem, robust methods for identifying *spatially varying co-expression* between molecule pairs remain limited. We introduce SpaceBF, a Bayesian fused modeling framework that estimates co-expression at both local (location-specific) and global (tissue-wide) levels. SpaceBF enforces spatial smoothness via a fused horseshoe prior on the edges of a predefined spatial adjacency graph, allowing large, edge-specific differences to escape shrinkage while preserving overall structure. In extensive simulations, SpaceBF achieves higher specificity and power than commonly used methods that leverage geospatial metrics, including bivariate Moran's I and Lee's L. We also benchmark the proposed prior against standard alternatives, such as intrinsic conditional autoregressive (ICAR) and Matérn priors. Applied to spatial transcriptomics and proteomics datasets, SpaceBF reveals cancer-relevant molecular interactions and patterns of cell–cell communication (e.g., ligand–receptor signaling), demonstrating its utility for principled, uncertainty-aware co-expression analysis of spatial omics data.

Keywords: Spatial co-expression, CCC, Bayesian fusion, Horseshoe prior, Bivariate association, GMRF

1 Introduction

Technological advances in spatial omics [1–3] have enabled *in situ* profiling of varying molecules, including genes (via spatial transcriptomics (ST)) [4–7], lipids or peptides (using mass spectrometry imaging (MSI)) [8–11], and immune proteins (through multiplex immunofluorescence (mIF)) [12–15], within tissues. The technologies offer distinct yet complementary biological insights, differing in spatial resolution and the number of detectable molecules (throughput). For example, the next-generation sequencing (NGS)-based ST platform Visium (from 10X Genomics) [16] offers transcriptome-wide gene-expression profiling (throughput $\sim 20,000$) at a 55 μm spot-level resolution. MALDI MSI-based platforms (from Bruker Daltonics [17] and others) offer profiling different types of molecules, such as peptides, lipids, nucleotides, proteins, metabolites, and N-glycans, (throughput $\sim 50 - 1000$) at 10 μm spot-level resolution. The mIF platform PhenoCycler (from Akoya Biosciences) [18] enables protein profiling (throughput ~ 40) at a 0.6 μm cellular resolution. Despite these differences, the underlying data structure remains largely consistent across technologies and platforms, comprising a collection of spatial locations (from single or multiple samples) with observed expression or intensity of various molecules. Consequently, common biostatistical questions arise, centering the spatial dynamics of molecules within the complex tissue or tumor microenvironment (TME) [19–23].

In the context of ST datasets, identifying spatially variable genes (SVGs), i.e., the genes exhibiting spatially structured expression patterns across the tissue, has gained significant attention [24–38]. It enables critical downstream analyses such as discovering potential biomarkers and defining tissue regions that influence cellular differentiation and function [39–42]. Analogously, for mIF or imaging mass cytometry (IMC) datasets, innovative methods [43–51] have been proposed to understand the spatial distribution of immune cell types (defined by binarizing the expression profile of immune proteins) across the TME. Building upon this univariate framework, which typically analyzes one molecule at a time, another widely investigated problem has been spatial domain detection, i.e., deconvolving the tissue into distinct, spatially contiguous neighborhoods based on multivariate gene expression (ST) [52–63] or immune cell type composition (mIF) [64–69]. It aids mapping the molecular and functional landscape of tissues, elucidating disease progression,

and guiding targeted therapies [70–72]. While some of the referenced methods can be adapted for use with MSI datasets, it is important to underscore the lack of sophisticated spatial functionalities of the existing bioinformatics toolboxes [73–76].

While univariate and multivariate spatial analyses have garnered significant attention, a critical intermediate task remains underexplored: bivariate spatial co-expression analysis of molecular pairs at both “local" (spot/cell-specific) and “global" (tissue-wide) levels, aimed at precisely characterizing the spatial interaction or binding pattern of any two molecules throughout the tissue plane. To emphasize the importance of such an analysis, we review the concepts of cell-cell communication (CCC) [77–80]. CCC is a fundamental biological process through which cells exchange information via direct contact or signaling molecules (ligands) binding to receptor molecules present on the same or different cells. It regulates essential biological functions, including tissue development [81] and immune responses [82], and its disruption has been implicated in the onset and progression of cancer [83]. Autocrine, juxtacrine, and paracrine signaling are three major pathways of CCC [84]. In autocrine signaling, ligands released by a cell bind to receptors on the same cell, while in juxtacrine and paracrine signaling, the ligands target adjacent and nearby cells. The study of ligand-receptor interactions (LRI), which involves identifying gene pairs (ligands and receptors) that show coordinated upregulation or downregulation across groups of cells, has become a fundamental approach for inferring CCC from single-cell RNA sequencing (scRNA-seq) datasets [85–93]. However, these approaches are prone to false positive interactions due to the lack of spatial context in scRNA-seq datasets, treating distant cell pairs similarly to nearby ones [94–96], which potentially leads to an overestimation of juxtacrine and paracrine signaling. ST datasets offer a natural avenue for improvement by enabling spatially constrained LRI analysis.

A limited number of tools exist for spatial LRI analysis or, more broadly, for assessing bivariate spatial co-expression of molecules in ST or MSI datasets. It should be emphasized that bivariate co-expression can manifest in two ways: (a) joint over- or under-expression within the same cells (correlation) and (b) joint over- or under-expression in neighboring cells (cross-correlation [97]). Some relevant methods include MERINGUE [98], Giotto [99], SpaGene [100], SpaTalk [101], SpatialDM [102], CellChat V2 [103],

LIANA+ [104], and Copulacci [105]. We skip the approaches that jointly analyze multiple LR pairs [106, 107]. Methods such as MERINGUE, Giotto, and SpaTalk provide only a global summary of spatial co-expression across a tissue, whereas others also offer local (spot/cell-specific) estimates. Let the standardized expression of two genes (m, m') be $X^m(s)$ and $X^{m'}(s)$ at location s for $s \in \{s_1, \ldots, s_n\}$, and $X^m = (X^m(s_1), \ldots, X^m(s_n))^\top$, $X^{m'} = (X^{m'}(s_1), \ldots, X^{m'}(s_n))^\top$. For a global summary of spatial co-expression, MERINGUE and SpatialDM leverage a popular geospatial metric termed the bivariate Moran's I (I_{BV}) [108, 109], interpreted as the Pearson correlation between one variable and the spatial lagged version of the other [110–112]. Mathematically, $I_{BV} \propto (X^m)^\top W X^{m'}$, where $W = [[w_{k_1k_2}]]$ is the spatial weight matrix that controls the spatial lagging. As W, MERINGUE uses a binary adjacency matrix based on the Delaunay triangulation [113] of the spatial locations ($w_{k_1k_2} = 1$ if locations (s_{k_1}, s_{k_2}) are connected, or 0 otherwise). SpatialDM uses a kernel covariance matrix or Gram matrix [114] based on the L^2 distance between locations ($w_{k_1k_2} = k_l(|s_{k_1} - s_{k_2}|^2)$, where k_l is a kernel function with lengthscale parameter l [115]). For local estimates of spatial correlation, SpatialDM considers the bivariate local Moran's I ($I_{BV}^{local}(s)$) based on the local indicators of spatial association (LISA) approach [116]. The LIANA+ toolbox implements SpatialDM and introduces a similar spatially weighted cosine similarity index. Of note, a newer package named Voyager [117] considers Lee's L statistic [110], which has a slightly different formulation than I_{BV}. A critical yet often overlooked aspect of ST data analysis is that gene expression, measured in terms of unique molecular identifier (UMI) count, is inherently a discrete random variable (RV). However, the above methods assume normality upon a variance-stabilizing transformation [118–120], which may obscure true signals and have been widely criticized both within the ST literature [26, 55, 121, 122] and in broader contexts [123–126]. Addressing this issue, Copulacci models a pair of genes as bivariate Poisson-distributed RVs, with their correlation in spatially adjacent cells captured using a Gaussian copula [127]. For inference, these methods typically rely on a permutation test [128].

Bivariate Moran's I (I_{BV}) and Lee's L statistic, as implemented in MERINGUE, SpatialDM, LIANA+, and Voyager, are primarily recommended as exploratory metrics for assessing cross-correlation rather than as rigorous hypothesis testing tools [97, 129], in traditional spatial statistical literature. In simulation studies

(see Section 2.2), we have shown that even when two variables are independently simulated with certain spatial covariance structures, the unmodeled spatial autocorrelation introduces a confounding effect on the bivariate association, leading to significantly inflated Type 1 error rates. A similar issue is well documented, as extensive literature highlights the limitations of using simple Pearson correlation to assess dependencies between two variables in the presence of spatial autocorrelation [130–134]. By extension, since I_{BV} and Lee's L are both fundamentally based on Pearson correlation between spatially lagged variables, they may be susceptible to similar pitfalls. In addition, we show that although the asymptotic mean of I_{BV} tends to 0 under the null hypothesis of independence, its asymptotic variance can be large when the marginal spatial autocorrelation patterns of the two molecules are aligned. Consequently, in real datasets I_{BV} may take spuriously large values even under independence. Further, these spatially weighted association indices, being model-free, are unable to seamlessly adjust for cell-level covariates such as cell type, a limitation also present in Copulacci. As a side note, mapping to the aforementioned CCC pathways, Pearson correlation between ligand and receptor can be interpreted as a proxy for autocrine signaling, while cross-correlation may reflect a combination of juxtacrine and paracrine signaling.

We approach the bivariate spatial co-expression detection as a generalized linear regression problem, modeling a molecule m as the outcome and the other molecule m' as the predictor (see Section 4). For ST datasets, gene expression or UMI count is modeled as an overdispersed negative binomial (NB)-distributed RV [135], while an alternative Gaussian model is considered for continuous cases. The regression coefficients, both intercept ($\beta_0^{mm'}(s)$) and slope ($\beta_1^{mm'}(s)$), are assumed to vary across locations (s) exhibiting spatial dependency. Known as the spatially varying coefficients (SVC) model [136], this framework provides exceptional flexibility and precision in capturing locally changing co-expression patterns through $\beta_1^{mm'}(s)$. A large positive $\beta_1^{mm'}(s)$ suggests strong positive co-expression at location s, i.e., joint up or down-regulation, whereas a large negative value indicates avoidance or repulsion. The average of $\beta_1^{mm'}(s)$'s, $\overline{\beta_1^{mm'}} = \sum_k \beta_1^{mm'}(s_k)/n$, provides a summary of the global co-expression pattern. Similar models have been widely used in fields such as disease mapping [137, 138], econometrics [139, 140], ecological studies [141, 142], and neuroimaging research [143, 144]. In the Bayesian paradigm, the spatial dependency between $\beta_0^{mm'}(s)$'s

and $\beta_1^{mm'}(s)$'s is typically modeled using a conditional autoregressive (CAR) [145–147] or Gaussian process (GP) priors [97, 148, 149]. In contrast, we introduce a locally adaptive spatial Gaussian Markov random field (GMRF) prior [150] based on the concepts of fusion penalties [151–153] and horseshoe prior [154–156], extending a related work in the frequentist setup [157]. Briefly, the prior incorporates the spatial similarity between two adjacent locations, (s_{k_1}, s_{k_2}), by encouraging $|\beta_0^{mm'}(s_{k_1}) - \beta_0^{mm'}(s_{k_2})| \approx 0$ and $|\beta_1^{mm'}(s_{k_1}) - \beta_1^{mm'}(s_{k_2})| \approx 0$. We parameterize spatial adjacency primarily with the minimum spanning tree (MST) [158–160], following Li et al. (2019), which provides cycle-free, globally economical connectivity by minimizing total edge weight. In practice, we find that modestly denser graphs, e.g., k-nearest neighbor with small k, can yield improved performance. We evaluate the proposed method SpaceBF against established approaches under realistic simulation scenarios, demonstrating high specificity and power. For broader applicability, we also benchmark our prior against the standard intrinsic CAR (ICAR) and a stochastic partial differential equation (SPDE)-based Matérn prior [161, 162], where SpaceBF consistently outperforms both alternatives. SpaceBF is applied to three real datasets: a) an ST dataset on cutaneous melanoma [39] for spatial LRI analysis, b) an ST dataset on cutaneous squamous cell carcinoma [163] for keratin-interaction analysis, and c) a spatial proteomics dataset on ductal carcinoma in situ (DCIS) from the Medical University of South Carolina (MUSC) for peptide co-localization analysis.

2 Result

2.1 Real Data Analysis

We use the MST as the spatial adjacency graph for the real datasets in the main text and provide complementary kNN–based results in the Supplementary Material.

2.1.1 Melanoma ST Dataset

We analyzed a cutaneous melanoma dataset [39] from a long-term survivor (10+ years), collected using the ST technology [4], comprising 293 spots, each 100 μm in size and at a 200 μm center-to-center distance.

There are 16,148 genes, forming 1,180 known ligand-receptor (LR) pairs as available from CellChatDB [88]. There are three major pathologist-annotated regions as seen in the histology image (Fig. 1A), collected from Thrane et al. (2018), and 6 major cell types (Fig. 1B) predicted using the RCTD [164] package based on overall gene expression [102]. After filtering out genes with extremely low expression ($< 0.2 \times 293 \approx 59$ reads), 161 LR pairs remain, which were examined using our method SpaceBF, without adjusting for any covariates. To briefly summarize the SpaceBF workflow (see Fig. 9), it first constructs an MST based on the spatial coordinates of the spots (Fig. 1C). Then, for every LR pair: (m', m), it considers Eq. 2 with the receptor expression as $X^m(s_k)$ and the ligand expression as $X^{m'}(s_k)$, and s_k representing a spot. Following parameter estimation via a Markov Chain Monte Carlo (MCMC) procedure, the framework performs two hypothesis tests to assess the significance of spatial co-expression at both global and local levels (see Section 4.3). Using the global test in this dataset, SpaceBF identified 53 LR pairs at a significance level 0.05 (33 at an FDR of 0.1). The estimated slope surface $\beta_1^{mm'}(s_k)$ of different LR pairs exhibits distinct patterns. To highlight these differences, we classify the detected LR pairs into 3 major patterns (Fig. 1E) based on hierarchical clustering [165] of the standardized vector $\boldsymbol{\beta}_1^{mm'*} = (\beta_1^{mm'}(s_1) - \overline{\beta_1^{mm'}}, \ldots, \beta_1^{mm'}(s_n) - \overline{\beta_1^{mm'}})^\top / \sigma_\beta^{mm'}$, where $\overline{\beta_1^{mm'}} = \sum_k \beta_1^{mm'}(s_k)/n$ and $\sigma_\beta^{mm'}$ are the tissue-wide average and the SD of estimated $\beta_1^{mm'}(s_k)$'s, respectively. 20 LR pairs follow pattern 1, while 22 and 11 LR pairs correspond to patterns 2 and 3, respectively. Similarly, the spots are grouped into 4 clusters based on the spot-level vectors of slopes corresponding to the 53 detected LR pairs (Fig. 1D). It is evident that clusters 1 and 3 correspond to the melanoma region, while clusters 2 and 4 loosely correspond to the stroma and lymphoid regions, respectively. Returning to the LR patterns, in Fig. 1F, the LR pairs are arranged sequentially from pattern 1 to 3, highlighting the enrichment of their interaction in three major cell types. For example, $\sum_{k \in \text{B/T cells}} \beta_1^{mm'*}(s_k)$ represents the enrichment within B/T cells relative to the average enrichment $\overline{\beta_1^{mm'}}$ and scaled by the SD. The levels "highest," "medium," and "lowest" indicate the degree of enrichment, with "highest" corresponding to the greatest or most positive enrichment and so on. The majority of LR pairs following pattern 1 exhibit higher or more positive interaction in B/T cells within the lymphoid region (some in CAF cells) and more negative interaction (avoidance or repulsion)

in the melanoma region or cells. Pattern 2 mostly corresponds to LR pairs with the highest enrichment in CAF cells, while pattern 3 clearly corresponds to the pairs with the highest enrichment in melanoma cells. Next, we investigate the biological relevance of the estimated slope surfaces for a selected set of LR pairs. The LR pair (IGF2, IGF1R) [166] corresponds to pattern 1 and demonstrates a negative association overall, with an estimated average slope of $\overline{\beta_1^{mm'}} = -0.212$, and the p-value = 0.024, which is consistent with a visual inspection (Fig. 1G). It could indicate a lack of binding between these genes, which would be a generally favorable factor for the survivor [167]. Setting the insignificant $\beta_1^{mm'*}(s_k)$ values to 0 based on the local test, the negative interaction found in the melanoma region has the highest credibility. The pair (PTPRC, CD22) [168] follows pattern 2, with $\overline{\beta_1^{mm'}} = 0.422$ and p-value of 6.28×10^{-6}. PTPRC, also known as CD45, is a facilitator of T-cell receptor (TCR) and B-cell receptor (BCR) signaling [169], while CD22 is primarily an inhibitor of BCR signaling [170]. Their overall positive co-expression, particularly in the lymphoid region, is likely associated with a balanced B cell regulation, helping to prevent autoimmunity and promoting lymphoid growth in other regions as part of the immune response. The final LR pair we discuss is (SPP1, CD44) [171], which follows pattern 3, exhibiting a highly positive overall co-expression with $\overline{\beta_1^{mm'}} = 0.79$ and p-value of 1.03×10^{-6}. This strong interaction displays a decreasing gradient from the melanoma region to the lymphoid region, which aligns with its known role in dysregulated cytoskeletal remodeling [172], facilitating melanoma cell invasion into surrounding tissues.

2.1.2 cSCC ST Dataset

We analyzed a cutaneous squamous cell carcinoma (cSCC) dataset [163] on a patient sample with a histopathologic subtype of "moderately differentiated" cSCC [173]. The dataset was collected using the ST technology with 621 spots, each of size 110 μm and a center-to-center distance of 150 μm. There are $16,643$ genes of which 45 are keratins (14 after filtering low-count genes, $< 0.2 \times 621 \approx 124$ reads). These keratins can be classified into two types: 1) Type 1, which includes KRT10, KRT14–KRT17, and KRT23, and 2) Type 2, which includes KRT1, KRT2, KRT5, KRT6A, KRT6B, KRT6C, KRT78, and KRT80. The keratins pair together to form intermediate filaments, providing structural support to epithelial cells

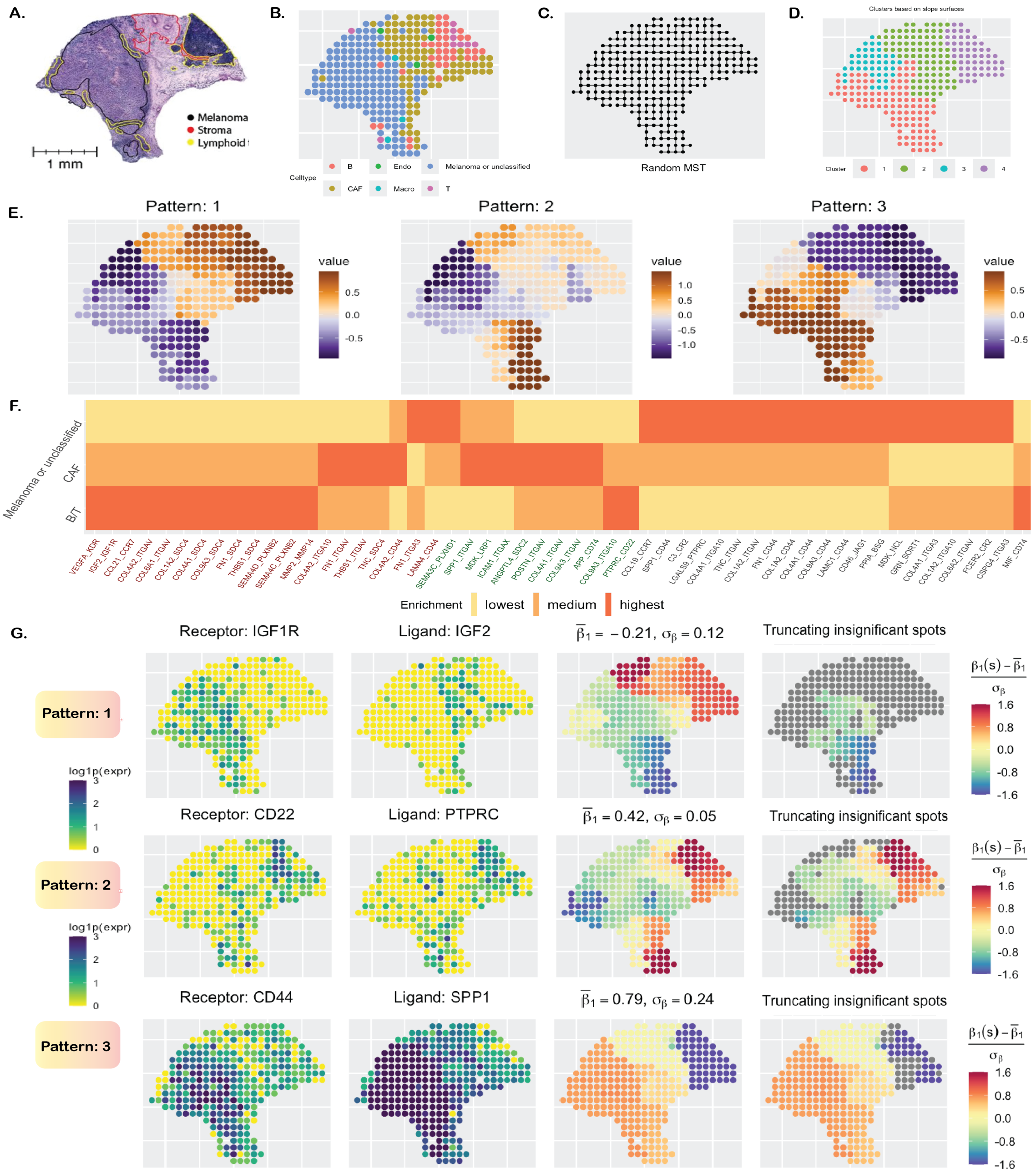

Figure 1: Cutaneous melanoma data analysis. **A**. Annotated H&E-stained image. **B**. Cell types based on gene expression. **C**. Minimum spanning tree (MST) capturing the spatial structure. **D**. Clustering of spots based on centered and scaled estimates of slope surfaces of 53 statistically significant LR pairs. **E**. The three main spatial patterns of the estimated surfaces. **F**. Enrichment of LR interactions in three major cell types, with LR names arranged and color-coded according to their respective patterns. **G**. The first two columns show the expression of three LR pairs. The third column displays the centered and scaled slope surfaces. In the fourth column, insignificant spot-level slope estimates are greyed.

[174]. In the context of cSCC and other carcinomas, keratins are emerging as highly significant targets for therapeutic intervention [175–177]. Of note, some of the keratins belong to the GO term: "keratinocyte differentiation" (GO:0030216) and were reported to exhibit strong spatial correlation in an earlier work [178] involving the same dataset. We utilized SpaceBF to investigate the binding between Type 1 and Type 2 keratins, resulting in a set of 48 keratin pairs. In the histology image (Fig. 2A), the deep blue areas at the top and left sides correspond to tumor regions, while the whitish region at the bottom represents a non-tumor region possibly composed of keratinized layers and stroma [179]. However, the tumor and non-tumor regions are not clearly delineated, a feature characteristic of moderately differentiated cSCC, though the spatial clusters obtained using the BayesSpace package [52] on the transcriptome-wide gene expression profile (Fig. 2C) partially elucidate this distinction. We emphasize that these clusters are shown for visualization only and are not used in our analysis. The constructed MST is shown in Fig. 2B. Using the global test, SpaceBF identified 39 keratin pairs at a significance level of 0.05 (41 at an FDR < 0.1), suggesting that most pairs bind to each other, albeit to varying degrees. Similar to the earlier analysis, we classify the detected slope surfaces into 3 major patterns (Fig. 2D) based on hierarchical clustering of the standardized vector $\boldsymbol{\beta}_1^{mm'*}$. We represent the keratin pairs as bipartite graphs between Type 1 and 2 keratins under each pattern (Fig. 2E). One important observation is that the Type 2 keratins KRT6A, KRT6B, and KRT6C are closely related isoforms of keratin 6 [180] and therefore tend to be strongly co-expressed. Consequently, their spatial association patterns with a given Type 1 keratin are expected to be similar, consistent with the patterns recovered by SpaceBF. For instance, the slope surfaces of KRT10 with KRT6A, 6B, and 6C all align with pattern 1, while the slope surfaces of KRT16 with KRT6A, 6B, and 6C all correspond to pattern 2. This consistency underscores the reliability of SpaceBF in identifying true local patterns. In Fig. 2F, we present the estimated slopes for KRT17, which is a well-established therapeutic target in various cancers [181–183], binding with three type 2 keratins: KRT80 (pattern 1, p-value $= 0.006$), KRT78 (pattern 2, p-value $= 0.03$), and KRT6B (pattern 3, p-value $= 5.59 \times 10^{-6}$). Notably, the average slope estimates for KRT17-KRT80 and KRT17-KRT78 interactions are small (≈ 0.1), whereas for KRT17-KRT6B, the average slope is substantially higher at 0.82, with the highest local estimates observed mostly

in tumor regions. These trends are also evident from the individual expression profiles provided in Fig. 2F. Although the expression patterns of KRT80 and KRT78 appear similar, a closer examination reveals that KRT80 exhibits a thicker band of expression on the left, specifically within the tumor regions. This distinction contributes to the difference in co-expression patterns of KRT17-KRT80 and KRT17-KRT78. As previously noted, both association levels are low, also indicated by the small number of significant spots identified by the local test, 72 and 49, respectively.

2.1.3 DCIS Proteomics Dataset

We analyzed a single-sample ductal carcinoma in situ (DCIS) dataset collected using the MALDI MSI spatial proteomics platform, as part of an ongoing study at the MUSC aimed at defining the proteomic landscape of DCIS and invasive breast cancer (IBC), in terms of collagen peptides and immune cell types. DCIS is marked by the abnormal growth of malignant epithelial cells confined to the breast's milk ducts, without invading the surrounding stromal tissue. While prognosis is excellent, around $20-40\%$ of diagnosed DCIS progress to IBC [184, 185]. Understanding proteomic co-localization within the extracellular matrix (ECM) of a DCIS tissue is crucial for assessing progression risk and predicting therapeutic response, as the ECM plays a key role in regulating tumor cell proliferation, migration, and survival [186]. In this dataset, there are 5,548 tissue spots and 12 ECM peptides, whose $\binom{12}{2}$ pairwise interactions were of our interest. As the peptide expression is continuous-valued, we used the Gaussian model of SpaceBF for this analysis (Eq. 1). Seven of the peptides are derived from the COL1A1 gene, while the remaining peptides originate from COL1A2, COL3A1, and FN1 (Fig. 3C). From the histology image (Fig. 3A), the stromal ECM can be identified by the light pink staining of fibrous connective tissue, while epithelium regions are highlighted in deep blue. Using hierarchical clustering, we group the standardized slope surfaces ($\boldsymbol{\beta}_1^{mm'*}$) into three patterns (Fig. 3B), and the spots into three clusters based on the spot-level vectors of standardized slopes (Fig. 3D). While the differences among the three patterns are subtle, the spot clusters are well-defined and spatially distinct: the red cluster (cluster 3) aligns with stromal regions, the green cluster (cluster 2) with epithelial regions, and the light blue cluster (cluster 1) is a mixture of both. It is important to note

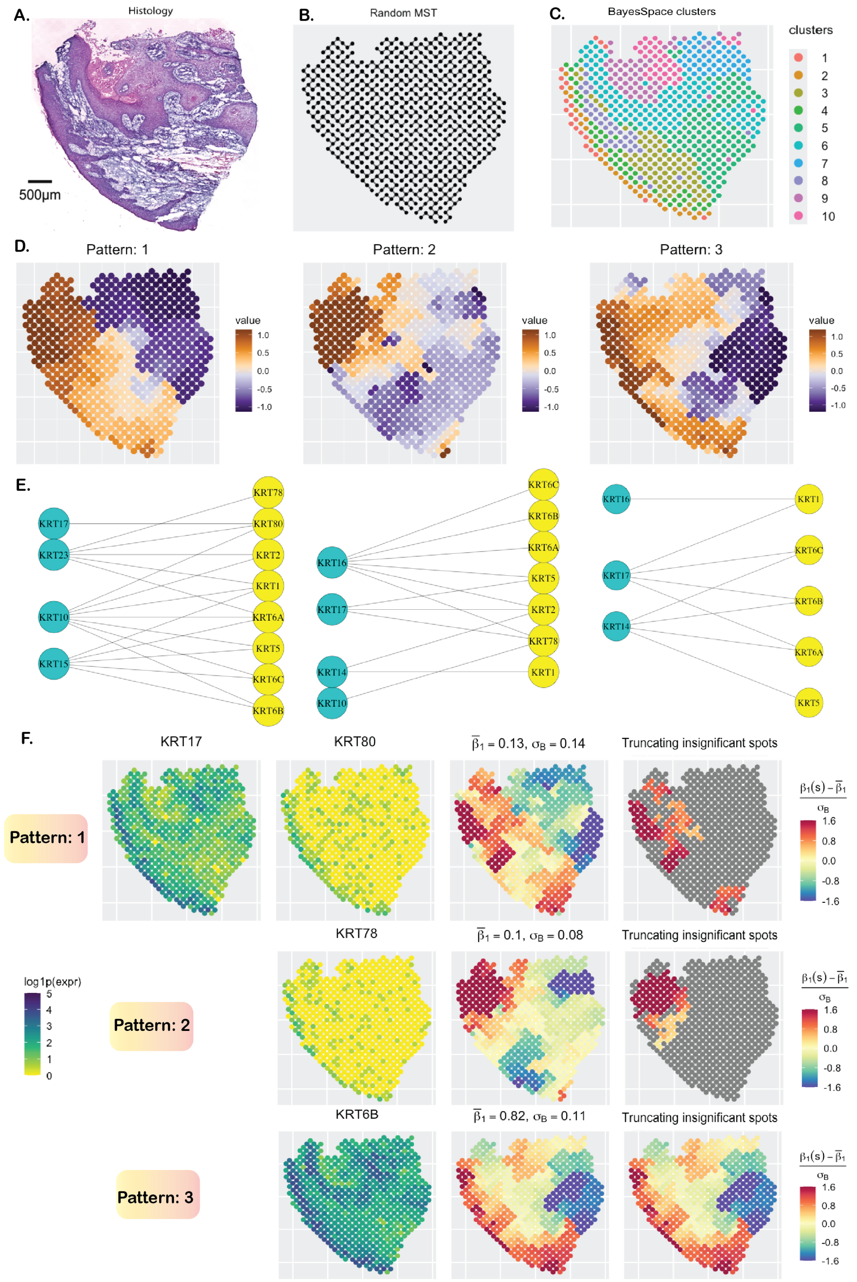

Figure 2: Cutaneous squamous cell carcinoma data analysis. **A**. H&E-stained image. **B**. MST capturing the spatial structure. **C**. Spatial clusters obtained using the BayesSpace package. **D**. The three main spatial patterns of the estimated surfaces. **E**. Bipartite graphs between Type 1 and Type 2 keratins based on their spatial pattern. **F**. Study of the binding between the Type 1 keratin KRT17 and three different type 2 keratins, with each slope surface exhibiting a unique spatial pattern. The insignificant spot-level slope estimates are greyed in the last column.

that the MSI image has substantially lower resolution compared to the histology image, making one-to-one correspondence between the two inherently challenging. Patterns 1 and 2 (Fig. 3B), which visually resemble each other, both suggest strong co-localization of the associated peptide pairs in the stroma. This is expected, as all of these peptides are known to constitute the stromal ECM. The tree diagram in Fig. 3C shows the hierarchical relationships between the peptide pairs, with their patterns indicated on the right. The module highlighted by the yellow box includes pairs involving peptide 1125 (from COL1A2) and 7 other peptides. From Figs. 3E and 3F (top row), peptides 1125, 1212, 1386, and 1681 (from the module) show pronounced co-expression in the stromal region. Correspondingly, the estimated slope surfaces (Fig. 3F, bottom row) for the pairs (1125, 1212), (1125, 1386), and (1125, 1681) all fall under pattern 1, but the association strength is notably higher for (1125, 1212): $\overline{\boldsymbol{\beta}_1^{mm'}} = 0.92$, compared to 0.72 and 0.68 for the other two. Although the existing literature on these interactions is limited, the findings will inform future comparative analyses of ECM compositions across DCIS subtypes and stages of progression [187, 188].

2.2 Simulation Studies

2.2.1 Simulation Design 1: Comparison Between Global Methods Under Linear Association

We consider the spatial coordinates ($n = 293$) from the cutaneous melanoma dataset. In simulation design 1, one NB-distributed random variable (RV), $\mathbf{X}^{m'}$ is generated using a Gaussian copula with a spatial covariance matrix H based on an exponential kernel (for varying lengthscale l) and the L^2 distance. Another NB-distributed RV, $\mathbf{X}^m$ is then generated using the NB model from Eq. 2 with a constant slope $\beta_1^{mm'}(s) = \nu$ and $\boldsymbol{\beta}_0^{mm'}$ simulated using a Gaussian process (GP) model [97] with the spatial covariance matrix H. More details on the design are provided in Section 4.4.1. From Fig. 4A, we notice how the structure of H changes as the lengthscale l varies. The off-diagonal elements of H ($H_{k_1k_2}$) can range between 0 and 1. When $l = 0.6$, only the nearest locations (k_1, k_2) exhibit high $H_{k_1k_2}$ with most of the other values being close to 0. In contrast, for $l = 18$, the majority of location pairs have high $H_{k_1k_2}$ (≈ 1), inducing an exceptionally strong spatial autocorrelation in both variables. To visibly understand how ν might affect the relationship between $\mathbf{X}^m$ and $\mathbf{X}^{m'}$, in Fig. 4B, we show the spatial expression of $\mathbf{X}^m$ for the same $\mathbf{X}^{m'}$

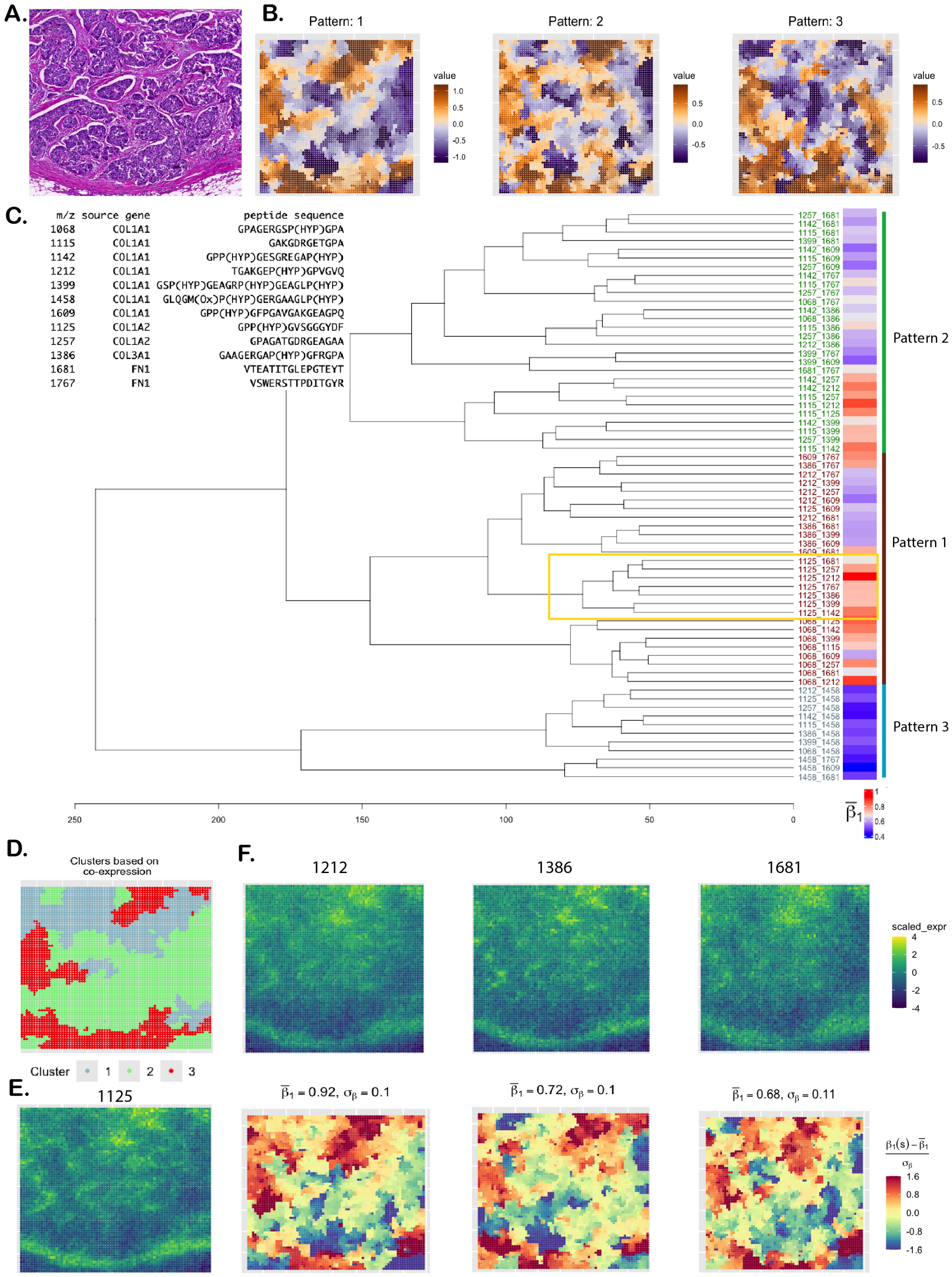

Figure 3: DCIS data analysis. **A**. H&E-stained image. **B**. Patterns of standardized co-expression (slope) of 66 peptide pairs (from 12 peptides). **C**. Peptide description and dendrogram corresponding to the patterns. Mean slope estimates are presented as a heatmap on the right. **D**. Clustering of spots based on the slope surfaces. **E**. Scaled expression of the peptide 1125 forming the yellow-bordered module in the dendrogram. **F**. Scaled expression of three peptides belonging to the same module (top row) and their spatial co-expression with peptide 1125 (bottom row).

but three different values of ν, $\{-0.75, 0, 0.75\}$. It is somewhat evident that nonzero ν's result in a visibly positive or negative association, while $\nu = 0$ produces a random pattern of $\mathbf{X}^m$. In Fig. 4C, we show the Type 1 error ($\nu = 0$) and power ($\nu \neq 0$) comparison of the different methods, including SpaceBF, for three values of the lengthscale l. When $l = 3.6$, both variables exhibit considerable spatial autocorrelation, yet SpaceBF maintains the correct Type 1 error. In contrast, all other methods suffer from inflated Type 1 errors. Notably, simple Pearson correlation, while still inflated, performs better in controlling Type 1 error compared to methods based on bivariate Moran's I or Lee's L. Although SpaGene does not rely on these traditional metrics, it still fails to control Type 1 error. The issue becomes more pronounced as l increases. Notably, Lee's L exhibits the highest inflation in the majority of cases. SpaceBF also retains a high detection power throughout all three cases. Although the power declines slightly for the largest l, as expected, due to a decrease in effective sample size from increased spatial autocorrelation. In summary, the simulation effectively demonstrates the specificity and power of our method.

2.2.2 Simulation Design 2: Comparison Between Global Methods Under Non-linear Association

In simulation design 2, we generate $(\mathbf{X}^m, \mathbf{X}^{m'})$ jointly as bivariate spatially correlated NB-distributed RVs. This setup is more complex than the previous one, as the association is non-linear and driven by the Kronecker product-based spatial covariance structure (see Section 4.4.2). The methods, except SpaceBF, perform poorly in terms of the Type 1 error for $l \geq 1.8$. When the spatial autocorrelation is the weakest ($l = 0.6$), Pearson correlation performs well as expected, while spatially weighted indices still show a slight inflation. SpaceBF achieves controlled Type 1 error and steady detection power across varying l's. As earlier, the power decreases as the effective sample size decreases. Together, these two simulation designs demonstrate SpaceBF's robustness under complex data generation processes. Finally, we argue that incorporating $\beta_0^{mm'}(s)$ in SpaceBF accounts for the spatial autocorrelation of variable m, thereby mitigating bias in the association analysis of (m, m'). As previously noted, Pearson correlation is already recognized to be suboptimal in such scenarios, and spatially weighted indices, essentially Pearson correlation between

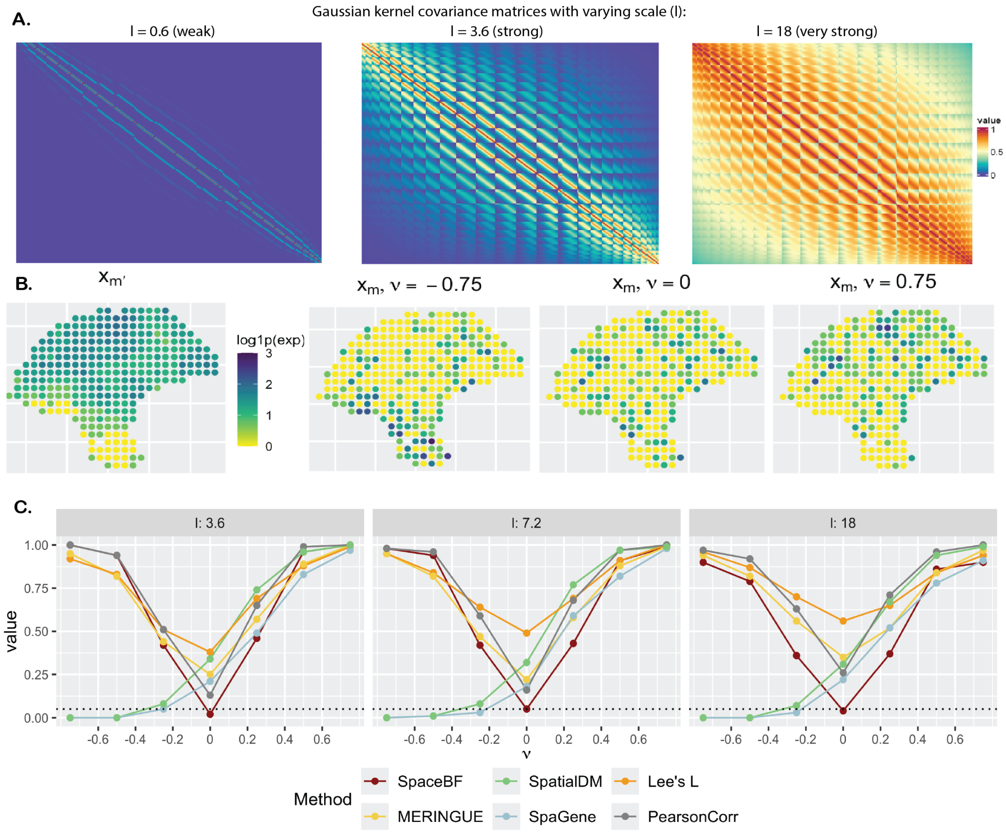

Figure 4: Comparison of global tests under the simulation design 1. **A**. Heatmap of the spatial covariance matrix H with an exponential kernel and L^2 distance, for varying values of the lengthscale parameter l. **B**. Simulated $\mathbf{X}^m$ based on Eq. 2, for a fixed $\mathbf{X}^{m'}$ but different values of the constant slope $\beta_1^{mm'}(s) = \nu$. **C**. Performance of the methods in terms of Type 1 error ($\nu = 0$) and power ($\nu \neq 0$). The dotted line represents the significance level 0.05.

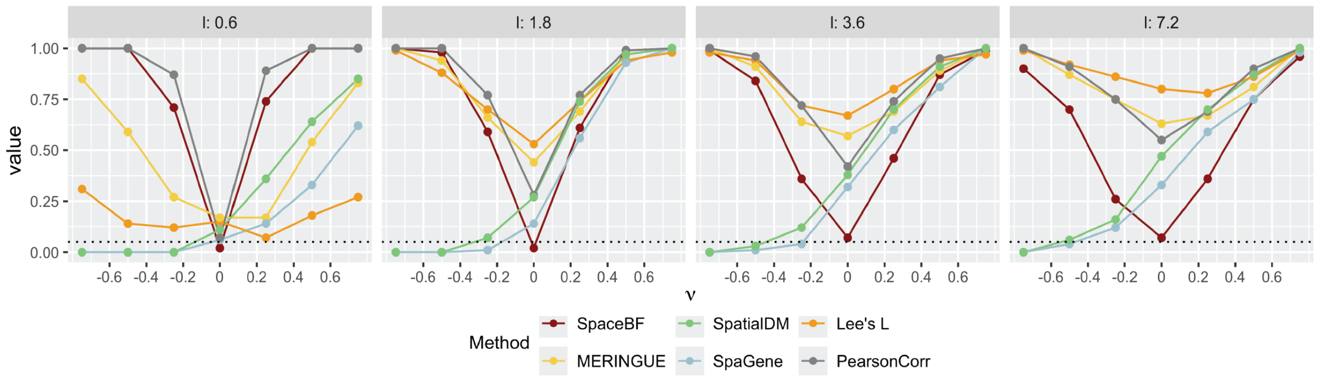

Figure 5: Comparison of global tests under simulation design 2 for lengthscale l between $\{0.6, 1.8, 3.6, 7.2\}$.

spatially lagged variables, thus also remain susceptible to spurious detections. Recall that MERINGUE uses a binary spatial weight matrix W based on the Delaunay triangulation, while SpatialDM uses a continuous spatial weight matrix having a similar form as H for a particular choice of the lengthscale. Lee's L is based on a binary $k-$NN network in our study. Hence, the performance of these methods could be sensitive to choices of the spatial weight matrices, i.e., different networks or lengthscale l values. SpatialDM and SpaGene focus solely on the joint over-expression of molecules, neglecting joint under-expression. As a result, for most values of $\nu < 0$, these methods show almost no detection power.

2.2.3 Simulation Design 3: Comparison Between Spatial Priors Under SVC framework

While the spatial horseshoe (HS) prior is introduced on a minimum spanning tree (MST), it can be placed on any spatial backbone (e.g., Delaunay or kNN graphs), albeit with a potential risk of oversmoothing. This simulation study evaluates how graph choice affects HS performance. A Delaunay network is substantially denser than an MST, whereas a kNN network can serve as a middle ground for small k. In Fig. 6, HS-MST denotes HS on the MST (the original SpaceBF setting used in previous simulations and applications), HS-Del denotes HS on the Delaunay graph, and HS-kNN denotes HS on a kNN graph with $k = 3$. As noted in the Methods section, the ICAR prior is a special case of the HS prior; we therefore include ICAR-Del and ICAR-kNN for comparison. For completeness, we also consider a stochastic partial differential equation (SPDE) [161]-based NB SVC model implemented in the efficient `R` package `sdmTMB` [162], which uses a Matérn prior: sdmTMB-Matérn1 uses a denser mesh (cutoff = 1), and sdmTMB-Matérn2 uses a coarser mesh (cutoff = 1.5), see the Supplementary Material for a visual comparison.

We first revisit the simulation design 2 to assess global performance under a nonlinear model. As shown in Fig. 6A, all priors perform similarly across lengthscales, with a modest inflation in Type I error for HS-Del and ICAR-Del. This aligns with the oversmoothing tendency of denser backbones, which can spuriously propagate a small local positive association (e.g., confined to one corner of the tissue) across the entire domain. The overall similarity among priors is unsurprising in this particular simulation setting: with co-expression effectively constant over space, the additional flexibility of HS (an adaptive precision matrix) is

not meaningfully exercised. Notably, `sdmTMB`-based methods failed to converge in several scenarios (and are therefore omitted here), especially when the outcome contained a high proportion of zeros. We attribute these failures to the near-vanishing curvature of the NB log-likelihood under heavy zero counts, which potentially destabilizes the Laplace-based frequentist optimization; in such cases, a Bayesian approach via `R-INLA` [189] may be preferable.

Next, we assess each prior's ability to recover *spatially varying* slopes under the linear and circular boundary designs in Section 4.4.3. In Fig. 6B, HS-kNN achieves the lowest mean squared error (MSE) across both linear-boundary settings (both values of r) and for both effect sizes ($\nu = 2$ and $\nu = 4$). The gain is most pronounced at $\nu = 4$, where its MSE is roughly half of the competing methods. By contrast, HS-MST, sdmTMB-Matérn1, and sdmTMB-Matérn2 generally perform the worst. For the circular boundary (Fig. 7C), HS-Del and HS-kNN perform similarly and outperform the remaining approaches. Overall, the MSE results favor the HS prior over standard ICAR models on generic spatial graphs and over the Matérn (SPDE-based) alternative. Because HS-MST is consistently outperformed by HS-kNN, we recommend using a denser graph such as kNN in typical applications. Moreover, Figs. 6C and 7C show that HS-kNN best preserves sharp slope changes at boundaries, whereas ICAR methods (especially ICAR-Del) and sdmTMB methods (notably sdmTMB-Matérn2 with a coarser mesh) tend to blur these transitions, highlighting the HS prior's ability to capture subtle co-expression shifts in the TME. Finally, while HS-Del can occasionally perform well, it may oversmooth and produce spurious detections, cautioning against the use of overly dense graphs such as Delaunay.

3 Discussion

We have developed a rigorous framework for studying spatial co-expression of a pair of molecules in the context of spatial transcriptomics (ST) and mass spectrometry imaging (MSI) datasets, at both global (tissue-wide) and local (cell/spot-specific) levels. Existing tools mostly rely on two exploratory geospatial metrics, namely, bivariate Moran's I [108] and Lee's L [110], which lead to highly spurious association inference as demonstrated by our simulation studies. Our proposed approach, SpaceBF, builds on the

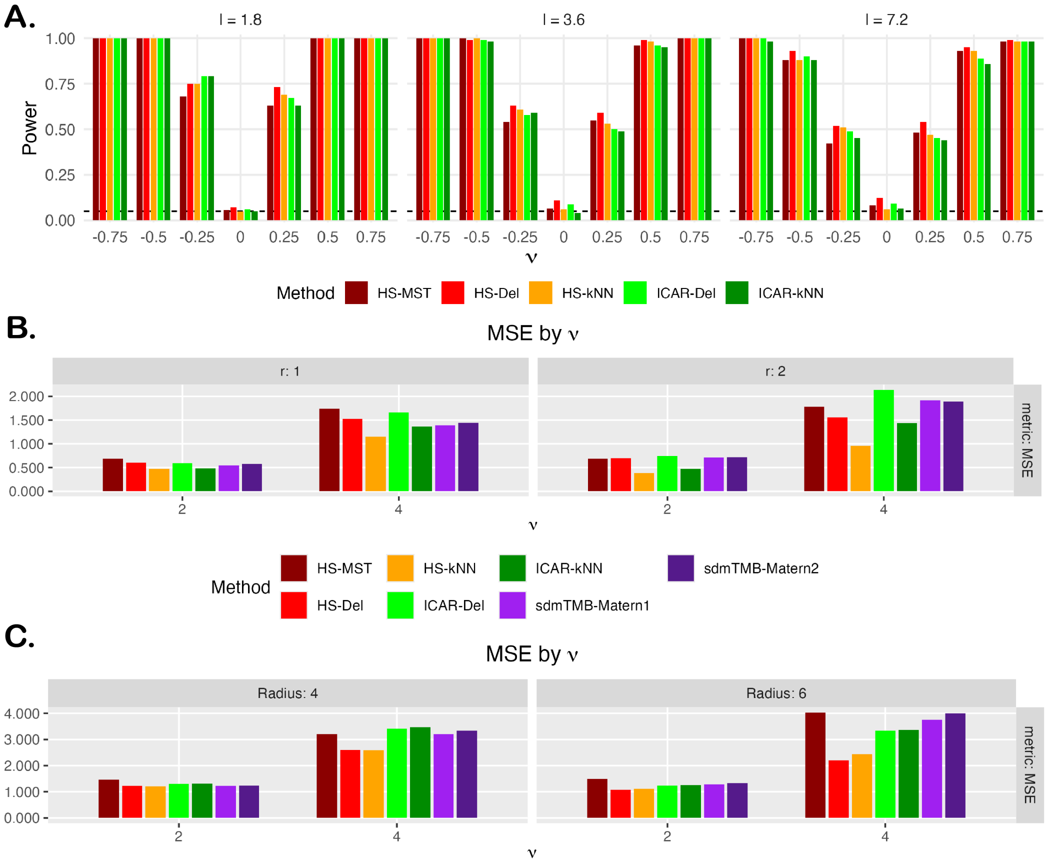

Figure 6: **A**. Power comparison of spatial priors under simulation design 2 for lengthscale l between $\{1.8, 3.6, 7.2\}$. **B**. MSE comparison of spatial priors under simulation design 3, linear partition boundary. **C**. MSE comparison of spatial priors under simulation design 3, circular boundary. In panel A, sdmTMB models are omitted due to recurrent convergence issues.

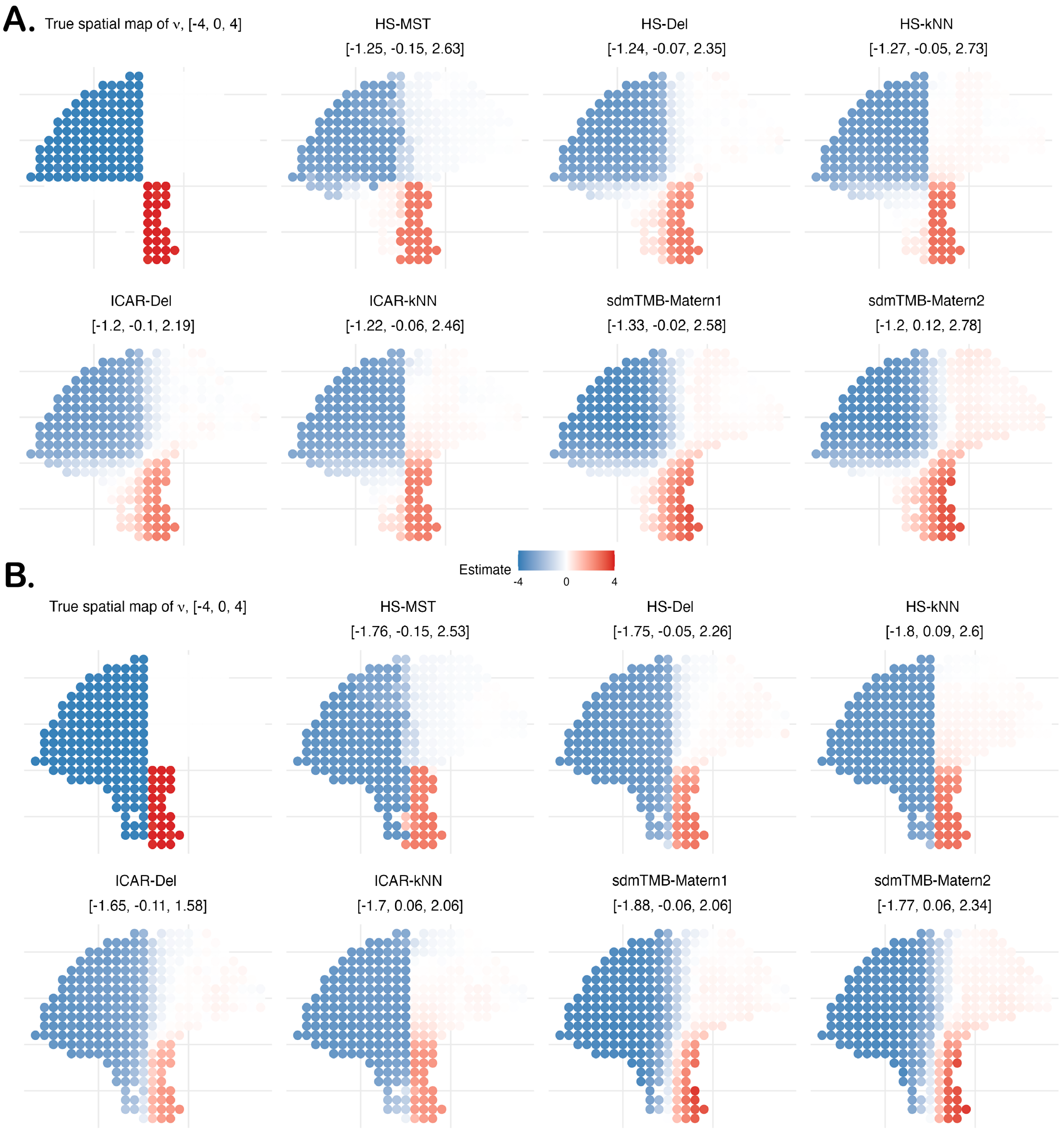

Figure 7: SVC simulation based on linear partition boundary from Section 4.4.3 with effect size $\nu = 4$. The boundary pattern changes based on the parameter r: **A**. $r = 1$, more zeros on the lower left, and **B**. $r = 2$, more negative slope values on the lower left.

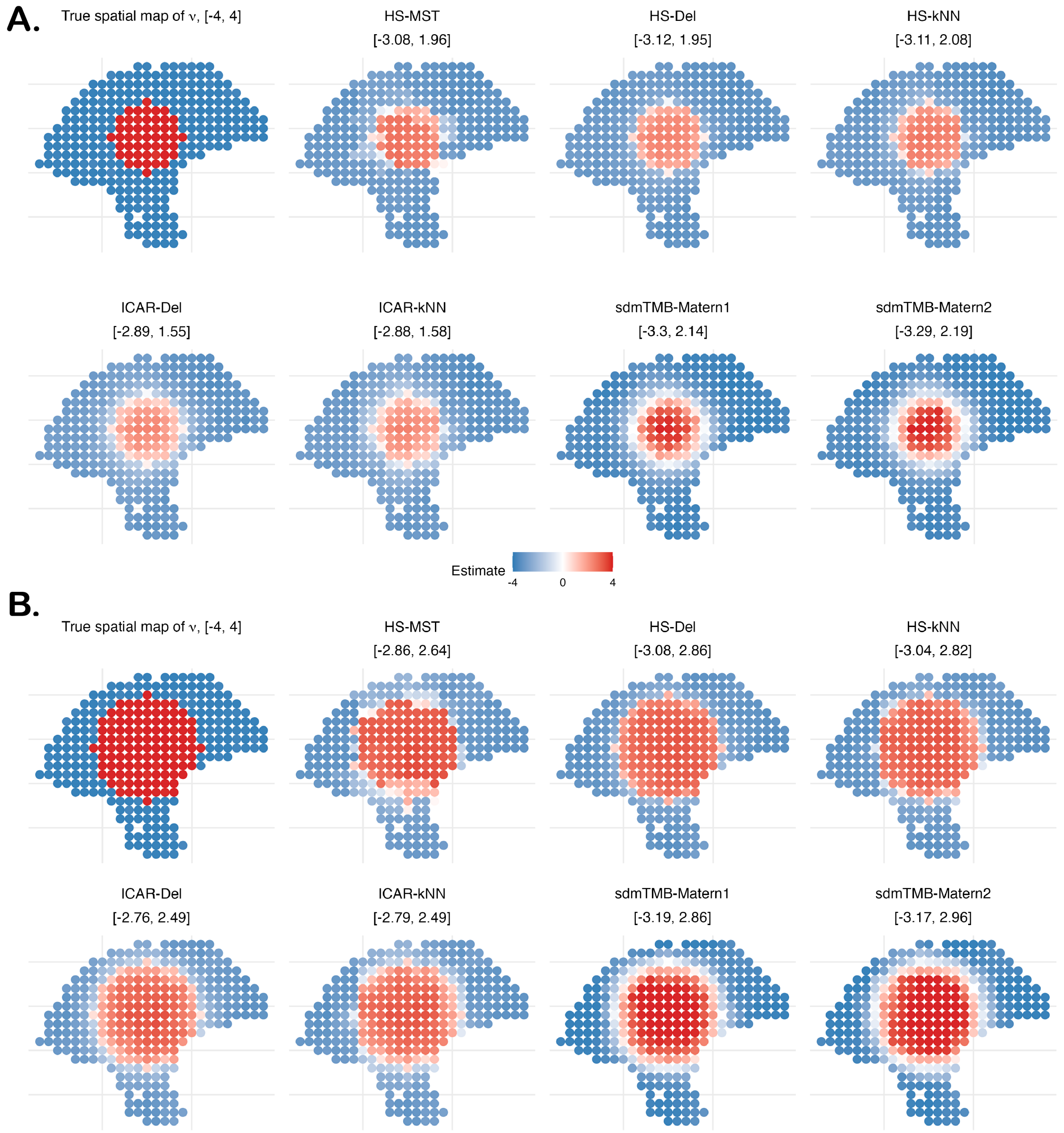

Figure 8: SVC simulation based on circular boundary from Section 4.4.3 with effect size $\nu = 4$. **A**. A smaller circle with positive ν values, radius $r = 4$, and **B**. A larger circle with positive ν values, $r = 6$.

widely used spatially varying coefficients model [136], effectively capturing spatial autocorrelation and locally varying co-expression patterns. We introduce a spatial GMRF prior inspired by the fused horseshoe [156], with a global scale that regulates smoothness along graph edges and edge-specific local scales that allow large discontinuities to escape shrinkage. Setting the local scales to unity recovers the standard ICAR prior. We elucidate the prior's theoretical properties and evaluate its performance against alternative spatial priors. Integrating the prior both within a Gaussian linear regression model and a more complex negative binomial regression framework [135], SpaceBF is broadly applicable across various analytical contexts and data types.

We conduct a comprehensive evaluation of the proposed method under challenging simulation scenarios, demonstrating its ability to maintain well-controlled Type 1 error rates alongside strong detection power. In three real-world applications, two spatial transcriptomics (ST) datasets and one mass spectrometry imaging (MSI) dataset, the method exhibits robust performance in identifying biologically meaningful molecular interactions, including ligand-receptor (LR) signaling, keratin binding, and peptide co-localization. Notably, the analysis of the cutaneous melanoma sample reveals spatially variable patterns of cell-cell communication, as assessed through LR interactions. The LR pairs exhibit coordinated over- or under-expression within spatially distinct tissue regions (identified from histology) or specific cell types (inferred from transcriptome-wide gene expression). This level of granular understanding may provide critical insights for developing novel, targeted tissue-specific therapies in broader clinical settings [190–192].

Using the MST as the spatial graph offers several benefits: (i) uniqueness, removing the need to tune additional graph hyperparameters (e.g., GP lengthscales [97]); (ii) reduced computational burden via an exceptionally sparse precision matrix; and (iii) exact Gibbs updates for local horseshoe scales. In our simulations with spatial autocorrelation generated from a Gaussian process with an exponential kernel and varying lengthscales (but a domain-constant slope), the MST performs well, underscoring its robustness. Nonetheless, restricting the spatial structure to a single spanning tree can exclude salient edges [193], yielding noisier local slope estimates and overly sharp transition boundaries when coefficients vary spatially. In practice, a moderately denser graph, such as a kNN network with a small k, often achieves a better

trade-off between computational efficiency and appropriate smoothness, as observed in our simulations. A more principled avenue could be to treat the spanning tree as unknown and update it iteratively within the model [194]. While we leverage the `spam` package [195] for fast sparse Cholesky factorization, overall complexity is graph-structure dependent (e.g., near $O(n)$ on trees/MSTs and typically around $O(n^{3/2})$ time for 2D planar/kNN graphs) [196]. As future work, we will pursue MCMC-free, variational-inference-based estimation to improve scalability [197, 198]. We have focused on pairwise analyses thus far; extending to joint modeling will follow prior works [199, 200]. Although we have primarily used SpaceBF in ST and MSI datasets, it could also be useful in multiplex immunofluorescence (mIF) or imaging mass cytometry datasets where the molecular outcome of interest is generally immune cell types. To study cell type co-localization in such cases, one could split an mIF image into regular grids and count how many cells of two types (m, m') fall into each grid. Assuming that the grid centers are the locations s_k's, a spatial graph can be constructed, and the spatial cell counts $X^m(s_k)$ and $X^{m'}(s_k)$ can be analyzed using our framework.

4 Methods

4.1 Gaussian and Negative Binomial Regression Models

We assume a single sample or image with n spots/cells. Let $X^m(s_k)$ and $X^{m'}(s_k)$ denote the expression of a pair of molecules m and m', and $C(s_k)$ be a vector of p covariates observed at spot/cell location s_k, for $k \in \{1, \ldots, n\}$. For example, $C(s_k)$ can be the cell Type 1ndicator or a vector of cell type proportions [164]. We consider the following Gaussian spatially varying coefficients (SVC) model [136]

$$X^m(s_k) = \beta_0^{mm'}(s_k) + X^{m'}(s_k)\beta_1^{mm'}(s_k) + C(s_k)^\top \alpha_m + \epsilon(s_k), \;\; k = 1, \ldots, n, \tag{1}$$

where $\beta_0^{mm'}(s_k)$ and $\beta_1^{mm'}(s_k)$ denote spatially varying intercept and slope, respectively, α_m is a fixed effect vector, and $\epsilon(s_k)$ is an independent error term. To interpret the model, a significantly positive $\beta_1^{mm'}(s_k)$ implies that the molecules (m, m') co-express at the location s_k, while a significantly negative value suggests avoidance. Intuitively, $\beta_0^{mm'}(s_k)$ accounts for spatial autocorrelation of molecule m. More discussion on the underlying bivariate spatial process is provided in the Supplementary Material. For a count-valued $X^m(s_k)$

(e.g., genes in the ST datasets), we consider a spatially varying negative binomial (NB) distribution [135] as $NB(\psi_m(s_k), r_m)$ with the failure probability $\psi_m(s_k)$ modeled as [201]

$$\begin{aligned} &\eta_m(s_k) = \beta_0^{mm'}(s_k) + X^{m'}(s_k)\beta_1^{mm'}(s_k) + C(s_k)^\top \alpha_m \\ &p(X^m(s_k)|\psi_m(s_k), r_m) \propto (1-\psi_m(s_k))^{r_m}\psi_m(s_k)^{X^m(s_k)}, \ \ \psi_m(s_k) = \frac{\exp(\eta_m(s_k))}{1+\exp(\eta_m(s_k))}, \end{aligned} \tag{2}$$

where $p(.|.)$ denotes the conditional probability mass function (PMF) and the dispersion parameter $r_m(> 0)$ is assumed to be constant across locations. To explain how this framework effectively models overdispersion: as $r_m \to \infty$, it reduces to a Poisson model; in contrast, as $r_m \to 0$, the counts become increasingly dispersed relative to the Poisson distribution [135]. Admittedly, this model is limited as the count-valued nature of the molecule $X^{m'}(s_k)$ is not prioritized, appearing as a spatially varying predictor. Jointly modeling $(X^m(s_k), X^{m'}(s_k))$ as bivariate NB (BNB) random variables is a possible approach that we do not pursue, as the existing definitions of the BNB distribution (outside of Copula-based constructions) [202–205] vary considerably, often leading to restrictive correlation structures and inefficient MCMC sampling. To clarify, all applications in the manuscript assume no covariates, i.e., we do not include $C(s_k)$ or α_m, for simplicity. The models in Eqs. 1 and 2 are over-parametrized and do not incorporate spatial dependency between $\beta_0^{mm'}(s_k)$'s and $\beta_1^{mm'}(s_k)$'s, which we discuss next. Let $G = (V, E)$ denote the MST network between the locations constructed using the L^2 distance for a pair (s_{k_1}, s_{k_2}), where V and E are the sets of vertices and edges, respectively. Given a connected, weighted graph, an MST is an acyclic subgraph that connects all vertices and minimizes the sum of the weights of the included edges. Because of this property, MST is routinely used to develop transportation and telecommunication networks [206]. For a regular grid, MST is not unique, as the inter-point distances are not distinct. However, a unique random MST can be curated by simply adding small random values to the distances [160, 207]. Our simulation studies indicate that alternative adjacency graphs, such as k-nearest-neighbor (kNN) networks, can yield comparable performance and, in some settings, provide improved results.

4.2 Spatial Modeling

4.2.1 Spatial Fused Lasso

In a recent study [157] of the temperature-salinity relationship in the Atlantic Ocean, Li et al. (2019) consider Eq. 1 and elegantly promote spatial homogeneity of the coefficients by considering fused lasso penalties [151, 208]: $|\beta_0^{mm'}(s_{k_1}) - \beta_0^{mm'}(s_{k_2})| \approx 0$ and $|\beta_1^{mm'}(s_{k_1}) - \beta_1^{mm'}(s_{k_2})| \approx 0$ for $(s_{k_1}, s_{k_2}) \in E$, in a frequentist setup. These constraints are intuitive, as it is reasonable to expect both the degree of co-expression, $\beta_1^{mm'}(s)$, and the effect of "unmeasured" factors, $\beta_0^{mm'}(s)$, to remain homogeneous across adjacent or connected locations. Extending this idea, a Bayesian fused lasso [153, 209] approach can be considered with Laplacian priors on the pair-wise differences of the coefficients. For a pair of locations $(s_{k_i^1}, s_{k_i^2})$ connected by edge $i \in E$, let $\Delta\beta_i^{(j)} \equiv \beta_j^{mm'}(s_{k_i^1}) - \beta_j^{mm'}(s_{k_i^2})$ denote the difference, for $j \in \{0, 1\}$. The fused lasso prior can be imposed as

$$
\begin{aligned}
\pi(\boldsymbol{\beta}_0^{mm'}|\ldots) &\propto \prod_{i\in E} \exp\left(-\frac{\lambda_0}{\sigma}\Delta\beta_i^{(0)}\right), \ \boldsymbol{\beta}_0^{mm'} = (\beta_0^{mm'}(s_1), \ldots, \beta_0^{mm'}(s_n))^\top, \\
\pi(\boldsymbol{\beta}_1^{mm'}|\ldots) &\propto \prod_{i\in E} \exp\left(-\frac{\lambda_1}{\sigma}\Delta\beta_i^{(1)}\right), \ \boldsymbol{\beta}_1^{mm'} = (\beta_1^{mm'}(s_1), \ldots, \beta_1^{mm'}(s_n))^\top,
\end{aligned} \tag{3}
$$

where σ^2 is the variance of the error term $\epsilon(s_k)$, λ_0 and λ_1 are regularization parameters that control the strength of fusion and are assumed to follow gamma priors. Note that σ^2 is only present in the Gaussian model (Eq. 1) and could be omitted from the above exponents. We discuss the resemblance of the prior to the intrinsic CAR (ICAR) prior [146] and, more generally, the intrinsic GMRF (IGMRF) prior [150] in the Supplementary Material. Theoretically, using the L_1 distance seems appealing, as it has the potential to achieve better spatial smoothing by "exactly" fusing coefficient values at adjacent locations, unlike the L^2 distance implied by the ICAR prior. This is analogous to how lasso regression enforces sparsity in solutions, while ridge regression only shrinks effect sizes toward 0 [210]. For transparency, such a spatial fused lasso prior has already been proposed in the existing literature [194, 211].

4.2.2 Spatial Fused Horseshoe

In variable selection problems, failure of the Bayesian lasso or Laplacian prior to achieve exact sparsity, unlike the frequentist analog, has been reported, while also underestimating larger effect sizes [212–214]. Consequently, the Bayesian fused lasso might struggle to promote spatial smoothness and preserve distinct local features simultaneously. For variable selection, the advantages of the horseshoe prior have been convincingly demonstrated to handle unknown sparsity and large outlying signals [154, 215, 216]. The horseshoe prior belongs to the class of global-local shrinkage priors [217], characterized by a "global" hyperparameter that controls overall shrinkage, while "local" hyperparameters control shrinkage per coefficient. Following recent developments on fused horseshoe priors [156, 218], we place horseshoe shrinkage on *edgewise differences* of the spatial coefficients. We specify

$$\begin{aligned} \Delta\beta_i^{(0)} \mid \Lambda_{0i}^2, \tau_0^2, \sigma^2 &\sim N(0,\ \Lambda_{0i}^2\,\tau_0^2\,\sigma^2),\ \ \Lambda_{0i} \sim C^+(0,1),\ \ \tau_0 \sim C^+(0,1), \\ \Delta\beta_i^{(1)} \mid \Lambda_{1i}^2, \tau_1^2, \sigma^2 &\sim N(0,\ \Lambda_{1i}^2\,\tau_1^2\,\sigma^2),\ \ \Lambda_{1i} \sim C^+(0,1),\ \ \tau_1 \sim C^+(0,1), \end{aligned} \tag{4}$$

independently across edges $i = 1, \dots, p$, where $|E| = p$ (e.g., $p = n - 1$ for the MST). The error variance $\sigma^2 = 1$ in the NB model. Let $D \in \mathbb{R}^{p \times n}$ be an oriented incidence matrix and define the weighted Laplacians $L(\Lambda_j) = D^\top \mathrm{diag}(\Lambda_j^{-2})\, D$ with $\Lambda_j^{-2} = \{\Lambda_{ji}^{-2}\}_{i=1}^p$ being the vector of edgewise precisions. The above construction induces the following intrinsic GMRF prior

$$\begin{aligned} \pi(\boldsymbol{\beta}_j^{mm'} \mid \Lambda_j, \tau_j^2, \sigma^2) \ &\propto\ (\tau_j^2\sigma^2)^{-\ell_{\text{rank}}/2}\ \exp\left\{ -\frac{1}{2\,\tau_j^2\sigma^2} \sum_{i=1}^{p} \frac{(\Delta\beta_i^{(j)})^2}{\Lambda_{ji}^2} \right\} \\ &=\ (\tau_j^2\sigma^2)^{-\ell_{\text{rank}}/2}\ \exp\left\{ -\frac{1}{2\,\tau_j^2\sigma^2}\, \boldsymbol{\beta}_j^{mm'\top} L(\Lambda_j) \boldsymbol{\beta}_j^{mm'} \right\}, \end{aligned} \tag{5}$$

where $\ell_{\text{rank}} = \text{rank}\ (L(\Lambda_j)) = n - C$ where C is the number of connected components of the graph ($\ell_{\text{rank}} = n - 1$ for a connected graph). When all local scales are set to one, $\Lambda_{ji} \equiv 1$, the prior reduces to the standard ICAR prior (up to a scale factor) [97]. The deliberately omitted normalizing factor [150] (the generalized determinant of $L(\Lambda_j)$) depends on Λ_j but not on $\boldsymbol{\beta}_j^{mm'}$ or τ_j^2; it therefore plays no direct role in the Gibbs updates for $\boldsymbol{\beta}_j^{mm'}$ or τ_j^2. In our implementation, we update the local scales Λ_{ji} with conjugate per-edge steps, which are exact on trees and constitute a composite or pseudo-likelihood approximation on general graphs [219–221]. The fused horseshoe's half-Cauchy global and local scales produce heavy tails (allowing large

jumps across edges) and an infinitely tall spike at zero (aggressively shrinking small differences), thereby preserving spatial homogeneity while still accommodating sharp local variations. This connection between locally adaptive fusion and GMRFs was emphasized by Faulkner and Minin (2017) [222] in a longitudinal context, encouraging fusion between coefficients across time points. In the Supplementary Material, we provide the details of the Gibbs sampling steps, which include the Pólya-Gamma data augmentation strategy [201, 223] for the NB model (Eq. 2).

One crucial aspect that deserves elucidation is the working assumption of independence between edge-wise differences. Specifically, for any two edges i, i', $\beta_1^{mm'}(s_{k_i^1}) - \beta_1^{mm'}(s_{k_i^2})$ and $\beta_1^{mm'}(s_{k_{i'}^1}) - \beta_1^{mm'}(s_{k_{i'}^2})$ are assumed to be independent in Eq. 4, conditional on the hyperparameters. To see how this assumption could be problematic for a general graph with cycles (i.e., not the MST), we briefly highlight one example from Rue and Held (2005) [150] provided in the context of IGMRF priors. Suppose there are only three locations A, B, and C, all neighbors of each other. Letting $e_1 = \beta_1^{mm'}(A) - \beta_1^{mm'}(B)$, $e_2 = \beta_1^{mm'}(B) - \beta_1^{mm'}(C)$, and $e_3 = \beta_1^{mm'}(C) - \beta_1^{mm'}(A)$, Eq. 4 proceeds to assume e_1, e_2, e_3 are independent and normally distributed with non-identical parameters, yet there is a "hidden" linear constraint, $e_1 + e_2 + e_3 = 0$, that contradicts independence. Analogously, using a highly connected or dense spatial neighborhood graph G introduces numerous hidden constraints corresponding to the cycles in G. Interestingly, as shown in Theorem 3 of the Supplementary Material, these constraints need not be enforced explicitly: the posterior sampling distribution of $\boldsymbol{\beta}^0$ and $\boldsymbol{\beta}^1$ under the constraints is unchanged. However, penalizing too many edge-wise differences in the presence of implicit dependencies can lead to oversmoothing and the loss of salient local structure. This consideration naturally favors the MST, which is acyclic (hence no hidden constraints) and removes redundant relationships; moreover, a sparser G yields faster Cholesky factorizations of the precision matrix and thus improved computational efficiency. That said, as we show in Section 4.4.3, a kNN graph with a small k can significantly outperform the MST in practice.

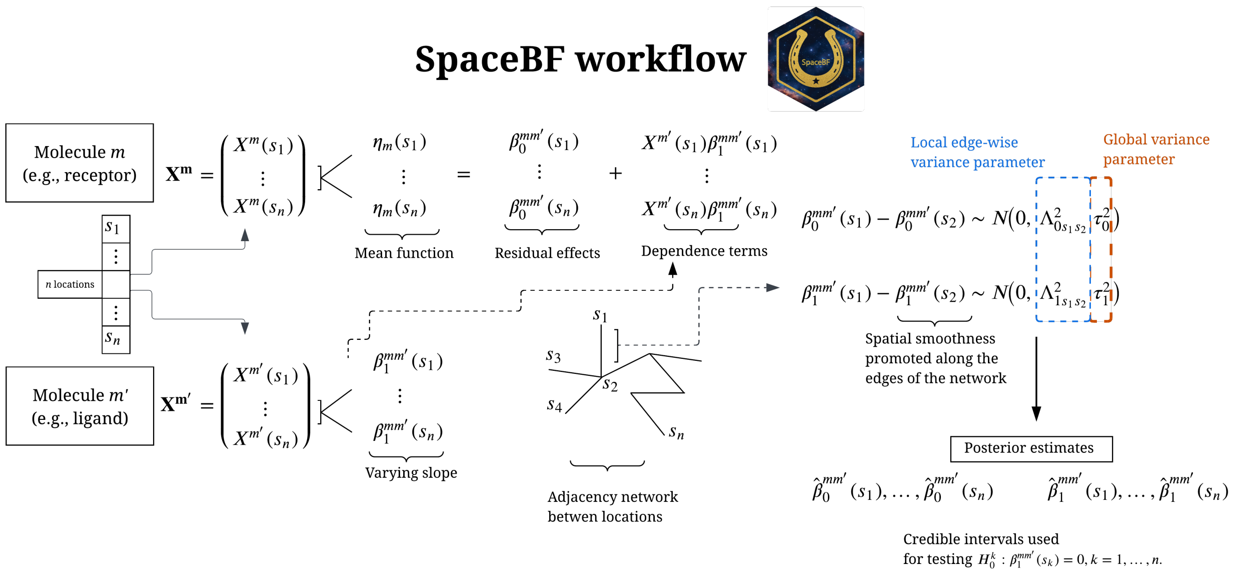

Figure 9: Graphical summary of the proposed approach.

4.3 Hypothesis Testing

We consider two types of hypothesis tests: 1) global test: to determine the significance of average association across the entire tissue domain $\left(H_0 : \overline{\beta_1^{mm'}} = \frac{1}{n}\sum_{k=1}^{n} \beta_1^{mm'}(s_k) = 0\right)$, based on the credible interval [224] of $\overline{\beta_1^{mm'}}$, and 2) local test: to determine the significance of location-level association $\left(H_0^k : \beta_1^{mm'}(s_k) = 0\right)$, directly based on the credible intervals of $\beta_1^{mm'}(s_k)$'s. Additionally, in the genomic context, having a measure analogous to the frequentist p-value is often beneficial. To this end, we utilize a metric termed the probability of direction (p_d), which quantifies the probability (between 0.5 and 1) that a parameter has an effect in a specific direction, either positive or negative [225, 226]. Mathematically, it is defined as the proportion of the posterior distribution that shares the same sign as the median. p_d resembles a two-sided frequentist p-value as $p_{\text{two-sided}} = 2(1 - p_d)$. It is implemented in the R package `bayestestR` [225]. For FDR control, we apply the Benjamini–Hochberg procedure using the `p.adjust` function in R.

4.4 Simulation Design

We consider two different simulation designs as outlined below, for assessing the Type 1 error and power of the model proposed in Eq. 2. The locations at which the variables are simulated are the same as the previously discussed cutaneous melanoma dataset ($n = 293$). We have observed that the results remain unaffected when a randomly generated set of locations or other real data-based sets of locations are used.

4.4.1 Simulation Design 1

In the first design, we directly consider the model from Eq. 2 to generate $(\mathbf{X}^m, \mathbf{X}^{m'})$ based on two steps. First, we generate an NB-distributed RV, $\mathbf{X}^{m'}$, using Gaussian copula [127], incorporating spatial dependency between the observations via a kernel covariance matrix H with an exponential kernel and varying lengthscale (l) parameters [114]. Then, based on the simulated $\mathbf{X}^{m'}$, we generate $\mathbf{X}^m$ following Eq. 2 with a fixed slope $\beta_1^{mm'}(s_k) = \nu$. More specifically, for a fixed choice of l, failure probability $\psi_{m'}$, dispersion parameter $r_{m'}$ for variable m', and dispersion parameter r_m for variable m, we consider the following steps

1. Simulate a spatially autocorrelated normal RV of size n using a Gaussian process (GP) model:

$$\mathbf{Z}^{m'} \sim MVN\left(\mathbf{0}, H\right), \;\; H_{k_1 k_2} = \exp\left(-\frac{||s_{k_1} - s_{k_2}||_1}{l}\right), \;\; ||.||_1 \text{ denotes the } L^1 \text{ norm.}$$

2. Transform to a vector of uniform RVs using the standard normal CDF (Φ):

$$\mathbf{U}^{m'} = \Phi(\mathbf{Z}^{m'}),$$

3. Convert to a vector of NB RVs using the inverse CDF of $NB(\psi_{m'}, r_{m'})$, denoted by $F^{-1}_{NB(\psi_{m'}, r_{m'})}$:

$$\mathbf{X}^{m'} = F^{-1}_{NB(\psi_{m'}, r_{m'})}(\mathbf{U}^{m'}),$$

   Each element of the resulting vector $\mathbf{X}^{m'}$ retains the marginal NB distribution, $NB(\psi_{m'}, r_{m'})$, where $\psi_{m'}$ is the failure probability and $r_{m'}$ is the dispersion.

4. Generate the link function to simulate variable m with a fixed slope of $\beta_1^{mm'}(s_k) = \nu$ (2):

$$\boldsymbol{\eta}_m = \boldsymbol{\beta}_0^{mm'} + \nu \log(\mathbf{X}^{m'} + 1), \quad \boldsymbol{\beta}_0^{mm'} \sim MVN\left(\mathbf{0}, 0.5H\right),$$

5. Convert the link vector to failure probabilities $\boldsymbol{\psi}_m$ and simulate $\mathbf{X}^m$ from the NB distribution:

$$\boldsymbol{\psi}_m = \frac{\exp(\boldsymbol{\eta}_m)}{1+\exp(\boldsymbol{\eta}_m)}, \quad \mathbf{X}^m \sim NB(\boldsymbol{\psi}_m, r_m)$$

where r_m is a prefixed dispersion parameter. The k-th element of $\mathbf{X}^m$ follows $NB(\psi_{mk}, r_m)$, where $\boldsymbol{\psi}_m = (\psi_{m1}, \ldots, \psi_{mn})^\top$ and $\boldsymbol{\eta}_m = (\eta_{m1}, \ldots, \eta_{mn})^\top$.

Three values of the lengthscale l are considered, $l = 3.6, 7.2, 18$, with the corresponding structure of H displayed in Fig. 4A. The failure probability of variable m' and dispersion parameters are kept fixed, $\psi_{m'} = 0.5$, $r_m = r_{m'} = 1$. The slope parameter ν is varied between $\{-0.75, -0.5, -0.25, 0, 0.25, 0.5, 0.75\}$, with negative and positive values representing negative and positive association, respectively. Higher absolute value of ν dictates the strength of association, and $\nu = 0$ corresponds to the null model, i.e., $\mathbf{X}^m$ and $\mathbf{X}^{m'}$ are independent.

4.4.2 Simulation Design 2

In this design, $(\mathbf{X}^m, \mathbf{X}^{m'})$ are simulated jointly using a bivariate Gaussian copula and spatial dependency incorporated using a bivariate GP framework, where the joint covariance matrix has a Kronecker product structure, comprising a 2×2 correlation matrix and the distance kernel covariance matrix H (Eq. 9.11 from Banerjee et al. (2014)[97] and Eq. 6 from the Supplementary Material). Specifically, we consider the following steps

1. Simulate spatially cross-correlated normal RVs:

$$(\mathbf{Z}^m, \mathbf{Z}^{m'})^\top \sim MVN\left(\begin{bmatrix}\mathbf{0}\\ \mathbf{0}\end{bmatrix}, \boldsymbol{\Sigma} = \begin{bmatrix}1 & \nu\\ \nu & 1\end{bmatrix} \otimes H\right), \quad H_{k_1k_2} = \exp\left(-\frac{||s_{k_1} - s_{k_2}||_1}{l}\right),$$

2. Transform to uniform RVs using the standard normal CDF (Φ):

$$\mathbf{U}^m = \Phi(\mathbf{Z}^m), \quad \mathbf{U}^{m'} = \Phi(\mathbf{Z}^{m'}),$$

3. Convert to NB random variables using the inverse CDFs:

$$\mathbf{X}^m = F^{-1}_{NB(\psi_m, r_m)}(\mathbf{U}^m), \quad \mathbf{X}^{m'} = F^{-1}_{NB(\psi_{m'}, r_{m'})}(\mathbf{U}^{m'}),$$

Each element of the resulting vectors $\mathbf{X}^m$ and $\mathbf{X}^{m'}$ retain the marginal NB distributions, $NB(\psi_m, r_m)$ and $NB(\psi_{m'}, r_{m'})$, respectively, where $\psi_m, \psi_{m'}$ are failure probabilities and $r_m, r_{m'}$ are dispersions.

The lengthscale l is varied between $\{0.6, 1.8, 3.6, 7.2\}$. The failure probabilities and dispersion parameters are kept fixed, $\psi_m = \psi_{m'} = 0.5$, $r_m = r_{m'} = 1$. The parameter ν is varied between $\{-0.75, -0.5, -0.25, 0, 0.25, 0.5, 0.75\}$, with similar implications on the direction and strength of association as before.

4.4.3 Simulation Design 3

We consider the model from Eq. 2 to generate $(\mathbf{X}^m, \mathbf{X}^{m'})$ based on two steps. First, we generate an NB-distributed RV, $\mathbf{X}^{m'}$, using Gaussian copula [127], incorporating spatial autocorrelation via H with a large lengthscale, $l = 7.2$. Then, based on the simulated $\mathbf{X}^{m'}$, we generate $\mathbf{X}^m$ following Eq. 2, this time with a spatially varying slope $\beta_1^{mm'}(s_k)$. Specifically, we consider the following steps

1. Simulate $\mathbf{X}^{m'}$ following steps 1, 2, and 3 from Section 4.4.1.

2. Simulate the slope $\boldsymbol{\beta}_1^{mm'}$ in two ways: based on a) linear partition boundary and b) circular boundary. Let $c_x = \text{median}(s_k^x)$, $c_y = \text{median}(s_k^y)$ denote the median of xy-coordinates, respectively.

   (a) *Linear partition boundary:* Define two partitioning subsets of the spatial domain as

   $$\mathcal{S}_1 = \{k : \; s_k^x > c_x, \; s_k^y \le c_y\}, \qquad \mathcal{S}_2 = \{k : \; s_k^x \le c_x, \; s_k^y > c_y/r\}.$$

   Draw $\sigma_k \sim \text{Unif}(0.3, 0.6)$. For $k \in \mathcal{S}_1$, draw $\beta_{1k}^{mm'}(s_k) \sim N(\nu, \sigma_k^2)$; for $k \in \mathcal{S}_2$, draw $\beta_{1k}^{mm'}(s_k) \sim N(-\nu, \sigma_k^2)$; for other k's $\beta_{1k}^{mm'}(s_k) = 0$. Vary $r \in \{1, 2\}$ to create two different partitioning configurations.

   (b) *Circular boundary*: Define the indicator for being inside a circle with radius $r \in \{1, 2\}$ and centered at the median

   $$\mathbb{I}_k \;=\; \mathbf{1}\left\{ \frac{(x_k - c_x)^2 + (y_k - c_y)^2}{r^2} \le 1 \right\}.$$

Set $\sigma_k = \sigma_{\text{in}}\, \mathbb{I}_k + \sigma_{\text{out}}\, (1 - \mathbb{I}_k)$ with $\sigma_{\text{in}} = 0.3, \sigma_{\text{out}} = 0.6$, and draw $\varepsilon_k \sim N(0, \sigma_k^2)$. Finally, define

$$\beta_{1k}^{mm'}(s_k) \;=\; \nu\,(2\mathbb{I}_k - 1) \;+\; \varepsilon_k.$$

Thus, $\mathbb{E}[\beta_{1k}^{mm'}(s_k) \mid \mathbb{I}_k = 1] = \nu$ (inside the circle) and $\mathbb{E}[\beta_{1k}^{mm'}(s_k) \mid \mathbb{I}_k = 0] = -\,\nu$ (outside).

3. Generate the link function to simulate variable m with the slope $\boldsymbol{\beta}_1^{mm'}$:

$$\boldsymbol{\eta}_m = \boldsymbol{\beta}_0^{mm'} + \boldsymbol{\beta}_1^{mm'} \log(\mathbf{X}^{m'} + 1), \quad \boldsymbol{\beta}_0^{mm'} \sim MVN\left(\mathbf{0}, 0.5H\right),$$

4. Convert the link vector to failure probabilities $\boldsymbol{\psi}_m$ and simulate $\mathbf{X}^m$ from the NB distribution:

$$\boldsymbol{\psi}_m = \frac{\exp(\boldsymbol{\eta}_m)}{1 + \exp(\boldsymbol{\eta}_m)}, \;\; \mathbf{X}^m \sim NB(\boldsymbol{\psi}_m, r_m)$$

where r_m is a prefixed dispersion parameter. The k-th element of $\mathbf{X}^m$ follows $NB(\psi_{mk}, r_m)$, where $\boldsymbol{\psi}_m = (\psi_{m1}, \ldots, \psi_{mn})^\top$ and $\boldsymbol{\eta}_m = (\eta_{m1}, \ldots, \eta_{mn})^\top$. Each element of the resulting vectors $\mathbf{X}^m$ and $\mathbf{X}^{m'}$ retain the marginal NB distributions, $NB(\psi_m, r_m)$ and $NB(\psi_{m'}, r_{m'})$, respectively, where $\psi_m, \psi_{m'}$ are failure probabilities and $r_m, r_{m'}$ are dispersions.

The dispersion parameters are kept fixed, $r_m = r_{m'} = 1$, and the failure probability $\psi_{m'} = 1$. The boundary parameter (or radius) r is varied between $\{1, 2\}$. The boundary (radius) parameter r varies over $\{1, 2\}$, and the effect-size parameter ν varies over $\{2, 4\}$. Results for $\nu = 2$ are provided in the Supplementary Material, while $\nu = 4$ results are shown in Figs. 7 and 8.

4.5 Competing Methods

We compare **SpaceBF** against five methods: (1) MERINGUE [98] with a Delaunay-triangulation graph; (2) SpatialDM [102] using a Gaussian kernel weight matrix (lengthscale 1.2, as in their original melanoma analysis); (3) Lee's L [110] on an ϵ-neighborhood network (implemented via the R package `spdep` [227]), where ϵ is set to the maximum nearest-neighbor distance to ensure connectivity; (4) SpaGene [100]; and (5) PearsonCorr, the standard Pearson correlation. We do not include LIANA+ [104] or Voyager [117], as the former essentially wraps SpatialDM and the latter directly applies Lee's L. We adopt an ϵ-neighborhood

graph rather than a fixed kNN graph to mitigate oversmoothing in Lee's L while maintaining graph connectivity; however, this modification did not improve performance, as evidenced by our simulation studies. Table 1 summarizes the methods in terms of their assumptions and limitations. Note that, in the Supplementary Material, we derive the asymptotic mean and variance of the bivariate Moran's I statistic and show that even under true independence, marginal spatial autocorrelation in each variable can inflate the estimated value. Moreover, this inflation is most pronounced when the autocorrelation patterns are aligned in the same direction. We also attempted to evaluate the performance of SpatialCorr [178] and Copulacci [105]. SpatialCorr was straightforward to use, but it proved highly sensitive to the choice of the lengthscale l in its innovative use of the spatial covariance matrix H. Copulacci was slightly difficult to use and will be benchmarked in a future study.

Method	Central metric or concept	Global and local co-expression tests	Test type	Potential sensitivity
**SpaceBF**	Spatially varying coefficients model with NB distribution	Global and local	Exact	Spatial adjacency graph
MERINGUE	Bivariate Moran's I	Global	Permutation test	Spatial adjacency graph
SpatialDM	Bivariate Moran's I	Global and local	Permutation or exact test	Lengthscale in the kernel weight matrix
LIANA+	Bivariate Moran's I and cosine similarity	Global and local	Permutation test	As above
Voyager	Lee's L	Global and local	Permutation test	Spatial adjacency graph
SpaGene	kNN network and earth mover's distance	Global but with local interaction scores	Permutation test	Choice of k
PearsonCorr	Pearson correlation	Global	Permutation or exact test	None
SpatialCorr *	Spatial kernel-weighted sample correlation	Global and local	Permutation test	Lengthscale in the kernel weight matrix
Copulacci *	Bivariate Poisson distribution along an adjacency graph	Global but with local interaction scores	Permutation test	Spatial adjacency graph and fixed correlation term across locations

Table 1: Comparison of the methods in terms of the underlying assumptions. *methods that are not evaluated in the simulations.

We further evaluate the performance of SpaceBF (the spatial horseshoe) on general spatial graphs, alongside standard spatial priors within our SVC framework (Sections 2.2.3 and 4.4.3). The configurations considered are summarized in Table 2, with brief notes on their limitations.

Method	Graph	Prior	Limitations
HS-MST	Minimum spanning tree (MST)	Horseshoe (HS)	May under-smooth within dense micro-domains
HS-Del	Delaunay triangulation (Del)	HS	Risk of over-smoothing and spurious association; approximate local scale update
HS-kNN	k-nearest neighbors (k=3)	HS	Sensitive to k: larger k increases density and risk of over-smoothing
ICAR-Del	Delaunay triangulation	ICAR (special case of HS)	Risk of diffusing boundaries on dense graphs; tends to attenuate sharp slope changes.
ICAR-kNN	k-nearest neighbors	ICAR	Similar boundary diffusion; performance depends on k
sdmTMB-Matérn1	Mesh (cutoff = 1; denser)	Matérn (SPDE)	Risk of diffusing boundaries; mesh tuning adds burden, Laplace approximation may not converge
sdmTMB-Matérn2	Mesh (cutoff = 1.5; coarser)	Matérn (SPDE)	Above problems and risk of oversmoothing

Table 2: Methods, graphs, priors, and limitations. HS can be placed on any spatial adjacency graph; denser graphs (e.g., Delaunay or large-k kNN) increase the risk of over-smoothing. HS-Del and HS-kNN (k=3) probe denser and intermediate backbones than HS-MST. ICAR variants are included for comparison. The `sdmTMB` Matérn models differ by mesh density; a coarser mesh leads to a smoother spatial field.

4.6 Runtime Comparison and Convergence Diagnostics

In most analyses we ran 5,000 MCMC iterations with 2,500 burn-in. We compared runtimes for our package `SpaceBF` across priors and spatial backbones (from sparser to denser). Figure 10 shows that HS and ICAR have comparable runtimes, scaling approximately linearly with n. Denser graphs (e.g., k-NN with $k = 9$) are marginally slower. For $n = 5{,}000$, `SpaceBF` completes in about 20 minutes on a Mac Pro (M3 Max). For substantially larger datasets, a practical alternative is to consider `sdmTMB` [162] which fits an NB SVC model via a Laplace-approximate maximum likelihood approach. It is extremely fast but can be less precise, may fail to converge, and often requires tuning the mesh density for interpretable results.

For the convergence diagnostics, we computed the Geweke statistic [228] for each $\beta_1^{mm'}(s_k)$, implemented in the *R* package *coda* [229], and investigated the trace plots of a few randomly chosen $\beta_1^{mm'}(s_k)$'s (see the Supplementary Material). When either the variable m or m' is highly sparse ($> 75\%$ zeroes), imposing ad-

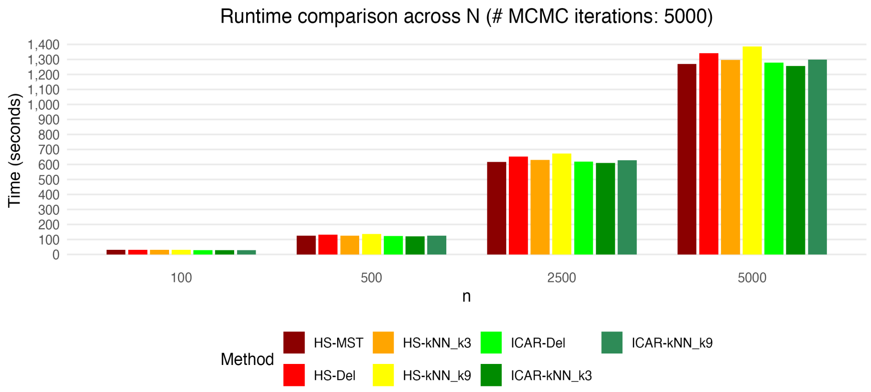

Figure 10: Run-time comparison of `SpaceBF`, with different priors: the horseshoe GMRF and ICAR, with varying spatial adjacency graphs.

ditional normal priors on $\beta_0^{mm'}(s_k)$'s and $\beta_1^{mm'}(s_k)$'s with a moderate variance, such as $N(0, 10)$, drastically improves mixing and overall convergence performance.

5 Data Availability

The melanoma and cSCC datasets are publicly available: 1) cutaneous melanoma [39, 230] with sample ID "`ST_mel1_rep2`", and 2) cSCC [163, 231] with sample ID "`GSM4284236 P6_cSCC_scRNA`". The spatial proteomics dataset is available on Zenodo [232]. The datasets are also provided in .rda format within the GitHub package.

6 Availability of Source Code and Requirements

- Project name: SpaceBF
- Project homepage: https://github.com/sealx017/SpaceBF/
- License: GPL-3.0
- Operating system: tested on macOS, Windows
- Programming language: R

- Package management: GitHub
- Hardware requirements: verified to run on laptops with 10 cores and 64 GB RAM

7 Funding

S.S. and B.N. were supported in part by the Biostatistics Shared Resource, Hollings Cancer Center, Medical University of South Carolina (P30 CA138313). S.S. was supported in part by NIH R21 CA286287-01A1. S.S. was supported by the American Cancer Society Institutional Research Grant: IRG-24-1290553-23-IRG. The content is solely the responsibility of the authors and does not necessarily represent the official views of the American Cancer Society, the National Cancer Institute, and the National Institutes of Health.

8 Acknowledgments

The authors thank Dr. Peggi Angel from the Medical University of South Carolina for her help in the spatial proteomics dataset acquisition and interpretation. S.S. and B.N. contributed equally to the conceptualization and methodology of the project, and jointly wrote the first draft. S.S. conducted the validation, simulation experiments, and software development. The authors do not have any competing interests.

References

[1] Jeffrey R Moffitt, Emma Lundberg, and Holger Heyn. The emerging landscape of spatial profiling technologies. *Nature Reviews Genetics*, 23(12):741–759, 2022. 10.1038/s41576-022-00515-3.

[2] Dario Bressan, Giorgia Battistoni, and Gregory J Hannon. The dawn of spatial omics. *Science*, 381(6657):eabq4964, 2023. 10.1126/science.abq4964.

[3] Katy Vandereyken, Alejandro Sifrim, Bernard Thienpont, and Thierry Voet. Methods and applications for single-cell and spatial multi-omics. *Nature Reviews Genetics*, pages 1–22, 2023. 10.1038/s41576-023-00580-2.

[4] Patrik L Ståhl, Fredrik Salmén, Sanja Vickovic, Anna Lundmark, José Fernández Navarro, Jens Magnusson, Stefania Giacomello, Michaela Asp, Jakub O Westholm, Mikael Huss, et al. Visualization and analysis of gene expression in tissue sections by spatial transcriptomics. *Science*, 353(6294):78–82, 2016. 10.1126/science.aaf2403.

[5] Sheel Shah, Eric Lubeck, Wen Zhou, and Long Cai. seqfish accurately detects transcripts in single cells and reveals robust spatial organization in the hippocampus. *Neuron*, 94(4):752–758, 2017. 10.1016/j.neuron.2017.05.008.

[6] Michaela Asp, Joseph Bergenstråhle, and Joakim Lundeberg. Spatially resolved transcriptomes—next generation tools for tissue exploration. *BioEssays*, 42(10):1900221, 2020. 10.1002/bies.201900221.

[7] Lambda Moses and Lior Pachter. Museum of spatial transcriptomics. *Nature Methods*, 19(5):534–546, 2022. 10.1038/s41592-022-01409-2.

[8] Peggi M Angel, Anand Mehta, Kim Norris-Caneda, and Richard R Drake. Maldi imaging mass spectrometry of n-glycans and tryptic peptides from the same formalin-fixed, paraffin-embedded tissue section. *Tissue proteomics: methods and protocols*, pages 225–241, 2018. 10.1002/cpps.68.

[9] Jeffrey M Spraggins, Katerina V Djambazova, Emilio S Rivera, Lukasz G Migas, Elizabeth K Neumann, Arne Fuetterer, Juergen Suetering, Niels Goedecke, Alice Ly, Raf Van de Plas, et al. High-performance molecular imaging with maldi trapped ion-mobility time-of-flight (timstof) mass spectrometry. *Analytical chemistry*, 91(22):14552–14560, 2019. 10.1021/acs.analchem.9b03612.

[10] Tara R Hawkinson, Harrison A Clarke, Lyndsay EA Young, Lindsey R Conroy, Kia H Markussen, Kayla M Kerch, Lance A Johnson, Peter T Nelson, Chi Wang, Derek B Allison, et al. In situ spatial glycomic imaging of mouse and human alzheimer's disease brains. *Alzheimer's & Dementia*, 18(10):1721–1735, 2022. 10.1002/alz.066433.

[11] Xin Ma and Facundo M Fernández. Advances in mass spectrometry imaging for spatial cancer metabolomics. *Mass spectrometry reviews*, 43(2):235–268, 2024. 10.54254/2753-8818/49/20241266.

[12] Kathrin Heinzmann, Lukas M Carter, Jason S Lewis, and Eric O Aboagye. Multiplexed imaging for diagnosis and therapy. *Nature Biomedical Engineering*, 1(9):697–713, 2017. 10.1038/s41551-017-0131-8.

[13] Erik A Burlingame, Jennifer Eng, Guillaume Thibault, Koei Chin, Joe W Gray, and Young Hwan Chang. Toward reproducible, scalable, and robust data analysis across multiplex tissue imaging platforms. *Cell reports methods*, 1(4):100053, 2021. 10.1016/j.crmeth.2021.100053.

[14] Sabrina M Lewis, Marie-Liesse Asselin-Labat, Quan Nguyen, Jean Berthelet, Xiao Tan, Verena C Wimmer, Delphine Merino, Kelly L Rogers, and Shalin H Naik. Spatial omics and multiplexed imaging to explore cancer biology. *Nature methods*, 18(9):997–1012, 2021. 10.1038/s41592-021-01203-6.

[15] Candace C Liu et al. Multiplexed ion beam imaging: insights into pathobiology. *Annual Review of Pathology: Mechanisms of Disease*, 17:403–423, 2022. 10.1146/annurev-pathmechdis-030321-091459.

[16] Visium spatial gene expression, 10x genomics. `https://www.10xgenomics.com/products/spatial-gene-expression`. Accessed: 2024-10-25.

[17] Maldi imaging - reveal greater molecular insight, bruker. `https://www.bruker.com/en/applications/academia-life-science/imaging/maldi-imaging.html`. Accessed: 2024-10-25.

[18] The phenocycler-fusion 2.0 solution, akoya biosciences. `https://www.akoyabio.com/phenocycler/`. Accessed: 2024-10-25.

[19] Andreas Heindl, Sidra Nawaz, and Yinyin Yuan. Mapping spatial heterogeneity in the tumor microenvironment: a new era for digital pathology. *Laboratory investigation*, 95(4):377–384, 2015. 10.1038/labinvest.2014.155.

[20] Yinyin Yuan. Spatial heterogeneity in the tumor microenvironment. *Cold Spring Harbor perspectives in medicine*, 6(8):a026583, 2016. 10.1101/cshperspect.a026583.

[21] David Lähnemann, Johannes Köster, Ewa Szczurek, Davis J McCarthy, Stephanie C Hicks, Mark D Robinson, Catalina A Vallejos, Kieran R Campbell, Niko Beerenwinkel, Ahmed Mahfouz, et al. Eleven grand challenges in single-cell data science. *Genome biology*, 21:1–35, 2020. 10.3389/fcosc.2020.588568.

[22] Rashid Ahmed, Robin Augustine, Enrique Valera, Anurup Ganguli, Nasrin Mesaeli, Irfan S Ahmad, Rashid Bashir, and Anwarul Hasan. Spatial mapping of cancer tissues by omics technologies. *Biochimica et Biophysica Acta (BBA)-Reviews on Cancer*, 1877(1):188663, 2022. 10.1016/j.bbcan.2021.188663.

[23] Satoi Nagasawa, Junko Zenkoh, Yutaka Suzuki, and Ayako Suzuki. Spatial omics technologies for understanding molecular status associated with cancer progression. *Cancer Science*, 115(10):3208–3217, 2024. 10.1111/cas.16283.

[24] Daniel Edsgärd, Per Johnsson, and Rickard Sandberg. Identification of spatial expression trends in single-cell gene expression data. *Nature methods*, 15(5):339–342, 2018. 10.1038/nmeth.4634.

[25] Valentine Svensson, Sarah A Teichmann, and Oliver Stegle. Spatialde: identification of spatially variable genes. *Nature methods*, 15(5):343–346, 2018. 10.1038/nmeth.4636.

[26] Shiquan Sun, Jiaqiang Zhu, and Xiang Zhou. Statistical analysis of spatial expression patterns for spatially resolved transcriptomic studies. *Nature methods*, 17(2):193–200, 2020.

[27] Jiaqiang Zhu, Shiquan Sun, and Xiang Zhou. Spark-x: non-parametric modeling enables scalable and robust detection of spatial expression patterns for large spatial transcriptomic studies. *Genome Biology*, 22(1):1–25, 2021.

[28] Qiwei Li, Minzhe Zhang, Yang Xie, and Guanghua Xiao. Bayesian modeling of spatial molecular profiling data via gaussian process. *Bioinformatics*, 37(22):4129–4136, 2021. 10.1093/bioinformatics/btab455.

[29] Yuhan Hao, Stephanie Hao, Erica Andersen-Nissen, William M Mauck, Shiwei Zheng, Andrew Butler,

Maddie J Lee, Aaron J Wilk, Charlotte Darby, Michael Zager, et al. Integrated analysis of multimodal single-cell data. *Cell*, 184(13):3573–3587, 2021. 10.1016/j.cell.2021.04.048.

[30] Xi Jiang, Guanghua Xiao, and Qiwei Li. A bayesian modified ising model for identifying spatially variable genes from spatial transcriptomics data. *Statistics in Medicine*, 41(23):4647–4665, 2022. 10.1002/sim.9530.

[31] Ke Zhang, Wanwan Feng, and Peng Wang. Identification of spatially variable genes with graph cuts. *Nature Communications*, 13(1):5488, 2022. 10.1038/s41467-022-33182-3.

[32] Lukas M Weber, Arkajyoti Saha, Abhirup Datta, Kasper D Hansen, and Stephanie C Hicks. nnsvg for the scalable identification of spatially variable genes using nearest-neighbor gaussian processes. *Nature Communications*, 14(1):4059, 2023. 10.1038/s41467-023-39748-z.

[33] Souvik Seal, Benjamin G Bitler, and Debashis Ghosh. Smash: Scalable method for analyzing spatial heterogeneity of genes in spatial transcriptomics data. *PLoS Genetics*, 19(10):e1010983, 2023. 10.1371/journal.pgen.1010983.

[34] Rui Jiang, Zhen Li, Yuhang Jia, Siyu Li, and Shengquan Chen. Sinfonia: scalable identification of spatially variable genes for deciphering spatial domains. *Cells*, 12(4):604, 2023. 10.3390/cells12040604.

[35] Sikta Das Adhikari, Jiaxin Yang, Jianrong Wang, and Yuehua Cui. Recent advances in spatially variable gene detection in spatial transcriptomics. *Computational and Structural Biotechnology Journal*, 2024. 10.1016/j.csbj.2024.01.016.

[36] Peiying Cai, Mark D Robinson, and Simone Tiberi. Despace: spatially variable gene detection via differential expression testing of spatial clusters. *Bioinformatics*, 40(2):btae027, 2024. 10.1093/bioinformatics/btae027.

[37] Carissa Chen, Hani Jieun Kim, and Pengyi Yang. Evaluating spatially variable gene detection methods for spatial transcriptomics data. *Genome Biology*, 25(1):18, 2024. 10.1016/j.csbj.2024.01.016.

[38] Guanao Yan, Shuo Harper Hua, and Jingyi Jessica Li. Categorization of 34 computational methods to detect spatially variable genes from spatially resolved transcriptomics data. *Nature Communications*, 16(1):1141, 2025. 10.1038/s41467-025-56080-w.

[39] Kim Thrane, Hanna Eriksson, Jonas Maaskola, Johan Hansson, and Joakim Lundeberg. Spatially resolved transcriptomics enables dissection of genetic heterogeneity in stage iii cutaneous malignant melanoma. *Cancer research*, 78(20):5970–5979, 2018. 10.1158/0008-5472.can-18-0747.

[40] José Fernández Navarro, Deborah L Croteau, Aleksandra Jurek, Zaneta Andrusivova, Beimeng Yang, Yue Wang, Benjamin Ogedegbe, Tahira Riaz, Mari Støen, Claus Desler, et al. Spatial transcriptomics reveals genes associated with dysregulated mitochondrial functions and stress signaling in alzheimer disease. *Iscience*, 23(10), 2020. 10.1016/j.isci.2020.101556.

[41] Yuliang Wang, Shuyi Ma, and Walter L Ruzzo. Spatial modeling of prostate cancer metabolic gene expression reveals extensive heterogeneity and selective vulnerabilities. *Scientific reports*, 10(1):3490, 2020. 10.1038/s41598-020-60384-w.

[42] Anjali Rao, Dalia Barkley, Gustavo S França, and Itai Yanai. Exploring tissue architecture using spatial transcriptomics. *Nature*, 596(7871):211–220, 2021. 10.1038/s41586-021-03634-9.

[43] Edwin Roger Parra. Methods to determine and analyze the cellular spatial distribution extracted from multiplex immunofluorescence data to understand the tumor microenvironment. *Frontiers in Molecular Biosciences*, 8:668340, 2021. 10.3389/fmolb.2021.668340.

[44] Oliver Vipond et al. Multiparameter persistent homology landscapes identify immune cell spatial patterns in tumors. *Proceedings of the National Academy of Sciences*, 118(41):e2102166118, 2021. 10.1073/pnas.2102166118.

[45] Nicolas P Canete, Sourish S Iyengar, John T Ormerod, Heeva Baharlou, Andrew N Harman, and Ellis Patrick. spicyr: Spatial analysis of in situ cytometry data in r. *Bioinformatics*, 38(11):3099–3105, 2022. 10.1093/bioinformatics/btac268.

[46] Christopher Wilson et al. Tumor immune cell clustering and its association with survival in african american women with ovarian cancer. *PLoS Computational Biology*, 18(3):e1009900, 2022. 10.1371/journal.pcbi.1009900.

[47] Wilson Kuswanto, Garry Nolan, and Guolan Lu. Highly multiplexed spatial profiling with codex: bioinformatic analysis and application in human disease. *Seminars in Immunopathology*, 45(1):145–157, 2023.

[48] Thao Vu, Julia Wrobel, Benjamin G Bitler, Erin L Schenk, Kimberly R Jordan, and Debashis Ghosh. Spf: a spatial and functional data analytic approach to cell imaging data. *PLOS Computational Biology*, 18(6):e1009486, 2022. 10.1371/journal.pcbi.1009486.

[49] Vladan Milosevic. Different approaches to imaging mass cytometry data analysis. *Bioinformatics Advances*, 3(1):vbad046, 2023. 10.1093/bioadv/vbad046.

[50] Nathaniel Osher, Jian Kang, Santhoshi Krishnan, Arvind Rao, and Veerabhadran Baladandayuthapani. Spartin: a bayesian method for the quantification and characterization of cell type interactions in spatial pathology data. *Frontiers in Genetics*, 14:1175603, 2023. 10.3389/fgene.2023.1175603.

[51] Souvik Seal, Brian Neelon, Peggi M Angel, Elizabeth C O'Quinn, Elizabeth Hill, Thao Vu, Debashis Ghosh, Anand S Mehta, Kristin Wallace, and Alexander V Alekseyenko. Spaceanova: Spatial co-occurrence analysis of cell types in multiplex imaging data using point process and functional anova. *Journal of Proteome Research*, 23(4):1131–1143, 2024. 10.1021/acs.jproteome.3c00462.

[52] Edward Zhao, Matthew R Stone, Xing Ren, Jamie Guenthoer, Kimberly S Smythe, Thomas Pulliam, Stephen R Williams, Cedric R Uytingco, Sarah EB Taylor, Paul Nghiem, et al. Spatial transcriptomics at subspot resolution with bayesspace. *Nature biotechnology*, 39(11):1375–1384, 2021. 10.1038/s41587-021-00935-2.

[53] Kangning Dong and Shihua Zhang. Deciphering spatial domains from spatially resolved transcrip-

tomics with an adaptive graph attention auto-encoder. *Nature communications*, 13(1):1739, 2022. 10.1038/s41467-022-29439-6.

[54] Chang Xu, Xiyun Jin, Songren Wei, Pingping Wang, Meng Luo, Zhaochun Xu, Wenyi Yang, Yideng Cai, Lixing Xiao, Xiaoyu Lin, et al. Deepst: identifying spatial domains in spatial transcriptomics by deep learning. *Nucleic Acids Research*, 50(22):e131–e131, 2022. 10.1093/nar/gkac901.

[55] Carter Allen, Yuzhou Chang, Brian Neelon, Won Chang, Hang J Kim, Zihai Li, Qin Ma, and Dongjun Chung. A bayesian multivariate mixture model for high throughput spatial transcriptomics. *Biometrics*, 2022. 10.1111/biom.13727.

[56] Lulu Shang and Xiang Zhou. Spatially aware dimension reduction for spatial transcriptomics. *Nature Communications*, 13(1):7203, 2022. 10.1038/s41467-022-34879-1.

[57] Carter Allen, Yuzhou Chang, Qin Ma, and Dongjun Chung. Maple: a hybrid framework for multi-sample spatial transcriptomics data. *bioRxiv*, pages 2022–02, 2022. 10.1101/2022.02.28.482296.

[58] Yahui Long, Kok Siong Ang, Mengwei Li, Kian Long Kelvin Chong, Raman Sethi, Chengwei Zhong, Hang Xu, Zhiwei Ong, Karishma Sachaphibulkij, Ao Chen, et al. Spatially informed clustering, integration, and deconvolution of spatial transcriptomics with graphst. *Nature Communications*, 14(1):1155, 2023. 10.1038/s41467-023-36796-3.

[59] Yinqiao Yan and Xiangyu Luo. Bayesian integrative region segmentation in spatially resolved transcriptomic studies. *Journal of the American Statistical Association*, pages 1–13, 2024. 10.1080/01621459.2024.2308323.

[60] Ying Ma and Xiang Zhou. Accurate and efficient integrative reference-informed spatial domain detection for spatial transcriptomics. *Nature Methods*, pages 1–14, 2024. 10.1038/s41592-024-02284-9.

[61] Vipul Singhal, Nigel Chou, Joseph Lee, Yifei Yue, Jinyue Liu, Wan Kee Chock, Li Lin, Yun-Ching Chang, Erica Mei Ling Teo, Jonathan Aow, et al. Banksy unifies cell typing and tissue domain segmen-

tation for scalable spatial omics data analysis. *Nature Genetics*, 56(3):431–441, 2024. 10.1038/s41588-024-01664-3.

[62] Marco Varrone, Daniele Tavernari, Albert Santamaria-Martínez, Logan A Walsh, and Giovanni Ciriello. Cellcharter reveals spatial cell niches associated with tissue remodeling and cell plasticity. *Nature Genetics*, 56(1):74–84, 2024. 10.1038/s41593-024-01796-z.

[63] Zhiyuan Yuan. Mender: fast and scalable tissue structure identification in spatial omics data. *Nature Communications*, 15(1):207, 2024. 10.1038/s41467-023-44367-9.

[64] Christian M Schürch et al. Coordinated cellular neighborhoods orchestrate antitumoral immunity at the colorectal cancer invasive front. *Cell*, 182(5):1341–1359, 2020. 10.1016/j.cell.2020.07.005.

[65] Zhenghao Chen, Ilya Soifer, Hugo Hilton, Leeat Keren, and Vladimir Jojic. Modeling multiplexed images with spatial-lda reveals novel tissue microenvironments. *Journal of Computational Biology*, 27(8):1204–1218, 2020. 10.1089/cmb.2019.0340.

[66] Ellis Patrick, Nicolas P Canete, Sourish S Iyengar, Andrew N Harman, Greg T Sutherland, and Pengyi Yang. Spatial analysis for highly multiplexed imaging data to identify tissue microenvironments. *Cytometry Part A*, 103(7):593–599, 2023. 10.1002/cyto.a.24729.

[67] Rezvan Ehsani, Inge Jonassen, Lars A Akslen, and Dimitrios Kleftogiannis. Locator: feature extraction and spatial analysis of the cancer tissue microenvironment using mass cytometry imaging technologies. *Bioinformatics Advances*, 3(1):vbad146, 2023. 10.1093/bioadv/vbad146.

[68] Xiyu Peng, James W Smithy, Mohammad Yosofvand, Caroline E Kostrzewa, MaryLena Bleile, Fiona D Ehrich, Jasme Lee, Michael A Postow, Margaret K Callahan, Katherine S Panageas, et al. Decoding spatial tissue architecture: A scalable bayesian topic model for multiplexed imaging analysis. *bioRxiv*, pages 2024–10, 2024. 10.1101/2024.10.08.617293.

[69] Haoyang Mi, Shamilene Sivagnanam, Won Jin Ho, Shuming Zhang, Daniel Bergman, Atul Deshpande, Alexander S Baras, Elizabeth M Jaffee, Lisa M Coussens, Elana J Fertig, et al. Computational

methods and biomarker discovery strategies for spatial proteomics: a review in immuno-oncology. *Briefings in Bioinformatics*, 25(5):bbae421, 2024. 10.1093/bib/bbae421.

[70] Linlin Zhang, Dongsheng Chen, Dongli Song, Xiaoxia Liu, Yanan Zhang, Xun Xu, and Xiangdong Wang. Clinical and translational values of spatial transcriptomics. *Signal Transduction and Targeted Therapy*, 7(1):111, 2022. 10.1038/s41392-022-00960-w.

[71] Rohit Arora, Christian Cao, Mehul Kumar, Sarthak Sinha, Ayan Chanda, Reid McNeil, Divya Samuel, Rahul K Arora, T Wayne Matthews, Shamir Chandarana, et al. Spatial transcriptomics reveals distinct and conserved tumor core and edge architectures that predict survival and targeted therapy response. *Nature Communications*, 14(1):5029, 2023. 10.1038/s41467-023-40271-4.

[72] Yang Jin, Yuanli Zuo, Gang Li, Wenrong Liu, Yitong Pan, Ting Fan, Xin Fu, Xiaojun Yao, and Yong Peng. Advances in spatial transcriptomics and its applications in cancer research. *Molecular Cancer*, 23(1):129, 2024. 10.1016/j.critrevonc.2024.104430.

[73] Sebastian Gibb and Korbinian Strimmer. Maldiquant: a versatile r package for the analysis of mass spectrometry data. *Bioinformatics*, 28(17):2270–2271, 2012. 10.1093/bioinformatics/bts447.

[74] Pere Ràfols, Dídac Vilalta, Jesús Brezmes, Nicolau Cañellas, Esteban Del Castillo, Oscar Yanes, Noelia Ramírez, and Xavier Correig. Signal preprocessing, multivariate analysis and software tools for ma (ldi)-tof mass spectrometry imaging for biological applications. *Mass spectrometry reviews*, 37(3):281–306, 2018. 10.4155/bio-2017-0281.

[75] Kylie Ariel Bemis, Melanie Christine Föll, Dan Guo, Sai Srikanth Lakkimsetty, and Olga Vitek. Cardinal v. 3: a versatile open-source software for mass spectrometry imaging analysis. *Nature Methods*, 20(12):1883–1886, 2023. 10.1038/s41592-023-02070-z.

[76] Yonghui Dong and Uwe Heinig. Mass spectrometry imaging data analysis with shinycardinal. *Preprint, Research Square*, 2024. 10.21203/rs.3.rs-4072606/v1.

[77] James E Trosko, Randall J Ruch, et al. Cell-cell communication in carcinogenesis. *Front Biosci*, 3(3):d208–236, 1998. 10.2741/a275.

[78] A áM DeLise, L Fischer, and RS Tuan. Cellular interactions and signaling in cartilage development. *Osteoarthritis and cartilage*, 8(5):309–334, 2000. 10.1053/joca.1999.0306.

[79] Bonnie L Bassler. Small talk: cell-to-cell communication in bacteria. *Cell*, 109(4):421–424, 2002. 10.1023/a:1020522919555.

[80] Dongli Song, Dawei Yang, Charles A Powell, and Xiangdong Wang. Cell–cell communication: old mystery and new opportunity, 2019.

[81] Jimeng Su, Ying Song, Zhipeng Zhu, Xinyue Huang, Jibiao Fan, Jie Qiao, and Fengbiao Mao. Cell–cell communication: new insights and clinical implications. *Signal Transduction and Targeted Therapy*, 9(1):196, 2024. 10.1038/s41392-024-01888-z.

[82] Martin Meier-Schellersheim, Rajat Varma, and Bastian R Angermann. Mechanistic models of cellular signaling, cytokine crosstalk, and cell-cell communication in immunology. *Frontiers in immunology*, 10:2268, 2019. 10.3389/fimmu.2019.02268.

[83] Agnieszka Dominiak, Beata Chełstowska, Wioletta Olejarz, and Grażyna Nowicka. Communication in the cancer microenvironment as a target for therapeutic interventions. *Cancers*, 12(5):1232, 2020. 10.3390/cancers12051232.

[84] Nita K Pandit. *Introduction to the pharmaceutical sciences.* Lippincott Williams & Wilkins, 2007. 10.14494/jnrs2000.8.93.

[85] Yuanxin Wang, Ruiping Wang, Shaojun Zhang, Shumei Song, Changying Jiang, Guangchun Han, Michael Wang, Jaffer Ajani, Andy Futreal, and Linghua Wang. italk: an r package to characterize and illustrate intercellular communication. *BioRxiv*, page 507871, 2019. 10.1101/507871.

[86] Mirjana Efremova, Miquel Vento-Tormo, Sarah A Teichmann, and Roser Vento-Tormo. Cellphonedb: inferring cell–cell communication from combined expression of multi-subunit ligand–receptor complexes. *Nature protocols*, 15(4):1484–1506, 2020. 10.1038/s41596-020-0292-x.

[87] Erick Armingol, Adam Officer, Olivier Harismendy, and Nathan E Lewis. Deciphering cell–cell interactions and communication from gene expression. *Nature Reviews Genetics*, 22(2):71–88, 2021. 10.1038/s43586-021-00046-x.

[88] Suoqin Jin, Christian F Guerrero-Juarez, Lihua Zhang, Ivan Chang, Raul Ramos, Chen-Hsiang Kuan, Peggy Myung, Maksim V Plikus, and Qing Nie. Inference and analysis of cell-cell communication using cellchat. *Nature communications*, 12(1):1088, 2021. 10.1038/s41467-021-21246-9.

[89] Yang Zhang, Tianyuan Liu, Xuesong Hu, Mei Wang, Jing Wang, Bohao Zou, Puwen Tan, Tianyu Cui, Yiying Dou, Lin Ning, et al. Cellcall: integrating paired ligand–receptor and transcription factor activities for cell–cell communication. *Nucleic acids research*, 49(15):8520–8534, 2021. 10.1093/nar/gkab638.

[90] Qi Liu, Chih-Yuan Hsu, Jia Li, and Yu Shyr. Dysregulated ligand–receptor interactions from single-cell transcriptomics. *Bioinformatics*, 38(12):3216–3221, 2022. 10.1093/bioinformatics/btac294.

[91] Daniel Dimitrov, Dénes Türei, Martin Garrido-Rodriguez, Paul L Burmedi, James S Nagai, Charlotte Boys, Ricardo O Ramirez Flores, Hyojin Kim, Bence Szalai, Ivan G Costa, et al. Comparison of methods and resources for cell-cell communication inference from single-cell rna-seq data. *Nature communications*, 13(1):3224, 2022. 10.1038/s41467-022-30755-0.

[92] Zhaoyang Liu, Dongqing Sun, and Chenfei Wang. Evaluation of cell-cell interaction methods by integrating single-cell rna sequencing data with spatial information. *Genome Biology*, 23(1):218, 2022. 10.1093/bib/bbab565.

[93] Jiaxin Luo, Minghua Deng, Xuegong Zhang, and Xiaoqiang Sun. Esiccc as a systematic computational

framework for evaluation, selection, and integration of cell-cell communication inference methods. *Genome Research*, 33(10):1788–1805, 2023. 10.1101/gr.278001.123.

[94] Axel A Almet, Zixuan Cang, Suoqin Jin, and Qing Nie. The landscape of cell–cell communication through single-cell transcriptomics. *Current opinion in systems biology*, 26:12–23, 2021. 10.1016/j.coisb.2021.03.007.

[95] Mihir Bafna, Hechen Li, and Xiuwei Zhang. Clarify: cell–cell interaction and gene regulatory network refinement from spatially resolved transcriptomics. *Bioinformatics*, 39(Supplement_1):i484–i493, 2023. 10.1093/bioinformatics/btad269.

[96] Erick Armingol, Hratch M Baghdassarian, and Nathan E Lewis. The diversification of methods for studying cell–cell interactions and communication. *Nature Reviews Genetics*, 25(6):381–400, 2024. 10.1038/s41576-023-00685-8.

[97] Sudipto Banerjee, Bradley P Carlin, and Alan E Gelfand. *Hierarchical modeling and analysis for spatial data*. Chapman and Hall/CRC, 2014. 10.1201/b17115.

[98] Brendan F Miller, Dhananjay Bambah-Mukku, Catherine Dulac, Xiaowei Zhuang, and Jean Fan. Characterizing spatial gene expression heterogeneity in spatially resolved single-cell transcriptomic data with nonuniform cellular densities. *Genome research*, 31(10):1843–1855, 2021. 10.1101/gr.271288.120.

[99] Ruben Dries, Qian Zhu, Rui Dong, Chee-Huat Linus Eng, Huipeng Li, Kan Liu, Yuntian Fu, Tianxiao Zhao, Arpan Sarkar, Feng Bao, et al. Giotto: a toolbox for integrative analysis and visualization of spatial expression data. *Genome biology*, 22:1–31, 2021. 10.33764/2411-1759-2021-26-4-73-82.

[100] Qi Liu, Chih-Yuan Hsu, and Yu Shyr. Scalable and model-free detection of spatial patterns and colocalization. *Genome research*, 32(9):1736–1745, 2022. 10.1101/gr.276851.122.

[101] Xin Shao, Chengyu Li, Haihong Yang, Xiaoyan Lu, Jie Liao, Jingyang Qian, Kai Wang, Junyun Cheng, Penghui Yang, Huajun Chen, et al. Knowledge-graph-based cell-cell communication inference

for spatially resolved transcriptomic data with spatalk. *Nature Communications*, 13(1):4429, 2022. 10.1038/s41467-022-32111-8.

[102] Zhuoxuan Li, Tianjie Wang, Pentao Liu, and Yuanhua Huang. Spatialdm for rapid identification of spatially co-expressed ligand–receptor and revealing cell–cell communication patterns. *Nature communications*, 14(1):3995, 2023. 10.1038/s41467-023-39608-w.

[103] Suoqin Jin, Maksim V Plikus, and Qing Nie. Cellchat for systematic analysis of cell–cell communication from single-cell transcriptomics. *Nature Protocols*, pages 1–40, 2024. 10.1038/s41596-024-01045-4.

[104] Daniel Dimitrov, Philipp Sven Lars Schäfer, Elias Farr, Pablo Rodriguez-Mier, Sebastian Lobentanzer, Pau Badia-i Mompel, Aurelien Dugourd, Jovan Tanevski, Ricardo Omar Ramirez Flores, and Julio Saez-Rodriguez. Liana+ provides an all-in-one framework for cell–cell communication inference. *Nature Cell Biology*, 26(9):1613–1622, 2024. 10.1038/s41556-024-01469-w.

[105] Hirak Sarkar, Uthsav Chitra, Julian Gold, and Benjamin J Raphael. A count-based model for delineating cell–cell interactions in spatial transcriptomics data. *Bioinformatics*, 40(Supplement_1):i481–i489, 2024. 10.1093/bioinformatics/btae219.

[106] Zixuan Cang and Qing Nie. Inferring spatial and signaling relationships between cells from single cell transcriptomic data. *Nature communications*, 11(1):2084, 2020. 10.1038/s41467-020-15968-5.

[107] Zixuan Cang, Yanxiang Zhao, Axel A Almet, Adam Stabell, Raul Ramos, Maksim V Plikus, Scott X Atwood, and Qing Nie. Screening cell–cell communication in spatial transcriptomics via collective optimal transport. *Nature Methods*, 20(2):218–228, 2023. 10.1038/s41592-022-01728-4.

[108] Daniel Wartenberg. Multivariate spatial correlation: a method for exploratory geographical analysis. *Geographical analysis*, 17(4):263–283, 1985. 10.1111/j.1538-4632.1985.tb00849.x.

[109] Manfred M Fischer and Peter Nijkamp. *Geographic information systems, spatial modelling and policy evaluation.* Springer, 1993. 10.1007/978-3-642-77500-0.

[110] Sang-Il Lee. Developing a bivariate spatial association measure: an integration of pearson's r and moran's i. *Journal of geographical systems*, 3:369–385, 2001.

[111] Luc Anselin, Ibnu Syabri, Oleg Smirnov, et al. Visualizing multivariate spatial correlation with dynamically linked windows. In *Proceedings, CSISS Workshop on New Tools for Spatial Data Analysis, Santa Barbara, CA*, volume 2, 2002.

[112] Sang-Il Lee. A generalized significance testing method for global measures of spatial association: an extension of the mantel test. *Environment and Planning A*, 36(9):1687–1703, 2004. 10.1068/a34143.

[113] Boris Delaunay. Sur la sphère vide. a la mémoire de georges voronoï. *Bulletin de l'Académie des Sciences de l'URSS*, (6):793–800, 1934. 10.3406/bavf.1934.16827.

[114] Dawei Liu, Xihong Lin, and Debashis Ghosh. Semiparametric regression of multidimensional genetic pathway data: least-squares kernel machines and linear mixed models. *Biometrics*, 63(4):1079–1088, 2007. 10.1111/j.1541-0420.2007.00799.x.

[115] Christopher KI Williams and Carl Edward Rasmussen. *Gaussian processes for machine learning*, volume 2. MIT press Cambridge, MA, 2006.

[116] Luc Anselin. Local indicators of spatial association—lisa. *Geographical analysis*, 27(2):93–115, 1995. 10.1111/j.1538-4632.1995.tb00338.x.

[117] Lambda Moses, Pétur Helgi Einarsson, Kayla Jackson, Laura Luebbert, A Sina Booeshaghi, Sindri Antonsson, Nicolas Bray, Páll Melsted, and Lior Pachter. Voyager: exploratory single-cell genomics data analysis with geospatial statistics. *bioRxiv*, 2023. 10.1101/2023.07.20.549945.

[118] Francis J Anscombe. The transformation of poisson, binomial and negative-binomial data. *Biometrika*, 35(3/4):246–254, 1948. 10.2307/2332343.

[119] Constantin Ahlmann-Eltze and Wolfgang Huber. Comparison of transformations for single-cell rna-seq data. *Nature Methods*, 20(5):665–672, 2023. 10.1038/s41592-023-01814-1.

[120] Yuhan Hao, Tim Stuart, Madeline H Kowalski, Saket Choudhary, Paul Hoffman, Austin Hartman, Avi Srivastava, Gesmira Molla, Shaista Madad, Carlos Fernandez-Granda, et al. Dictionary learning for integrative, multimodal and scalable single-cell analysis. *Nature biotechnology*, 42(2):293–304, 2024. 10.1038/s41592-024-02436-x.

[121] Huimin Li, Bencong Zhu, Xi Jiang, Lei Guo, Yang Xie, Lin Xu, and Qiwei Li. An interpretable bayesian clustering approach with feature selection for analyzing spatially resolved transcriptomics data. *Biometrics*, 80(3):ujae066, 2024. 10.1093/biomtc/ujae066.

[122] Lyla Atta, Kalen Clifton, Manjari Anant, Gohta Aihara, and Jean Fan. Gene count normalization in single-cell imaging-based spatially resolved transcriptomics. *Genome Biology*, 25(1):153, 2024. 10.1093/bib/bbae576.

[123] Robert O'Hara and Johan Kotze. Do not log-transform count data. *Nature Precedings*, pages 1–1, 2010. 10.1038/npre.2010.4136.1.

[124] Xiao Xiao, Ethan P White, Mevin B Hooten, and Susan L Durham. On the use of log-transformation vs. nonlinear regression for analyzing biological power laws. *Ecology*, 92(10):1887–1894, 2011. 10.1890/11-0538.1.

[125] Changyong Feng, Hongyue Wang, Naiji Lu, Tian Chen, Hua He, Ying Lu, and Xin M Tu. Log-transformation and its implications for data analysis. *Shanghai archives of psychiatry*, 26(2):105–109, 2014. 10.1145/2790755.2790772.

[126] Robert M West. Best practice in statistics: The use of log transformation. *Annals of Clinical Biochemistry*, 59(3):162–165, 2022. 10.1055/a-1978-5575.

[127] Thorsten Schmidt. Coping with copulas. *Copulas-From theory to application in finance*, 3:1–34, 2007. 10.69645/fqiq6780.

[128] Jason H Moore. Bootstrapping, permutation testing and the method of surrogate data. *Physics in Medicine & Biology*, 44(6):L11, 1999. 10.1088/0031-9155/44/6/101.

[129] Robert P Haining. *Spatial data analysis: theory and practice.* Cambridge university press, 2003. 10.1109/tkde.2003.1198387.

[130] Peter Clifford, Sylvia Richardson, and Denis Hemon. Assessing the significance of the correlation between two spatial processes. *Biometrics*, pages 123–134, 1989. 10.2307/2532039.

[131] Robert Haining. Bivariate correlation with spatial data. *Geographical Analysis*, 23(3):210–227, 1991. 10.1111/j.1538-4632.1991.tb00235.x.

[132] Sylvia Richardson and Peter Clifford. Testing association between spatial processes. *Lecture Notes-Monograph Series*, pages 295–308, 1991. 10.1214/lnms/1215460509.

[133] Mark RT Dale and Marie-Josee Fortin. Spatial autocorrelation and statistical tests: some solutions. *Journal of Agricultural, Biological, and Environmental Statistics*, 14:188–206, 2009. 10.1198/jabes.2009.0012.

[134] Daniel A Griffith. *Advanced spatial statistics: special topics in the exploration of quantitative spatial data series*, volume 12. Springer Science & Business Media, 2012. 10.1016/j.stamet.2011.08.003.

[135] Fedelis Mutiso, John L Pearce, Sara E Benjamin-Neelon, Noel T Mueller, Hong Li, and Brian Neelon. Bayesian negative binomial regression with spatially varying dispersion: Modeling covid-19 incidence in georgia. *Spatial Statistics*, 52:100703, 2022. 10.1016/j.spasta.2022.100703.

[136] Alan E Gelfand, Hyon-Jung Kim, CF Sirmans, and Sudipto Banerjee. Spatial modeling with spatially varying coefficient processes. *Journal of the American Statistical Association*, 98(462):387–396, 2003. 10.1198/016214503000170.

[137] Tomoki Nakaya, Alexander S Fotheringham, Chris Brunsdon, and Martin Charlton. Geographically weighted poisson regression for disease association mapping. *Statistics in medicine*, 24(17):2695–2717, 2005. 10.1002/sim.2129.

[138] Peter Congdon. Spatial heterogeneity in bayesian disease mapping. *GeoJournal*, 84(5):1303–1316, 2019. 10.1016/j.atherosclerosis.2019.06.147.

[139] Binbin Lu, Martin Charlton, Paul Harris, and A Stewart Fotheringham. Geographically weighted regression with a non-euclidean distance metric: a case study using hedonic house price data. *International Journal of Geographical Information Science*, 28(4):660–681, 2014. 10.1080/13658816.2013.865739.

[140] Marco Helbich and Daniel A Griffith. Spatially varying coefficient models in real estate: Eigenvector spatial filtering and alternative approaches. *Computers, Environment and Urban Systems*, 57:1–11, 2016. 10.1016/j.compenvurbsys.2015.12.002.

[141] Andrew O Finley. Comparing spatially-varying coefficients models for analysis of ecological data with non-stationary and anisotropic residual dependence. *Methods in ecology and evolution*, 2(2):143–154, 2011. 10.1068/a43201.

[142] NAS Hamm, AO Finley, M Schaap, and A Stein. A spatially varying coefficient model for mapping pm10 air quality at the european scale. *Atmospheric Environment*, 102:393–405, 2015. 10.1016/j.atmosenv.2014.11.043.

[143] Hongtu Zhu, Jianqing Fan, and Linglong Kong. Spatially varying coefficient model for neuroimaging data with jump discontinuities. *Journal of the American Statistical Association*, 109(507):1084–1098, 2014. 10.1080/01621459.2014.881742.

[144] Tian Ge, Nicole Müller-Lenke, Kerstin Bendfeldt, Thomas E Nichols, and Timothy D Johnson. Analysis of multiple sclerosis lesions via spatially varying coefficients. *The annals of applied statistics*, 8(2):1095, 2014. 10.1214/14-aoas718.

[145] Julian Besag. Spatial interaction and the statistical analysis of lattice systems. *Journal of the Royal Statistical Society: Series B (Methodological)*, 36(2):192–225, 1974. 10.1111/j.2517-6161.1974.tb00999.x.

[146] Julian Besag, Jeremy York, and Annie Mollié. Bayesian image restoration, with two applications in spatial statistics. *Annals of the institute of statistical mathematics*, 43:1–20, 1991. 10.1007/bf00116466.

[147] Julian Besag and Charles Kooperberg. On conditional and intrinsic autoregressions. *Biometrika*, 82(4):733–746, 1995. 10.1093/biomet/82.4.733.

[148] Sudipto Banerjee, Alan E Gelfand, Andrew O Finley, and Huiyan Sang. Gaussian predictive process models for large spatial data sets. *Journal of the Royal Statistical Society Series B: Statistical Methodology*, 70(4):825–848, 2008. 10.1111/j.1467-9868.2008.00663.x.

[149] Abhirup Datta, Sudipto Banerjee, Andrew O Finley, and Alan E Gelfand. Hierarchical nearest-neighbor gaussian process models for large geostatistical datasets. *Journal of the American Statistical Association*, 111(514):800–812, 2016. 10.1080/01621459.2015.1044091.

[150] Havard Rue and Leonhard Held. *Gaussian Markov random fields: theory and applications*. Chapman and Hall/CRC, 2005. 10.1201/9780203492024.ch2.

[151] Robert Tibshirani, Michael Saunders, Saharon Rosset, Ji Zhu, and Keith Knight. Sparsity and smoothness via the fused lasso. *Journal of the Royal Statistical Society Series B: Statistical Methodology*, 67(1):91–108, 2005.

[152] Alessandro Rinaldo. Properties and refinements of the fused lasso. *The Annals of Statistics*, 95(5B):2922–2952, 2009. 10.1214/08-aos665.

[153] George Casella, Malay Ghosh, Jeff Gill, and Minjung Kyung. Penalized regression, standard errors, and Bayesian lassos. *Bayesian Analysis*, 5(2):369 – 411, 2010. 10.1214/10-ba607.

[154] Carlos M Carvalho, Nicholas G Polson, and James G Scott. Handling sparsity via the horseshoe. In *Artificial intelligence and statistics*, pages 73–80. PMLR, 2009.

[155] Sayantan Banerjee. Horseshoe shrinkage methods for bayesian fusion estimation. *Computational Statistics & Data Analysis*, 174:107450, 2022. 10.1016/j.csda.2022.107450.

[156] Yuko Kakikawa, Kaito Shimamura, and Shuichi Kawano. Bayesian fused lasso modeling via horseshoe prior. *Japanese Journal of Statistics and Data Science*, 6(2):705–727, 2023. 10.1007/s42081-023-00213-2.

[157] Furong Li and Huiyan Sang. Spatial homogeneity pursuit of regression coefficients for large datasets. *Journal of the American Statistical Association*, 2019. 10.1080/01621459.2018.1529595.

[158] Robert Clay Prim. Shortest connection networks and some generalizations. *The Bell System Technical Journal*, 36(6):1389–1401, 1957. 10.1002/j.1538-7305.1957.tb01515.x.

[159] Ronald L Graham and Pavol Hell. On the history of the minimum spanning tree problem. *Annals of the History of Computing*, 7(1):43–57, 1985. 10.1109/mahc.1985.10011.

[160] J Michael Steele. Minimal spanning trees for graphs with random edge lengths. In *Mathematics and Computer Science II: Algorithms, Trees, Combinatorics and Probabilities*, pages 223–245. Springer, 2002.

[161] Finn Lindgren, Håvard Rue, and Johan Lindström. An explicit link between gaussian fields and gaussian markov random fields: the stochastic partial differential equation approach. *Journal of the Royal Statistical Society Series B: Statistical Methodology*, 73(4):423–498, 2011. 10.1111/j.1467-9868.2011.00777.x.

[162] Sean C Anderson, Eric J Ward, Philina A English, and Lewis AK Barnett. sdmtmb: an r package for fast, flexible, and user-friendly generalized linear mixed effects models with spatial and spatiotemporal random fields. *BioRxiv*, pages 2022–03, 2022. 10.1101/2022.03.24.485545.

[163] Andrew L Ji, Adam J Rubin, Kim Thrane, Sizun Jiang, David L Reynolds, Robin M Meyers, Margaret G Guo, Benson M George, Annelie Mollbrink, Joseph Bergenstråhle, et al. Multimodal analysis of composition and spatial architecture in human squamous cell carcinoma. *cell*, 182(2):497–514, 2020. 10.1016/j.cell.2020.05.039.

[164] Dylan M Cable, Evan Murray, Luli S Zou, Aleksandrina Goeva, Evan Z Macosko, Fei Chen, and Rafael A Irizarry. Robust decomposition of cell type mixtures in spatial transcriptomics. *Nature biotechnology*, 40(4):517–526, 2022. 10.1038/s41592-022-01575-3.

[165] Peter Langfelder and Steve Horvath. Fast r functions for robust correlations and hierarchical clustering. *Journal of statistical software*, 46:1–17, 2012. 10.18637/jss.v046.i11.

[166] Patrice Delafontaine, Yao-Hua Song, and Yangxin Li. Expression, regulation, and function of igf-1, igf-1r, and igf-1 binding proteins in blood vessels. *Arteriosclerosis, thrombosis, and vascular biology*, 24(3):435–444, 2004. 10.1161/01.atv.0000105902.89459.09.

[167] Ettore Capoluongo. Insulin-like growth factor system and sporadic malignant melanoma. *The American journal of pathology*, 178(1):26–31, 2011. 10.1016/j.ajpath.2010.11.004.

[168] Talitha R Bakker, Christina Piperi, Elizabeth A Davies, and P Anton van der Merwe. Comparison of cd22 binding to native cd45 and synthetic oligosaccharide. *European journal of immunology*, 32(7):1924–1932, 2002. 10.1002/1521-4141(200207)32:7<1924::aid-immu1924>3.0.co;2-n.

[169] Xuemei Li, Zhanghui Yue, Dan Wang, and Lu Zhou. Ptprc functions as a prognosis biomarker in the tumor microenvironment of cutaneous melanoma. *Scientific Reports*, 13(1):20617, 2023. 10.1038/s41598-023-46794-6.

[170] Lars Nitschke, Rita Carsetti, Bettina Ocker, Georges Köhler, and Marinus C Lamers. Cd22 is a negative regulator of b-cell receptor signalling. *Current Biology*, 7(2):133–143, 1997. 10.1016/s0960-9822(06)00057-1.

[171] Fuller W Bazer, Gwonhwa Song, Jinyoung Kim, David W Erikson, Greg A Johnson, Robert C Burghardt, Haijun Gao, M Carey Satterfield, Thomas E Spencer, and Guoyao Wu. Mechanistic mammalian target of rapamycin (mtor) cell signaling: effects of select nutrients and secreted phosphoprotein 1 on development of mammalian conceptuses. *Molecular and cellular endocrinology*, 354(1-2):22–33, 2012. 10.1016/j.mce.2011.08.026.

[172] Wen Xie, Jia Cheng, Zhijun Hong, Wangyu Cai, Huiqin Zhuo, Jingjing Hou, Lingyun Lin, Xujin Wei, Kang Wang, Xin Chen, et al. Multi-transcriptomic analysis reveals the heterogeneity and tumor-promoting role of spp1/cd44-mediated intratumoral crosstalk in gastric cancer. *Cancers*, 15(1):164, 2022. 10.3390/cancers15010164.

[173] Dan E Rowe, Raymond J Carroll, and Calvin L Day Jr. Prognostic factors for local recurrence, metastasis, and survival rates in squamous cell carcinoma of the skin, ear, and lip: implications for treatment modality selection. *Journal of the American Academy of Dermatology*, 26(6):976–990, 1992. 10.1016/0190-9622(92)70144-5.

[174] Justin T Jacob, Pierre A Coulombe, Raymond Kwan, and M Bishr Omary. Types i and ii keratin intermediate filaments. *Cold Spring Harbor perspectives in biology*, 10(4):a018275, 2018. 10.1101/cshperspect.a018275.

[175] Luting Yang, Shaolong Zhang, and Gang Wang. Keratin 17 in disease pathogenesis: from cancer to dermatoses. *The Journal of pathology*, 247(2):158–165, 2019. 10.1016/j.jid.2019.03.528.

[176] Stefan Werner, Laura Keller, and Klaus Pantel. Epithelial keratins: Biology and implications as diagnostic markers for liquid biopsies. *Molecular aspects of medicine*, 72:100817, 2020. 10.1016/j.mam.2019.09.001.

[177] Oluseye Ogunnigbagbe, Christopher G Bunick, and Kamaljit Kaur. Keratin 1 as a cell-surface receptor in cancer. *Biochimica et Biophysica Acta (BBA)-Reviews on Cancer*, 1877(1):188664, 2022. 10.1016/j.bbcan.2021.188664.

[178] Matthew N Bernstein, Zijian Ni, Aman Prasad, Jared Brown, Chitrasen Mohanty, Ron Stewart, Michael A Newton, and Christina Kendziorski. Spatialcorr identifies gene sets with spatially varying correlation structure. *Cell Reports Methods*, 2(12), 2022. 10.1016/j.crmeth.2022.100369.

[179] Alexandra Buruiană, Bogdan-Alexandru Gheban, Ioana-Andreea Gheban-Roșca, Carmen Georgiu,

Doința Crișan, and Maria Crișan. The tumor stroma of squamous cell carcinoma: a complex environment that fuels cancer progression. *Cancers*, 16(9):1727, 2024. 10.3390/cancers16091727.

[180] Paul E Bowden. Mutations in a keratin 6 isomer (k6c) cause a type of focal palmoplantar keratoderma. *Journal of Investigative Dermatology*, 130(2):336–338, 2010. 10.1038/jid.2009.395.

[181] Meng Fu and Gang Wang. Keratin 17 as a therapeutic target for the treatment of psoriasis. *Journal of Dermatological Science*, 67(3):161–165, 2012. 10.1016/j.jdermsci.2012.06.008.

[182] Gabriella Baraks, Robert Tseng, Chun-Hao Pan, Saumya Kasliwal, Cindy V Leiton, Kenneth R Shroyer, and Luisa F Escobar-Hoyos. Dissecting the oncogenic roles of keratin 17 in the hallmarks of cancer. *Cancer research*, 82(7):1159–1166, 2022. 10.3389/fonc.2022.855807.

[183] Yiting Lin, Weigang Zhang, Bing Li, and Gang Wang. Keratin 17 in psoriasis: Current understanding and future perspectives. In *Seminars in cell & developmental biology*, volume 128, pages 112–119. Elsevier, 2022.

[184] Sarocha Chootipongchaivat, Nicolien T van Ravesteyn, Xiaoxue Li, Hui Huang, Harald Weedon-Fekjær, Marc D Ryser, Donald L Weaver, Elizabeth S Burnside, Brandy M Heckman-Stoddard, Harry J de Koning, et al. Modeling the natural history of ductal carcinoma in situ based on population data. *Breast Cancer Research*, 22:1–12, 2020. 10.1177/0969141320945736.

[185] Rebecca L Siegel, Kimberly D Miller, Hannah E Fuchs, and Ahmedin Jemal. Cancer statistics, 2022. *CA: a cancer journal for clinicians*, 72(1):7–33, 2022. 10.3322/caac.21708.

[186] Ekaterina Sergeevna Novoseletskaya, Pavel Vladimirovich Evdokimov, and Anastasia Yurievna Efimenko. Extracellular matrix-induced signaling pathways in mesenchymal stem/stromal cells. *Cell Communication and Signaling*, 21(1):244, 2023. 10.47056/1814-3490-2023-2-119-125.

[187] Taylor S Hulahan and Peggi M Angel. From ductal carcinoma in situ to invasive breast cancer: the prognostic value of the extracellular microenvironment. *Journal of Experimental & Clinical Cancer Research*, 43(1):329, 2024. 10.1038/s41392-024-01779-3.

[188] Caroline G Kittrell, Jade Macdonald, Blake Sells, Lyndsay E Young, David DeNardo, Peggi M Angel, and Richard R Drake. Establishing a multi-omic spatial ecm proteome and n-glycome of pancreatic ductal adenocarcinoma tissues. *Cancer Research*, 85(8_Supplement_1):2581–2581, 2025. 10.1158/1538-7445.am2025-2581.

[189] Haakon Bakka, Håvard Rue, Geir-Arne Fuglstad, Andrea Riebler, David Bolin, Janine Illian, Elias Krainski, Daniel Simpson, and Finn Lindgren. Spatial modeling with r-inla: A review. *Wiley Interdisciplinary Reviews: Computational Statistics*, 10(6):e1443, 2018. 10.1002/wics.1443.

[190] Zongmin Zhao, Anvay Ukidve, Jayoung Kim, and Samir Mitragotri. Targeting strategies for tissue-specific drug delivery. *Cell*, 181(1):151–167, 2020. 10.1016/j.cell.2020.02.001.

[191] Shuxin Yan, Jintong Na, Xiyu Liu, and Pan Wu. Different targeting ligands-mediated drug delivery systems for tumor therapy. *Pharmaceutics*, 16(2):248, 2024. 10.3390/pharmaceutics16020248.

[192] Yejin Sung, Youngjin Choi, Eun Sun Kim, Ju Hee Ryu, and Ick Chan Kwon. Receptor-ligand interactions for optimized endocytosis in targeted therapies. *Journal of Controlled Release*, 380:524–538, 2025. 10.1016/j.jconrel.2025.01.060.

[193] Abhirup Datta, Sudipto Banerjee, James S Hodges, and Leiwen Gao. Spatial disease mapping using directed acyclic graph auto-regressive (dagar) models. *Bayesian analysis*, 14(4):1221, 2019. 10.1214/19-ba1177.

[194] Zhao Tang Luo, Huiyan Sang, and Bani Mallick. A bayesian contiguous partitioning method for learning clustered latent variables. *Journal of Machine Learning Research*, 22(37):1–52, 2021. 10.1214/20-sts788.

[195] Reinhard Furrer and Stephan R Sain. spam: A sparse matrix r package with emphasis on mcmc methods for gaussian markov random fields. *Journal of Statistical Software*, 36:1–25, 2010. 10.18637/jss.v036.i10.

[196] Richard J Lipton, Donald J Rose, and Robert Endre Tarjan. Generalized nested dissection. *SIAM journal on numerical analysis*, 16(2):346–358, 1979. 10.1137/0716027.

[197] Sarah E Neville, John T Ormerod, and MP Wand. Mean field variational bayes for continuous sparse signal shrinkage: pitfalls and remedies. *Electronic Journal of Statistics*, 8:1113–1151, 2014. 10.1214/14-ejs910.

[198] Visweswaran Ravikumar, Tong Xu, Wajd N Al-Holou, Salar Fattahi, and Arvind Rao. Efficient inference of spatially-varying gaussian markov random fields with applications in gene regulatory networks. *IEEE/ACM transactions on computational biology and bioinformatics*, 20(5):2920–2932, 2023. 10.1109/tcbb.2023.3282028.

[199] Satwik Acharyya, Xiang Zhou, and Veerabhadran Baladandayuthapani. Spacex: gene co-expression network estimation for spatial transcriptomics. *Bioinformatics*, 38(22):5033–5041, 2022. 10.1093/bioinformatics/btac645.

[200] Arhit Chakrabarti, Yang Ni, and Bani K Mallick. Joint bayesian estimation of cell dependence and gene associations in spatially resolved transcriptomic data. *Scientific Reports*, 14(1):9516, 2024. 10.1038/s41598-024-60002-z.

[201] Jonathan Pillow and James Scott. Fully bayesian inference for neural models with negative-binomial spiking. *Advances in neural information processing systems*, 25, 2012. 10.1061/9780784412442.367.

[202] Sooyoung Cheon, Seuck Heun Song, and Byoung Cheol Jung. Tests for independence in a bivariate negative binomial model. *Journal of the Korean Statistical Society*, 38(2):185–190, 2009. 10.1016/j.jkss.2008.11.004.

[203] Felix Famoye. On the bivariate negative binomial regression model. *Journal of Applied Statistics*, 37(6):969–981, 2010. 10.1080/02664760902984618.

[204] Hunyong Cho, Chuwen Liu, John S Preisser, and Di Wu. A bivariate zero-inflated negative binomial

model and its applications to biomedical settings. *Statistical Methods in Medical Research*, 32(7):1300–1317, 2023. 10.1177/09622802231172028.

[205] Numan Ahmad, Vikash V Gayah, and Eric T Donnell. Copula-based bivariate count data regression models for simultaneous estimation of crash counts based on severity and number of vehicles. *Accident Analysis & Prevention*, 181:106928, 2023. 10.1016/j.aap.2022.106928.

[206] Muhammad Iqbal, Andysah Putera Utama Siahaan, Nathania Elizabeth Purba, and Dedi Purwanto. Prim's algorithm for optimizing fiber optic trajectory planning. *Int. J. Sci. Res. Sci. Technol*, 3(6):504–509, 2017. 10.31227/osf.io/w3rzg.

[207] Alan M Frieze. On the value of a random minimum spanning tree problem. *Discrete Applied Mathematics*, 10(1):47–56, 1985. 10.1016/0166-218x(85)90058-7.

[208] David Hallac, Jure Leskovec, and Stephen Boyd. Network lasso: Clustering and optimization in large graphs. In *Proceedings of the 21th ACM SIGKDD international conference on knowledge discovery and data mining*, pages 387–396, 2015.

[209] Trevor Park and George Casella. The bayesian lasso. *Journal of the american statistical association*, 103(482):681–686, 2008. 10.1198/016214508000000337.

[210] Robert Tibshirani. Regression shrinkage and selection via the lasso. *Journal of the Royal Statistical Society Series B: Statistical Methodology*, 58(1):267–288, 1996. 10.1111/j.2517-6161.1996.tb02080.x.

[211] Toshiki Sakai, Jun Tsuchida, and Hiroshi Yadohisa. Bayesian geographically weighted regression using fused lasso prior. *Spatial Statistics*, page 100884, 2025. 10.1016/j.spasta.2025.100884.

[212] Veronika Rockova, Emmanuel Lesaffre, Jolanda Luime, and Bob Löwenberg. Hierarchical bayesian formulations for selecting variables in regression models. *Statistics in medicine*, 31(11-12):1221–1237, 2012. 10.1002/sim.4439.

[213] Menelaos Pavlou, Gareth Ambler, Shaun Seaman, Maria De Iorio, and Rumana Z Omar. Review and evaluation of penalised regression methods for risk prediction in low-dimensional data with few events. *Statistics in medicine*, 35(7):1159–1177, 2016. 10.1016/j.gpb.2016.03.006.

[214] Kevin Walters, Angela Cox, and Hannuun Yaacob. The utility of the laplace effect size prior distribution in bayesian fine-mapping studies. *Genetic epidemiology*, 45(4):386–401, 2021. 10.1002/gepi.22375.

[215] Anindya Bhadra, Jyotishka Datta, Nicholas G Polson, and Brandon Willard. The horseshoe+ estimator of ultra-sparse signals. *Arxiv*, 2017. 10.1214/16-ba1028.

[216] Juho Piironen and Aki Vehtari. Sparsity information and regularization in the horseshoe and other shrinkage priors. *Arxiv*, 2017. 10.1214/17-ejs1337si.

[217] Anindya Bhadra, Jyotishka Datta, Nicholas G Polson, and Brandon Willard. Default bayesian analysis with global-local shrinkage priors. *Biometrika*, 103(4):955–969, 2016. 10.1093/biomet/asw041.

[218] Marie Denis and Mahlet G Tadesse. Graph-structured variable selection with gaussian markov random field horseshoe prior. *Statistical Modelling*, page 1471082X241310958, 2023. 10.1109/icip49359.2023.10222900.

[219] Julian Besag. Statistical analysis of non-lattice data. *Journal of the Royal Statistical Society Series D: The Statistician*, 24(3):179–195, 1975. 10.2307/2987782.

[220] Bruce G Lindsay. Composite likelihood methods. In *Statistical Inference from Stochastic Processes: Proceedings of the AMS-IMS-SIAM Joint Summer Research Conference Held August 9-15, 1987, with Support from the National Science Foundation and the Army Research Office*, volume 80, page 221. American Mathematical Soc., 1988.

[221] Cristiano Varin, Nancy Reid, and David Firth. An overview of composite likelihood methods. *Statistica Sinica*, pages 5–42, 2011. 10.1016/j.jspi.2011.03.026.

[222] James R Faulkner and Vladimir N Minin. Locally adaptive smoothing with markov random fields and shrinkage priors. *Bayesian analysis*, 13(1):225, 2017. 10.1186/s40535-017-0039-0.

[223] Nicholas G Polson, James G Scott, and Jesse Windle. Bayesian inference for logistic models using pólya–gamma latent variables. *Journal of the American statistical Association*, 108(504):1339–1349, 2013. 10.1080/01621459.2013.829001.

[224] Peter M Lee. *Bayesian statistics*. Oxford University Press London:, 1989. 10.2307/2348313.

[225] Dominique Makowski, Mattan S Ben-Shachar, and Daniel Lüdecke. bayestestr: Describing effects and their uncertainty, existence and significance within the bayesian framework. *Journal of open source software*, 4(40):1541, 2019. 10.21105/joss.01541.

[226] Dominique Makowski, Mattan S Ben-Shachar, SH Annabel Chen, and Daniel Lüdecke. Indices of effect existence and significance in the bayesian framework. *Frontiers in psychology*, 10:2767, 2019. 10.3389/fpsyg.2019.02767.

[227] Roger Bivand. R packages for analyzing spatial data: A comparative case study with areal data. *Geographical Analysis*, 54(3):488–518, 2022. 10.1111/gean.12319.

[228] John Geweke. Evaluating the accuracy of sampling-based approaches to the calculation of posterior moments. Technical report, Federal Reserve Bank of Minneapolis, 1991.

[229] Martyn Plummer, Nicky Best, Kate Cowles, and Karen Vines. Package 'coda'. *URL http://cran. r-project. org/web/packages/coda/coda. pdf, accessed January*, 25:2015, 2015. 10.1177/0740277515578630.

[230] Melanoma dataset, zenodo. `https://zenodo.org/records/8215682`. Accessed: 2026-01-05.

[231] cscc dataset, zenodo. `https://zenodo.org/records/8215682`. Accessed: 2026-01-05.

[232] Spatial proteomics dataset, zenodo. `https://zenodo.org/records/15866928`. Accessed: 2026-01-05.

Click here to access/download
Supplementary Material
SpaceBF_supplementary_GG.pdf

Replies to the review of "SpaceBF: Spatial coexpression analysis using Bayesian Fused approaches in spatial omics datasets"

Souvik Seal and Brian Neelon

Department of Public Health Sciences, College of Medicine, Medical University of South Carolina, Charleston, USA

October 2025

1 General comments

We thank the reviewers for their insightful comments, which motivated several substantial additions, including a lot of new results and theoretical developments. In particular, we have extended the proposed spatial horseshoe prior to support general graphs (beyond the MST), and we now include additional benchmarks against commonly used spatial priors, including ICAR and Matérn priors. In addition, we derive the asymptotic distribution of the bivariate Moran's I statistic and argue mathematically why it can be erratic when the two variables (e.g., genes) are truly independent, yet each exhibits spatial autocorrelation. All changes to the main text are highlighted in the color blue. The reference numbers here will not match the main text as they appear in document-specific order.

2 Reviewer 1

Summary: The manuscript introduces a novel statistical framework for analyzing spatially varying molecular co-expression. Leveraging a Bayesian fused modeling approach, SpaceBF estimates both local (location-

specific) and global (tissue-wide) co-expression patterns, particularly useful for studying cell-cell communication via ligand-receptor interactions. The method outperforms traditional geospatial metrics like bivariate Moran's I and Lee's L in terms of specificity and precision. Application of SpaceBF to spatial omics data reveals new insights into molecular interactions across various cancer types, offering a powerful tool for spatial omics research. The paper is nicely written, well structured, and has great visualizations, but I have the following comments.

1. The authors missed a couple of key references related to co-expression analysis of spatial omics data such as JOBS (Chakrabarti et al., 2024) and SpaceX (Acharyya et al., 2022). The authors are recommended to include these references in the Introduction Section.

   Response: We thank the reviewer for pointing out the manuscripts. We believe that these methods could be invaluable in extending our framework further, so we have now cited them in the Discussion section:

   "We have focused on pairwise analyses thus far; extending to joint modeling will follow prior works [1, 2]."

2. A method related figure can be included for visual illustration of the method.

   Response: We thank the reviewer for the comment. We have now added the following summary figure:

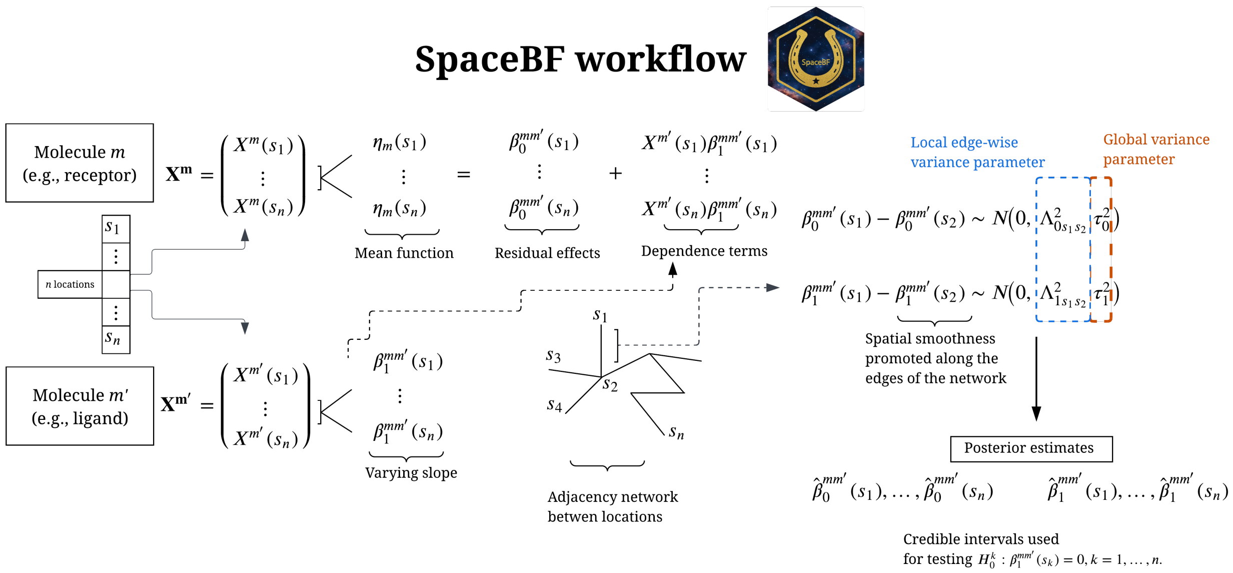

Figure 1: Graphical summary of the proposed approach.

3. In Melanoma ST data analysis, authors have used the RCTD algorithm (Cable et al., 2022) for cell-type estimation. It seems like the gene expression matrix has been used twice in the whole process: once in case of cell-type estimation and co-expression analysis afterwards. The obtained results can be highly correlated due to multiple uses of the gene expression matrix. It would be great if authors can address this issue.

Response: We thank the reviewer for the comment and apologize for the confusion. To clarify, the RCTD cell-types are shown solely for visual comparison and are not used in our model. We now state explicitly that the model includes no covariates:

"After filtering out genes with extremely low expression ($< 0.2 \times 293 \approx 59$ reads), 161 LR pairs remain, which were examined using our method SpaceBF, without adjusting for any covariates".

We also mention in the methods section that:

"To clarify, all applications in the manuscript assume no covariates, i.e., we do not include $C(s_k)$ or α_m, for simplicity. "

4. In the cSCC ST data analysis, BayesSpace (Zhao et al., 2021) algorithm has been used for spatial region identification. In Figure 2C, cluster numbers are provided only and those are not transferred to spatial regions. It is difficult to make spatial region specific inference without such regional annotation of clusters. The gene expression matrix is used multiple times in this case as well (spatial region identification and co-expression analysis).

   Response: We thank the reviewer for the comment. The BayesSpace clusters are presented solely to visually elucidate the tumor microenvironment (TME) and are not used in our model. Our intention was to illustrate that tumor versus non-tumor regions are not readily discernible from histology alone, whereas BayesSpace reveals differences in molecular expression/co-expression patterns. We state more clearly now in the main text:

   "We emphasize that these clusters are shown for visualization only and are not used in our analysis."

5. The spatial omics datasets are sparse in nature. It is possible that some these edges may not exist if the molecules are far apart. Authors are requested to justify the use shrinkage prior such as horseshoe rather than spike-and-slab prior.

   Response: We thank the reviewer for this insightful comment. To clarify, our prior penalizes pairwise differences between coefficients at adjacent locations in the spatial graph. Specifically, for the i-th edge connecting $(s_{k_i^1}, s_{k_i^2})$, the penalty acts on $|\beta_1^{mm'}(s_{k_i^1}) - \beta_1^{mm'}(s_{k_i^2})|$. Locations that are distant are not linked by an edge and are therefore not directly penalized. A spike-and-slab prior on pairwise differences is feasible, but it is likely more computationally challenging than a horseshoe prior, which affords more tractable MCMC sampling. Furthermore, one can induce additional sparsity on the coefficients themselves by placing extra shrinkage priors on the individual $\beta_1^{mm'}(s_k)$ terms.

6. While the authors briefly mention about the associated computational costs, it is recommended to include a comparison of the computational costs for different approaches in the simulation studies. This would provide a more comprehensive understanding of the proposed method's efficiency and feasibility. It will be also interesting to see the scalability of the method for large scale datasets.

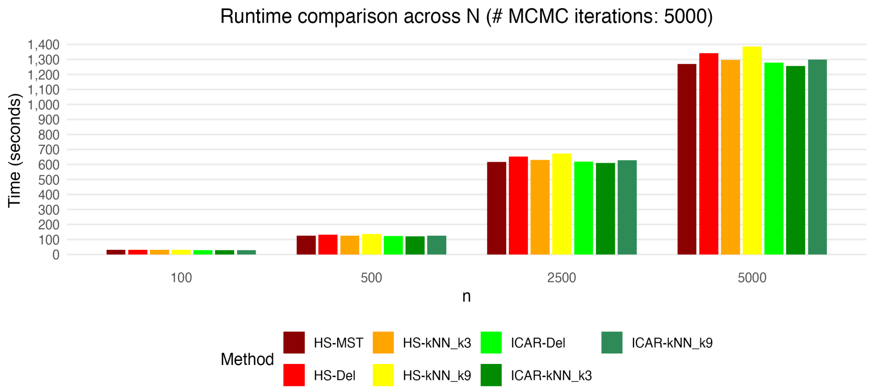

Figure 2: Run-time comparison of `SpaceBF`, with different priors: the horseshoe GMRF and ICAR, with varying spatial adjacency graphs.

Response: We thank the reviewer for the input. We have now moved the computational cost section from the supplementary to the main text and enhanced it with more benchmarks (with different adjacency graphs and priors) and comments in a new subsection titled "Runtime comparison and convergence diagnostics":

"In most analyses we ran 5,000 MCMC iterations with 2,500 burn-in. We compared runtimes for our package `SpaceBF` across priors and spatial backbones (from sparser to denser). Figure 2 shows that HS and ICAR have comparable runtimes, scaling approximately linearly with n. Denser graphs (e.g., k-NN with $k = 9$) are marginally slower. For $n = 5{,}000$, `SpaceBF` completes in about 20 minutes on a Mac Pro (M3 Max). For substantially larger datasets, a practical alternative is to consider `sdmTMB` [3], which fits an NB SVC model via a Laplace-approximate maximum likelihood approach. It is extremely fast but can be less precise, may fail to converge, and often requires tuning the mesh density for interpretable results.

For the convergence diagnostics, we computed the Geweke statistic [4] for each $\beta_1^{mm'}(s_k)$, implemented in the *R* package *coda* [5], and investigated the trace plots of a few randomly chosen $\beta_1^{mm'}(s_k)$'s (see the Supplementary Material). When either the variable m or m' is highly sparse ($> 75\%$ zeroes), imposing additional normal priors on $\beta_0^{mm'}(s_k)$'s and $\beta_1^{mm'}(s_k)$'s with a moderate variance, such as $N(0, 10)$, drastically improves mixing and overall convergence performance."

We further discuss the complexity in the Discussion section:

"Using the MST as the spatial graph offers several benefits: (i) uniqueness, removing the need to tune additional graph hyperparameters (e.g., GP lengthscales [6]); (ii) reduced computational burden via an exceptionally sparse precision matrix; and (iii) exact Gibbs updates for local horseshoe scales. In our simulations with spatial autocorrelation generated from a Gaussian process with an exponential kernel and varying lengthscales (but a domain-constant slope), the MST performs well, underscoring its robustness. Nonetheless, restricting the spatial structure to a single spanning tree can exclude salient edges [7], yielding noisier local slope estimates and overly sharp transition boundaries when coefficients vary spatially. In practice, a moderately denser graph, such as a kNN network with a small k, often achieves a better trade-off between computational efficiency and appropriate smoothness, as observed in our simulations. A more principled avenue could be to treat the spanning tree as unknown and update it iteratively within the model [8]. While we leverage the `spam` package [9] for fast sparse Cholesky factorization, overall complexity is graph-structure dependent (e.g., near $O(n)$ on trees/MSTs and typically around $O(n^{3/2})$ time for 2D planar/kNN graphs) [10]. As future work, we will pursue MCMC-free, variational-inference-based estimation to improve scalability [11, 12]."

7. To ensure the robustness of the proposed methodology, it is requested that the authors include a detailed sensitivity analysis for the selected priors and parameters.

Response: We thank the reviewer for the comment. For the horseshoe prior, we have simply used Carvalho et al. (2009)'s formulation [13],

$$\beta_1^{mm'}(s_{k_i^1}) - \beta_1^{mm'}(s_{k_i^2}) | \Lambda_{1i}^2, \tau_1^2 \sim N(0, \Lambda_{1i}^2 \tau_1^2), \;\; \Lambda_{1i} \sim C^+(0,1), \;\; \tau_1 \sim C^+(0,1),$$

where $C^+(0,1)$ denotes a half-cauchy distribution with location 0 and scale 1, and no additional hyperparameters require tuning ($\sigma^2 = 1$ in the NB model). Subsequent work [14] has suggested alternative priors for the global scale τ_1 that may further improve performance; we leave this extension to future work. However, we conducted a comprehensive sensitivity analysis with respect to the choice

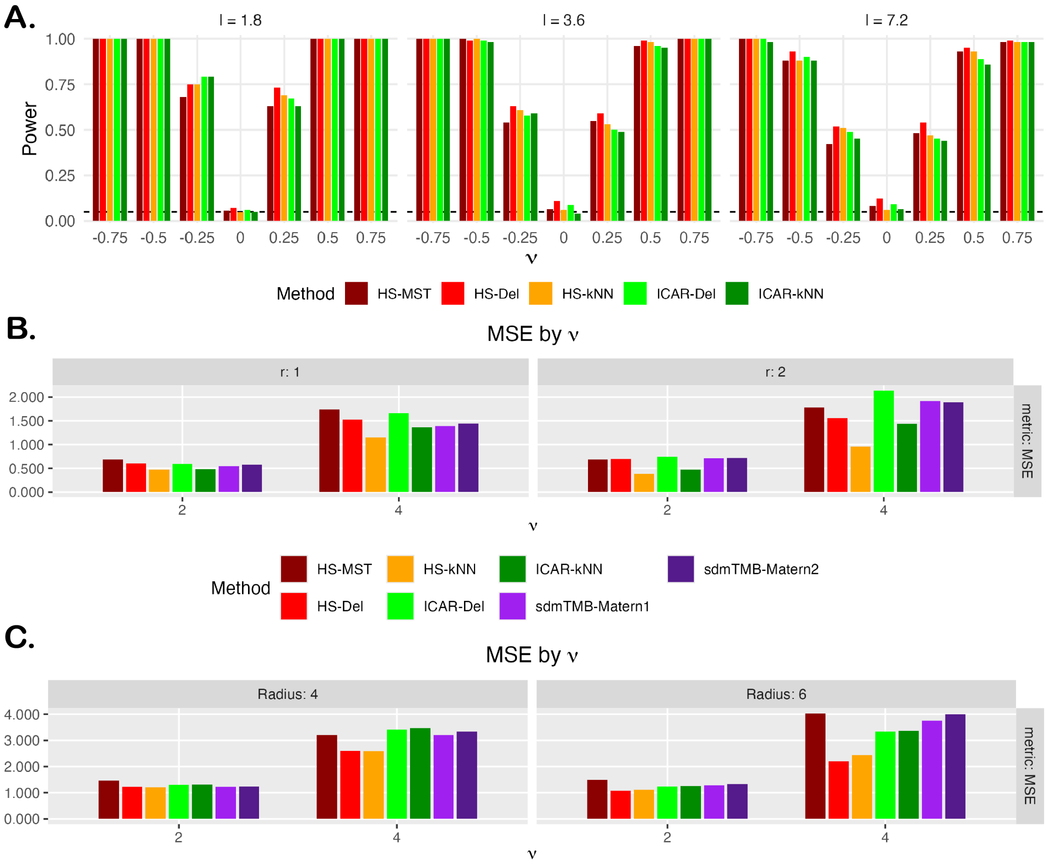

Figure 3: **A**. Power comparison of spatial priors under simulation design 2 for lengthscale l between $\{1.8, 3.6, 7.2\}$. **B**. MSE comparison of spatial priors under simulation design 3, linear partition boundary. **C**. MSE comparison of spatial priors under simulation design 3, circular boundary. In panel A, sdmTMB models are omitted due to recurrent convergence issues.

of adjacency graph, which indicated that the MST may not always be the most suitable option for our prior. Please see Sections 2.2.3 and 4.4.3. We have added substantial new text and several additional figures; for brevity, we present one representative figure here for reference.

3 Reviewer 2

I read your manuscript "SpaceBF: Spatial coexpression analysis using Bayesian Fused approaches in spatial omics datasets" with interest. The manuscript presents SpaceBF, a Bayesian method for detecting spatial

co-expression between pairs of molecules in spatial omics data. The topic is relevant since new technologies like spatial transcriptomics, mass spectrometry imaging, and multiplex immunofluorescence produce large data but current tools for co-expression are limited. The authors try to solve this gap with a new model and they also test it on real datasets. The paper is technical, but it also gives biological examples, which is helpful for readers. The paper has many strong points. First, the idea to use Bayesian fused horseshoe prior together with MST spatial structure is new and well explained. Second, the authors apply their method on three real datasets and they show interesting biology, for example IGF2-IGF1R relation, keratin isoform consistency, and stromal ECM peptides. Third, I appreciate that the code is open on GitHub. Also, the paper compares with other methods and deals with the common problem of variance-stabilizing transform by modeling UMI counts directly with negative binomial distribution. Overall, the work is clear and well organized, but there are some points where more explanation or clarification would help. In my review I give major and minor remarks that I hope will improve the paper.

3.1 Major remarks:

1. Were you worried choosing MST may oversimplify spatial relationships, since many meaningful local neighborhoods may be excluded? Would the results of SpaceBF be significantly different if a different spatial graph, such as kNN, Delaunay triangulation, or kernel-based, was used instead of MST? *Response:* We thank the reviewer for this helpful comment. As noted by the reviewer and mentioned in the Discussion section, an MST may be insufficient to capture spatial relationships in general datasets. Following the reviewer's suggestion, we went back and conducted additional experiments, finding that our framework extends naturally to other spatial graphs, including kNN and Delaunay. We now report results using a Delaunay graph, where our initial concern about oversmoothing did not materialize in practice. We also compare our priors (with both MST and Delaunay) against a standard ICAR prior (with Delaunay), showing that our approach outperforms ICAR with minimal computational overhead. Note that, compared with an MST, a general spatial graph yields a precision matrix with more nonzero entries, increasing the computational cost of the Cholesky decomposition.

We have added these new results in Sections 2.2.3 and 4.4.3, including the Figure 3 that is listed above. We are attaching only a small excerpt of the additions below for reference:

"While the spatial horseshoe (HS) prior is introduced on a minimum spanning tree (MST), it can be placed on any spatial backbone (e.g., Delaunay or kNN graphs), albeit with a potential risk of oversmoothing. This simulation study evaluates how graph choice affects HS performance. A Delaunay network is substantially denser than an MST, whereas a kNN network can serve as a middle ground for small k. In Fig. 3, HS-MST denotes HS on the MST (the original SpaceBF setting used in previous simulations and applications), HS-Del denotes HS on the Delaunay graph, and HS-kNN denotes HS on a kNN graph with $k = 3$. As noted in the Methods section, the ICAR prior is a special case of the HS prior; we therefore include ICAR-Del and ICAR-kNN for comparison. For completeness, we also consider a stochastic partial differential equation (SPDE) [15]-based NB SVC model implemented in the efficient `R` package `sdmTMB` [3], which uses a Matérn prior: sdmTMB-Matérn1 uses a denser mesh (cutoff = 1), and sdmTMB-Matérn2 uses a coarser mesh (cutoff = 1.5), see the Supplementary Material for a visual comparison."

2. Since MST edges depend a lot on pairwise L2 distances, how stable are the results if spatial coordinates are a little noisy, or if there are tissue registration errors?

   Response: We thank the reviewer for this excellent comment. Following the previous response, we believe that using a general, denser spatial graph will mitigate this problem. It will more strongly smooth the local slope estimates, potentially avoiding random registration errors.

3. The model puts one molecule as outcome and the other as predictor. Are the co-expression estimates still the same if you switch roles?

   Response: We thank the reviewer for the comment. In our experiments, interchanging the roles of the outcome and predictor genes had minimal impact on the results, i.e., the top selected pairs are the same. This is a minor limitation of the framework, and we briefly discuss it in the Supplementary Material. The choice can also be guided by biological considerations, e.g., treating the receptor as

the outcome and the ligand as the predictor, reflecting the canonical direction of signal transduction. In practice, such prior knowledge of pathway architecture can inform the choice and improve interpretability.

4. In the Results you mention "FDR < 0.1". Can you explain which method you used for FDR? Also, are the discoveries robust if you change the threshold (for example 0.05 vs 0.1)?

   Response: We thank the reviewer for the comment. We kept FDR at a modest 0.1 as our study is exploratory. As mentioned in the manuscript, we compute the approximate "p-value" using the *R* package `bayestestR` [16], and then adjust those values using the Benjamini-Hochberg method using "p.adjust" function in *R*. We have now added to Section 4.3 of the main text:

   "For FDR control, we apply the Benjamini–Hochberg procedure using the `p.adjust` function in *R*."

   Changing the threshold, we lose 6 LR pairs.

5. Do the simulation parameters (lengthscale, slope, dispersion) correspond to realistic biological signal strengths and spatial scales observed in real datasets? Three values of the lengthscale l are considered, l = 3.6, 7.2, 18. Why exactly these values? What does $\nu = 0.75$ mean in terms of effect size? How does l=18 compare to real tissue lengthscales?

   Response: We thank the reviewer for this helpful comment. The kernel lengthscale governs the range of spatial correlation: larger lengthscales induce stronger, longer-range dependence and smoother fields. In Figure 4A of the main text, we illustrate how the kernel matrix evolves as the lengthscale l increases from low to high. Although the values $l \in \{3.6, 7.2, 18\}$ may appear arbitrary, they were selected to span regimes of increasing correlation range. At $l = 18$, most off-diagonal entries of the kernel covariance matrix approach 1, indicating extremely high spatial dependence. While we did not estimate l from real data in this study, we deliberately explored a broad range of values in our simulations to stress-test the method and cover worst-case scenarios. ν is a correlation parameter taking values in $[-1, 1]$, with larger (smaller) values indicating a stronger positive (negative) association. A value of $\nu = 0.75$ denotes strong positive co-expression, which is likely extreme in the real

data context.

6. Can you describe runtime and memory for larger datasets, like 10X Visium with 5,000-20,000 spots? Is the current MCMC practical for this scale, or do you think approximate inference (like variational Bayes or INLA) is needed?

   Response: We thank the reviewer for raising this important point. Leveraging the efficient `spam` package in *R*, our methods are tractable for datasets with approximately 10,000 spots, with runtimes on the order of minutes (as shown in the figure). As a natural next step toward scaling to larger platforms such as Xenium, we plan to develop a variational inference implementation following several recent works in this area. Please see the detailed response to comment 6 of reviewer 1 to find our new additions to the text regarding this topic.

3.2 Minor remarks:

1. How sensitive are the results to the choice of hyperparameters for the Horseshoe prior?

   Response: We thank the reviewer for the comment. For the horseshoe prior, we have simply used Carvalho et al. (2009)'s formulation [13],

$$\beta_1^{mm'}(s_{k_i^1}) - \beta_1^{mm'}(s_{k_i^2})|\Lambda_{1i}^2, \tau_1^2 \sim N(0, \Lambda_{1i}^2\tau_1^2),\ \ \Lambda_{1i} \sim C^+(0,1),\ \ \tau_1 \sim C^+(0,1),$$

   where $C^+(0,1)$ denotes a half-cauchy distribution with location 0 and scale 1, and no additional hyperparameters require tuning ($\sigma^2 = 1$ in the NB model). Subsequent work [14] has suggested alternative priors for the global scale τ_1 that may further improve performance; we leave this extension to future work.

2. In the Results you state that keratins "co-express highly, meaning their binding patterns with any specific type 1 keratin should be similar." Please make clear that SpaceBF measures co-expression, not direct binding, so that conclusions are not overstated.

Response: We thank the reviewer for the suggestion. We have now edited the text as

"One important observation is that the Type 2 keratins KRT6A, KRT6B, and KRT6C are closely related isoforms of keratin 6 [17] and therefore tend to be strongly co-expressed. Consequently, their spatial association patterns with a given Type 1 keratin are expected to be similar, consistent with the patterns recovered by SpaceBF."

3. You mention SpatialCorr and Copulacci, but the comparison was not successful. Even if parameters were sensitive, I think one short numerical comparison in the supplement would be helpful.

   Response: We thank the reviewer for this suggestion. In attempting to benchmark `SpatialCorr` and `Copulacci`, we found that both packages incorporate substantial built-in preprocessing and/or require explicit cell-type information, which is not straightforward to disable and is often unnecessary for the simple bivariate association testing problem considered in our manuscript. In addition, the available documentation did not provide sufficient guidance. After corresponding with the authors via GitHub and adapting portions of the implementations to ensure applicability to our simulation design, we obtained unstable and/or clearly incorrect outputs (e.g., `SpatialCorr` had almost 0 power in every case) that we could not reconcile within the scope of this revision. As these issues may reflect our implementation choices or an incomplete interpretation of the software, we do not present these results as a formal comparison at this time. We will continue to investigate these packages in follow-up work.

4. You filter out genes with fewer than 59 total reads (0.2 x number of spots). Can you justify the choice of this threshold and show if results are stable for other thresholds (for example 0.1x or 0.5x)? Since many ligands and receptors are lowly expressed, is there a risk of losing meaningful biology? Since the dataset has only 293 spots, thresholds can have strong effect.

   Response: We thank the reviewer for this comment. Applying expression-based filtering is standard practice in spatial transcriptomics to reduce spurious findings driven by extremely low counts. For example, the MERINGUE pipeline (link) uses a minimum total-read threshold (e.g., `min.reads =`

100). We emphasize that our filtering is based solely on the *total* read count per gene and does not depend on the number of spots in which the gene is detected (i.e., we do not exclude genes simply because they are expressed in fewer than a specified number of spots). We agree with the reviewer's general point that, in highly sparse settings, the method may have difficulty reliably estimating a large number of parameters.

Following the reviewer's suggestion, we re-ran the analysis using a more permissive ($0.1\times$) filtering threshold. This yielded 130 additional ligand–receptor pairs in the melanoma dataset (291 pairs in total), of which only 19 were significant. Because our method is applied to each pair independently, all previously identified pairs remained detected.

References

[1] S. Acharyya, X. Zhou, and V. Baladandayuthapani. SpaceX: gene co-expression network estimation for spatial transcriptomics. *Bioinformatics*, 38(22):5033–5041, 2022. PMCID: PMC9665869.

[2] A. Chakrabarti, Y. Ni, and B. K. Mallick. Joint Bayesian estimation of cell dependence and gene associations in spatially resolved transcriptomic data. *Scientific Reports*, 14(1):9516, 2024.

[3] S. C. Anderson, E. J. Ward, P. A. English, and L. A. Barnett. sdmTMB: an R package for fast, flexible, and user-friendly generalized linear mixed effects models with spatial and spatiotemporal random fields. *BioRxiv*, pages 2022–03, 2022.

[4] J. Geweke. Evaluating the accuracy of sampling-based approaches to the calculation of posterior moments. Technical report, Federal Reserve Bank of Minneapolis, 1991.

[5] M. Plummer, N. Best, K. Cowles, and K. Vines. Package 'coda'. *URL http://cran. r-project. org/web/packages/coda/coda. pdf, accessed January*, 25:2015, 2015.

[6] S. Banerjee, B. P. Carlin, and A. E. Gelfand. *Hierarchical modeling and analysis for spatial data.* Chapman and Hall/CRC, 2014.

[7] A. Datta, S. Banerjee, J. S. Hodges, and L. Gao. Spatial disease mapping using directed acyclic graph auto-regressive (DAGAR) models. *Bayesian analysis*, 14(4):1221, 2019. PMCID: PMC8046356.

[8] Z. T. Luo, H. Sang, and B. Mallick. A Bayesian contiguous partitioning method for learning clustered latent variables. *Journal of Machine Learning Research*, 22(37):1–52, 2021.

[9] R. Furrer and S. R. Sain. spam: A sparse matrix R package with emphasis on MCMC methods for Gaussian Markov random fields. *Journal of Statistical Software*, 36:1–25, 2010.

[10] R. J. Lipton, D. J. Rose, and R. E. Tarjan. Generalized nested dissection. *SIAM journal on numerical analysis*, 16(2):346–358, 1979.

[11] S. E. Neville, J. T. Ormerod, and M. Wand. Mean field variational Bayes for continuous sparse signal shrinkage: pitfalls and remedies. *Electronic Journal of Statistics*, 8:1113–1151, 2014.

[12] V. Ravikumar, T. Xu, W. N. Al-Holou, S. Fattahi, and A. Rao. Efficient inference of spatially-varying Gaussian Markov random fields with applications in gene regulatory networks. *IEEE/ACM transactions on computational biology and bioinformatics*, 20(5):2920–2932, 2023.

[13] C. M. Carvalho, N. G. Polson, and J. G. Scott. Handling sparsity via the horseshoe. In *Artificial intelligence and statistics*, pages 73–80. PMLR, 2009.

[14] J. Piironen and A. Vehtari. Sparsity information and regularization in the horseshoe and other shrinkage priors. *Arxiv*, 2017.

[15] F. Lindgren, H. Rue, and J. Lindström. An explicit link between Gaussian fields and Gaussian Markov random fields: the stochastic partial differential equation approach. *Journal of the Royal Statistical Society Series B: Statistical Methodology*, 73(4):423–498, 2011.

[16] D. Makowski, M. S. Ben-Shachar, and D. Lüdecke. bayestestR: Describing effects and their uncertainty, existence and significance within the Bayesian framework. *Journal of open source software*, 4(40):1541, 2019.

[17] P. E. Bowden. Mutations in a keratin 6 isomer (K6c) cause a type of focal palmoplantar keratoderma. *Journal of Investigative Dermatology*, 130(2):336–338, 2010.
