# Supplementary material for: SpaceBF: spatial coexpression analysis using Bayesian fused approaches in spatial omics datasets
Source: Gigascience. 2026 Jan 20;15:giag006. doi: 10.1093/gigascience/giag006 (PMC12954175; doi:10.1093/gigascience/giag006)
Supplement: giag006_Authors_Response_To_Reviewer_Comments_Original_Submission [file giag006_authors_response_to_reviewer_comments_original_submission.pdf]

Author's Response To Reviewer Comments

We thank the reviewers and the editor for their invaluable comments. We have attached a PDF file to the resubmission with our responses, which we will try to copy-paste here, but the formatting might be off with figures not displaying. Please consider the PDF file for review.

1 General comments
We thank the reviewers for their insightful comments, which motivated several substantial additions, including a lot of new results and theoretical developments. In particular, we have extended the proposed spatial horseshoe prior to support general graphs (beyond the MST), and we now include additional benchmarks against commonly used spatial priors, including ICAR and Mat´ern priors. In addition, we derive the asymptotic distribution of the bivariate Moran's I statistic and argue mathematically why it can be erratic when the two variables (e.g., genes) are truly independent, yet each exhibits spatial autocorrelation. All changes to the main text are highlighted in the color blue. The reference numbers here will not match the main text as they appear in document-specific order.

2 Reviewer 1

Summary: The manuscript introduces a novel statistical framework for analyzing spatially varying molecular co-expression. Leveraging a Bayesian fused modeling approach, SpaceBF estimates both local (locationspecific) and global (tissue-wide) co-expression patterns, particularly useful for studying cell-cell communication via ligand-receptor interactions. The method outperforms traditional geospatial metrics like bivariate Moran's I and Lee's L in terms of specificity and precision. Application of SpaceBF to spatial omics data reveals new insights into molecular interactions across various cancer types, offering a powerful tool for spatial omics research. The paper is nicely written, well structured, and has great visualizations, but I have the following comments.

1. The authors missed a couple of key references related to co-expression analysis of spatial omics data such as JOBS (Chakrabarti et al., 2024) and SpaceX (Acharyya et al., 2022). The authors are recommended to include these references in the Introduction Section.

Response: We thank the reviewer for pointing out the manuscripts. We believe that these methods could be invaluable in extending our framework further, so we have now cited them in the Discussion section:
"We have focused on pairwise analyses thus far; extending to joint modeling will follow prior works [1, 2]."

2. A method-related figure can be included for visual illustration of the method.
Response: We thank the reviewer for the comment. We have now added a summary figure.

3. In Melanoma ST data analysis, authors have used the RCTD algorithm (Cable et al., 2022) for celltype estimation. It seems like the gene expression matrix has been used twice in the whole process: once in case of cell-type estimation and co-expression analysis afterwards. The obtained results can be highly correlated due to multiple uses of the gene expression matrix. It would be great if authors can address this issue.

Response: We thank the reviewer for the comment and apologize for the confusion. To clarify, the RCTD cell-types are shown solely for visual comparison and are not used in our model. We now state explicitly that the model includes no covariates:
"After filtering out genes with extremely low expression ($< 0.2 \times 293 \approx 59$ reads), 161 LR pairs remain, which were examined using our method SpaceBF, without adjusting for any covariates".
We also mention in the methods section that:
"To clarify, all applications in the manuscript assume no covariates, i.e., we do not include C(sk) or αm, for simplicity. "
4. In the cSCC ST data analysis, BayesSpace (Zhao et al., 2021) algorithm has been used for spatial region

identification. In Figure 2C, cluster numbers are provided only and those are not transferred to spatial regions. It is difficult to make spatial region specific inference without such regional annotation of clusters. The gene expression matrix is used multiple times in this case as well (spatial region identification and co-expression analysis).

Response: We thank the reviewer for the comment. The BayesSpace clusters are presented solely to visually elucidate the tumor microenvironment (TME) and are not used in our model. Our intention was to illustrate that tumor versus non-tumor regions are not readily discernible from histology alone, whereas BayesSpace reveals differences in molecular expression/co-expression patterns. We state more clearly now in the main text:
"We emphasize that these clusters are shown for visualization only and are not used in our analysis."

5. The spatial omics datasets are sparse in nature. It is possible that some these edges may not exist if the molecules are far apart. Authors are requested to justify the use shrinkage prior such as horseshoe rather than spike-and-slab prior.
Response: We thank the reviewer for this insightful comment. To clarify, our prior penalizes pairwise differences between coefficients at adjacent locations in the spatial graph. Specifically, for the i-th edge connecting (ski1,ski2), the penalty acts on . Locations that are distant
are not linked by an edge and are therefore not directly penalized. A spike-and-slab prior on pairwise differences is feasible, but it is likely more computationally challenging than a horseshoe prior, which affords more tractable MCMC sampling. Furthermore, one can induce additional sparsity on the coefficients themselves by placing extra shrinkage priors on the individual) terms.

6. While the authors briefly mention about the associated computational costs, it is recommended to include a comparison of the computational costs for different approaches in the simulation studies. This would provide a more comprehensive understanding of the proposed method's efficiency and feasibility. It will be also interesting to see the scalability of the method for large scale datasets.

Response: We thank the reviewer for the input. We have now moved the computational cost section from the supplementary to the main text and enhanced it with more benchmarks (with different adjacency graphs and priors) and comments in a new subsection titled "Runtime comparison and convergence diagnostics":
"In most analyses we ran 5,000 MCMC iterations with 2,500 burn-in. We compared runtimes for our package SpaceBF across priors and spatial backbones (from sparser to denser). Figure 2 shows that HS and ICAR have comparable runtimes, scaling approximately linearly with n. Denser graphs (e.g., k-NN with k = 9) are marginally slower. For n = 5,000, SpaceBF completes in about 20 minutes on a Mac Pro (M3 Max). For substantially larger datasets, a practical alternative is to consider sdmTMB [3], which fits an NB SVC model via a Laplace-approximate maximum likelihood approach. It is extremely fast but can be less precise, may fail to converge, and often requires tuning the mesh density for interpretable results.
For the convergence diagnostics, we computed the Geweke statistic [4] for each), implemented in the R package coda [5], and investigated the trace plots of a few randomly chosen)'s (see the Supplementary Material). When either the variable m or m′ is highly sparse (> 75% zeroes), imposing additional normal priors on)'s and)'s with a moderate variance, such as
N(0,10), drastically improves mixing and overall convergence performance."
We further discuss the complexity in the Discussion section:
"Using the MST as the spatial graph offers several benefits: (i) uniqueness, removing the need to tune additional graph hyperparameters (e.g., GP lengthscales [6]); (ii) reduced computational burden via an exceptionally sparse precision matrix; and (iii) exact Gibbs updates for local horseshoe scales. In our simulations with spatial autocorrelation generated from a Gaussian process with an exponential kernel and varying lengthscales (but a domain-constant slope), the MST performs well, underscoring its robustness. Nonetheless, restricting the spatial structure to a single spanning tree can exclude salient edges [7], yielding noisier local slope estimates and overly sharp transition boundaries when coefficients vary spatially. In practice, a moderately denser graph, such as a kNN network with a small k, often achieves a better trade-off between computational efficiency and appropriate smoothness, as observed in our simulations. A more principled avenue could be to treat the spanning tree as unknown and update it iteratively within the model [8]. While we leverage the spam package [9] for fast sparse Cholesky factorization, overall complexity is graph-structure dependent (e.g., near O(n) on trees/MSTs and typically

around O(n3/2) time for 2D planar/kNN graphs) [10]. As future work, we will pursue MCMC-free, variational-inference-based estimation to improve scalability [11, 12]."

7. To ensure the robustness of the proposed methodology, it is requested that the authors include a detailed sensitivity analysis for the selected priors and parameters.

Response: We thank the reviewer for the comment. For the horseshoe prior, we have simply used Carvalho et al. (2009)'s formulation [13],

,

where C+(0,1) denotes a half-cauchy distribution with location 0 and scale 1, and no additional hyperparameters require tuning (σ2 = 1 in the NB model). Subsequent work [14] has suggested alternative priors for the global scale τ1 that may further improve performance; we leave this extension to future work. However, we conducted a comprehensive sensitivity analysis with respect to the choice

Figure 3: A. Power comparison of spatial priors under simulation design 2 for lengthscale l between {1.8,3.6,7.2}. B. MSE comparison of spatial priors under simulation design 3, linear partition boundary. C. MSE comparison of spatial priors under simulation design 3, circular boundary. In panel A, sdmTMB models are omitted due to recurrent convergence issues.

of adjacency graph, which indicated that the MST may not always be the most suitable option for our prior. Please see Sections 2.2.3 and 4.4.3. We have added substantial new text and several additional figures; for brevity, we present one representative figure here for reference.

3 Reviewer 2

I read your manuscript "SpaceBF: Spatial coexpression analysis using Bayesian Fused approaches in spatial omics datasets" with interest. The manuscript presents SpaceBF, a Bayesian method for detecting spatial co-expression between pairs of molecules in spatial omics data. The topic is relevant since new technologies like spatial transcriptomics, mass spectrometry imaging, and multiplex immunofluorescence produce large data but current tools for co-expression are limited. The authors try to solve this gap with a new model and they also test it on real datasets. The paper is technical, but it also gives biological examples, which is helpful for readers. The paper has many strong points. First, the idea to use Bayesian fused horseshoe prior together with MST spatial structure is new and well explained. Second, the authors apply their method on three real datasets and they show interesting biology, for example IGF2-IGF1R relation, keratin isoform consistency, and stromal ECM peptides. Third, I appreciate that the code is open on GitHub. Also, the paper compares with other methods and deals with the common problem of variance-stabilizing transform by modeling UMI counts directly with negative binomial distribution. Overall, the work is clear and well organized, but there are some points where more explanation or clarification would help. In my review I give major and minor remarks that I hope will improve the paper.

3.1 Major remarks:

1. Were you worried choosing MST may oversimplify spatial relationships, since many meaningful local neighborhoods may be excluded? Would the results of SpaceBF be significantly different if a different spatial graph, such as kNN, Delaunay triangulation, or kernel-based, was used instead of MST?

Response: We thank the reviewer for this helpful comment. As noted by the reviewer and mentioned in the Discussion section, an MST may be insufficient to capture spatial relationships in general datasets. Following the reviewer's suggestion, we went back and conducted additional experiments, finding that our framework extends naturally to other spatial graphs, including kNN and Delaunay. We now report results using a Delaunay graph, where our initial concern about oversmoothing did not materialize in practice. We also compare our priors (with both MST and Delaunay) against a standard ICAR prior (with Delaunay), showing that our approach outperforms ICAR with minimal computational overhead. Note that, compared with an MST, a general spatial graph yields a precision matrix with more nonzero entries, increasing the computational cost of the Cholesky decomposition.

We have added these new results in Sections 2.2.3 and 4.4.3, including the Figure 3 that is listed above. We are attaching only a small excerpt of the additions below for reference:

"While the spatial horseshoe (HS) prior is introduced on a minimum spanning tree (MST), it can be placed on any spatial backbone (e.g., Delaunay or kNN graphs), albeit with a potential risk of oversmoothing. This simulation study evaluates how graph choice affects HS performance. A Delaunay network is substantially denser than an MST, whereas a kNN network can serve as a middle ground for small k. In Fig. 3, HS-MST denotes HS on the MST (the original SpaceBF setting used in previous simulations and applications), HS-Del denotes HS on the Delaunay graph, and HS-kNN denotes HS on a kNN graph with k = 3. As noted in the Methods section, the ICAR prior is a special case of the HS prior; we therefore include ICAR-Del and

ICAR-kNN for comparison. For completeness, we also consider a stochastic partial differential equation (SPDE) [15]-based NB SVC model implemented in the efficient R package sdmTMB [3], which uses a Mat´ern prior: sdmTMB-Mat´ern1 uses a denser mesh (cutoff = 1), and sdmTMB-Mat´ern2 uses a coarser mesh (cutoff = 1.5), see the Supplementary Material for a visual comparison."

2. Since MST edges depend a lot on pairwise L2 distances, how stable are the results if spatial coordinates are a little noisy, or if there are tissue registration errors?

Response: We thank the reviewer for this excellent comment. Following the previous response, we believe that using a general, denser spatial graph will mitigate this problem. It will more strongly smooth the local slope estimates, potentially avoiding random registration errors.

3. The model puts one molecule as outcome and the other as predictor. Are the co-expression estimates still the same if you switch roles?

Response: We thank the reviewer for the comment. In our experiments, interchanging the roles of the outcome and predictor genes had minimal impact on the results, i.e., the top selected pairs are the same. This is a minor limitation of the framework, and we briefly discuss it in the Supplementary Material. The choice can also be guided by biological considerations, e.g., treating the receptor as the outcome and the ligand as the predictor, reflecting the canonical direction of signal transduction. In practice, such prior knowledge of pathway architecture can inform the choice and improve interpretability.

4. In the Results you mention "FDR < 0.1". Can you explain which method you used for FDR? Also, are the discoveries robust if you change the threshold (for example 0.05 vs 0.1)?

Response: We thank the reviewer for the comment. We kept FDR at a modest 0.1 as our study is exploratory. As mentioned in the manuscript, we compute the approximate "p-value" using the R package bayestestR [16], and then adjust those values using the Benjamini-Hochberg method using "p.adjust" function in R. We have now added to Section 4.3 of the main text:
"For FDR control, we apply the Benjamini–Hochberg procedure using the p.adjust function in R." Changing the threshold, we lose 6 LR pairs.

5. Do the simulation parameters (lengthscale, slope, dispersion) correspond to realistic biological signal strengths and spatial scales observed in real datasets? Three values of the lengthscale l are considered, l = 3.6, 7.2, 18. Why exactly these values? What does v = 0.75 mean in terms of effect size? How does l=18 compare to real tissue lengthscales?

Response: We thank the reviewer for this helpful comment. The kernel lengthscale governs the range of spatial correlation: larger lengthscales induce stronger, longer-range dependence and smoother fields. In Figure 4A of the main text, we illustrate how the kernel matrix evolves as the lengthscale l increases from low to high. Although the values $l \in \{3.6,7.2,18\}$ may appear arbitrary, they were selected to span regimes of increasing correlation range. At l = 18, most off-diagonal entries of the kernel covariance matrix approach 1, indicating extremely high spatial dependence. While we did not estimate l from real data in this study, we deliberately explored a broad range of values in our simulations to stress-test the method and cover worst-case scenarios. v is a correlation parameter taking values in $[-1,1]$, with larger (smaller) values indicating a stronger positive (negative) association. A value of v = 0.75 denotes strong positive co-expression, which is likely extreme in the real data context.

6. Can you describe runtime and memory for larger datasets, like 10X Visium with 5,000-20,000 spots? Is the current MCMC practical for this scale, or do you think approximate inference (like variational Bayes or INLA) is needed?

Response: We thank the reviewer for raising this important point. Leveraging the efficient spam package in R, our methods are tractable for datasets with approximately 10,000 spots, with runtimes on the order of minutes (as shown in the figure). As a natural next step toward scaling to larger platforms such as Xenium, we plan to develop a variational inference implementation following several recent works in this area. Please see the detailed response to comment 6 of reviewer 1 to find our new additions to the text

regarding this topic.
3.2 Minor remarks:
1. How sensitive are the results to the choice of hyperparameters for the Horseshoe prior?
Response: We thank the reviewer for the comment. For the horseshoe prior, we have simply used Carvalho et al. (2009)'s formulation [13],

,

where C+(0,1) denotes a half-cauchy distribution with location 0 and scale 1, and no additional hyperparameters require tuning ($\sigma 2 = 1$ in the NB model). Subsequent work [14] has suggested alternative priors for the global scale $\tau 1$ that may further improve performance; we leave this extension to future work.

2. In the Results you state that keratins "co-express highly, meaning their binding patterns with any specific type 1 keratin should be similar." Please make clear that SpaceBF measures co-expression, not direct binding, so that conclusions are not overstated.

Response: We thank the reviewer for the suggestion. We have now edited the text as
"One important observation is that the Type 2 keratins KRT6A, KRT6B, and KRT6C are closely related isoforms of keratin 6 [17] and therefore tend to be strongly co-expressed. Consequently, their spatial association patterns with a given Type 1 keratin are expected to be similar, consistent with the patterns recovered by SpaceBF."

3. You mention SpatialCorr and Copulacci, but the comparison was not successful. Even if parameters were sensitive, I think one short numerical comparison in the supplement would be helpful.

Response: We thank the reviewer for this suggestion. In attempting to benchmark SpatialCorr and Copulacci, we found that both packages incorporate substantial built-in preprocessing and/or require explicit cell-type information, which is not straightforward to disable and is often unnecessary for the simple bivariate association testing problem considered in our manuscript. In addition, the available documentation did not provide sufficient guidance. After corresponding with the authors via GitHub and adapting portions of the implementations to ensure applicability to our simulation design, we obtained unstable and/or clearly incorrect outputs (e.g., SpatialCorr had almost 0 power in every case) that we could not reconcile within the scope of this revision. As these issues may reflect our implementation choices or an incomplete interpretation of the software, we do not present these results as a formal comparison at this time. We will continue to investigate these packages in follow-up work.

4. You filter out genes with fewer than 59 total reads (0.2 x number of spots). Can you justify the choice of this threshold and show if results are stable for other thresholds (for example 0.1x or 0.5x)? Since many ligands and receptors are lowly
expressed, is there a risk of losing meaningful biology? Since the dataset has only 293 spots, thresholds can have strong effect.

Response: We thank the reviewer for this comment. Applying expression-based filtering is standard practice in spatial transcriptomics to reduce spurious findings driven by extremely low counts. For example, the MERINGUE pipeline (link) uses a minimum total-read threshold (e.g., min.reads = 100). We emphasize that our filtering is based solely on the total read count per gene and does not depend on the number of spots in which the gene is detected (i.e., we do not exclude genes simply because they are expressed in fewer than a specified number of spots). We agree with the reviewer's general point that, in highly sparse settings, the method may have difficulty reliably estimating a large number of parameters.
Following the reviewer's suggestion, we re-ran the analysis using a more permissive (0.1×) filtering threshold. This yielded 130 additional ligand–receptor pairs in the melanoma dataset (291 pairs in total), of which only 19 were significant. Because our method is applied to each pair independently, all previously identified pairs remained detected.
References
[1] S. Acharyya, X. Zhou, and V. Baladandayuthapani. SpaceX: gene co-expression network estimation for spatial transcriptomics. Bioinformatics, 38(22):5033–5041, 2022. PMCID: PMC9665869.
[2] A. Chakrabarti, Y. Ni, and B. K. Mallick. Joint Bayesian estimation of cell dependence and gene associations in spatially resolved transcriptomic data. Scientific Reports, 14(1):9516, 2024.

[3] S. C. Anderson, E. J. Ward, P. A. English, and L. A. Barnett. sdmTMB: an R package for fast, flexible, and user-friendly generalized linear mixed effects models with spatial and spatiotemporal random fields. BioRxiv, pages 2022–03, 2022.
[4] J. Geweke. Evaluating the accuracy of sampling-based approaches to the calculation of posterior moments. Technical report, Federal Reserve Bank of Minneapolis, 1991.
[5] M. Plummer, N. Best, K. Cowles, and K. Vines. Package ‘coda’. URL http://cran. r-project. org/web/packages/coda/coda. pdf, accessed January, 25:2015, 2015.
[6] S. Banerjee, B. P. Carlin, and A. E. Gelfand. Hierarchical modeling and analysis for spatial data. Chapman and Hall/CRC, 2014.
[7] A. Datta, S. Banerjee, J. S. Hodges, and L. Gao. Spatial disease mapping using directed acyclic graph auto-regressive (DAGAR) models. Bayesian analysis, 14(4):1221, 2019. PMCID: PMC8046356.
[8] Z. T. Luo, H. Sang, and B. Mallick. A Bayesian contiguous partitioning method for learning clustered latent variables. Journal of Machine Learning Research, 22(37):1–52, 2021.
[9] R. Furrer and S. R. Sain. spam: A sparse matrix R package with emphasis on MCMC methods for Gaussian Markov random fields. Journal of Statistical Software, 36:1–25, 2010.
[10] R. J. Lipton, D. J. Rose, and R. E. Tarjan. Generalized nested dissection. SIAM journal on numerical analysis, 16(2):346–358, 1979.
[11] S. E. Neville, J. T. Ormerod, and M. Wand. Mean field variational Bayes for continuous sparse signal shrinkage: pitfalls and remedies. Electronic Journal of Statistics, 8:1113–1151, 2014.
[12] V. Ravikumar, T. Xu, W. N. Al-Holou, S. Fattahi, and A. Rao. Efficient inference of spatiallyvarying Gaussian Markov random fields with applications in gene regulatory networks. IEEE/ACM transactions on computational biology and bioinformatics, 20(5):2920–2932, 2023.
[13] C. M. Carvalho, N. G. Polson, and J. G. Scott. Handling sparsity via the horseshoe. In Artificial intelligence and statistics, pages 73–80. PMLR, 2009.
[14] J. Piironen and A. Vehtari. Sparsity information and regularization in the horseshoe and other shrinkage priors. Arxiv, 2017.
[15] F. Lindgren, H. Rue, and J. Lindstr¨om. An explicit link between Gaussian fields and Gaussian Markov random fields: the stochastic partial differential equation approach. Journal of the Royal Statistical Society Series B: Statistical Methodology, 73(4):423–498, 2011.
[16] D. Makowski, M. S. Ben-Shachar, and D. Lu¨decke. bayestestR: Describing effects and their uncertainty, existence and significance within the Bayesian framework. Journal of open source software, 4(40):1541, 2019.
[17] P. E. Bowden. Mutations in a keratin 6 isomer (K6c) cause a type of focal palmoplantar keratoderma. Journal of Investigative Dermatology, 130(2):336–338, 2010.

em Editorial Manager®

Cookie Preference Center

We use cookies which are necessary to make our site work. We may also use additional cookies to analyze, improve and personalize our content and your digital experience. For more information, see our Cookie Policy.

You may choose not to allow some types of cookies. However, blocking some types may impact your experience of our site and the services we are able to offer. See the different category headings below to find out more or change your settings.

Allow all

Manage Consent Preferences

Strictly Necessary Cookies

Always active

These cookies are necessary for the website to function and cannot be switched off in our systems. They are usually only set in response to actions made by you which amount to a request for services, such as setting your privacy preferences, logging in or filling in forms. You can set your browser to block or alert you about these cookies, but some parts of the site will not then work.

Functional Cookies

These cookies enable the website to provide enhanced functionality and personalisation. They may be set by us or by third party providers whose services we have added to our pages. If you do not allow these cookies then some or all of these services may not function properly.

Performance Cookies

These cookies allow us to count visits and traffic sources so we can measure and improve the performance of our site. They help us to know which pages are the most and least popular and see how visitors move around the site.

Targeting Cookies

These cookies may be set through our site by our advertising partners. They may be used by those companies to build a profile of your interests and show you relevant adverts on other sites. If you do not allow these cookies, you will experience less targeted advertising.

Cookie List

Clear

Apply Cancel

Consent Leg.Interest

Confirm my choices

Powered by onetrust
