# Supplementary material for: SpaceBF: spatial coexpression analysis using Bayesian fused approaches in spatial omics datasets
Source: Gigascience. 2026 Jan 20;15:giag006. doi: 10.1093/gigascience/giag006 (PMC12954175; doi:10.1093/gigascience/giag006)
Supplement: giag006_Reviewer_1_Report_Original_Submission — Satwik Acharyya -- 7/27/2025 [file giag006_reviewer_1_report_original_submission.pdf]

Reviewer Report

Title: SpaceBF: Spatial coexpression analysis using Bayesian Fused approaches in spatial omics datasets

Version: Original Submission **Date:** 7/27/2025

Reviewer name: Satwik Acharyya

Reviewer Comments to Author:

Summary: The manuscript introduces a novel statistical framework for analyzing spa- tially varying molecular co-expression. Leveraging a Bayesian fused modeling approach, SpaceBF estimates both local (location-specific) and global (tissue-wide) co-expression pat- terns, particularly useful for studying cell-cell communication via ligand-receptor interac- tions. The method outperforms traditional geospatial metrics like bivariate Moran's I and Lee's L in terms of specificity and precision. Application of SpaceBF to spatial omics data reveals new insights into molecular interactions across various cancer types, offering a pow- erful tool for spatial omics research. The paper is nicely written, well structured, and great visualizations but I have the following comments.1. The authors missed a couple of key references related to co-expression analysis of spatial omics data such as JOBS (Chakrabarti et al., 2024) and SpaceX (Acharyya et al., 2022). The authors are recommended to include these references in the Introduction Section.2. A method related figure can be included for visual illustration of the method.3. In Melanoma ST data analysis, authors have used the RCTD algorithm (Cable et al., 2022) for cell-type estimation. It seems like the gene expression matrix has been used twice in the whole process: once in case of cell-type estimation and co-expression analysis afterwards. The obtained results can be highly correlated due to multiple uses of the gene expression matrix. It would be great if authors can address this issue.4. In the cSCC ST data analysis, BayesSpace (Zhao et al., 2021) algorithm has been used for spatial region identification. In Figure 2C, cluster numbers are provided only and those are not transferred to spatial regions. It is difficult to make spatial region specific inference without such regional annotation of clusters. The gene expression matrix is used multiple times in this case as well (spatial region identification and co-expression analysis).5. The spatial omcis datasets are sparse in nature. It possible that some these edges may not exist if the molecules are far apart. Authors are requested to justify the use shrinkage prior such as horseshoe rather than spike-and-slab prior.6. While the authors briefly mention about the associated computational costs, it is recommended to include a comparison of the computational costs for different approaches in the simulation studies. This would provide a more comprehensive understanding of the proposed method's efficiency and feasibility. It will be also interesting to see the scalability of the method for large scale datasets.7. To ensure the robustness of the proposed methodology, it is requested that the authors include a detailed sensitivity analysis for the selected priors and parameters.

Level of Interest

Please indicate how interesting you found the manuscript: Choose an item.

Quality of Written English

Please indicate the quality of language in the manuscript: Choose an item.

Declaration of Competing Interests

Please complete a declaration of competing interests, considering the following questions:

- Have you in the past five years received reimbursements, fees, funding, or salary from an organisation that may in any way gain or lose financially from the publication of this manuscript, either now or in the future?
- Do you hold any stocks or shares in an organisation that may in any way gain or lose financially from the publication of this manuscript, either now or in the future?
- Do you hold or are you currently applying for any patents relating to the content of the manuscript?
- Have you received reimbursements, fees, funding, or salary from an organization that holds or has applied for patents relating to the content of the manuscript?
- Do you have any other financial competing interests?
- Do you have any non-financial competing interests in relation to this paper?

If you can answer no to all of the above, write 'I declare that I have no competing interests' below. If your reply is yes to any, please give details below.

I declare that I have no competing interests.

I agree to the open peer review policy of the journal. I understand that my name will be included on my report to the authors and, if the manuscript is accepted for publication, my named report including any attachments I upload will be posted on the website along with the authors' responses. I agree for my report to be made available under an Open Access Creative Commons CC-BY license (http://creativecommons.org/licenses/by/4.0/). I understand that any comments which I do not wish to be included in my named report can be included as confidential comments to the editors, which will not be published.

Choose an item.

To further support our reviewers, we have joined with Publons, where you can gain additional credit to further highlight your hard work (see: https://publons.com/journal/530/gigascience). On publication of this paper, your review will be automatically added to Publons, you can then choose whether or not to claim your Publons credit. I understand this statement.

Yes Choose an item.
