# Supplementary material for: SpaceBF: spatial coexpression analysis using Bayesian fused approaches in spatial omics datasets
Source: Gigascience. 2026 Jan 20;15:giag006. doi: 10.1093/gigascience/giag006 (PMC12954175; doi:10.1093/gigascience/giag006)
Supplement: giag006_Reviewer_2_Report_Original_Submission — Daniel Domovic -- 8/22/2025 [file giag006_reviewer_2_report_original_submission.pdf]

Reviewer Report

Title: SpaceBF: Spatial coexpression analysis using Bayesian Fused approaches in spatial omics datasets

Version: Original Submission **Date:** 8/22/2025

Reviewer name: Daniel Domovic

Reviewer Comments to Author:

Dear authors,I read your manuscript "SpaceBF: Spatial coexpression analysis using Bayesian Fused approaches in spatial omics datasets" with interest.The manuscript presents SpaceBF, a Bayesian method for detecting spatial co-expression between pairs of molecules in spatial omics data. The topic is relevant since new technologies like spatial transcriptomics, mass spectrometry imaging, and multiplex immunofluorescence produce large data but current tools for co-expression are limited. The authors try to solve this gap with a new model and they also test it on real datasets. The paper is technical, but it also gives biological examples, which is helpful for readers.The paper has many strong points. First, the idea to use Bayesian fused horseshoe prior together with MST spatial structure is new and well explained. Second, the authors apply their method on three real datasets and they show interesting biology, for example IGF2-IGF1R relation, keratin isoform consistency, and stromal ECM peptides. Third, I appreciate that the code is open on GitHub. Also, the paper compares with other methods and deals with the common problem of variance-stabilizing transform by modeling UMI counts directly with negative binomial distribution.Overall, the work is clear and well organized, but there are some points where more explanation or clarification would help. In my review I give major and minor remarks that I hope will improve the paper.Major remarks1. Were you worried choosing MST may oversimplify spatial relationships, since many meaningful local neighborhoods may be excluded? Would the results of SpaceBF be significantly different if a different spatial graph, such as kNN, Delaunay triangulation, or kernel-based, was used instead of MST?2. Since MST edges depend a lot on pairwise L2 distances, how stable are the results if spatial coordinates are a little noisy, or if there are tissue registration errors?3. The model puts one molecule as outcome and the other as predictor. Are the co-expression estimates still the same if you switch roles?4. In the Results you mention "FDR < 0.1." Can you explain which method you used for FDR? Also, are the discoveries robust if you change the threshold (for example 0.05 vs 0.1)?5. Do the simulation parameters (lengthscale, slope, dispersion) correspond to realistic biological signal strengths and spatial scales observed in real datasets? Three values of the lengthscale l are considered, l = 3.6, 7.2, 18. Why exactly these values? What does Î½=0.75 mean in terms of effect size? How does l=18 compare to real tissue lengthscales?6. Can you describe runtime and memory for larger datasets, like 10X Visium with 5,000-20,000 spots? Is the current MCMC practical for this scale, or do you think approximate inference (like variational Bayes or INLA) is needed?Minor remark1. How sensitive are the results to the choice of hyperparameters for the Horseshoe prior?2. In the Results you state that keratins "co-express highly, meaning their binding patterns with any specific type 1 keratin should be similar." Please make clear that SpaceBF measures co-expression, not direct binding, so that conclusions are not overstated.3. You mention SpatialCorr and Copulacci, but the comparison was not

successful. Even if parameters were sensitive, I think one short numerical comparison in the supplement would be helpful.4. You filter out genes with fewer than ~59 total reads (0.2 x number of spots). Can you justify the choice of this threshold and show if results are stable for other thresholds (for example 0.1x or 0.5x)? Since many ligands and receptors are lowly expressed, is there a risk of losing meaningful biology? Since the dataset has only 293 spots, thresholds can have strong effect.

Level of Interest

Please indicate how interesting you found the manuscript: Choose an item.

Quality of Written English

Please indicate the quality of language in the manuscript: Choose an item.

Declaration of Competing Interests

Please complete a declaration of competing interests, considering the following questions:

- Have you in the past five years received reimbursements, fees, funding, or salary from an organisation that may in any way gain or lose financially from the publication of this manuscript, either now or in the future?
- Do you hold any stocks or shares in an organisation that may in any way gain or lose financially from the publication of this manuscript, either now or in the future?
- Do you hold or are you currently applying for any patents relating to the content of the manuscript?
- Have you received reimbursements, fees, funding, or salary from an organization that holds or has applied for patents relating to the content of the manuscript?
- Do you have any other financial competing interests?
- Do you have any non-financial competing interests in relation to this paper?

If you can answer no to all of the above, write 'I declare that I have no competing interests' below. If your reply is yes to any, please give details below.

I declare that I have no competing interests.

I agree to the open peer review policy of the journal. I understand that my name will be included on my report to the authors and, if the manuscript is accepted for publication, my named report including any attachments I upload will be posted on the website along with the authors' responses. I agree for my report to be made available under an Open Access Creative Commons CC-BY license (http://creativecommons.org/licenses/by/4.0/). I understand that any comments which I do not wish to be included in my named report can be included as confidential comments to the editors, which will not be published.

Choose an item.

To further support our reviewers, we have joined with Publons, where you can gain additional credit to further highlight your hard work (see: https://publons.com/journal/530/gigascience). On publication of this paper, your review will be automatically added to Publons, you can then choose whether or not to claim your Publons credit. I understand this statement.

Yes Choose an item.
